# Supplementary material for: Pattern recognition in the nucleation kinetics of non-equilibrium self-assembly
Source: Nature. 2024 Jan 17;625(7995):500–7. doi: 10.1038/s41586-023-06890-z (PMC10794147; doi:10.1038/s41586-023-06890-z)
Supplement: Supplementary file 1 — Mathematical and conceptual connections between self-assembly and neural networks; Nucleation model and pattern recognition training; fluorescence readout, shape layouts and simulated structures; DNA sequences; data and analysis of flag and pattern recognition experiments; figs., tables and references. [file 41586_2023_6890_MOESM1_ESM.pdf]

nature portfolio

https://doi.org/10.1038/s41586-023-06890-z

Supplementary information

Pattern recognition in the nucleation kinetics of non-equilibrium self-assembly

In the format provided by the authors and unedited

Pattern recognition in the nucleation kinetics of non-equilibrium self-assembly

Supplementary Information

Constantine Glen Evans, Jackson O'Brien, Erik Winfree, Arvind Murugan

Final published version, compiled on December 26, 2023.

Contents

1 Self-assembly and neural networks **7**
1.1 The Hopfield associative memory perspective . . . 9
1.2 The Boltzmann machine perspective . . . 13
1.3 The place cell perspective . . . 14
1.4 The machine learning and function approximation perspective . . . 17
1.5 The reservoir computing perspective . . . 21
1.6 A compact, robust, and scalable molecular architecture . . . 23

2 Nucleation models and pattern recognition training **25**
2.1 The kinetic Tile Assembly Model and Xgrow . . . 26
2.1.1 Estimates of kTAM parameters for SSTs and double-crossover tiles . . . 28
2.2 Stochastic Greedy Model of nucleation . . . 30
2.2.1 Trajectories . . . 32
2.2.2 Nucleation rate . . . 33
2.3 Simplified models of depletion and winner-take-all effects . . . 37
2.4 Pattern recognition training . . . 41
2.5 Window Nucleation Model . . . 42
2.6 A simple alternative pattern recognition training method . . . 43
2.7 Training and testing image sources and processing . . . 45
2.8 Mixes for pattern recognition experiments . . . 47

3 Fluorescence readout, shape layouts, and simulated structures **49**
3.1 Initial fluorophore design . . . 49
3.2 Final fluorophore design . . . 51
3.3 Shape layouts . . . 53
3.4 Simulated structures . . . 57

Bibliography **59**

4 Sequences **65**
4.1 Unmodified strands . . . 65
4.2 Fluorophore- and quencher-modified strands . . . 81

5 Flag patterns **83**
5.1 Protocols . . . 83
5.1.1 Flag patterns, constant temperature . . . 83
5.1.2 Flag patterns, ramp . . . 84
5.2 Nucleation model summaries . . . 85
5.3 Individual results . . . 86
5.3.1 H flag 1 . . . 86
5.3.2 H flag 2 . . . 87
5.3.3 H flag 3 . . . 89
5.3.4 H flag 4 . . . 90
5.3.5 H flag 5 . . . 91
5.3.6 H flag 6 . . . 92

5.3.7 H flag 7 . . . 94
5.3.8 H flag 8 . . . 95
5.3.9 H flag 9 . . . 96
5.3.10 H flag 10 . . . 97
5.3.11 H flag 11 . . . 99
5.3.12 H flag 12 . . . 100
5.3.13 A flag 1 . . . 101
5.3.14 A flag 2 . . . 103
5.3.15 A flag 3 . . . 104
5.3.16 A flag 4 . . . 106
5.3.17 A flag 5 . . . 107
5.3.18 A flag 6 . . . 108
5.3.19 A flag 7 . . . 109
5.3.20 A flag 8 . . . 110
5.3.21 A flag 9 . . . 111
5.3.22 A flag 10 . . . 113
5.3.23 A flag 11 . . . 114
5.3.24 A flag 12 . . . 115
5.3.25 M flag 1 . . . 117
5.3.26 M flag 2 . . . 118
5.3.27 M flag 3 . . . 119
5.3.28 M flag 4 . . . 120
5.3.29 M flag 5 . . . 121
5.3.30 M flag 6 . . . 123
5.3.31 M flag 7 . . . 124
5.3.32 M flag 8 . . . 125
5.3.33 M flag 9 . . . 126
5.3.34 M flag 10 . . . 128
5.3.35 M flag 11 . . . 129
5.3.36 M flag 12 . . . 130
5.3.37 M flag 13 . . . 131
5.3.38 Uniform concentrations (50 nM) . . . 133
5.4 Plate-level fluorescence data used in Section 5.3 . . . 134

6 Pattern recognition **139**
6.1 Protocol . . . 139
6.2 Nucleation model summaries . . . 140
6.3 AFM shapes and counts summary . . . 141
6.3.1 Counts averaged across co-authors and images, normalized to shapes per 25 µm^2: . . . 141
6.3.2 Counts averaged across co-authors, images and samples, normalized to shapes per 25 µm^2: . 144
6.4 Individual fluorescence and AFM results . . . 145
6.4.1 Hodgkin . . . 145
6.4.2 Avogadro . . . 146
6.4.3 Mitscherlich . . . 147
6.4.4 Hopfield . . . 148
6.4.5 Abbott . . . 149
6.4.6 Moser . . . 150
6.4.7 Horse . . . 151
6.4.8 Anchovy . . . 153
6.4.9 Mockingbird . . . 154
6.4.10 Hazelnuts . . . 156
6.4.11 Apples . . . 157
6.4.12 Magnolia . . . 158
6.4.13 Harom . . . 160
6.4.14 Aon . . . 161
6.4.15 Mbili . . . 163

6.4.16 LetterH . . . 165
6.4.17 LetterA . . . 167
6.4.18 LetterM . . . 168
6.4.19 NoisyApple1 . . . 169
6.4.20 NoisyApple2 . . . 170
6.4.21 NoisyApple3 . . . 171
6.4.22 NoisyHorse1 . . . 172
6.4.23 NoisyHorse2 . . . 173
6.4.24 NoisyHorse3 . . . 174
6.4.25 ObscMagn1 . . . 175
6.4.26 ObscMagn2 . . . 176
6.4.27 ObscMagn3 . . . 177
6.4.28 ObscHazeln1 . . . 178
6.4.29 ObscHazeln2 . . . 179
6.4.30 ObscHazeln3 . . . 180
6.4.31 VarHarom1 . . . 181
6.4.32 VarHarom2 . . . 182
6.4.33 VarHarom3 . . . 183
6.4.34 VarHarom4 . . . 184
6.4.35 VarHarom5 . . . 185
6.4.36 VarHarom6 . . . 186
6.4.37 Uniform concentrations (60 nM) . . . 187

Section 1

Self-assembly and neural networks

Relationships between self-assembly phenomena and classical neural network concepts motivated our work. Pattern recognition by nucleation kinetics in multicomponent molecular self-assembly shares many conceptual features that have been well understood in the context of neural networks and machine learning. However, the self-assembling system is not simply a neural network by another name: in self-assembly there is no exact equivalent of a 'neuron' that performs a weighted linear sum with a threshold. Consequently, understanding the similarities and differences between these alternative implementations can provide insight into the nature of computation in distributed highly interconnected systems.

In this supplemental discussion, we attempt to highlight the similarities while delineating important differences. This section is not necessary for understanding the content of our results, but may be helpful for appreciating the wider context of our work, especially for those who are not already immersed in the machine learning and neuroscience literature. Since we do not rely on advanced modern notions for these comparisons, a useful reference is Hertz, Krogh, and Palmer's "Introduction to the Theory of Neural Computation"[1] (1991). This text is notable for emphasizing connections to physics and biology. More modern texts that focus more on the mathematical and algorithmic perspectives include Bishop's "Pattern Recognition and Machine Learning"[2] (2006) and Goodfellow, Bengio, and Courville's "Deep Learning"[3] (2018). Our aim here is to isolate a few relevant themes and illustrate them with (perhaps over-simplified) examples, thus helping connect the dots between disparate fields, even though the relationships are not always precise.

Connecting the dots between the theory of neural computation and non-neural systems in biology and engineering has a long history. It was a relatively short path from early mathematical formalisms for neural function[4,5] to special-purpose electronic systems implementing neural architectures with discrete components[6] and VLSI transistor physics.[7] A prominent (though not universal) theme in such work was to engineer explicit circuit components for weights (e.g. resistors), summation (e.g. via Kirchoff's current law) and thresholds (e.g. transistors) so as to match the mathematical weighted linear sum and (hard or soft) threshold as developed in the theory. A similar "engineering" approach was used to establish that well-mixed chemistry is capable of mimicking neural computation, first by by Rössler[8] demonstrating how electrical circuits can be converted into theoretical chemical reactions with equivalent dynamics and in particular how excitable "spiking" behavior analogous to conduction in neural axons could be designed in the reaction-diffusion context, and later by Hjelmfelt et al[9] who showed, again theoretically, that a system of coupled enzymatic conversions of metabolites behaved like Hopfield neural networks, where weights correspond to enzyme concentrations, summation is of concentrations, and the sharp threshold arises from substrate interconversion. The general form of these observations was also reflected in theoretical models of genetic regulatory networks, wherein weights correspond to promiscuous transcription factor binding constants, the summation occurs at the probabilistic level to govern the occupancy of active transcriptional apparatus, and the nonlinearity arises from statistical mechanical considerations.[10,11] In the case of cellular signal transduction cascades, Bray[12] argued that despite their dynamics not having the exact form of a traditional neural network, they shared important features such as each unit integrating input from many other elements (perhaps nonlinearly) and having many parameters (such as reaction rate constants) that can tune the overall system function. Phenomenological modeling of genetic regulatory and signal transduction networks argued that, independent of explicit mechanisms, such systems share generic behaviors with neural networks, such as characteristic global dynamics[13] and emergent decision-making.[14] The prospect and potential of synthetic biology, in living cells or in cell-free systems, led to efforts to experimentally demonstrate biochemical analogs of neural networks. Two simplified cell-free systems – formulated as simplified genetic regulatory architectures that use RNA[15–17] or DNA[18–20] molecules as signals and just two or three enzymes (RNA polymerase and RNase, or DNA polymerase, nickase, and exonuclease) to produce

and destroy signals – were developed to implement neural computing units wherein each weight corresponds to a transcription template and thresholding involves competitive hybridization, and have been scaled (with limited connectivity) to about 15 units so far. Enzyme-free DNA strand displacement circuitry has scaled better, from a 4-bit Hopfield network[21] to a 100-bit winner-take-all classifier[22] but still, in existing designs, has required a new molecule specific to each weight in the neural network. These cell-free and enzyme-free biochemical circuits are explicit mechanistic implementations of neural network models, such that a theoretical parameters (weights) for the neural network can be translated into experimental parameters (concentrations) for the biochemical circuit that will, in principle, compute the same function. Cell-free neural networks using the full transcription/translation (TX-TL) system of the central dogma[23] and synthetic metabolic circuits[24] and genetic circuits[25–27] within living cells have not yet scaled beyond 4 units, reflecting the challenges of engineering with complex pre-existing biochemical components.

In contrast to the foregoing mechanistic designs for neural computation in well-mixed chemical reaction networks, the basis for neural computation in molecular self-assembly was not established based on detailed mechanistic correspondences, but rather on general shared principles such as energy landscapes with multiple deep minima, Hebbian learning of interaction strengths, robustness of random wiring, and colocalization as a computational element. To reiterate: we are *not* saying that neural network models and self-assembly models are mathematically identical through a change of variables, or that there is an embedding such that one system can simulate an arbitrary instance of the other, so we make no claims that their range of capabilities coincide precisely. We *are* saying that they exhibit a variety of related phenomena, that similar concepts can be used to design and understand them, and that the natural questions in one domain are likely to stimulate related productive questions in the other domain.

Central to the broader perspective for neural networks is the theme of high-dimensional input to each neural computing unit – perhaps even all-to-all connectivity. In a trained neural network, it may be that most connections are of similar weak magnitude, or it may be that there a few dominant strong interactions that drive the behavior, or (more surprisingly) there can be a few easily-identified strong interactions yet it is the profusion of many weak interactions that actually drives the behavior[28] – that is to say, distinct collective dynamics may arise from the global interaction patterns. Multifarious self-assembly provides a concrete example of similar phenomena in engineered molecular self-assembly. However, the principles behind our work also hold lessons for biology. Molecular biology is often presented as a story of intentional highly-specific interactions between numerous molecular agents. As a consequence, promiscuous interactions between components are often seen as deleterious perturbations that degrade performance; for example, signals in one signaling pathway might leak into another and activate the wrong output or an attempted assembly of the ribosome from 60 different proteins might result in an uncontrolled toxic aggregate of ribosomal proteins. Nonetheless, in other contexts it has been appreciated that many promiscuous interactions may collectively provide a function if the interactions are cooperative, like neural networks.[12] While promiscuous interactions are pervasive and even inevitable[29–31] at the molecular scale, it is not yet well understood in what contexts and to what extent promiscuous interactions can be a blessing for molecular systems. Our work adds to a small but growing list of examples[32–36] of functionality that exploits molecular promiscuity and cannot be understood as deleterious perturbations of a conventional picture with specific interactions.

Beyond biology, our work also provides a concrete example of neural computing principles within physical systems. From this perspective, neural networks can be seen as a special class of algorithms that effectively simulate a many-body system with two key characteristics: disordered interactions between the agents and strong non-linearities. Here we use competitive nucleation between different stable structures of a large collective of interacting molecules to perform high dimensional pattern recognition. But more generally, disordered interactions help sculpt complex decision surfaces in high dimensional input space while non-linearities then map these different regions to different outputs. Thus, the fact that certain physical and molecular systems can have disordered interactions and non-linearities in their collective phenomena suggests that neural network-like behavior might naturally emerge in these systems.

The following technical discussion presumes familiarity with the main paper. Section 1.1 discusses connections to the seminal Hopfield associative memory model for recurrent neural networks, and Section 1.2 presents the generalization to Boltzmann machines. Section 1.3 illustrates a concrete relation between the architecture of our self-assembling system and a real neural system first identified in rodents. Section 1.4 then provides a broader machine learning perspective on the computation achieved by the self-assembling system. Finally, Section 1.5 discusses how our training procedure relates to phenomena underlying reservoir computing.

1.1 The Hopfield associative memory perspective

The Hopfield associative memory is a simple Ising-like model for the memorization of binary patterns and their recall from partial or imperfect information. Given a set of M target patterns (aka **memories**) $\{x^1, \ldots, x^M\}$, each being a vector of N spins so $x^\alpha \in \{-1, +1\}^N$, weights for a neural network are set by a direct **Hebbian learning rule**:

$$w^\alpha_{i,j} = x^\alpha_i x^\alpha_j$$

that implements a "fire together, wire together" principle for memory stimulus α being active that increases the strength of synaptic connections between neurons that are active at the same time. For multiple memories, simple Hebbian learning linearly accumulates these synaptic updates and results in the sum:

$$w_{i,j} = \sum_{\alpha=1}^{M} w^\alpha_{i,j}$$

for $i \neq j$ and $w_{i,i} = 0$.

In addition to coupling between units via $w_{i,j}$, Hopfield networks can include a unit-specific bias term b_i that provides a threshold for activation. Hebbian learning sets $b^\alpha_i = x_i$ and thus $b_i = \sum_\alpha b^\alpha_i$, which is equivalent to standard Hebbian learning of coupling to a special unit that is always clamped on.

Dynamics are as follows. Starting from an arbitrary initial state x, updates proceed one-at-a-time asynchronously according to **linear threshold unit** semantics:

$$x_i \leftarrow \operatorname{sign}\left(\sum_j w_{i,j} x_j + b_i\right) .$$

The dynamics respect an **energy function**

$$E(x) = -\frac{1}{2}\sum_{i,j} w_{i,j} x_i x_j - \sum_i b_i x_i \tag{1.1}$$

that is strictly decreasing every time a unit changes state, and is bounded below so that convergence to a **stable local minimum** is guaranteed.

This implies that, if the target memories are the only stable local minima, then initializing the network with an arbitrary pattern will result in convergence to one or another of the target memories – which we may call **associative memory recall** because there is a sense in which the "most similar" memory is identified. This is guaranteed to be the case if there is just a single memory (along with its exact inverse if biases are not used) that corresponds to a single basin in the energy landscape. As more memories are learned, the Hebbian rule results in additional basins being dug into the landscape, which do not significantly overlap so long as the memories are sufficiently distinct. Thus, the dynamics can be characterized as having **point attractors**. However, as the number of memories grows, for a fixed N, it becomes possible and even likely that **spurious memories** (local minima that are not target memories used in training) will appear and even that target memories will become unstable (perhaps defaulting to a similar spurious memory nearby).

Problems with unstable target memories and spurious memories are exacerbated by correlations between the target memories, for example, if two memory patterns are identical for a substantial number of bits. For the case of **uniform random memory** patterns, which will be statistically near-orthogonal, it is possible to determine a **capacity** for how many memories the Hopfield network can store reliably, depending on criteria for "reliable": if a few bit-errors in recalled memories can be tolerated, then $M \leq 0.138N$ memories can be stored with high probability; for perfect recall with high probability, the limit is $O(N/\log N)$. That said, improvements on the basic Hebbian learning rule are possible, for example using the Perceptron learning rule that only changes weights when they are not already sufficient for correct behavior, or more advanced global linear algebra methods to set weights analytically.

Parallels with multifarious self-assembly: In the original work on multifarious self-assembly[37] the M target shapes could be considered **uniform random memories** – the same N tiles arranged randomly within a square. Promiscuous pairwise binding energies are determined by a **Hebbian learning rule** we call "get together, glue together", which is analogous to the familiar "fire together, wire together". Without postulating a specific molecular mechanism, we examine the consequence of any process whose net effect over time is to increase the binding affinities of molecules that have been brought together into particular geometric arrangements by external circumstances. (For example, consider membrane proteins in a fluid mosaic that bind to an external surface exhibiting

a particular spatial arrangement of ligands, which thus re-arrange the membrane proteins correspondingly. Each environmentally-driven arrangement would be called a "memory", and the "get together, glue together" rule would result in certain membrane protein species developing specific affinities for each other, by the unknown hypothetical mechanism. The consequence is that after learning, the membrane proteins would tend to associate with each other in geometrical arrangements matching or similar to the memories, even in the absence of environmentally-driven stimuli.) Formally, for *in-silico* training, the Hebbian self-assembly learning rule is applied to each memory α and then summed over memories,

$$J^{\alpha,\delta}_{i,j} = \begin{cases} E_0 & \text{if tile } i \text{ is the } \delta\text{-neighbor of tile } j \text{ in memory } \alpha, \text{ for direction } \delta \in \{north, east, south, west\} \\ 0 & \text{otherwise} \end{cases}$$

where $E_0 > 0$ is a standard interaction strength; thus, in total,

$$J^{\delta}_{i,j} = \sum_{\alpha=1}^{M} J^{\alpha,\delta}_{i,j} \quad .$$

(The main text of the original work[37] oversimplifies this equation by ignoring orientations; here we are following the explanation from equations S14 and S15 of the SI, which is necessary for correct function.) The resulting interaction matrix can potentially contain entries of size $2E_0, 3E_0, \ldots$ for i, j that are neighbors in multiple memories. As uniform binding energies yields better performance, this matrix was 'clipped',[37] $J^{clipped,\delta}_{i,j} = \min(J^{\delta}_{i,j}, E_0)$, giving a Perceptron-like learning rule that avoids reinforcing interactions that appear in multiple stored memories. Thus, the interaction matrix is essentially binary, with energies being either 0 or E_0 based on whether the tiles should interact or not interact. In this model, it is assumed that each of the N molecules are capable of being adjusted to have **arbitrary promiscuous interactions** with any chosen subset of the other molecules; i.e., there are no constraints on which interaction matrices are considered to be implementable.

DNA implementation of interactions derived from Hebbian learning: Because our DNA tile architecture relies on perfect Watson-Crick complementarity for matching domains, we cannot use this Hebbian rule directly: only interaction matrices that respect a certain transitivity constraint can be directly implemented. For example, if the "north" of tile i can bind to the "south" of tiles k and l, and if the "south" of tile k can bind to the "north" of tiles i and j, then by Watson-Crick transitivity it must also be that the "north" of tile j can bind to the "south" of tile l, even though this binding may not be dictated by the Hebbian learning rule (Figure S1.1). More generally, with M shapes, interior tiles will have M (probably distinct) neighbors in each direction δ, all of whom will have to have the same domain sequence, leading to a percolation-like system of constraints that force too many (possibly all) domain sequences to be identical or complementary.

Designing a multifarious DNA system based on Hebbian learning is nonetheless possible, with a slight change of perspective. Consider M shapes on a square lattice, superimposing a black and white checkerboard pattern, and suppose that each shape has N black squares. These will be the shared tiles, and all their $4N$ domains will be distinct. In each shape, we randomly assign each of N shared tiles to the N black squares so that each shared tile appears in M locations, and we introduce a new unique tile for each white square, whose domains are determined by its neighboring black tiles. Thus, its black and white squares are balanced, there will be N shared black tiles and MN shape-specific white tiles, utilizing $4N$ binding domains. In this construction, we can consider the white tiles to implement a form of Hebbian learning that "memorizes" the M shapes based on the shared-tile adjacency interactions[1]. This construction is called the "simple checkerboard" in Extended Data Fig. E3, which also discusses

[1] Beyond noting that the black shared tiles and white shape-specific tiles of the checkerboard construction could correspond to pattern species and weight species, respectively, required to implement the results of a conceptual Hebbian learning analog, the checkerboard construction also suggest hypothetical mechanisms that could physically implement the learning process itself at the molecular level. In this footnote, we loosely consider two such hypothetical implementations that could lead to "get together, glue together" dynamics. The first would be an explicit direct mechanism in which some proximity-directed ligation or synthesis reaction[38,39] creates weight species W_{ij} when pattern species X_i and X_j have been brought near each other by environmentally-driven events. For example, there might be a full set of single-domain oligonucleotides that have been activated so as to covalently link with each other at a very slow rate that is accelerated if they are bound to adjacent SST tiles (the pattern species). While the resulting two-domain "tiles" (the synthesized weight species) would not have the standard four-domain SST format, they would still be likely to stabilize a multifarious set of shapes that geometrically arrange the shared pattern species similarly to the environmentally-driven arrangements. A conceptual mechanism of this type is illustrated in Extended Data Fig. E1. The second hypothetical would be an indirect mechanism that nonetheless also ends up stabilizing a multifarious pattern of binding affinities. In this case our pattern species will be arbitrary, perhaps proteins, and there will be no weight species; promiscuous binding affinities between the protein pattern species will be evolved to stabilize the environmentally-driven geometrical arrangements. For example, Hochberg et al[40] have argued that evolution naturally stabilizes and entrenches hydrophobic interactions between proteins in multicomponent complexes, as a consequence of the need to avoid harmful aggregation. If the relevant proteins participate in multiple distinct multicomponent complexes, as documented by Sartori and Leibler,[41] a multifarious system could effectively be learned by evolution. Such approaches could be compared to prior approaches to brain-like learning in DNA computing.[42–44]

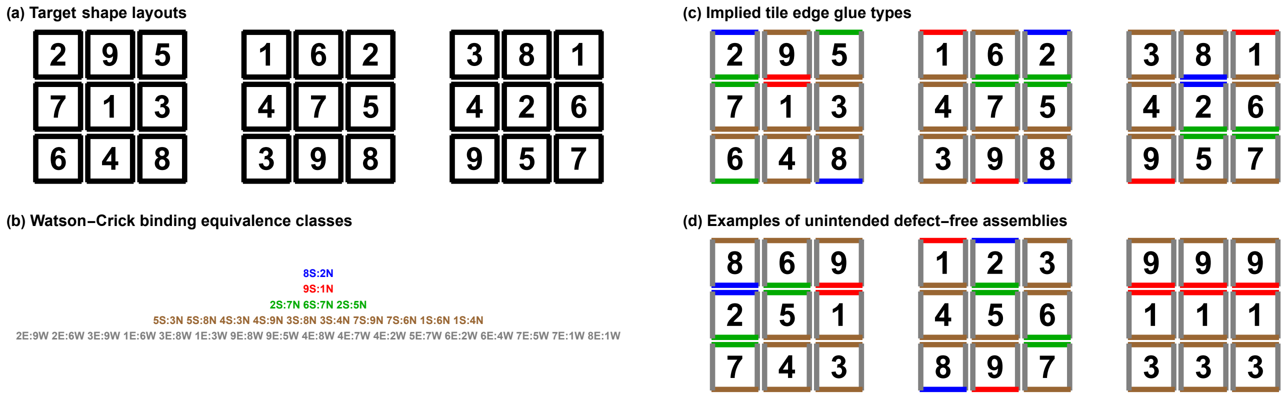

Figure S1.1: The non-implementability of arbitrary tile layouts with DNA tiles and Watson-Crick binding. **(a)** A randomly-chosen multifarious set of three 3×3 shapes using 9 tile types. **(b)** Equivalence classes of glues dictated by the layout. If a north-south pair (e.g. tile 5 being above tile 3, denoted 5S:3N for the glues that must match) is in an equivalence class, then so must any other pair that appears in any shape and contains either of the two glues. Here, this leads to four north-south equivalence classes, but — remarkably, due to full percolation of constraints — just one east-west equivalence class. **(c)** Each (color-coded) equivalence class will correspond to a specific glue type (i.e. a Watson-Crick complementary pair of DNA domain sequences). Together they specify the maximally-distinct design of tiles consistent with the layout. **(d)** The percolation of transitive Watson-Crick binding allows tiles to be adjacent that were not adjacent in any of the three target shape layouts. This gives rise to alternate possible layouts – not only 3×3 but also larger assemblies that are not shown.

an improvement ("guarded edges") that ensures shared tiles on the boundary of a shape will also be on a similar boundary in other shapes, so that the boundary domains of all completed shapes can be implemented in DNA using minimally-interacting poly-T sequences that reduce molecular aggregation. Our actual DNA implementation used a more systematic algorithm to optimize self-assembly robustness to rigorously ensure a proofreading self-assembly property, which is only statistically ensured by the checkerboard construction. (Note that while the checkerboard design ensures that tiles can bind to each other if and only if they are adjacent in one of the target memory shapes, our optimization does not ensure that property, and therefore the transitivity of Watson-Crick binding may give rise to additional possible interactions (see Figure S3.3). Because of proofreading, these interactions do not induce assembly errors. The putative advantage of the optimization is that fewer DNA domain sequences are needed – 698 versus the 992 that a checkerboard design would entail – and thus sequence design can ensure more uniform binding energies and better orthogonality.

Energy landscape: Regardless of how the tile set is obtained, the tiles and their binding energies induce an **energy landscape** that governs the self-assembly process. In our idealized model, the chemical potential for an assembly A is

$$G(A) = -\sum_{\delta} \sum_{\delta\text{-neighbors } i,j \text{ in } A} J^{\delta}_{i,j} - \sum_{\text{tiles } i \text{ in } A} RT \ln \frac{[t_i]}{u_0} = -\frac{1}{2} \sum_{p,p'} \sum_{i,j} J^{\delta(p,p')}_{i,j} x^i_p x^j_{p'} - \sum_p \sum_i \Theta_i x^i_p \tag{1.2}$$

for binary variables $x^i_p \in \{0, 1\}$ that indicate position p in assembly A is occupied by tile type i, with $\delta(p, p')$ being the relative orientation of neighboring positions p and p' or else zero, for which $J^0_{i,j} = 0$, and with $\Theta_i = RT \ln \frac{[t_i]}{u_0}$ being the concentration-dependent cost of tile addition. (Typically, $J^{\delta}_{i,j} \geq 0$ while $\Theta_i < 0$, although this latter depends on the reference concentration e.g. $u_0 = 1$ M.) This can be seen as a quadratic (pairwise) energy function like the Hopfield model with biases, with the coupling energies J playing the role of the synaptic weights w and the (inverted) chemical potentials Θ playing the role of the biases b. Additionally, however, the self-assembly system must observe constraints on x^i_p imposing that at most a single tile type can be in a given location, and that the assembly remains as a single connected component. From the perspective of a single assembly growing in a solution where tile concentrations are unchanging, thermodynamically favorable tile additions correspond to downhill steps in this energy function. Ideally, the Hebbian learning principle would ensure that each **local energy minimum** corresponds to the correct assembly of one of the target memorized shapes – and this is almost true. However, "off-pathway" assembly such as the chimeric structures illustrated in Extended Data Fig. E3 can result in even lower energies. The "checkerboard with guarded edges" design introduces a large barrier to the formation of chimera by enforcing that tiles on the boundary of each shape present non-binding domains on the outside, while

the optimized design of the experimental system additionally ensures a local proofreading property during growth. For such designs, it's reasonable then to assume that the assembly process remain "on-pathway" with respect to growing one of the target memorized shapes, and with that restriction (together with there not being too many shapes to be memorized), the dominant energy basins are indeed the target memories — that is, they are **point attractors**.

With this restriction, thermodynamically favorable tile additions have a direct correspondence to a neural **linear threshold unit**. To see this more clearly, note that the energy landscape assumes a simpler form when the assembly A is a (not necessarily proper) subset of one of the memorized shapes, $1 \leq \alpha \leq M$. For well-designed multifarious systems such as ours, where no multi-tile subassembly of a memorized shape is also a subassembly of another memorized shape and where proofreading largely prevents assembly pathways that form chimeras and other erroneous assemblies, the simpler form is sufficient to treat on-pathway processes. In this case, since we are assuming that no tile occurs more than once in a given shape, its position in the shape is implied and we only need the binary variables x^i that indicate tile i is present. For the same reason, we can use a shape-specific coupling energy $J^{\alpha}_{i,j}$ that is zero except for tiles that, in α, are adjacent. Thus, the chemical potential for assembly A that is a subassembly of shape α is:

$$G(A) = -\frac{1}{2}\sum_{i,j} J^{\alpha}_{i,j} x_i x_j - \sum_i \Theta_i x_i \tag{1.3}$$

which has the exact form of the Hopfield model (eq. 1.1 with $w_{i,j} = J_{i,j}$ and $b_i = \Theta_i$) except that the coupling matrix depends on which shape is growing. Furthermore, the dynamics is not identical to the Hopfield model: there, any neuron could potentially flip state at any time, whereas in multifarious self-assembly, the only allowed attachments are positions on the edge of the assembly, as it must remain connected. (Reversible self-assembly, in which tiles sometimes detach, would have a similar correspondence with the Boltzmann machine perspective discussed below.) That said, when a tile does attach, the energy change is the exact same linear weighted sum as in the Hopfield model:

$$\Delta G(x_i \text{ turns on}) = -\left(\sum_j J^{\alpha}_{i,j} x_j + \Theta_i\right)$$

which is downhill exactly when the sum is positive. (If a tile detaches, the energy change is the opposite.) This is interesting because there is no explicit molecular representation of the summation process (unlike well-mixed biochemical neural networks where distinct molecules and reactions are employed for each multiplication, addition, and thresholding step [15, 21, 22]) – rather, the summation is implicit in the energy landscape.

Successfully trained multifarious self-assembly performs **associative memory recall** from an initial seed assembly by taking a series of downhill (and occasional uphill) steps until a deep local minimum is obtained, corresponding to a completed shape. (Note that the potential for arbitrarily large self-assembled structures, such as tubes or repeated chimeric errors, implies that there could be infinitely many x^i_p in a full model of the physics, so our metaphor only applies if we restrict the size by fiat.)

Our pattern recognition experiments do not involve seed assemblies; instead, concentration patterns are presented by setting biases Θ_i in the above energy functional. (Hopfield models can also be used in such a modality, with inputs presented as biases rather than initial conditions for firing rates.) In this modality, the self-assembly model shows **robustness to noise** in pattern recognition, much like a Hopfield model. For example, if a small fraction of tiles have perturbed concentrations, nucleation rates are not strongly affected unless all of those tiles are strongly colocalized. Consequently, pattern recognition is robust, especially to random 'speckle' noise.

The **network of interactions** between species in the self-assembly model reflect the 2-dimensional (2D) geometry of structures. Each species potentially interacts with $\sim 4M$ other species where M is the number of memories stored. A subset of the connections (e.g., blue in Extended Data Fig. E1) will form a 2-dimensional lattice for a specific spatial ordering of the species with the other connections (red in Extended Data Fig. E1) being long range. An alternative ordering of species will reveal that the red connections also form a 2D lattice. In contrast, Hopfield Associative memory is typically based on fully connected networks where each neuron has $O(N)$ connections where N is the number of neurons; in fact, restricting Hopfield associative memories to a finite lattice does not allow for storing an extensive number (i.e., growing linearly with N) of memories.[45,46]

The concept of a **capacity** is a novel but natural question about self-assembly inspired by the Hopfield associative memory connection. While earlier work has explored limits on function due to non-specific interactions,[47–51] here, promiscuous interactions are not failures of design or oddities of evolution. Instead, promiscuous interactions enable alternate functions. Nevertheless, these interactions needed for other functions do get in each other's way and set a capacity on the number of stored memories. The capacity for self-assembly was shown to be $\sim N^{(z-2)/z}$ where

z is the coordination number ($z = 4$ for our 2D structures) and N is the number of distinct molecular species; this scaling is lower than the linear $\sim N$ scaling of fully connected Hopfield models.

A **phase diagram** for the self-assembly system[37] as a function of temperature and the number of memories M reveals three phases, similar to the Hopfield model: a successful associative memory phase (low temperature, low M), a dissolved-in-solution phase (high temperatures), and a spin glass-like phase with numerous spurious memories (low temperature, large M). The topology of the relative placement of the phases is distinct from the Hopfield model but resembles the place cell network model discussed below.

Finally, self-assembly plays out in **real space** which has no analog in the Hopfield model. Each molecular species might be present at copy number $\sim 10^{10}$ in a 10 μL sample but each neuron is present only once in a neural network. Thus, molecules carry out the same pattern recognition computation in parallel, with interactions only through depletion effects described in the discussion of winner-take-all selection in Section 2.3. As a consequence, in self-assembly, multiple distinct nucleation events will typically grow into distinct structures in different parts of real space in a test tube. In contrast, multiple parallel 'nucleation' events amongst different neurons in a Hopfield model (more precisely, in place cell models described below) will interact strongly and must either merge or compete with each other.

1.2 The Boltzmann machine perspective

Boltzmann machines generalize Hopfield networks from "temperature-zero" strictly downhill steps that define memories as attractor basins, to "finite-temperature" dynamics that define a probability distribution over the state space. We now have (ignoring bias terms for simplicity):

$$P(x) = \frac{1}{Z} e^{-E(x)/T} \qquad \text{where} \qquad Z = \sum_x e^{-E(x)/T}$$

as the equilibrium for a stochastic dynamics

$$x_i \leftarrow 1 \text{ with probability } \tfrac{1}{1+e^{-\Delta E/T}} \text{ where } \Delta E = E(x|_{x_i=-1}) - E(x|_{x_i=+1}) = 2\sum_j w_{i,j}\, x_j$$

that involves both uphill and downhill steps that follow detailed balance with respect to the energy function. Given known values of some of the x_i, such a network can infer the exact conditional probability distribution $P(x|v)$ by "clamping" the known units v, i.e. just never updating them. Importantly there is a learning rule for adjusting the weights $w_{i,j}$ so as to approximate a target distribution that is given by examples. Considering the case where the target distribution $Q(v)$ is specified on a given subset of the variables x, we can consider the network to define $P(v) = \sum_h P(hv)$ where $x \equiv hv$ by concatenation and thus v represent the "visible" units and h represent the "hidden" units. More hidden units allow Boltzmann machines to approximate more complex probability distributions; remarkably, the learning rule (though not computationally efficient) is very simply expressed as a combination of Hebbian learning during a "wake" phase where the visible units are clamped to samples from $Q(v)$, balanced by anti-Hebbian unlearning during a "sleep" phase during which no units are clamped:

$$\frac{dw_{i,j}}{dt} = \langle x_i x_j \rangle_{Q(x)} - \langle x_i x_j \rangle_{P(x)}$$

where $Q(x) = Q(v)P(x|v)$, and this simple expression exactly corresponds to gradient descent minimization of the relative entropy of $Q(v)$ and $P(v)$, i.e.,

$$\frac{dw_{i,j}}{dt} = -\frac{\partial D_{KL}(Q||P)}{\partial w_{i,j}}$$

where $D_{KL}(Q||P) = \sum_v Q(v) \ln \frac{Q(v)}{P(v)} = \sum_x Q(x) \ln \frac{Q(x)}{P(x)}$ is the relative entropy, aka the Kullback-Leibler divergence.

The result of this wake/sleep phase alternation between Hebbian and anti-Hebbian learning is that Boltzmann machines fail to suffer the catastrophic failure experienced by Hopfield networks when attempting to store too many memories. Instead, Boltzmann machines transition naturally between inducing an energy landscape with **point attractors** (when there are relatively few distinct memories in the training set) and one with **continuous attractors** (when the training set contains extensive variations, such as images of an object seen from many angles). The continuous attractor may have any dimensionality, depending on the nature of variation in the training data. Because the Boltzmann machine state space is discrete, we are using the term "continuous attractor" to refer a

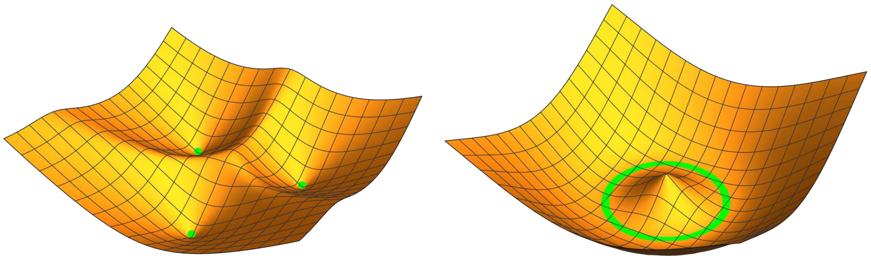

Figure S1.2: An energy landscape with three point attractors (left) and an energy landscape with a continuous line attractor (right).

contiguous set of states that may be easily explored by the Boltzmann machine's random-walk stochastic dynamics at the given temperature; i.e. there are no high-energy barriers disconnecting the set of reasonably-high-probability states. See Figure S1.2.

The relevance to self-assembly begins with statistical mechanics at finite temperature, in contrast to the Hopfield network's zero-temperature dynamics. We do not have a general interpretation of multifarious self-assembly in terms of generative probability distributions, however, so that is an open question. Further, we are not aware of a connection between the elegant Boltzmann machine learning rule and any method for design of molecular self-assembling systems – another avenue to explore. That said, some key features of multifarious self-assembly have direct interpretations – and were inspired by – a neural architecture for so-called "place cells" in rodents, as described in the next section, where the Boltzmann machine perspective provides an elegant way to articulate a toy model. Furthermore, we will see that while the energy landscape of multifarious self-assembly is akin to the point attractors of Hopfield associative memories, the structure of the kinetic barriers relevant to nucleation and pattern recognition is akin to the continuous attractor structure of place cell models.

1.3 The place cell perspective

Place cells and grid cells were discovered by John O'Keefe, May-Britt Moser, Edvard Moser and colleagues[52,53] to be key components of the rodent brain's navigational system. Parts of the computational architecture they discovered re-appear in the principles of multifarious self-assemnbly.[37,54] We review those connections here.

Let us begin with a story, adapted from Hopfield's musings about place cells.[55] You are recording from a grid of electrodes in the hippocampus of an awake behaving rat as it explores a small room or a maze, which as illustrated in Figure S1.3 is in the shape of an H. You find that some neurons don't fire at all, but most neurons fire every now and again, stochastically – and each one seems to prefer a particular location in the maze. Plotting where the rat was when neuron i fired, you see that it fires most frequently near position p_i^H and that its firing rate decreases with the rat's distance from that location. So, having computed the mean position p_i^H for each neuron, you can now re-examine your data (or analyze newly-taken data) at any given instant in time, plotting the positions p_i^H for all neurons that fired during that instant. This will give a scatter of points around the true position of the rat: a **population code** for where the rat is. A neuron elsewhere in the rat's brain could look at this population of neurons in the hippocampus, and know where the rat is, and perhaps perform other computations based on the rat's position encoded this way.

An interesting thing occurs when you pick up the rat and drop it in a new maze, which perhaps has the shape of an A. The same electrodes are still recording from the same neurons. To encode where the rat is in A, will the neurons that responded while in H now be silent, and the neurons that were silent in H now be active and encode positional information in A? What if we were to drop the rat into yet another environment, M? In fact, what you see is that most of the same neurons, as well as some of the previously-silent ones, are active in A, but the positions that they encode for are apparently randomly distributed in the new maze. Let's call these positions p_i^A. So this means, if the rat is wandering around in H, at any given moment the firing neurons can have their p_i^H coordinates plotted, and they will form a **roughly colocalized clump** in the H maze; but if the same firing neurons are plotted according to p_i^A in the A maze, they will be randomly scattered all over the place.

Consequently, the same neurons can underlie population codes for the rat's position in H, the rat's position in A, and even the rat's position in M. That is, neurons elsewhere in the brain can easily compute not only "If I am somewhere in H, where exactly am I?" and "If I am somewhere in A, where exactly am I?" and "If I am somewhere in M, where exactly am I?" just by averaging the associated positions for each neuron that's firing, but they can

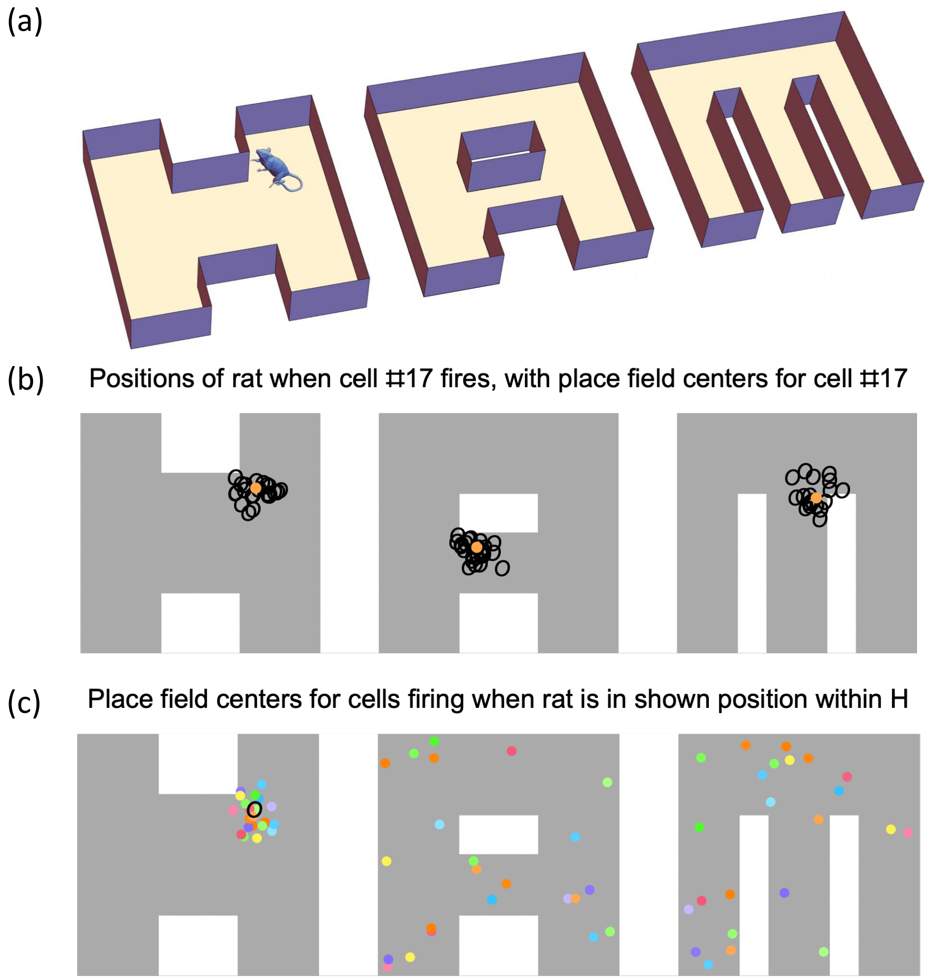

Figure S1.3: Place cells in rat hippocampus. (a) A rat may be placed in one of three mazes, which it explores as an array of electrodes records the activity of a few hundred neurons. (b) If we record the location of the rat every time that a certain cell fired (black circles), we see that it has a selective "place field" within each maze (center plotted as orange dot). (c) If at a given instant we look at which neurons are firing, we see that those neurons' respective place fields (colored dots) are colocalized near each other within the maze the rat is exploring (at position indicated by black circle), but they are distributed randomly in the other mazes.

also easily compute "Am I in H?" and "Am I in A?" and "Am I in M?" by considering the pairwise activity of cells that are nearby in one map or another. The rat is likely to be in H if the firing neurons are roughly colocalized, anywhere in H.

This is a **pattern recognition problem**: given the set of neurons currently firing, which maze is the rat in? The answer comes from examining how the neurons' associated positions are colocalized, or not, in each of the candidate mazes.

The usefulness of this computational architecture comes from the fact that, obviously, an animal needs to be able to navigate through many environments over its lifetime, and it would seem to be wasteful to have a neuron that only fired in exactly one position in the entire world, say three inches to the left of the stove in your grandmother's kitchen. Utilizing a randomized map between sensory perceptions (which in their entirety might be unique to a position in the world, but which considered partially will recur in many environments) provides a way for the same neurons to underlie populations codes in different environments based on the effective orthogonality of colocalization vs scattered activity.

Standard Boltzmann machine learning serves to illustrate how such place cell maps could function in a toy model. We consider N neurons in the hippocampus; the rest of the brain, which provides input to the hippocampus or reads the output of the hippocampus, is not explicitly modeled as a neural network – but the input driving the

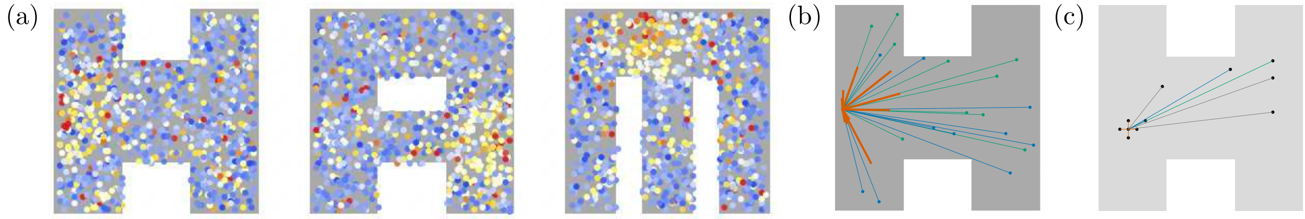

Figure S1.4: Visualization of synaptic weights learned by a Boltzmann machine as described in the text. **(a)** A single cell i was chosen, and for each environment, the weight to cell j was plotted as a point at the center of cell j's receptive field in that environment. The color scale, from strongest to weakest, is red-yellow-blue. Cells with nearby place fields in any environment will tend to have the strongest weights, resulting in a cloud of bright dots near cell i's receptive field (due to colocalization within this maze) along with a scattering of occasional bright dots randomly and uniformly throughout the rest of the maze (due to colocalization with respect to another maze). **(b)** Synaptic weights are classified into those between neurons that are closest to cell i in maze H (red), those closest to cell i when in maze A (green), and those closest to cell i when in maze M (blue). The 10 strongest synapses of each type are shown. **(c)** Multifarious self-assembly has an analogous interaction pattern, except that each cell will have at most four local neighbors in each environment, and no inhibitory long-range interactions. This is illustrated for tile S291: a line is drawn to every other tile in shape H that it can bind to (via either its N, E, S, or W sides) using color red for tiles that are adjacent in H, green for tiles adjacent in A, and blue for tiles adjacent in M. Gray is used for tiles that are not adjacent in any shape, but for which binding ensues from the transitivity of Watson-Crick binding. (Also c.f. Extended Data Fig. E1, where tile binding affinities due to colocalization within the same shape are shown in one color, while tile binding affinities due to colocalization with respect to another shape are shown in another color). See Figure S3.3 for a representation of the full SHAM tile wiring diagram.

hippocampus will be given as training data. Our perspective (appropriate for Boltzmann machine models) is that the purpose of our N neurons is to encode a probabilistic generative model that matches the distribution of the training data. That is, in the "wake" phase, the model rat wanders around randomly inside one of the mazes, with the hippocampal neurons being driven by the environment. Specifically, each neuron is given a fixed-for-all-time random preferred location in each maze, p_i^H, p_i^A, and p_i^M, and when the rat is at position p in maze H, neuron i fires with Gaussian probability $\mathcal{N}(p_i^H, \sigma)$, i.e. it is driven by sensory perception. During the "sleep" phase, neurons fire according to Boltzmann machine stochastic dynamics, as described above, based on the current weights $w_{i,j}$ between the hippocampal neurons. Learning occurs by slow Hebbian weight changes during the wake phase and slow anti-Hebbian weight changes during the sleep phase, exactly as in a classical Boltzmann machine. After sufficient learning, the free-running (sleeping, dreaming) network encodes a probability distribution much like a **continuous attractor**: high probability states are those in which the firing neurons are clumped with radius roughly σ in either H, A, or M, centered around any point within the continuous maze area. Due to the stochasticity of firing, the clump randomly moves around the maze, occasionally decohering and "teleporting" to become clumped in a different maze. Looking at the learned weights, we see that they are strongest between neurons whose receptive fields are near each other (in any maze), but also with negative interactions well balanced such that the clump of co-activated neurons does not grow larger than the typical size during the wake phase (Figure S1.4). Roughly, we can describe the results of Boltzmann machine learning as:

$$w_{i,j}^{\alpha} \approx \begin{cases} \text{high} & \text{if } || \, p_i^{\alpha} - p_j^{\alpha} || < \sigma \\ \text{negative} & \text{otherwise} \end{cases}$$

$$w_{i,j} = \sum_{\alpha} w_{i,j}^{\alpha}$$

where $\alpha \in \{H, A, M\}$ specifies an environment.

The value of this behavior – reproducing the probability distribution of the training data – comes from how a Boltzmann machine can use the distribution to perform inference based on partial knowledge: clamping (driving) neurons with known values and letting the others evolve by stochastic dynamics generates exactly the conditional probability distribution. Suppose the rat is in a perceptually challenging environment, such that only a fraction of hippocampal neurons are driven by the environment, and some neurons are erroneously active. With the environment clamping these neurons but the internal stochastic dynamics controlling the activity of the environmentally-unconstrained neurons, generation according to the conditional probability distribution will infer a highly probable "fleshed out" population code that other parts of the brain can read in order to infer which maze the rat is in, as well as where within the maze the rat is – whereas without the inference, the poor-quality directly stimulated activity may not be sufficient.

While this toy model is oversimplified even with respect to other rudimentary models of hippocampal place cells,[55,56] it serves well as a reference point for considering natural parallels to pattern recognition by multifarious self-assembly. Here, each tile corresponds to a hippocampal neuron; its position within the self-assembled shapes H, A, and M correspond to its receptive fields in those mazes; and its initial concentration corresponds to the (possibly low-quality) environmental stimulation. The binding interactions between neighboring tiles correspond to weights between hippocampal neurons with neighboring receptive fields (although the neurons may have longer-range interactions). Nucleation and self-assembly of some low-concentration tiles together with high-concentration tiles corresponds to formation of a clump of active neurons during inference that activates neurons that were not environmentally driven.

We can now clearly see how multifarious self-assembly shares aspects both of Hopfield associative memories based on **point attractors**, and of Boltzmann machine pattern recognition based on **continuous attractors**. The energy landscape for assemblies in a multifarious system has point-attractor basins; in our system these correspond to the three shapes H, A, and M, which are our "memories". In contrast, the energy barriers for nucleation of assemblies in a multifarious system share the structure of place cell continuous attractors; the set of critical nuclei, closely corresponding to the set of "flag" patterns that stimulate nucleation due to colocalization of high-concentration tiles, form a continuous set. In other words, the thermodynamics of self-assembly has point-attractor structure reflected in the energy basins, while the kinetics of self-assembly has continuous-attractor structure reflected in the nucleation barriers. Note that Figure 1 of the main paper illustrates the three-basin point-attractor nature of the energy landscape, but does not illustrate the continuous-attractor nature of the multifarious nucleation pathways: while two possible nucleation and growth pathways are illustrated for the green shape, a more realistic illustration would include a whole continuous space of possible nucleation and growth pathways.

That said, the analogy with place cells is far from exact. A notable difference is that in self-assembly, once seed has formed, the associated shape will grow to completion – whereas for a rat in a fixed position in the maze, a clump of neurons will remain firing but will not grow to include neurons that have place fields within that maze, due to long-range inhibitory connections. Consequently, unlike the free-running neural network model of place cells, which "dreams" of moving throughout the maze as the clump of currently-active neurons stochastically diffuses, the self-assembly system as explored here does not have a corresponding "mental exploration" behavior.[54] It is natural to ask what physical modification to the self-assembly process would correspond better to "mental exploration": it would have to be an assembly that stays roughly the same size, growing in some places while shrinking in other places, thus at each moment remaining a "clump" of tiles of roughly constant size, but positioned in different places within the full shape. In principle, could the necessary feedback to limit assembly size come, for example, from depletion of a limited shared resource that all tiles consume/release upon assembly/disassembly, or alternatively, from accumulated strain due to geometrical frustration?[57,58] This is meant as an example of the kind of novel question that arises from serious consideration of the metaphors between neural networks and molecular self-assembly.

1.4 The machine learning and function approximation perspective

A general framework for **supervised learning** considers training a parameterized **function class** $f(x;\theta)$ to minimize a **cost function** $\mathcal{L}(D;\theta)$ on a **training set** of data D, with the objective of performing well on out-of-sample data as assessed by an independent **test set** D'. A common choice for the function class is a multilayer neural network with a certain number of input units, hidden units, and output units. In a basic set-up, D and D' are independent identically distributed (iid) samples from an unknown probability distribution over input-output pairs, and $\mathcal{L}$ is the mean squared error with respect to a target function, e.g.,

$$\mathcal{L}(D;\theta) = \frac{1}{|D|} \sum_{(x^\alpha, y^\alpha) \in D} \| f(x^\alpha;\theta) - y^\alpha \|^2 \ .$$

Attempts to find a value θ^* that performs well (finding the global minimum, or even a guaranteed local minimum, is often too much to hope for) may proceed by gradient descent, hill-climbing, genetic algorithms, or other **optimization algorithms**. If the **training error** $\mathcal{L}(D;\theta^*)$ is small, then we say that the training data has been learned well. If $\mathcal{L}(D';\theta^*)$ is close to $\mathcal{L}(D;\theta^*)$ then we say that the learned function has **generalized** well, without **overfitting** the training data. The **power** of a function class can be characterized by the range of functions that can be obtained by varying the parameters, e.g., the VC-dimension describes when all N-dimensional Boolean functions can be implemented and has strong implications with respect to generalization. All else being equal, weaker function classes generalize better given the same data.

Viewing the SHAM experiments through this lens, we can easily establish a correspondence. The **parameters** θ specify the pixel-to-tile map. The input x will be 900-pixel gray-scale images. The **function class** $f(x;\theta)$ returns the output vector $y = (h, a, m)$ of percentages of the respective types of self-assembled shapes after experimental annealing with initial tile concentrations specified by x and the pixel-to-tile map θ. This description takes the function class to be defined by the experimental reality, but of course theoretical analysis would have to adopt a mathematical model thereof, such as the Stochastic Greedy Model developed in Section 2.2 or the simpler abstract models described below. Our **training data** is just the 18 specified images together with their target classes, e.g., for x^α being "Hopfield", $y^\alpha = (1, 0, 0)$. Since our function class is not analytically specified but rather defined by a physical process, for **training optimization** we work with a surrogate cost function $\mathrm{Score}(x)$ based on our model of nucleation kinetics; this surrogate function tallies the estimated log-difference in nucleation rates between the target shape and its best competitor. Although not directly optimizing the mean squared error of shape percentages, a low $\mathrm{Score}(x^\alpha)$ would imply (in the model, at least) that the target shape nucleates much faster than the other shapes, and thus the output shape percentage will approach being 100% the target shape. Other differences are that we minimized the maximum (i.e. worst) image score rather than the mean, which was sensible given that our training set only had 18 images and we wanted all of our experiments to work, and that the optimization used the simplified (but much faster) Window Nucleation Model for the first stage of optimization. The **optimization algorithm** itself was a discrete stochastic hill-climbing algorithm rather than a continuous gradient descent algorithm (such as back-propagation) due the non-differentiable nature of the pixel-to-tile map θ. Finally, it would be unreasonable to claim that our 18 test images are additional iid samples from the same source distribution as the training samples, as the training images were hand-chosen for interest and the testing images were concocted to test robustness to certain kinds of noise and variation. (The exception is the "Harom" training image of a 3, which was taken from the same MNIST database of digit images as the six testing images of 3s.) Despite these differences, we can calculate the mean squared error of the analog classification probabilities from our experimental results, with the training set error $\mathcal{L}(D;\theta^*) = 0.042$ and the test set error $\mathcal{L}(D';\theta^*) = 0.202$ based on the AFM count fractions reported in Figure 5g (substituting $(\frac{1}{3}, \frac{1}{3}, \frac{1}{3})$ for the 7 test images with no AFM counts).

These parallels suggest several questions for further study. While we used the analog probabilities of nucleating the H, A, and M shapes to approximate a discrete classification task with 3 categories, could multifarious self-assembly be trained to approximate an arbitrary real-valued function $g : \mathcal{R}^{900} \to \mathcal{R}^3$ whose outputs represent probabilities? What are the limitations to the range of functions that can approximated by nucleation kinetics, and are there variations on the molecular system design that help or hinder the ability approximate complex multidimensional functions – analogous to the differences in representational capacity of single-layer vs multi-layer neural networks, with linear vs locally-nonlinear vs globally-nonlinear activation functions? What would be analogous to hidden units? Does pattern recognition by self-assembly nucleation benefit from **generalization** and suffer from **overfitting** analogously to neural networks and other machine learning models? Can one train not just the pixel-to-tile map, but train the tile set itself to improve pattern recognition performance? Can the pixel-to-tile map, and/or the tile set, be generalized to be parameterized with continuous variables, so as to allow differentiable gradient descent optimization algorithms?

Notable reference points for neural network classification and function approximation models are linear threshold units (LTU), polynomial threshold functions (PTF), winner-take-all units (WTA), their soft-transition analogs, and multilayer networks thereof. For dimension n analog real input vectors x, an LTU computes a single binary scalar output based on weight vector w and bias b:

$$f^{LTU}(x; w, b) = \varphi(w \cdot x + b > 0) \ ,$$

a PTF (also known as a sigma-pi unit) specifies a polynomial by a set of terms using variables V (which may be the empty set for a bias) and their coefficients w:

$$f^{PTF}(x; w, V) = \varphi\left(\sum_k w_k \prod_{i \in V_k} x_i > 0\right) \ ,$$

and a weighted WTA is typically formulated as having a vector output calculated using a weight matrix W and bias vector B:

$$f_i^{WTA}(x; W, B) = \varphi(W_i \cdot x + B_i > W_j \cdot x + B_j \quad \forall j \neq i)$$

where φ is the 0/1 indicator function for a Boolean truth value. (Using ± 1 to indicate Boolean input and output values, and changing φ accordingly, is also common and can affect the complexity of function computation under resource constraints,[59–61] such as network depth or degree or number of polynomial terms. Remarkably, using

{1,2} for False and True, which may correspond better to low and high concentrations, gives polynomial threshold functions even more computational power.[62]) Comparing LTU to PTF and WTA, key results are that a single weighted WTA can compute functions that require networks of LTUs with a single layer of hidden units[63] and that a single PTF is similarly strictly more powerful than a single LTU but less powerful than a network of LTUs with a single layer of hidden units;[59] the increased computational power of single PTFs with monomial degree bounded by d or with the number of monomials bounded by m have also been studied.[64]

An abstract model of pattern recognition by tile self-assembly nucleation can be formulated based on the Window Nucleation Model that is defined in Section 2.5. Ignoring the heuristic weights used to bias the WNM scores to favor experimentally preferred regions, the nucleation rate estimate (from equation 2.25) for $k \times k$ windows can be written as

$$\eta^{WNM}_{\text{shape}} = \gamma' e^{-G^{WNM}_{\text{ce}}} = \gamma \sum_{\substack{A \text{ is } k\times k \\ \text{in shape}}} \prod_{i \in A} x_i$$

for a given shape, where $x_i = c_i/c$ gives the concentration of tile i normalized to the base concentration c, and γ and γ' lump physical constants. If we define recognition to occur if the initial nucleation rate (considering the tile concentrations to be unchanging) exceeds a threshold, then the classification function

$$f^{WNM}(x; \text{shape}, \text{rate}) = \varphi\left(\sum_{A \text{ is } k\times k} \prod_{i \in A} x_i > \text{rate} \right)$$

is formally a PTF with polynomial degree k^2. It is not presently clear, however, how to characterize the power of this subset of PTF functions, where the set of monomials derives from placement of tiles in the shape, and all weights are 1. That said, perhaps a more interesting abstract model would take into account the fact that tile concentrations deplete, leading to a WTA effect. Using a simplified idealization of this effect, we can abstract the classification function for a multifarious set of shapes as

$$f^{WNM:WTA}(x; \text{shapes}) = \varphi\left(\eta^{WNM}_{\text{shape}_i} > \eta^{WNM}_{\text{shape}_j} \quad \forall j \neq i \right)$$

which might be expected to combine the power of PTF and WTA, but again is restricted by the shape layout constraints.

As an informal empirical assessment of the power of pattern recognition by the nucleation kinetics of multifarious self-assembly, we trained the $f^{WNM:WTA}(x; \text{shapes})$ model to classify the MNIST database of handwritten digits.[65] The 28×28 pixel grayscale images were used as concentration patterns with the white background being interpreted as the base concentration c and the darker digit pixels linearly scaled up to $10c$. The tile layout within 10 shapes, one for each digit class, was optimized by stochastic hill-climbing (tile position swaps and mutations) to maximize the fraction of training set images that were correctly classified. The standard training set of $60,000$ images was used, and after training the performance on a test set of $10,000$ images was assessed. The WNM window size was $k = 3$. Three types of constraints on the tile layouts were considered. (1) Each shape is a 28×28 square containing exactly one copy of each tile. This corresponds to the assumption that arbitrary promiscuous interactions can be designed, as in the original work on multifarious self-assembly.[37] A training error of 7.4% and test error of 10.8% was achieved. (2) Each shape is a 10×10 square containing whatever tiles work best, including possibly multiple copies of the same tile within the same shape. Again, implementation would require arbitrary promiscuous interactions to be designable. A training error of 7.7% and test error of 9.8% was achieved. (3) Each shape is a 12×12 square in which half the tiles, arranged as a checkerboard, are shared among all 10 shapes, while the other half are unique to the given shape. This "simple checkerboard" design could be implemented with SST DNA tiles and Watson-Crick domain interactions. A training error of 12.8% and test error of 13.9% was achieved. Presumably, slightly worse performance could be expected if further restrictions for "guarded edges" were imposed. Furthermore, unlike the shapes designed for the experimental system, no check was performed to ensure that the proofreading property is respected so malformed assemblies such as chimera are largely prevented; enforcing this property could in principle reduce performance somewhat as well.

These results are not interesting in and of themselves – there is no need to solve image recognition problems at the molecular level – but they can serve as an initial calibration of the computational power of nucleation in molecular self-assembly. Is it more like a linear classifier? Or worse? Or better, perhaps giving similar performance as a two-layer neural network? Or even a multilayer deep neural network? Those familiar with the MNIST problem already know the answer: In LeCun's 1998 study,[65] a single-layer neural network with output WTA for classification achieved a 12.0% test set error, a two-layer neural network with 300 hidden units achieved a 4.7% test set error,

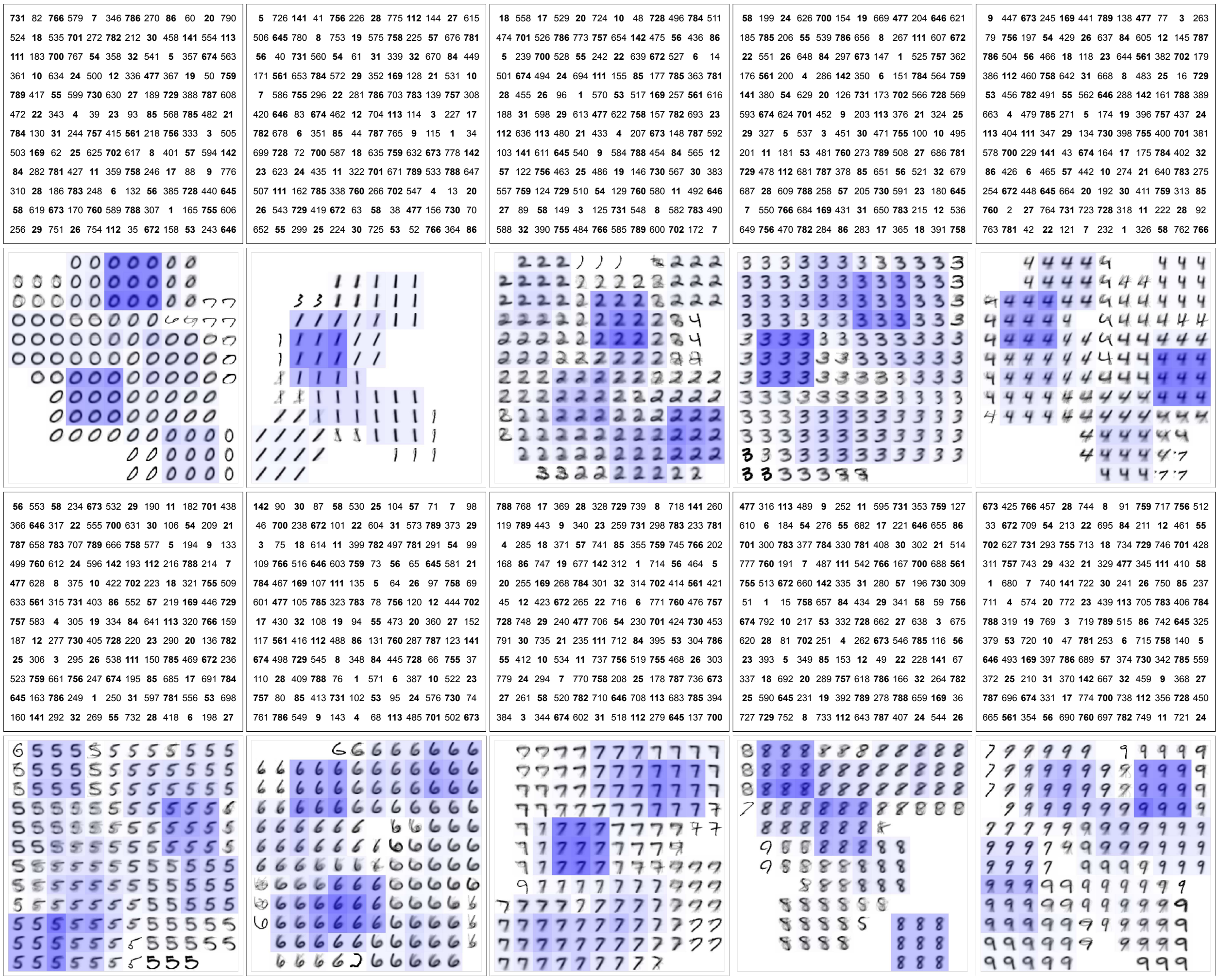

Figure S1.5: Tile layouts for the 12 × 12 shapes classifying each digit (boldface indicates shared tiles) and nucleation maps showing the average image when a given tile is part of the winning nucleation window (blue shading is proportional to the frequency of winning). Empty locations indicate tiles that were never part of a winning nucleation window. This simple checkerboard design has a 13.9% error on the MNIST test set according to the WNM:WTA model with window size $k = 3$. Tiles are numbered consecutively with the pixels in MNIST images, listed as vectors.

and a multilayer convolutional neural network achieved a 0.8% test set error. This comparison places the readily implementable checkerboard design as roughly comparable with a single-layer WTA, but even though it's a reach goal, less-constrained multifarious layouts still perform worse than two-layer neural networks. Thus one might imagine that when intracellular decision-making requires high-dimensional information-processing comparable to a single-layer WTA, multicomponent nucleation might be a suitable mechanism for accomplishing the task.

Training on the MNIST database also reveals a further connection to place cell and Boltzmann machine architectures: training sculpts the energy barrier landscape to be structured analogously to a continuous attractor. To maximize performance, each part of a layout must take responsibility for nucleation in response to different subsets of class images, and overlapping parts of the layout respond to overlapping subsets of image pixels, encouraging neighboring parts of the layout to respond to similar images. This is illustrated in Figure S1.5 for the checkerboard design, which shows the average of images "claimed" by each tile (in the sense that the tile is a part of the fastest-nucleating $k \times k$ window within the fastest-nucleating shape when the image's tile concentrations are used).

One might question whether the simplifications inherent in the above abstractions are appropriate. For example, for the winner-take-all effect during annealing, one might be more concerned with comparing which shape experiences significant nucleation *first*, at the highest temperature, rather than which shape experiences the most nucleation at a given fixed temperature. In this case, the choice of window size k coarsely reflects the choice of annealing speed. Beyond that, as is discussed in Section 2.5, the Window Nucleation Model is only a coarse approximation of nucleation rates for multifarious self-assembly, and as is shown in Section 2.3, the dynamics of tile

concentration depletion only imperfectly results in a winner-take-all effect – thus highly abstracted mathematical models can only serve as a coarse guide for understanding the computational power of real molecular systems, and the question is whether their study can lead to insights and decisions that prove useful when working with the real thing. This bottom line, unsurprisingly, is also very familiar in neural computation, where the LTU, PTF, and WTA models (and special cases thereof, such as the clusteron variant of sigma-pi units) naturally arise as abstractions of single-cell and network computational capability, despite the real thing being incredibly more complex.[66–68]

1.5 The reservoir computing perspective

Our experimental system was first designed to robustly demonstrate multifarious self-assembly of the three shapes H, A, and M, and only later, after that was successfully demonstrated, did we choose a set of images and the system to recognize and classify the images based on nucleation rates with winner-take-all competition. It may at first seem implausible that having first designed a multifarious system with three shapes, agnostic of any specific pattern recognition problem, it just so happened that this set of molecules, with no modifications, could be re-interpreted (via the pixel-to-tile map) as correctly classifying our later choice of 18 training images. Further, we claim it could have performed equally well for most any other set of 18 training images (with similar pixel intensity histograms). Shouldn't the actual physical system – the molecules we use for computing – have at least something to do with with the problem being solved? But in fact, our approach and claims are neither implausible nor unreasonable – they are actually consistent with the essential findings of **reservoir computing**.[69]

Our first perspective will frame the conundrum in the context of DNA computing and molecular programming. One might be more comfortable if there were no pixel-to-tile map, and the task were simply stated as "given the following training set of images using pixels 1 through N, design corresponding DNA tiles 1 through N that will correctly classify each image, when using the pixel intensity to set the corresponding tile concentration." This task allows the designer complete freedom in sequence choice, so that an entirely new set of molecules can be synthesized and used for each new pattern recognition problem. Despite not designing new molecules, our results do solve this task as posed, but additionally we implicitly show that the full design freedom is often not necessary: we restrict not only to using SST but also to using the exact sequences from the SHAM design, just renumbering the tiles.

As a second perspective, note that it is standard in the field for work that focuses on molecular information processing to allow computational signals to be encoded however the system designer wishes, rather than requiring the system to interface with pre-determined input and output molecules. This is analogous to the situation in robotics where sensors and actuators may have electronic components whose construction is specialized to interact with the physical world (photodiodes, electromagnetic motors) but within the programmable microprocessor, all wires and logic gates process information (0s and 1s, low and high voltages) using a **representation that is independent of the physical meaning**. In molecular systems, we may also have processes that translate various chemical signals into a form compatible with the information processing core, as well as those that translate the output of information processing to actuate some chemical response. For example, the DNA strand displacement circuits may use "translator" molecules to convert the presence or absence of a free biological RNA strand into the the presence or absence of a free DNA strand with unrelated sequence that is suitable for circuit computation.[70,71] Thus, work on circuit computation principles often ignores the translation step, and assumes that input signals come in whatever molecular format is needed for the computing subsystem.

From this perspective, we could imagine an input sensor layer upstream of the actual multifarious self-assembly that performs pattern recognition: a set of N translator molecules converts each target input (a DNA or RNA strand, protein, small molecule, ...) to a comparable amount of the corresponding SST strand, which will nucleate and grow into one shape or another once the concentrations of key components become large enough (Figure S1.6). It's clear that the computation for recognizing patterns is not taking place within the translator layer, as this layer just performs a 1-to-1 conversion of molecular sequence. Thus, in our work, training the pixel-to-tile map θ corresponds directly to designing a (virtual) translator layer upstream of the self-assembly process. On the output side, relative positions of neighboring tiles are unique to each shape, and thus could directly support cooperative binding of scaffolding to activate e.g. an enzyme cascade[72] or trigger other shape-selective downstream processes.

A third perspective supposes that we want to classify a set of N target DNA strands, with specific sequences, according to their concentration patterns – and we don't want a separate input translation layer. Our work here suggests that the following approach should be sufficient: Lay out the target strands within three shapes (if we have 3 categories of output) by assigning each strand to a random 'white' checkerboard of positions, then for each shape design an additional $\sim N$ shape-specific tiles to fill the 'black' checkerboard positions. The sequences of the 'black' strands will be dictated by the sequences of the target 'white' strands. For pattern recognition experiments, keep the shape-specific tile concentrations constant at the minimum standard concentration, and add the target

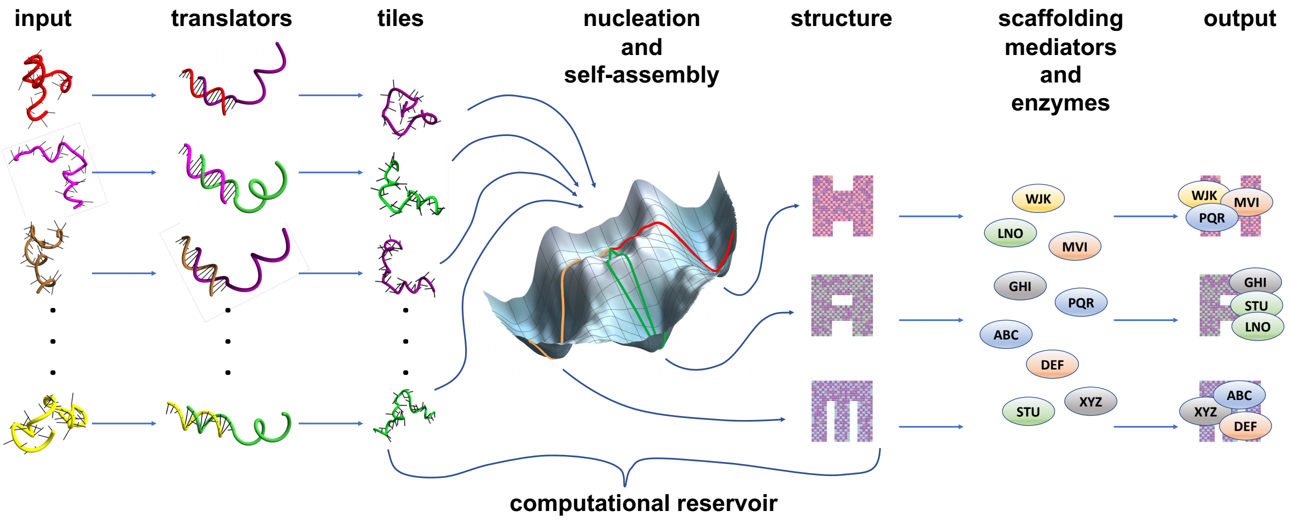

Figure S1.6: Conceptual illustration of multifarious self-assembly as reservoir computing. An input layer of translator gates detects analytes (here nucleic acid strands, but they could be proteins, small organic chemicals, or other molecules that can be recognized by aptamers, for example) by triggering the release of a sequestered tile strand. Design of the translator gate determines which analyte produces which tile strand, analogous to the pixel-to-tile map θ, and thus selects what pattern recognition task the nucleation-and-self-assembly-based computational reservoir will perform. In the presence of an output layer of proteins that cooperatively bind to pairs of tiles that are specific to each shape, the assembly of a specific structure results in activation of a specific enzyme cascade. Other modalities of output could of course be envisioned as well.

DNA according to the pattern to be classified. Computation done this way is maximally ‘entangled’; it does not have a separate sensor layer and a separate compute layer, and thus it would be more compact.

Now let’s return to why one might not be too surprised that using the same SHAM molecules, but just re-assigning the pixel-to-tile map, is likely to be successful for many different pattern recognition training sets. As we’ve said, we consider the phenomenon to be analogous to – or an example of – reservoir computing.

Reservoir computing was initially developed as “echo state networks”[73] and “liquid state machines”,[74] and later generalized. The common motivation was to find an easier and faster way to train recurrent neural networks to predict or classify time-series data, compared to standard approaches such as backpropagation-through-time, which can be very slow because of the difficulty of adjusting network weights that control its dynamical behavior. So instead of modifying the network’s dynamical behavior during training, reservoir computing takes a **fixed recurrent network** that has fixed dynamical behavior, and only trains a linear input layer and a linear output layer – in fact, the standard set-up only trains the output layer, as a simple and fast learning algorithm suffices when there is a direct error signal.

Remarkably, this often works. Perhaps more remarkably, the fixed recurrent network may often be generated randomly, so long as the generation method ensures with high probability that certain properties will hold, such as having damped non-chaotic dynamics, having modes with a broad range of time scales, and having sufficient diversity of nonlinearities that guarantee better approximations for larger networks. Once chosen, this fixed network is referred to as a “reservoir” for dynamical behavior that can be stimulated by the input layer mapping and read out by the output layer mapping. Furthermore, the reservoir needn’t be a neural network, but could be other forms of dynamical systems or even physical systems – indeed, an early example of a reservoir was waves in a pond stimulated by pebbles dropped into it. The fundamental observation is that there exist classes of randomly-generated networks that contain within them the potential for a remarkable diversity of behaviors, which can be accessed easily via training simple (e.g. linear) output and/or input encodings. Although typically used for spatiotemporal pattern recognition, reservoir computing can also be applied to static pattern recognition problems, where the fundamental observation still holds (although seldom being competitive with standard backprop).

In the molecular programming field, reservoir computing has previously been proposed as an architecture for temporal pattern recognition by programmable coupled biochemical oscillators.[75]

To view the SHAM self-assembly system through the lens of reservoir computing, we ask, “What is the task? What is the reservoir? What is the input layer? What is the output layer?” Our task is static pattern recognition, so we don’t need complex temporal dynamics in the reservoir. The reservoir itself is the fixed design of the three-shape multifarious tile set, together with the standard experimental protocol of a constant temperature hold

or a temperature anneal. The dynamics stabilize and yield an output that is the distribution of self-assembled shapes; the output encoding is trivial: shape H means class "H", shape A means class "A", and shape M means class "M". The reservoir is trained to solve different problems by training the input encoding, i.e. the pixel-to-tile map. Unlike the standard reservoir computing set-up, where only the output layer is trained and a simple and fast learning algorithm suffices, the dependence of the output on the input encoding is complicated in the SHAM system, so a brute-force hill-climbing optimization was needed in order to train the system for a particular pattern recognition task. Thus, the characteristic ease of training reservoir systems does not carry over to the SHAM system. However, the fact that a **single fixed core system** contains within itself the potential to solve nearly arbitrary pattern recognition problems (within the system's capacity) is very much in line with the findings and principles of reservoir computing. Furthermore, it helps explain the at-first paradoxical-seeming fact that we do not need to design new molecules in order to use the SHAM system to solve new pattern recognition tasks.

1.6 A compact, robust, and scalable molecular architecture

We consider the multifarious self-assembly architecture explored in this paper to be a remarkably compact, robust, and scalable molecular architecture for solving pattern recognition and classification problems. However, this claim is difficult to quantify, and solid conclusions will have to wait for future investigations. Nonetheless, comparisons to several other experimentally-demonstrated molecular implementations of neural network computation may serve as useful reference points.

As for our work, the architecture is **compact** in the sense that processing 900-variable input vectors required only 900 distinct DNA strands, which are themselves the input strands; it is **robust** in the sense that the strands could be used without purification and in that speckle errors did not hinder recognition; it is **scalable** in the sense that processing N input variables would entail just N strands, and prior demonstrations of uniquely-addressed SST assemblies (in 3D) utilised as many as $N = 30{,}000$ SST tiles.[76] That said, as the computational power of this architecture is not yet characterized, and the tile assembly geometry imposes limitations, we cannot at this time assess how general the pattern recognition would be for $N = 30{,}000$.

The first reference point is the earliest attempt to synthesize a biochemical Hopfield network using DNA 'genelet' templates and 2 essential enzymes.[15,16] Here, a 2-neuron bistable memory required 6 DNA strands; it established a dynamic steady-state that could correct small errors. As each genelet template directly implemented a neural synapse, generalizing to a fully connected N-unit Hopfield memory would require $3N^2$ strands. However, imperfect enzyme activity leads to an accumulation of 'waste' strands that limit function. Modern improvements on the genelet architecture has allowed scaling up to $\sim$ 15-genelet systems.[17]

A second reference point are enzyme-free DNA strand displacement implementations of neural network computation. A use-once implementation of a fully-connected 4-neuron Hopfield memory[21] required 112 strands, which were prepared and purified as 72 separate complexes to avoid performance-limiting leak reactions. Scaling to N-bit patterns would entail $O(N^2)$ purified complexes if all $O(N^2)$ weights are used, but presumably many relevant pattern recognition tasks would not need a fully-connected network. A use-once implementation of a 100-bit input, 6-neuron winner-take-all computation[22] used 225 purified species to classify downscaled and binarized 10×10 MNIST digits using just 20% of available weights. Using the same architecture for a 3-neuron WTA that classifies 900-bit input, analogous to our 900-variable analog pattern classification task, would require two strands per network weight; if just 20% of all possible weights were required to obtain adequate performance, then roughly 1200 strands would be needed, in addition to the 900 input strands. As an alternate comparison, Section 1.4 makes the case that the full-scale MNIST images (28×28 grayscale) could be classified by a multifarious system of ten 12×12 tile shapes, using just 792 DNA strands – again suggesting more bang for the buck. Most recently, a convolutional neural network was implemented with DNA strand displacement circuits,[77] using 512 strands to classify 144-bit input into 32 categories. An impediment to drawing conclusions from these numbers is the hard-to-compare quality of each computation, such as the accuracy performance of the actual experimental system, or its performance on out-of-sample inputs – for example, the convolutional network exhibited robustness to rotated patterns.

As a third reference point, due to the difficulties of protein design and genetic engineering and the intracellular environment, synthetic neuromorphic computation in living cells has not yet advanced beyond three inputs and two neurons.[24–27]

A major consideration for system robustness is the effect of imperfect molecules. Most large strand displacement circuits and enzyme circuits, including the ones mentioned above, require PAGE or HPLC purified strands and complexes in order to reduce failures due to leak and side reactions. Remarkably, our system based on multifarious self-assembly worked using unpurified synthetic DNA strands for the SST, similar to the prior demonstration of algorithmic self-assembly using unpurified SST.[78] There, the robustness was partially attributed to the ability of

crystal growth to exclude impurities under only slightly supersaturated conditions (i.e. only slightly below the melting temperature) – a phenomenon thought to be enhanced by the proofreading tile set design. Since DNA oligonucleotide synthesis errors are dominated by truncations that would cause affected tiles to have weaker binding to other tiles, it is possible that critical nuclei are systematically enriched for non-erroneous strands, conferring an extra degree of robustness in the nucleation energy barrier heights and thus in the pattern recognition kinetics. Furthermore, although not studied here, it is reasonable to expect that theoretical connections between multifarious nucleation and neural networks confers some additional degree of robustness to imperfect inputs, as this is often found in distributed neural computation.

With respect to speed, the DNA strand displacement circuit for classifying MNIST digits operated in 10-hour experiments, which is faster than our 150-hour experiments for multifarious nucleation. However, neither of these represent the limits for the molecular computing. Subsequent work on DNA strand displacement circuits identified new principles, new molecular architectures, and new experimental protocols that reduced the computation time for comparably-sized circuits to just a few minutes.[71,79] The speed limits for computation by nucleation remain to be studied in detail; while general arguments (such as those outlined in Extended Data Fig. E10) suggest an exponential slowdown for problems that require larger critical nucleus sizes, it is not yet clear exactly how much extra computational power the larger critical nucleus sizes would provide – nor do we yet understand the trade-offs with other factors such as concentration ranges, temperature, and the potential for active regulation of nucleation such as is seen in microtubules.[80]

These distinctions in compactness, robustness and scalability between prior work on DNA neural networks and ours are partly explained by our not designing molecules explicitly to carry out each steo of the weighted sum and thresholding mechanisms for individual neurons. Rather, we exploit what molecules do naturally and note the implicit effect on the assembly energy, thereby exploiting their collective behavior to carry out a pattern recognition task.

Put another way, our molecular pattern recognition architecture is founded on uncovering the latent capabilities of *inevitable* molecular processes. *Any* system that self-assembles must go through a nucleation phase, so the question is what features does the process's energy landscape contain and how can they be programmed? In this case the self-assembly energy landscape and nucleation energy barriers share similarities with Hopfield networks, Boltzmann machines, and place cell models, and they can be programmed by geometric layout of tiles within target shapes. It is natural to assume that energy landscapes and energy barriers for other "inevitable" physical processes will also be seen to have inherent computational capabilities in the multicomponent limit where "more is different".

Prior work on programmable self-assembly of DNA tiles primarily focused on a different inevitable process in the multicomponent limit: layer by layer crystalline growth, rather than crystalline nucleation. In that work, nucleation is dictated by an explicit seed, such as a long strand or DNA origami structure to which futher tiles can bind.[78,81–84] There, the seemingly natural connection was to one-dimensional (usually deterministic) cellular automata, whose space-time history pattern is constructed as the algorithmic crystal grows by selecting the best-fitting tile at each growth site. While one-dimensional cellular automata are a powerful model of computation, and they can directly simulate Turing machines and even a class of parallel multihead Turing machines, programming them has a discrete symbolic flavor as it is usually done, which can come across as brittle and belabored. For example, it is currently unclear how to program a cellular automaton to perform the same pattern recognition task as was done here (classifying 18 arbitrary images into three arbitrary classes).

Section 2

Nucleation models and pattern recognition training

We have demonstrated high dimensional pattern recognition by exploiting competitive nucleation in a system of molecules with multiple crystal structures i.e., crystal polymorphism, first described by Mitscherlich[85] and a bane to crystallographers such as Hodgkin.[86] Indeed, many scientific and industrial applications rely on biasing nucleation towards one of the crystal polymorphs by tuning annealing protocols. Our work updates these classic ideas for heterogeneous crystals where the number of distinct components is of the size of the crystal itself.[37,49,87] This new heterogeneous context introduces novel elements such as pattern recognition and winner-take-all nucleation: in our system, depletion of components can lower nucleation rates of off-target structures more so than for the desired structure while in classical crystal polymorphism, depletion lowers nucleation rates for all crystalline forms.

Selection of crystal polymorph formed during nucleation and growth is inherently governed by kinetic, rather than thermodynamic, principles. In the limit of an infinitely slow anneal, of course a finite-sized system will eventually adopt the energetically most favorable polymorph, but the basins in the energy landscape corresponding to the polymorphs may be so deep and with such high barriers between them that this limit is of little or no relevance to experimental systems. For experimentally-relevant time scales, the kinetics determining which basin becomes occupied most quickly.

In the SHAM system, there are four natural kinetic regimes to consider. First, on the fastest time scale where most nucleation occurs at a low enough temperature that the critical nuclei are just dimers of two tiles, the amount of nucleation will be roughly proportional to the number of critical nucleation pathways, i.e. the number of distinct dimers that could form. In this case, structure A is slightly favored, as it has an area of 496 tiles compared to 480 for the other two shapes (c.f. Figure 2b). Second, a slower anneal will allow significant nucleation before the temperature gets so low, so critical nuclei will be large enough that their energy barrier will depend on the integrated contributions of perhaps dozens of tiles, and thus on the spatial colocalization of high-concentration tiles. Once nucleated, continued growth will trap the assembly within a deep energy basin, regardless of which structure has formed. This is the regime explored in detail in our work here. Third, an anneal may so slow that the relative melting temperatures of each structure determines which is observed: During the temperature interval between the highest-melting-temperature structure and the next highest, only one structure can form, and given enough time, it will, to the exclusion of the others. As shown e.g. in Section S5.3.38, the model we discuss below predicts that structure H has the highest melting temperature. Finally, the fourth regime is an anneal slow enough to reach thermodynamic equilibrium, in which case the structure H will almost completely dominate, as it has the highest area-to-perimeter ratio (c.f. Figure 3e). Although in this case the third and fourth regimes select for the same structure, it need not in general be the case, because the DNA hybridization and entropic effects can give different energy-vs-temperature slopes for different structures. These conclusions still generally hold, with some adjustments and within certain limits, when the tile concentrations are not uniform.

Similar kinetic selection of non-equilibrium trapped states has been seen more broadly in polymer folding, e.g., in synthetic DNA origami,[88] natural co-transcriptional folding of RNA,[89] and annealing of DNA hairpin systems.[90,91] Further, such non-equilibrium self-assembly could be made contingent on a complex chemical context, defined by combinations of molecules rather than single species – illustrating how such systems could process information and make decisions.

The following section develops rudimentary mathematical models and simulation algorithms for quantitatively treating these issues in the context of multifarious self-assembly of DNA tiles.

Models of nucleation and growth in self-assembling systems must strike a balance between a closeness to physical reality that allows them to capture details of complex behaviors, and an abstraction that allows broader theoretical reasoning. To investigate different questions about our system, we used four different models of nucleation at different points in this spectrum.

More detailed and physically realistic than any of the models we used would be atomic-level[92] and coarse-grained[93,94] molecular dynamics simulations of the DNA molecules themselves. Although we do not consider any such models explicitly here, we make use of conclusions derived from such studies by others.[95]

The lowest-level model we use directly is the single-assembly kinetic Tile Assembly Model (kTAM),[96] for which a stochastic simulator, Xgrow, is available (Section 2.1). This model abstracts the details of DNA tiles – whose implementations involve strands that interact through hybridization and folding processes – into abstract tiles that attach and detach in single steps and form perfectly rigid lattices. We use the kTAM to analyze the growth of assemblies in our system, and the potential for the formation of chimeric assemblies. To bring the simulation predictions into the ballpark of experimental reality, we attempted to tune the model parameters to fit the single-stranded tile (SST) motif, using experimental and computational results on DNA tile energetics.[95,97,98]

While simulations in the single-assembly kTAM are useful for favorable growth processes, where assemblies are likely to grow through similar pathways into similar structures and thus the simulation of one, or a few, individual assemblies can be instructive, nucleation involves the exploration of many unfavorable pathways by many small assemblies at once, many of which will melt, and so direct simulation of nucleation by the single-assembly kTAM would be computationally expensive. However, the model was generalized by Schulman & Winfree to consider multiple assemblies growing according to mass-action kinetics; they used this to prove theoretical bounds on nucleation rates that can be calculated with some knowledge of the energy landscape of a system's possible assemblies and assembly pathways.[99] To sample assembly pathways for this bound without needing to exhaustively enumerate them, which would be computationally intractable, we developed the Stochastic Greedy Model, which seeks to find likely assembly pathways for assemblies in our system through greedily making favorable attachments and stochastically choosing unfavorable ones. The assembly pathways thus enumerated are then evaluated with respect to the reactions in the generalized kTAM, allowing estimation of pattern-dependent differences in nucleation rates. We used the Stochastic Greedy Model (Section 2.2) for predicting pattern-dependent nucleation rates in our experiments (Figures 3, 4, and 5) as well as for final optimization of pixel-to-tile mapping for pattern recognition.

However, as a stochastic model requiring the collection of many trajectories to produce low-variance estimates of nucleation rates, the Stochastic Greedy Model was either too slow (with more trajectories) or too noisy (with fewer trajectories) to provide the fast, stable comparisons of nucleation rates between similar concentration patterns needed for optimization algorithms searching within the large space of pixel to tile mappings. For this purpose, we instead developed a simple, deterministic, heuristic model, the Window Nucleation Model (Section 2.5), based on intuitive reasoning about the thermodynamics of small assemblies and potential critical nuclei along assembly pathways. This model does not provide a nucleation rate, but instead provides a score value for each concentration pattern such that, if one pattern has a higher score than another, then that first pattern is likely, though not guaranteed, to have a higher nucleation rate in the Stochastic Greedy Model than the second. Using only simple functions on arrays, the calculations for this model were used for the optimization of pattern-to-tile mappings in our pattern recognition experiments, starting from random assignments; once optimization with the Window Nucleation Model was ended, further optimization was performed with the Stochastic Greedy Model, to fine-tune the pixel-to-tile map and to verify nucleation rates under that more physically-meaningful model.

The Stochastic Greedy Model and Window Nucleation Model, as well as kTAM simulations in Xgrow, consider monomer tile concentrations as being constant over time and not being depleted by assembly, as though controlled by a chemostat. In reality, monomer concentrations deplete as structures nucleate and grow, potentially changing nucleation and growth rates and leading to a winner-take-all effect seen in experiments. We build a simplified model of such a winner-take-all phenomena using a simplified 1-dimensional Markov chain model with representing nucleation and growth, modeled with time-dependent transition rates (Section 2.3).

2.1 The kinetic Tile Assembly Model and Xgrow

The kinetic Tile Assembly Model (kTAM) abstracts DNA tiles as oriented squares with labeled sides.[96,100] It assumes topologically correct self-assembly, so an assembled object is a connected arrangement of tiles on the square lattice. Tiles are "connected" if they have abutting sides, in which case we may speak of the "bond" between those tiles, whose strength may depend on the pair of labels indicating bond type.

Thermodynamics is specified by associating an energy to each possible assembly based on the tiles involved and how well their side labels match; kinetics is specified by associating rates for each possible tile addition or

removal, satisfying detailed balance. Association between two multi-tile assemblies will not be considered in the basic kTAM model, nor will splitting of an assembly into two multi-tile assemblies. (Such interactions, along with lattice defects, can be important for DNA tile assembly,[82,83] but we did not encounter strong evidence for them in this work.) Thus, considered as a chemical reaction network (CRN) with infinitely many possible species, the reactions are of the form:

$$A + t_i \underset{k_r}{\overset{k_f}{\rightleftharpoons}} A'$$

where A and A' are assemblies, t_i is a tile of type i, and A' is the same as A but for the addition of tile t at some location. The base model only considers monomer addition reactions, which must be reversible in order to satisfy physical detailed balance, and thus single tile detachments that would break the assembly are not considered. Except when explicitly specified otherwise, kTAM models do not incorporate any notion of globally correct tile placement; they allow any tile to bind in any location so long as at least one bond matches. Thus, tile attachments and assemblies that contain errors with respect to the target complete structure will be considered.

The simplest model for the thermodynamic energy of an assembly considers just the summed interaction strength of all side-to-side bonds based on whether the labels match, and assumes identical bonding strength for matching labels with non-matching labels contributing nothing:

$$G_{bonds}(A) = B \ G_{\text{se}}$$

where B is the total number of matching bonds and G_{se} is the generic bond strength for a suitable choice of units. With respect to these units, detailed balance is expressed, for equilibrium concentrations, as

$$\frac{[A][t_i]}{[A']} = \frac{k_r}{k_f} = \hat{u}_0 \ e^{-b \ G_{\text{se}}}$$

where b is the number of new matching bonds in A' that were not already in A, and $\hat{u}_0$ is the reference concentration for these units. We will see later that $\hat{u}_0$ must be chosen carefully, and will most likely not be standard units. Note that $G_{\text{se}} \geq 0$ and more positive values correspond to stronger bonds.

Our simplifying assumption for kinetics is that k_f does not depend on assembly size or tile type. Thus it can be considered as a parameter, and k_r can be calculated for a given tile dissociation from a given assembly based on the detailed balance equation.

Further, it is often simpler and sufficiently insightful to consider assemblies growing in a bath of tiles whose concentrations are held constant. (This scenario is relevant, for example, during the time before significant nucleation has depleted the monomer concentrations.) Here, we will need to allow different tile types to have different concentrations, and we use the notation

$$[t_i] \overset{def}{=} c_i = \hat{u}_0 \ e^{-G^i_{\text{mc}}} \tag{2.1}$$

where G^i_{mc} is the parameter controlling the concentration of tile type t_i, with larger values corresponding to lower concentrations (and thus greater positional entropy per molecule). With tile monomer concentrations held constant, telescoping detailed balance immediately gives us the equilibrium concentration for each assembly:

$$[A]_{eq} = \hat{u}_0 \ e^{G_{bonds}(A)} \prod_{i \in A} \frac{c_i}{\hat{u}_0} = \hat{u}_0 \ e^{B \ G_{\text{se}} - \sum_{i \in A} G^i_{\text{mc}}} \overset{def}{=} \hat{u}_0 \ e^{-\hat{G}(A)} \tag{2.2}$$

where the assembly energy

$$\hat{G}(A) = \sum_{i \in A} G^i_{\text{mc}} - B \ G_{\text{se}} \tag{2.3}$$

is a chemical potential with respect to the tile monomer concentrations.

Because our model does not incorporate interactions between assemblies, each assembly's behavior is independent from the others in the powered scenario, so a single assembly's growth can be modeled as a continuous-time Markov chain (CTMC) where each state is a possible assembly, transitions correspond to the addition or removal of a single tile t_i that is connected by b bonds, and transition rates are given by:

$$r_f = k_f c_i = k_f \ \hat{u}_0 \ e^{-G^i_{mc}} \tag{2.4}$$

$$r_{r,b} = k_r = k_f \ \hat{u}_0 \ e^{-b \ G_{\text{se}}} \tag{2.5}$$

With every reaction being reversible, this CTMC also satisfies detailed balance, and if it reaches equilibrium (which is only possible for parameters below the melting temperature) then the probability of observing assembly A is:

$$p_{eq}(A) = \frac{1}{Z} e^{-\hat{G}(A)} \qquad \text{where} \quad Z = \sum_A e^{-\hat{G}(A)} \ . \tag{2.6}$$

Note that if a tile is removed from an assembly of size 2, then either tile can be considered the 'remaining' single-tile assembly. As mentioned above, tile removals that would result in multiple, disconnected assemblies cannot be allowed if detailed balance is to be respected.

This simple model can be efficiently simulated by the stochastic Gillespie algorithm[101,102] using a k-d tree to store rates for log-time steps, as done in Xgrow.[103] The Xgrow simulator has options to allow a limited variety of reverse reactions that break the assembly into multiple pieces, at the cost of also breaking detailed balance. Specifically, the 'chunk fission' option can be roughly described as: (a) if after a single tile is removed, the remaining assembly is not connected, an irreversible reaction that removes this tile and keeps only the largest remaining assembly is used, with a rate $k_{r,b}$ set by the bonds broken when removing just that tile, and (b) multi-tile subassemblies up to size 2×2 are considered for irreversible removal with rate $k_{r,b}$ where now b is the number of bonds along the perimeter of the subassembly. Without this option, unrealistic surface roughening occurs near the melting temperature due to the inability of small clusters to fall off when they are well-connected internally but have only 1 bond with the main assembly. Although Xgrow does not implement arbitrary fragmentation of large assemblies, during growth of chimeric multifarious structures wherein partial assemblies of two distinct shapes are connected in just one place, circumstances can arise where removal of a single subassembly of size 2×2 or smaller will result in separation of the two sides. This is observed in Extended Data Figure E2.

Xgrow has been used extensively in the literature[82,104,105] as it provides a simplifying clarity, but quantitative comparison with experimental studies of DNA tile systems requires further adjustments, as discussed below.[100]

2.1.1 Estimates of kTAM parameters for SSTs and double-crossover tiles

There are now extensive experimental demonstrations of tile-based DNA self-assembly of linear (tubes and ribbons[106–108]), two-dimensional (finite and unbounded[109–111]), and three-dimensional (finite and unbounded[112–114]) structures using double-crossover tiles,[115] single-stranded tiles[108] as well as other DNA tile motifs. Each has its own features that make the kTAM and its variants more or less accurate.

Suppose we have experimentally measured free energies and rate constants for assembly, given for natural units of concentration, time, and energy. We will show that if a few plausible conditions hold, exactly or approximately, then we can find corresponding parameters for the simple kTAM model – thus allowing us use parameters extracted from the literature as a baseline for our simulations of nucleation kinetics.

Specifically, let us assume (1) that the rate constant for tile attachments is identical for all locations and all tile types, so that always $r_f = k_f c_i$ as before; and (2) that the standard free energy of attachment depends only on the the number of matching bonds formed, b, and is linear. Therefore, we can extract the offset and slope, and write:

$$\Delta G_b^\circ = (\alpha - b\ G_{\text{se}})\ R\ T$$

where α and G_{se} are unitless, R is the gas constant, T is the temperature in Kelvin, and the energies use standard units with respect to a standard concentration such as $u_0 = 1$ M.

Considering the per-second rates of tile attachment and detachment at a specific site in an assembly, the simple basic model and the experimentally parameterized model should agree exactly, so

$$r_f = k_f c_i \qquad = k_f\ \hat{u}_0\ e^{-G^i_{mc}} \qquad = k_f\ u_0\ e^{-G^i_{\text{mc}}+\alpha} \tag{2.7}$$

$$r_{r,b} = k_f\ u_0\ e^{\Delta G_b^\circ/RT} \qquad = k_f\ \hat{u}_0\ e^{-b\ G_{\text{se}}} \qquad = k_f\ u_0\ e^{-b\ G_{\text{se}}+\alpha} \tag{2.8}$$

where $\alpha = \ln \frac{\hat{u}_0}{u_0}$ and thus the choice of units for the simple model are determined by the offset α via $\hat{u}_0 = u_0\ e^{\alpha}$.

Unfortunately (though not surprisingly) experimental and computational studies[95,97,98] report that the assumptions we used are only approximately correct for DNA tiles:

1. For DNA double-crossover tiles assembling into lattices on mica, as observed by atomic force microscopy movies,[97] ΔG_b° was linear in b (within error bars) over a 100-fold range of off-rates, while on-rates were constant within a factor of 2 (except where fewer than 10 data points were collected, e.g., for $b = 4$).

2. For DNA double-crossover tiles assembling in solution, as measured by fluorescence,[98] a roughly linear dependence on b was also found for ΔG°_b, now covering a 10^9-fold range of rates, with on-rates remaining constant within a factor of about 4. Importantly, this work also introduced additional loop energy terms that the basic kTAM cannot accommodate, but fortunately these only come into play for "non-convex" assemblies that are rare during nucleation and growth near the melting temperature. In particular, tiles attaching or detaching by 4 bonds involve rare non-convex assemblies, and we are less concerned about inaccuracies of energies and rates for such reactions.

3. For DNA single-stranded tiles assembling in solution, examined computationally by coarse-grained molecular dynamics of 32 distinct local contexts,[95] and averaging over contexts with the same b, again we see a roughly linear dependence on b for ΔG°_b covering again a roughly 10^9-fold range of rates. Although they don't calculate rates, they too assume a constant for the on-rate. Again, their value for $b = 4$ is anomalous (by $\sim 2\ R\ T$), and again we are content to ignore it.

To find reasonable parameters for the kTAM to model our SST system, we will make use of the computational work[95] because the underlying oxDNA model[116] has been well calibrated, and detailed experimental measurements for SST are not yet available. From pages 7-18 of the supplementary materials, the rightmost value of F/kT gives the value of $\Delta G^\circ_b/RT - \ln([t_i]/u_0) \approx 9 - 6\ b$ for $b \in \{1, 2, 3\}$. Since $\Delta G^\circ_b = (\alpha - b\ G_{\rm se})RT$ and in the computational work $[t_i] = 100$ nM and $u_0 = 1$ M, we take $G_{\rm se} = 6$ and $\alpha = -7.1$, and use $k_f = 10^6\ /M/s$. Given α and $[t_i]$, $G^i_{\rm mc}$ is determined, completing the parameterization needed for simulation. (Note that recent work[78] with SSTs did not model an α-like effect, resulting in an estimated $G_{\rm se} \approx 8$ for 100 nM tiles that may be too high.)

Note, however, that these parameters are for $T = 50$ °C, as that was the temperature used in the simulations. For our purposes, we are interested in nucleation and growth rates at different temperatures, in particular the critical range between 53 °C and 45 °C. Therefore, we must consider the temperature-dependence of parameters k_f, α, $G_{\rm se}$, and $G^i_{\rm mc}$. A roughly 50% increase in the association rate constant was observed[98] for DNA double-crossover molecules between 12 °C and 21 °C, but we will be satisfied holding k_f constant. We also make the simplifying assumption that α is temperature independent, i.e. that it represents purely entropic factors. Consequently, $G^i_{\rm mc}$ will also be temperature independent.

To provide a temperature dependence for $G_{\rm se}$, we note that association $b = 1$ corresponds to uncomplicated hybridization of two single-stranded species, for which the thermodynamics is well-understood.[117] In particular,

$$G^\circ_b = (\alpha - G_{\rm se})RT = \Delta H^\circ - T\Delta S^\circ \quad \text{for } b = 1 \tag{2.9}$$

$$G_{\rm se} = \Delta S^\circ/R - \Delta H^\circ/RT + \alpha \tag{2.10}$$

$$T = \Delta H^\circ/(\Delta S^\circ - R(G_{\rm se} - \alpha)) \tag{2.11}$$

We used an implementation of nearest-neighbor energy calculations with SantaLucia 2004 parameters[117,118] to calculate ΔH° and ΔS° averaged over all binding domain sequences in the SHAM system (excluding the poly-T domains used on shape edges): $\Delta H^\circ = -72.76$ kcal/mol and $\Delta S^\circ = -0.2013$ kcal/(mol· K). For $\alpha = -7.1$, this is effectively linear over the range of interest, with

$$T(G_{\rm se}) \approx 49.74 - 2.85(G_{\rm se} - 5)\ ^\circ\text{C}\ . \tag{2.12}$$

For reference, $T = 50$ °C corresponds to $G_{\rm se} = 4.91$, $T = 48$ °C corresponds to $G_{\rm se} = 5.61$, and $T = 46$ °C corresponds to $G_{\rm se} = 6.33$. Concentrations of 16.6 nM, 50 nM and 60 nM correspond to $G_{\rm mc}$ values of 10.81, 9.71 and 9.53, respectively, for pattern recognition, flag, and control experiments.

The above discussion makes it clear that the parameterization of the nucleation model is fairly rough, and we do not expect quantitative agreement with experimental results. Our aim for the model is to provide quantitative insight into the nucleation phenomena with this highly heterogenous system, to observe qualitative effects that may also be present in the real system, and to provide a baseline against which to assess the effectiveness of our sequence design and pixel-to-tile mapping.

With respect to these standard units, we define the free energy (chemical potential)

$$G(A) = \hat{G}(A) - \alpha = -B\ G_{\rm se} + \sum_{i \in A} G^i_{\rm mc} - \alpha \tag{2.13}$$

where as before B is the total number of matching bonds between tiles in the assembly. Now, at equilibrium, with monomer concentrations held constant at their initial values,

$$[A]_{eq} = u_0\ e^{-G(A)} = u_0\ e^{B\ G_{\rm se} - \sum_{i \in A} G^i_{\rm mc} + \alpha}\ . \tag{2.14}$$

In the following, we will sometimes find it convenient to refer to a *base concentration* $c = \hat{u}_0\ e^{-G_{\mathrm{mc}}} = u_0\ e^{-G_{\mathrm{mc}}+\alpha}$ such that individual tiles have concentrations $[t_i] = c_i = cf_i = \hat{u}_0\ e^{-G^i_{\mathrm{mc}}} = u_0\ e^{-G^i_{\mathrm{mc}}+\alpha}$.

2.2 Stochastic Greedy Model of nucleation

The equilibrium concentration of a particular assembly given by Equation 2.14 does not generally hold while the system is out of equilibrium. In particular, as in the experiments considered here, in an isolated system that initially only contains monomers and does not replenish them as they are incorporated into structures, the total tile concentrations will remain constant, and thus free monomer concentrations will deplete. When there is a significant barrier to nucleation, the subset of pre-nucleation structures may quickly approach equilibrium with respect to each other, contributing to some tile depletion, while further growth of nucleated structures continues the depletion on a longer time scale.

Let us first review classical nucleation theory of homogeneous crystals before delving into the more complex heterogeneous case.[48,119] Using the above formalism for homogeneous crystals, there is is a single tile type and thus a single concentration, c. To estimate the nucleation rate we find the size (in number of tiles) of the critical nuclei, as their concentration limits nucleation. For a given number of tiles, K, the highest concentration assembly A_K will have a shape as close as possible to being square, as that maximizes the number of bonds. (This can be interpreted as minimizing perimeter for a given area, which generalizes as surface area versus volume.) For simplicity, we will just treat $K = n^2$ for integer n and then interpolate results as if K were a continuous variable. In this case,

$$[A_K]_{eq} = u_0\ e^{-G(A_K)} = u_0\ e^{2n(n-1)\ G_{\mathrm{se}} - n^2 G_{\mathrm{mc}} + \alpha}$$

since the number of bonds in an $n \times n$ square is $B = 2n(n-1)$. For continuous n, we find the critical nucleus size by minimizing $[A_K]$ with respect to n: $\frac{d[A_K]}{dn} = 0$ when $n = \frac{G_{\mathrm{se}}}{2G_{\mathrm{se}} - G_{\mathrm{mc}}}$. As the crystal melting temperature, in this notation, occurs when $G_{\mathrm{mc}} = 2G_{\mathrm{se}}$, we see that the critical nucleus size becomes arbitrarily large as the system approaches arbitrarily close to the melting temperature. More concretely, a little algebra yields $[A_K] \sim e^{-nG_{\mathrm{se}}} = e^{-\sqrt{K} G_{\mathrm{se}}}$. The nucleation rate η measures the production of larger-than-critical assemblies; ignoring smaller factors, such as the role of non-square critical assemblies and the multiplicities of possible nucleation pathways, we can approximate

$$\eta \approx k_f c [A_K] \sim e^{-\sqrt{K} G_{\mathrm{se}}}$$

as the order-of-magnitude rate of nucleation, in molar per second. Correspondingly, the time scale for nucleation, τ, will be inversely proportional to the critical nucleus concentration, so $\tau \sim e^{\sqrt{K} G_{\mathrm{se}}}$. This already suggests an insight relevant to pattern recognition by multifarious self-assembly: to make use of large critical nuclei that integrate more information, we can change reaction conditions to increase K, for example by decreasing the base concentration (increasing G_{mc}) while keeping the temperature constant (fixed G_{se}), but this comes with a corresponding exponential slow-down in the computing speed. The result is essentially the same if we hold the concentration (via G_{mc}) constant and adjust the temperature (via G_{se}) to obtain larger K. That said, it can't be taken for granted that insights derived from classical nucleation theory of homogeneous crystals will apply unchanged to the heterogeneous multicomponent, as has been highlighted by significant theoretical and experimental work that extends this framework.[50,51,87,99,107,120–123]

For multifarious self-assembly of single-stranded tiles, we are interested in the overall rate of nucleation η_{shape} for each possible final assembly shape. With multiple tile types, each having a different concentration and specific binding interactions, there may be many distinct nucleation pathways involving many types of critical nuclei with particular shapes and compositions, and the situation becomes far more complex than for homogeneous systems. Nonetheless, Schulman & Winfree [99] show that the equilibrium concentration for an assembly, with respect to the initial monomer tile concentrations, is an upper bound for the concentration of that assembly at any time in the time evolution of a mass-action model with monomer concentrations that deplete during growth, if the initial state consists of monomers and assemblies grow by reversible monomer addition only. As a result, the total rate at which larger assemblies form from a particular assembly A, which we will call its growth flux, has an upper bound of

$$\eta^+_{\{A\}} = k_f\ [A]_{eq} \sum_{j \in J(A)} c_j = k_f\ u_0\ e^{-G(A)} \sum_{j \in J(A)} c_j \tag{2.15}$$

where $J(A)$ is the set of possible tile attachments to A and we use that for all times t, $[A](t) < [A]_{eq} = u_0\ e^{-G(A)}$.

From this per-assembly growth flux, we can derive an upper bound for nucleation, or more generally, a limiting rate for growth from monomers to a set of particular target assemblies. Consider any set of assemblies S in the space of all possible assemblies, such that every series of tile attachment and detachment events going from an individual tile monomer to the set of target assemblies must contain at least one of the assemblies in S as an intermediate assembly. We might want to consider S to be a 'boundary surface' that separates pre-nucleated structures from post-nucleated structures. For example, for a target assembly of size N, the set of all assemblies with size exactly K (for some $K < N$) would satisfy this property. However, in general S can be any set of assemblies that must be crossed during assembly, and this gives us considerable flexibility when finding suitable S. For any such S, considering all series of attachments and detachments, the total flux through the 'surface' S, and thus from monomers to target assemblies, has an upper bound of

$$\eta^+ = \eta^+_S = \sum_{A \in S} \eta^+_{\{A\}} \tag{2.16}$$

This result is considered more rigorously by Schulman & Winfree [99]. It relies only on S containing a complete surface that all growth must pass through. A poor choice of surface (or a set with extraneous members) will simply result in an unnecessarily high upper bound: for example, in conditions where $G(A)$ does not begin to decrease until assembly sizes much larger than dimers, the set of all dimers D would still result in η^+_D constraining the total rate of target production, but the bound would be very high. Generally, a surface that all growth must pass through where $G(A)$ is high for each $A \in S$ will tend to result in the smallest η^+_S, and thus the tightest bound on nucleation. It would be desirable to find an optimal surface, i.e. one that strictly minimizes η^+_S.

Even in cases where there are few tile types, regular periodic order, and homogeneous concentrations, enumeration of a complete minimal surface may be infeasible, but excellent approximations can be articulated by taking advantage of symmetry and considering just the most dominant terms. For example, in such cases S may be all assemblies of a well-chosen critical size K, for which the dominant species may be easy to describe. Specifically, as assemblies with higher $G(A)$ within a set S will contribute exponentially less to η^+_S, it often suffices to consider the assemblies within the set with the *smallest* $G(A)$ values, which will contribute the most to η^+_S. For homogeneous systems, these will simply be those that have the most bonds with the fewest tiles, often corresponding geometrically to having the largest area for the smallest perimeter.

In the case of unequal concentrations and inhomogenous designs, however, symmetry breaks. Each possible assembly must be considered separately, as each tile may have a different concentration, resulting in a different $G(A)$ despite geometric similarity, and the incorporation of more high concentration tiles at the expense of increasing perimeter may result in $G(A)$ being smaller, breaking the link between geometric compactness and free energy. As a result, simple approaches to finding the largest contributors to a particular η^+_S, for example, considering squares of increasing size in a homogeneous, single-tile-type system,[100] cannot be easily adapted to a non-homogeneous system with many tile types.

More generally, consider the set of all possible trajectories from a single tile to a full assembly by monomer addition, and collect – without duplication – the highest-energy point along each trajectory, which we may call the 'critical nucleus' for that trajectory. (Using 'critical nucleus' for these assemblies is a bit of an abuse of terminology, but please bear with us.) The set S of all such critical nuclei contains a complete separating surface (along with much else) and its lowest-energy point must be reached or exceeded by any trajectory. Pruning this conceptual S down to a smaller subset that contains just enough of the lower-energy assemblies could provide a tractable approximation for the nucleation rate.

Thus, we developed the *Stochastic Greedy Model* to stochastically generate a set S of 'critical nuclei' for the nucleation of a single shape, essentially considering the tile system as a uniquely-addressed tile system assembling that one shape. Only correct tile attachments on the uniquely-addressed lattice are considered: attachments of incorrect tiles by one bond are ignored, as are any attachments of tiles that aren't present in the shape, and each tile i will only appear at a particular site (x, y) on the lattice. The algorithm functions by taking some series of probabilistic trajectories using a 'two step' process for each change in assembly: taking an unfavorable step probabilistically, and then deterministically taking the resulting series of possible favorable attachments, while recording the free energy of the assembly immediately after the unfavorable step to try to find a critical nucleus.

Given a set S thus enumerated by the Stochastic Greedy Model, the nucleation rate estimated by Equation 2.16 may be above or below the actual nucleation rate for monomer addition assembly in the bulk mass-action kTAM. To the extent that there are gaps in S such that some kTAM assembly trajectories do not go through S, the estimate will be too low. Both having too few sampled trajectories, and considering only greedy trajectories, contribute to this effect. To the extent that some kTAM assembly trajectories go through multiple states in S, the estimate will be too high. Both enumerating a "thick" surface due to stochastic variability, and the fact that kTAM

trajectories may reversibly cross a perfect surface multiple times, contribute to this effect. Finally, to the extent that assembly concentrations for the non-equilibrium kinetics remain lower than the equilibrium concentrations given fixed monomer concentrations, the estimate will be too high. Both tile depletion during growth, and the inclusion of "critical nuclei" whose concentrations are drained by nearly-irreversible post-critical growth, contribute to this effect. Accepting these limitations, we characterize the estimated effective nucleation rate, given the enumerated set of critical nuclei S, in terms of an effective ensemble barrier energy G_{ce} such that

$$\eta_S^+ = k_f \; u_0^2 \; e^{-G_{\text{ce}}} \qquad \text{where} \qquad G_{\text{ce}} = -\ln \sum_{A \in S} \sum_{j \in J(A)} \frac{c_j}{u_0} \; e^{-G(A)} \; . \tag{2.17}$$

We can interpret G_{ce} as the macrostate free energy for a set of microstates that consist of the identified critical nuclei together with an arriving tile that has lost its translational entropy by colocalizing at a prospective site of attachment, but which has not yet formed bonds.

Since our multifarious self-assembly design has the property that a pair of tiles that are adjacent (in a given orientation) in one shape are guaranteed to be non-adjacent (in that orientation) in the other shapes, any multi-tile critical nucleus can be expected to grow specifically into the complete shape that it is consistent with. Therefore, when S was enumerated based on a specific shape, the nucleation rate estimate for that shape can be written

$$\eta_{shape}^+ = \eta_S^+ \; .$$

While our algorithm is sufficient for the concentration patterns used in this work, more subtle concentration patterns that are nearly equally localized in multiple structures will require solving the issues raised here. One approach to addressing many of these issues is to adapt a transition path sampling method,[124] like forward flux sampling[125] as a wrapper around the kinetic Tile Assembly Model described above. These statistical sampling methods are explicitly designed to determine the rate of processes controlled by rare events and have been applied to nucleation,[126] albeit not in the multicomponent limit with unequal concentrations needed here.

We now provide details of the Stochastic Greedy Model.

2.2.1 Trajectories

For each target shape (H, A, M), we generated 40,000 trajectories of possible assembly paths from a single tile all the way to a complete shape. Each trajectory starts from a single tile, used as the initial seed for growth, that is chosen probabilistically by concentration, such that the probability of choosing tile i is $P^i_{\text{start}} = c_i / \left(\sum_i c_i\right)$. The trajectory will exclusively involve tile additions, such that assembly A_0 is just tile t_i and after s tile addition steps, assembly A_s consists of $(s+1)$ tiles in total and has free energy (chemical potential) $G(A_s)$ as defined above.

At each point in trajectory construction, where A_s is the current assembly, the algorithm calculates $\Delta G_{\text{att}}(x,y) = G(A'_s) - G(A_s)$ for every point (x,y), where A'_s reflects the potential addition of the correct tile at position (x,y). We further calculate a Boltzmann weight $p_{\text{att}}(x,y) = e^{-\Delta G_{\text{att}}(x,y)}$, which we use for the probability that the algorithm will select this site for the next tile attachment. For speed, this calculation is done by keeping values in two arrays, and only changing values adjacent to the change in the assembly array: i.e., for every tile attachment, these two values are recalculated for whichever of the four locations adjacent to that tile attachment are empty. For a given site (x,y) where tile i can attach and form b bonds, we use $\Delta G_{\text{att}}(x,y) = G_{\text{mc}} - b \; G_{\text{se}} - \ln f_i$ if $b > 0$, while $\Delta G_{\text{att}}(x,y) = \infty$ and thus $p_{\text{att}}(x,y) = 0$ if $b = 0$.

For the first (presumed unfavorable) step, and subsequently whenever the assembly is again in a state where only $\Delta G_{\text{att}} > 0$ attachments are possible, the algorithm chooses a step to take with a probability of attachment $P(x,y) = p_{\text{att}}(x,y) / \sum_{X,Y} p_{\text{att}}(X,Y)$. Note that this is not the actual probability of that tile attachment taking place in the kTAM, which would be determined only by tile concentrations. If there is no attachment possible at all, i.e. the assembly is complete, then the trajectory ends.

After selecting (x,y), the algorithm makes the selected attachment (the first of the step's two parts) and updates the ΔG_{att} and p_{att} arrays. The G of the assembly is stored as $G(s)$, where s is the step number. Note that this is stored after the $\Delta G_{\text{att}} > 0$ (unfavorable, and probably $b = 1$) attachment. The maximum value of $G(s)$ is stored as the current best guess for the critical nucleus free energy G_{cn} along the trajectory, and the assembly state that resulted in that maximum is stored until replaced by a higher-$G(s)$ assembly.

After the $\Delta G_{\text{att}} > 0$ attachment, the algorithm runs a filling routine that repeatedly attempts to make an arbitrary $\Delta G_{\text{att}} < 0$ attachment, until no such attachments are possible. Because of the properties of the assembly (all correct tile attachments, only one possible tile per site, no detachment, no negative glues), the order of attachments is not important. This is the second part of the step: making every possible subsequent favorable step after the initial unfavorable step.

As used in this work, the algorithm always continues until the final complete shape is formed and thus no more correct attachments are possible; however, for increased efficiency, it is also defined such that it may stop after a given number of tiles have formed, which the user may believe to be sufficient to guarantee nucleation has occurred.

At the end of a trajectory, the algorithm checks to see whether the final assembly free energy $G(\text{end}) < G(\text{base})$. In other words, it checks whether the final assembly is more favorable than a low-concentration monomer tile, for equilibrium with respect to fixed tile concentrations. If this is not the case, then the algorithm reports the trajectory as a failure. As used in this work, where trajectories are computed all the way to the complete shape, either all trajectories are successful or all trajectories are failures, given a concentration pattern, temperature, and shape. With respect to the fixed tile concentrations, we define the melting temperature for a given pattern and shape as point where $G(\text{complete}) = G(\text{base})$. (Melting temperatures would not be pattern-dependent if all tiles were shared in all shapes, but as it is, different patterns may place more high-concentration tiles in different shapes; the additional shape-dependence comes from the different area-to-perimeter ratios.)

The trajectory algorithm then returns the following information to the nucleation rate algorithm:

- The G_{cn} found by the algorithm: the highest $G(s)$ along the trajectory.
- The critical nucleus assembly that corresponds to that G_{cn}.
- $G(s)$ for every step s.
- The step s where G_{cn} is found.
- Whether $G(\text{end}) < G(\text{base})$.

Example greedy trajectories for H flag 6 are shown in Fig. 3c. Figure S2.1 shows a Boltzmann-weighted sample of critical nuclei in the enumerated sets S for each shape, for the H flag 6 concentrations at $T = 49.2$ °C, at $T = 48.3$ °C, and at $T = 45.5$ °C. Given the quantitative differences between the theoretical model and experimental results, we only look to interpret qualitative features of the critical nucleus sets. It is immediately clear that (a) most critical nuclei in H are near the flag, while critical nuclei in the other shapes are scattered around; and (b) the typical critical nucleus size is strongly dependent on temperature in the samples shown, varying from 3 to 16 for the on-target shape and from 3 to 73 for the off-target shapes, with the greatest difference being in the off-target shapes. Figure S2.2 shows an analogous Boltzmann-weighted sample of critical nuclei for the Avogadro pattern recognition experiment. The phenomena are similar here, although the on-target critical nuclei are somewhat larger on average, off-target critical nuclei are somewhat smaller, and estimated nucleation rates are somewhat closer to each other, consistent with the narrower range of temperatures used. As the critical nuclei for the on-target shape can be quite small, there is a sense in which the selectivity of pattern recognition relies on the rejection of nucleation attempts in the off-target shapes, where the amount of information being considered is governed by the size of their critical nuclei at the relevant temperature.

Fig. 3e shows macrostate ensemble free energy for assemblies of a given size, using the ensemble of trajectories generated by the Stochastic Greedy Model. Let S_K be the set of size-K assemblies that appear in some enumerated trajectory. Then we calculate and plot $G_{SGM}(K) = -\ln \sum_{A \in S_K} e^{-G(A)}$. Because the greedy trajectories are biased toward the low-energy assemblies that dominate this Boltzmann-weighted sum, G_{SGM} is an adequate approximation of the macrostate ensemble free energy for the set of *all* size-K assemblies for the given shape.

2.2.2 Nucleation rate

The Stochastic Greedy Model generates a set number of trajectories. Once these trajectories have been generated, the algorithm has a set of distinct critical nuclei, S, and associated information. Using the enumerated set S, the algorithm estimates the ensemble nucleation barrier energy G_{ce} and the nucleation rate η^+ according to equations 2.15, 2.16, and 2.17, with one adjustment: rather than summing over all possible tile additions from each critical nucleus, $J(A)$, we sum over only the favorable (energetically downhill) tile additions, which we call $J^+(A)$. While the full set $J(A)$ is required for a rigorous upper bound in the general case, for the set of critical nuclei enumerated by the Stochastic Greedy Model, which are guaranteed to have at least one downhill tile addition step available, neglecting the uphill tile additions avoids counting flux that is likely to be immediately reversed, while keeping the

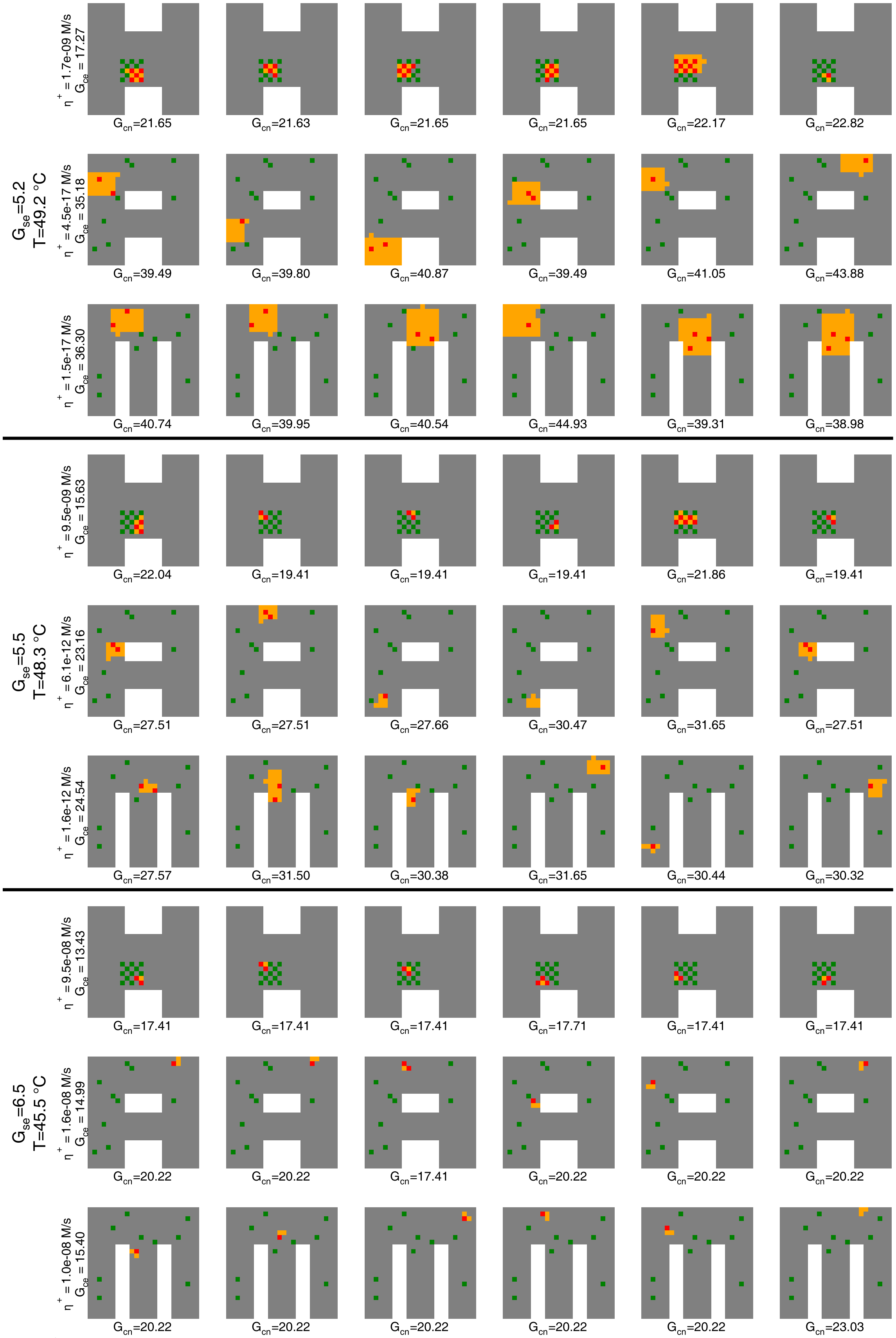

Figure S2.1: A Boltzmann-weighted sample of 6 'critical nuclei' for each shape, at three temperatures, for H flag 6. Orange and red indicate low- and high-concentration tiles that are part of the critical nucleus, respectively; grey and green are used analogously for tiles not in the nucleus. η^+ is the estimated nucleation rate for the shape; G_{ce} is the corresponding critical nucleus ensemble free energy; G_{se} is per-bond differential sticky-end energy at the shown temperature; and G_{cn} is the chemical potential of the given critical nucleus.

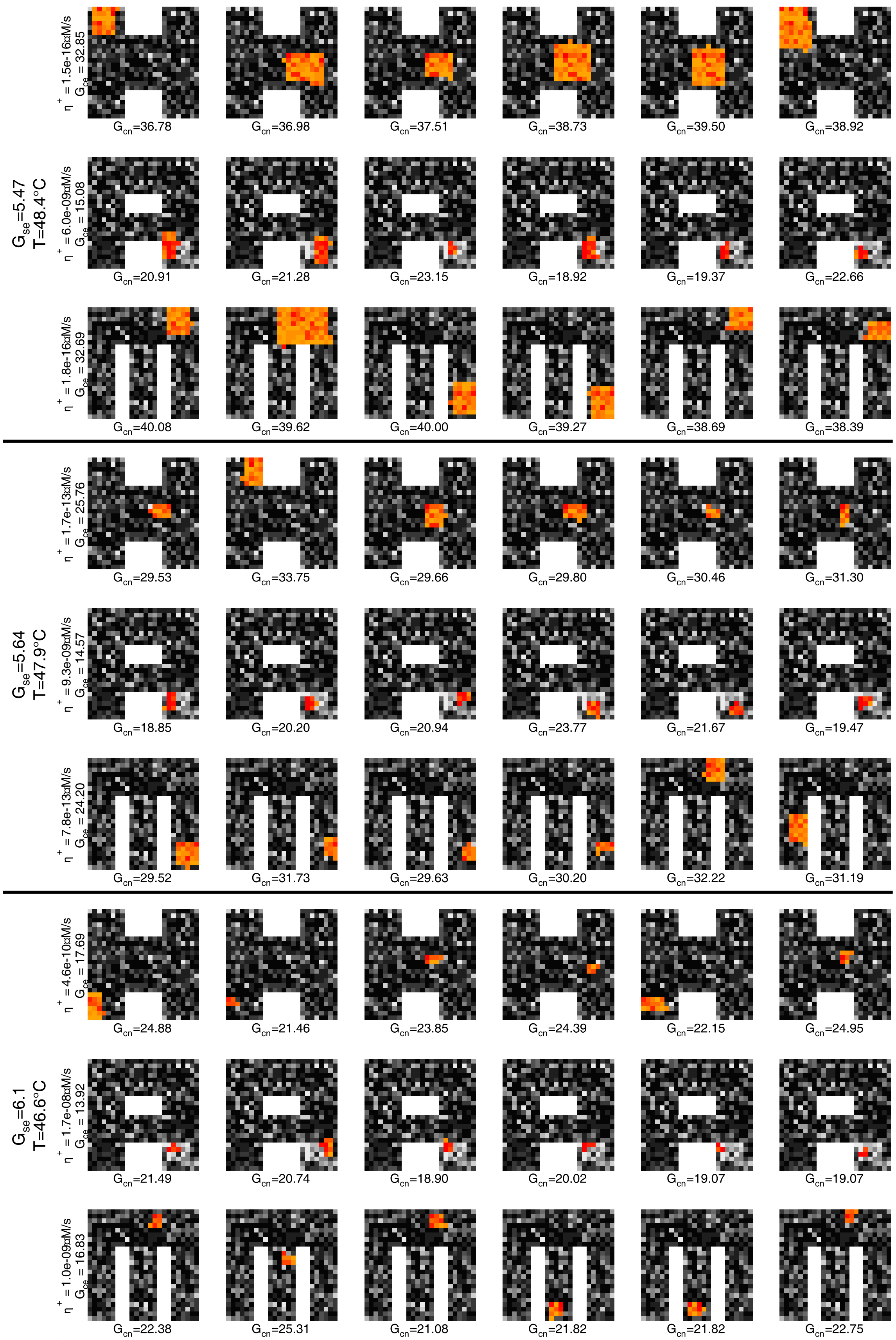

Figure S2.2: A Boltzmann-weighted sample of 6 'critical nuclei' for each shape, at three temperatures, for Avogadro. The color scale from orange to red scales linearly from the lowest to highest concentration, and is used for tiles that are part of the critical nucleus; the color scale from black to white is used analogously for tiles not in the nucleus. η^+, G_{ce}, G_{se}, and G_{cn} are as in Figure S2.1

dominant favorable tile additions. Thus, we *replace* the previous formulas with:

$$\eta^+_{\{A\}} = k_f\ [A]_{eq} \sum_{j\in J^+(A)} c_j = k_f\ u_0\ e^{-G(A)} \sum_{j\in J^+(A)} c_j \tag{2.18}$$

$$\eta^+ = \eta^+_S = \sum_{A\in S} \eta^+_{\{A\}} = k_f\ u_0^2\ e^{-G_{\mathrm{ce}}} \tag{2.19}$$

$$G_{\mathrm{ce}} = -\ln \sum_{A\in S} \sum_{j\in J^+(A)} \frac{c_j}{u_0}\ e^{-G(A)}\ . \tag{2.20}$$

The total nucleation rate for a shape gives no information on the region where nucleation began, even though, with unequal tile concentrations, some regions may be much more likely than others to participate in nucleation rather than to grow by tile attachments after a structure has nucleated. While trajectories in the Stochastic Greedy Model start from individual tiles, the trajectories are used to find distinct critical nuclei, and a particular critical nucleus may arise from trajectories starting from many different positions. An alternative measure of the contribution of a particular location to the nucleation rate of a shape, calculated only from the set of distinct critical nuclei S that was enumerated for the given shape, is the sum over all critical nuclei that include the given location, of the nucleation flux through the nucleus divided by its size:

$$\eta^+_{shape:x,y} = \sum_{A\in S \text{ s.t. } x,y\ \in A} \eta^+_A / N(A) \tag{2.21}$$

where $N(A)$ is the number of tiles in A. By weighting the nucleation rate in this way, the contributions of regions to the total nucleation rate of a shape can be calculated by adding the contributions of each location, and the sum of contributions for all locations in a shape equals the total nucleation rate:

$$\sum_{x,y\ \in shape} \eta^+_{shape:x,y} = \eta^+_{shape}$$

Because we performed a rough calibration of the kTAM parameters in Section 2.1.1, we can use the Stochastic Greedy Model to estimate the temperature dependence of nucleation rates for any given shape and pattern of tile concentrations. Simultaneously, for each temperature we can calculate the Boltzmann-weighted (i.e. weighted proportional to equilibrium concentration) average critical nucleus size. In Sections 5.3 and 6.4, we perform this analysis for each sample from the flag scan and pattern recognition experiments, respectively. These calculations use the initial concentrations of each tile, and do not model the depletion process. Although both nucleation rate and average critical nucleus size can be calculated above the shape- and pattern-dependent melting temperature (where $G(\text{complete}) = G(\text{base})$), their interpretation becomes more fraught, so we mark the melting temperature with a dashed vertical line and plot only at lower temperatures.

It should be emphasized that our nucleation rate estimates are not intended to be quantitatively accurate: the kTAM model and its calibration only roughly capture the thermodynamics and kinetics of our system; the further approximations used in the Stochastic Greedy Model are also rough; and nucleation kinetics are notoriously sensitive with order-of-magnitude differences between theory and experiment being common and often difficult to explain.[48,127] (Example deficiencies in our estimates can be seen in the plots for flag scan experiments, which contain abrupt jumps in the nucleation rate estimate that are artifacts of the Stochastic Greedy Model's enumeration process for 'critical nuclei'.)

In particular, we do not expect the model to accurately predict the temperature (during a ramp experiment) at which nucleation becomes measurable, nor the absolute or relative nucleation rates at a given temperature. For example, in the pattern recognition experiments, the time to go from about 5% to 10% quenched was at least about an hour, indicating that nucleation was never faster than about 5×10^{-13} M/s, and this mostly occurred between 47 °C and 48 °C. The nucleation model, on the other hand, estimated that this rate would occur between 48.4 °C and 48.6 °C for most patterns, and that at this temperature, the on-target shape would nucleate at least 2 (and often more than 5) orders of magnitude faster than the off-target shape − which fails to explain cases such as Mockingbird where the off-target shape nucleates at nearly the same temperature. This suggests that the nucleation rate estimates may be a few orders of magnitude too high; were k_f decreased, the experimentally-observed nucleation rates would correspond to a lower temperature where on-target and off-target rates are more similar in the model.

Nonetheless, our model provides several important insights (perhaps better phrased as hypotheses) that help us appreciate features of nucleation in multifarious self-assembly systems and general characteristics of their ability to

perform pattern recognition. Most notable is the sharp increase in nucleation rate for the on-target shape as the temperature drops below its melting temperature, in contrast to the much more gradual increase for the off-target shapes. This is accompanied by a fast transition (i.e. over a narrow temperature range) from large critical nuclei to small critical nuclei for the on-target shape, whereas the transition occurs much more slowly for the off-target shapes.

Consequently, selectivity for the correct target shape (i.e. the ratio of nucleation rates) increases as one considers increasing temperatures, with the best selectivity near the melting temperature where absolute nucleation rates are low. However, consistent with the shapes having different melting temperatures (M has the lowest melting temperature due it having the highest perimeter-to-area ratio), it is possible that as one gets *too* close to their melting temperatures, the target shapes' nucleation rate may plummet below the off-target shapes (as occurs for all the M flag scan patterns). That said, the relative stability of on-target nucleation rates sufficiently below the melting temperatures provides another perspective on the previously-mentioned interpretation that pattern recognition selectivity comes primarily from rejection of off-target nucleation attempts.

2.3 Simplified models of depletion and winner-take-all effects

The above model of nucleation in multicomponent systems provides a basis for understanding the dependence of nucleation rate on concentration patterns, and thus the ability of self-assembly to perform pattern recognition, under the assumption that the temperature and monomer tile concentrations are fixed. The model considers each shape independently, and thus could be used to ask whether a single uniquely-addressed shape could recognize patterns via a nucleation rate that is above a "YES" threshold or below a "NO" threshold, depending on whether the pattern should be recognized. To classify patterns into multiple groups (such as H, A, and M) one could use three distinct uniquely-addressed shapes, sharing no tiles, if the patterns contain enough redundant information that a YES/NO decision for each shape is possible based on a partitioning of pixels into three groups. We expect this would be possible for the training set of 18 images used in this work, for example, using 3 new shapes each containing 300 tiles.

What then is the advantage of a multifarious self-assembly system with shared tiles, if any? Intuitively, we expect two advantages. First, a system with fully-shared tiles would enable all shapes to be responsive to all pixels, and thus redundancy in the pattern information would not be necessary. Our system, with a partially shared tile set, partially addresses this issue. Second, when one shape nucleates first, it will deplete the concentrations of the shared tiles, and this may limit the nucleation of other shapes, resulting in a winner-take-all effect. Such a winner-take-all effect allows experiments with a temperature ramp that does not require precise knowledge of the nucleation temperatures for on- and off-target structures. In contrast, in systems without shared components, experiments would have to be carried out at a specific temperature that allows specificity; such a temperature may be hard to know *a priori* and will not be universal, varying with the specific patterns and structures in question.

Here, we wish to quantify this winner-take-all (WTA) effect and provide some understanding of under what circumstances it will or will not occur, and how it can aid the pattern-recognition process. These questions could presumably be addressed by building on the kTAM or SGM, adding equations to model the depletion of each tile type during the nucleation and growth of self-assembling structures, but doing so would be prohibitively expensive computationally. Further, it is hoped that simplifying to the essential ingredients provides more insight.

Absence of winner-take-all in homogeneous systems. Before examining how monomer depletion can lead to a winner-take-all effect in multifarious systems, it is worth commenting on why this effect would not be expected in simpler systems. Crystal polymorphism has been well-studied since the time of Mitscherlich; the ability to nucleate only the most stable form of several polymorphs is an industrially relevant problem, in domains ranging from making chocolate to drugs. Consequently, numerous annealing protocols have been developed to enhance the yield of, say, the most stable polymorph while minimizing nucleation and growth of the competing phases.

The winner-take-all effect described here makes the selectivity problem easier in the multicomponent limit and is absent in the well-studied case of homogeneous crystals. In the multicomponent structures studied here, the on-target structure, e.g., H in the models above, can nucleate from a region of high concentration tiles while critical seeds of the off-target structure, say A, predominantly involve low concentration tiles (since the same high concentration tiles are not spatially localized on A). Depletion affects the low concentration tiles much more significantly than the high concentration tiles; hence depletion will more significantly affect the nucleation rate of A (the off-target structure) than the nucleation rate of H (the on-target structure). In contrast, in homogeneous crystal polymorphs, depletion will affect nucleation of both on and off target structures to similar extents because both nucleation rates are determined by the concentration of the same component(s). Hence, we do not expect a winner-take-all effect for crystal polymorphic structures involving only one or a few kinds of components.

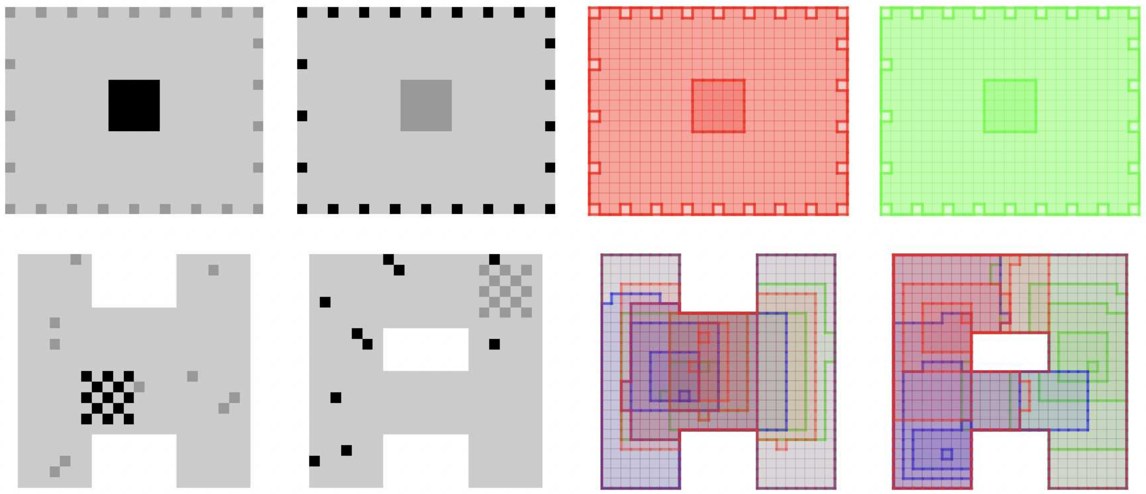

Figure S2.3: Schematic illustration of the CG CRN model (top) and the SGM-lite CRN model (bottom). On the left are the two shapes, called "H" and "A", with black indicating the set of tiles whose concentrations will be enhanced in this study to favor nucleation of H, and intermediate grey indicating another set of tiles that could be used to favor nucleation of A. On the right we indicate the assemblies that are considered in the models: The CG CRN just uses three assemblies for each shape, e.g. H_{nuc}, H_{mid}, and H_{full}. The SGM-lite CRN considers all assemblies that occur in M pre-computed stochastic greedy trajectories for each shape, starting from a single high-concentration tile and proceeding by monomer addition to the full shape. Outlines of sample assemblies along three color-coded trajectories are shown for each shape.

Presence of winner-take-all in multifarious systems with shared components but not in multi-component systems with independent components. We will consider the assembly of two structures, say 'H' and 'A', from a pool of monomers that deplete over time as monomers get incorporated into the structures. The structures may be competing for shared monomers, or may not be competing. Two models will be developed. The first is a highly simplified model that just tries to capture the notion of classical Arrhenius nucleation kinetics combined with uniform growth and maturation using shared components. We call it the "coarse-grained" (CG) CRN model. The second model is still oversimplified, but it adopts more details and an overall framework similar to the SGM, with individual tiles and individual assemblies growing (or shrinking) by reversible monomer addition reactions that satisfy detailed balance. We call it the "SGM-lite" CRN model. They are both illustrated schematically in Figure S2.3.

The coarse-grained model. The CG model has two variants, one considering all tiles being shared between the two shapes, and the other considering all tiles to be distinct. The shared-tiles (S) variant has the following 6 reactions:

$$
\begin{aligned}
c_n^H &\longrightarrow H_{nuc} && \text{with rate} \quad k_{nuc}(T, c_n^H) \\
c_n^A &\longrightarrow A_{nuc} && \text{with rate} \quad k_{nuc}(T, c_n^A) \\
c_g + H_{nuc} &\longrightarrow H_{mid} && \text{with rate} \quad k_{grow}(T, c_g)N/(N-2s) \\
c_g + A_{nuc} &\longrightarrow A_{mid} && \text{with rate} \quad k_{grow}(T, c_g)N/(N-2s) \\
c_n^A + H_{mid} &\longrightarrow H_{full} && \text{with rate} \quad k_{grow}(T, c_n^A)N/s \\
c_n^H + A_{mid} &\longrightarrow A_{full} && \text{with rate} \quad k_{grow}(T, c_n^H)N/s
\end{aligned}
$$

where $N = 500$ is the total number of tiles in each shape, $s = 25$ is the number of high concentration tiles and also the number of tiles in the assemblies H_{nuc} and A_{nuc}, and species c_n^H, c_n^A, and c_g respectively represent the tiles in two nucleation regions and the growth region (black, intermediate grey, and light grey in Figure S2.3). Because our reactions represent coarse-grained pathways involving many individual tile addition steps, in each class the concentrations of all tiles will be the same, and we can model it using a single species concentration. That is, the first pair of reactions represent the nucleation and growth of all s tiles in the nucleation regions; the second pair of reactions represent the growth of the assembly to include all but the tiles that could nucleate the opposing shape; and the third pair of reactions completes the shape. Thus, one copy of each relevant tile is consumed in each coarse-grained reaction step.

The rates for these coarse-grained reactions are calculated from approximations of kTAM kinetics incorporating

classical nucleation theory. Specifically, we assume that in the nucleation region there is a critical nucleus whose size depends on temperature (via the sticky-end binding strength G_{se}) and tile concentration (via the energy parameter $G_{\mathrm{mc}} = \alpha - \ln c/u_0$), and the nucleation rate is proportional to its equilibrium concentration. (As usual, $\alpha = -7.1$ and $u_0 = 1$ M.) That is, we take $k_{nuc} = k_n\ c_{\mathrm{critical}} = k_n\ u_0 e^{-G(A_{\mathrm{critical}})}$ where $G(A_{\mathrm{critical}}) = -2k(k-1)G_{\mathrm{se}} + k^2\, G_{\mathrm{mc}} - \alpha$ for a $k \times k$ square critical nucleus, and $k = G_{\mathrm{se}}/(2\, G_{\mathrm{se}} - G_{\mathrm{mc}})$ is determined by maximizing $G(A_{\mathrm{critical}})$ to find the free energy barrier. We interpolate k to continuous real values, rather than integers, for convenience. We set $k_n = 0.01$ /s to roughly match SGM nucleation rates for the range of concentrations considered. The time for growth of the rest of the nucleation region is assumed to be negligible with respect to nucleation itself. (Note that H_{nuc} and A_{nuc} do not represent the critical nuclei themselves, but rather the entire region that is considered prone to nucleation.) A weakness of this simple model is that it does not consider entropic effects due to there being many places where nucleation could initiate and likewise many alternative critical assemblies that could contribute to the overall nucleation rate.

For growth of assemblies, our reference rate considers the expected time for a biased random walk to complete N steps, with forward steps occurring at rate $k_f\, c$ and reverse steps occurring at rate $k_{r,2}$, where as per the kTAM, $k_{r,b} = k_f\ u_0\ e^{-b\, G_{se} + \alpha}$ and $k_f = 10^6$ /M/s. That is, it is the net rate for completing the growth of the entire shape assuming each reversible step involves forming or breaking 2 bonds. For coarse-grained steps that involve fewer net steps, the rate is accordingly rescaled. Note that barriers for initiating a new layer of tiles on a facet are not considered here, although they can be very significant near the melting temperature when $2\, G_{\mathrm{se}} \approx G_{\mathrm{mc}}$.

Altogether, this gives:

$$\begin{aligned} k_{nuc}(T,c) &= k_n\ u_0 e^{-G_{se}^2/(2\ G_{se} - G_{mc})} && \text{if}\ \ 2\, G_{se} > G_{mc}\ \ \text{else}\ \ 0 \\ k_{grow}(T,c) &= (k_f\ c - k_{r,2})/N && \text{if}\ \ 2\, G_{se} > G_{mc}\ \ \text{else}\ \ 0 \end{aligned}$$

where G_{mc} is a function of c and G_{se} is a function of the temperature T as per Equation 2.12. We will consider an anneal from well above the nucleation temperature for either shape (for our range of concentrations) down to a temperature where growth is strongly favored, specifically, from 55 °C to 30 °C over some time t_{ramp} during which G_{se} increases linearly with time.

The no-shared-tiles (NS) variant has just the following 4 reactions, as there is no need to distinguish two populations of growth tiles:

$$\begin{aligned} c_n^H &\longrightarrow H_{nuc} && \text{with rate}\ \ k_{nuc}(T, c_n^H) \\ c_n^A &\longrightarrow A_{nuc} && \text{with rate}\ \ k_{nuc}(T, c_n^A) \\ c_g^H + H_{nuc} &\longrightarrow H_{full} && \text{with rate}\ \ k_{grow}(T, c_g^H)N/(N-s) \\ c_g^A + A_{nuc} &\longrightarrow A_{full} && \text{with rate}\ \ k_{grow}(T, c_g^A)N/(N-s) \end{aligned}$$

Results from simulations of the CG model are shown on the left in Figure S2.4. For comparison, the flag experiments of Figure 4 cooled from 48 °C to 46 °C over 100 hours, for a rate of 0.02 °C per hour; the slowest simulation in the CG model traverses 25 °C over the same time, i.e., it is 12.5 times faster. We consider this range because the CG model's approximations appear to overestimate reaction rates, making the CG results "too perfect" for slower ramps. Similarly, the fact that the CG model considers high-concentration tiles within a solid region, rather than in a checkerboard pattern, makes the model more sensitive to concentration enhancements, and thus we consider a maximum 6-fold enhancement instead of the 17.6-fold enhancement used in the flag experiments.

The general trends are as expected: For model variant (S) with shared tiles, greater concentration enhancements and/or slower temperature ramps result in both higher selectivity and greater completion. In contrast, while the model variant (NS) with no shared tiles similarly obtains greater completion for greater concentration enhancements and/or slower temperature ramps, it only obtains high selectivity for very fast ramps (for which completion is poor). This is because, without competition for tiles, both shapes will eventually grow to completion in the NS variant of the model; observing the results before it has had time to grow is the only way to obtain selectivity, which results from significant nucleation from colocalized high-concentration tiles occurring at an earlier time in the ramp than nucleation from low-concentration tiles in the off-target shape.

(Note that if we considered a temperature hold instead of a ramp, then we could work at a temperature that is initially very selective for the target shape, if only we knew what temperature that should be. But due to concentration depletion, nucleation and growth would come to a halt before reaching completion.)

Given the simplification inherent in this model, it is worth considering which phenomena are likely to hold up in more accurate models, differing quantitatively but not qualitatively, and which phenomena might change substantially. A few concerns may be considered. The CG behavior for fast ramps may not transfer to the

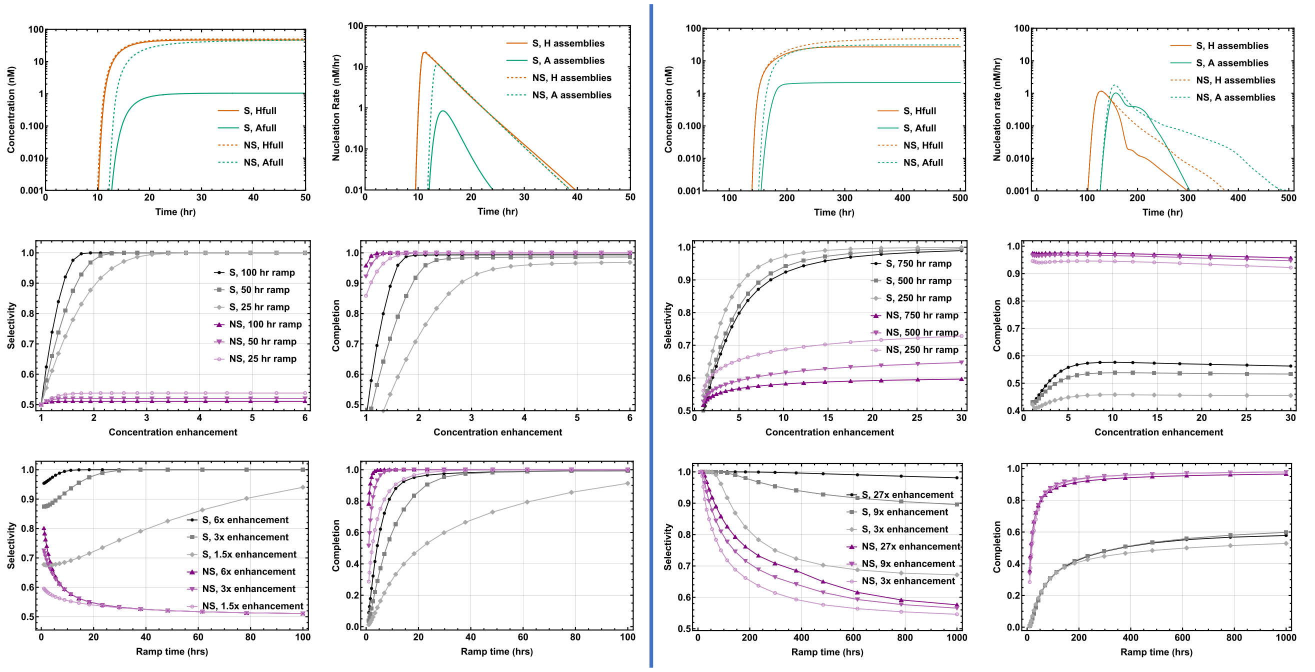

Figure S2.4: Simulation results examining the winner-take-all effect due to depletion during an anneal. The CG model is shown on the left, and the SGM-lite CRN model is shown on the right. All simulations were done with a base concentration of 50 nM. The top plots show concentrations of complete assemblies and nucleation rates as a function of time for an intermediate temperature ramp rate (50 hours and 500 hours respectively) and an intermediate concentration enhancement (3-fold and 9-fold respectively). In the lower plots, selectivity is computed as the fraction of final complete assemblies that are the target shape, H, and completion is computed as the final concentration of the target shape as a fraction of the limiting base concentration. Nucleation is computed as the rate of production of H_{nuc} or A_{nuc} in the CG model, and as the net rate of production of assemblies containing at least 25 tiles in the SGM-lite model.

experimental system because: (1) SST form unstructured aggregates rather than well-defined crystalline assemblies when cooled too fast; (2) classical nucleation theory assumes that the critical nuclei are at equilibrium instantly, whereas our experimental results suggests that nucleation and growth kinetics are not well separated. (Specifically, when a single shape contains multiple labels at different locations, experimental fluorescence trajectories commonly exhibit a temporal order of quenching consistent with the distance between the expected nucleating center and the fluorophore position within the shape, indicating that timescales for creating critical nuclei and for completing the growth process are overlapping.) The CG behavior for slow ramps may not transfer to the experimental system because: (1) near the melting temperature, facet nucleation barriers will limit growth, but these are ignored by the model; (2) nucleation and growth are modeled with irreversible reactions, but as concentrations drop, small assemblies will become unfavorable and melt due to Ostwald ripening. To gauge the severity of these issues, we explore a slightly less oversimplified model.

The more detailed model. The SGM-lite model also has two variants, one considering tiles being shared between the two shapes identically as in the experimental system, and the other considering all tiles to be distinct. All reactions in this CRN are of the form:

$$A + t_i \underset{k_{r,b}}{\overset{k_f}{\rightleftharpoons}} A'$$

where A is an assembly (or single tile), t_i is a single tile, and A' is assembly A with tile t_i added in the correct position with respect to either the on-target or off-target shape, forming $b \geq 1$ bonds. Due to computational constraints, not all such reactions are considered. Rather, for each shape we generate $M = 8$ trajectories starting with a single high-concentration tile, adding a random tile that attaches by at least 2 bonds if that is possible, else adding a random tile that attaches by just 1 bond, until the full shape has been grown. As we use the actual tile arrangement for the experimental H and A shapes, which have respectively 480 and 496 tiles, this leads to roughly 8000 reactions and a comparable number of species. Because nucleation in the model is confined to start with the high-concentration tiles for a particular pattern, in this case H flag 6, the resulting CRN is only suitable for simulating that concentration pattern – but for any level of concentration enhancement and at any temperature. Thus, by letting G_{se} be a function of time, as in the CG model, we may use a standard simulator for mass-action

chemical kinetics [128].

Results from simulations of the SGM-lite model are shown on the right in Figure S2.4. The simulated cooling rate is now ten times slower than for the CG model, 0.025 °C/h compared to the experimental 0.02 °C/h, but over a wider range, again from 55 °C to 30 °C.

Many trends are similar as in the CG model, but there are some noticeable differences. Perhaps most noticeable is that in the shared-tile model variant, the yield of complete on-target shapes never exceeds 60% for our parameters, even under circumstances where the selectivity is near perfect. This is related to another striking difference: that the off-target nucleation rates are delayed from but not suppressed by the preceding on-target nucleation and growth – both peak at about 1 nM/h although the off-target nucleation occurs later, unlike in the CG model where the maximum off-target nucleation rate is more than 10-fold lower than the off-target rate. What we observe happening is that while the on-target shape nucleates and slowly grows to its full size, the off-target shape nucleates but grows to only on average a little less than 100 tiles in size. Sensitivity to slow growth kinetics can also be seen in the surprising reduction of selectivity in the shared-tile model as the ramp gets slower, for low concentration enhancements. Slowing the annealing further by 10-fold only moderately ameliorates this effect.

A fuller picture of the winner-take-all effect in pattern recognition by nucleation in multifarious self-assembly will require further investigation.

2.4 Pattern recognition training

The goal of pattern recognition training is to optimize the pixel-to-tile map θ so as to enhance nucleation of the target shape for each training image, while suppressing nucleation of the off-target shapes. The tile set itself stays the same, so with the SHAM system, the set of images will be classified into three groups according to the resulting shapes H, A, and M. However, generalization to a greater number of classes/shapes is straightforward, just requiring the design of a multifarious system with more possible shapes.

Several decisions must be made in order to numerically define the objective function for optimization. How should nucleation rates be predicted? By what metric should deviations from the ideal be measured? Should additional criteria be incorporated to address experimental concerns? We will use a score based on maximizing the ratio of predicted nucleation rates using a fast and simple approximation, adjusted to impose preferences for locating nucleation near areas that worked well in flag experiments, and to impose penalties against using high concentrations for fluorophore / quencher strands and their neighbors.

The overall score for optimization will be based on a modification of the Stochastic Greedy Model's critical ensemble nucleation barrier energy estimate G_{ce}, simplified and adjusted (using penalties $w_{x,y}$) to predict low nucleation rates for empirically poor areas in each shape. This modification is described below as the Window Nucleation Model. Thus, given a pixel-to-tile map,

$$\mathrm{Score}(\mathrm{image}, \mathrm{shape}) = G^{\approx}_{\mathrm{ce}}(\mathrm{image}, \mathrm{shape}) \tag{2.22}$$

gives a score that is lower for faster-nucleating cases. Since the nucleation rate is exponential in G_{ce}, the difference in scores between the target shape and its closest competitor is the log of the ratio of their nucleation rates. This gives a score for the image, in which we also add penalty for tiles that should remain at low concentrations.

$$\mathrm{Score}(\mathrm{image}) = \mathrm{Score}(\mathrm{image}, \mathrm{target\ shape}) - \min \mathrm{Score}(\mathrm{image}, \mathrm{other\ shape}) + \sum_i d_i f_i \tag{2.23}$$

where $f_i = c_i/c$ is the concentration of tile i relative to the base concentration c, so $1 \leq f_i \leq 27$. This per-image score is also lower for images predicted to perform better. The penalties d_i are detailed below.

Finally, these scores were combined for all 18 training images to give a total score for that assignment map:

$$\mathrm{Score}(\mathrm{overall}) = \max_{\mathrm{images}} \mathrm{Score}(\mathrm{image}) \tag{2.24}$$

That is, we try to optimize the pixel-to-tile map by improving the worst-performing image, in hope of obtaining a system whose performance is reasonably consistent across the training images.

Specifically, the overall score was minimized using a naive, parallel "hill-climbing" (in our case, hill-descending) algorithm. Moves consisted of attempted swaps of randomly-chosen pairs tiles, accepted when the score decreases. 72 parallel hill-climbing processes attempted to minimize the score, but every 100,000 steps, all hill-climbing processes switched their current state to the best state any of them had found so far.

After minimization with the Window Nucleation Model, we continued with a slower fine-tuning optimization stage using the Stochastic Greedy Model's estimate for G_{ce} instead of $G^{\approx}_{\mathrm{ce}}$. (Thus, the position-dependent $w_{x,y}$

penalties were no longer applied, although the tile-specific concentration penalties d_i were still applied.) At the time of systems design, this process used an older version of model, in which (i) $G_{\rm se} = 7.0$ and $G_{\rm mc} = 12.75$, (ii) $G_{\rm ce}$ was estimated from just 50 trajectories, each of which took only 14 unfavorable steps rather than completing the entire shape, and (iii) there was no cutoff for the final G of the trajectory. Additionally, each parallel hill-climbing process synchronized with others every step, as each step was quite slow so synchronization adds little delay. During the fine-tuning step, ~ 25 tiles were swapped.

To promote consistent levels and behavior of fluorescence signals, we planned to use fluorophore- and quencher-labeled strands at the base (i.e. lowest) concentration for each experiment; therefore, we wanted to restrict the pixel-to-tile map so as to accommodate this choice. The tiles with non-zero tile-concentration penalty d_i were as follows: locations with fluorophore labels (d_i = 1,000), locations with quenchers ($d_i = 1$), and tiles adjacent to flourophore-quencher pairs that bound to both the fluorophore and quencher ($d_i = 0.5$). Because the system used 917 tiles and 900 pixel locations, there were 17 assignments that were, for any pattern, always at base concentration. Consequently, the 12 fluorophores, with such a high penalty, always ended up with those assignments, while some quenchers and adjacent tiles were not always at base concentration, but were optimized toward the lower end of concentrations.

In experiments, both fluorophore- and quencher-labeled strands were used at the base concentration, regardless of the concentration implied by the corresponding image pixel (but adjacent tiles were used at the pixel-specified concentration). Accordingly, during training the nucleation rate estimates used base concentrations for fluorophore- and quencher-labeled strands, regardless of the value of the pixel indicated by the current pixel-to-tile map (but again adjacent tiles used pixel-specified concentrations).

2.5 Window Nucleation Model

To optimize the pixel-to-tile map for training pattern recognition by nucleation, we need a fast inner loop, and thus it is infeasible to use the full Stochastic Greedy Model. Finding a pixel-to-tile map that will work well experimentally does not necessarily require an accurate nucleation rate estimate; any function that provides a sufficiently similar rank ordering should be usable. Our choice was to use the simpler and faster Window Nucleation Model, which can be interpreted as calculating $G_{\rm ce}$ as in equations 2.18, but for a boundary set of 'critical nuclei' S that consist of every $k \times k$ region ('window') in the shape, and with the sum over $J^+(A)$ replaced by a single constant concentration, the base concentration c. In that case, we approximate

$$G_{\rm ce} \approx -\ln \sum_{A \text{ is } k\times k} \frac{c}{u_0}\, e^{-G(A)} = -\ln \sum_{A \text{ is } k\times k} \frac{c}{u_0}\, e^{\alpha + B\, G_{\rm se} - \sum_{i\in A} G^i_{\rm mc}} = \text{const} - \ln \sum_{A \text{ is } k\times k} \prod_{i\in A} f_i \qquad (2.25)$$

where $c_i = u_0\, e^{-G^i_{\rm mc}+\alpha} = c\, f_i$ and $c, B, G_{\rm se}$, and α are constants that can be factored out as an additive offset to $G_{\rm se}$.

The Window Nucleation Model calculates a score related to this approximation for $G_{\rm ce}$, but also including position-dependent weights $w_{x,y}$ to encourage pattern recognition to make use of tiles that were demonstrated to work well in the flag scan experiments. Given a pixel-to-tile map θ to be evaluated, tile concentrations are stored in a zero-indexed $L \times L$ array as $f(x,y) = c_i/c$ where i is the tile at position (x,y) within the shape, or $f(x,y) = 0$ if the position is outside the shape. The concentration $c_i = c\, e^{3\, p_n \ln 3}$ is determined from the pattern image being evaluated based on $i = \theta(n)$, with p_n being the grayscale level for image pixel n, and the base concentration is $c = 16.67$ nM. Then the score for window size k is calculated as:

$$\text{Score(image, shape)} = -\ln \sum_{X=0,Y=0}^{L-k,L-k} \exp \sum_{x=X,y=Y}^{X+k-1,Y+k-1} w_{x,y} \ln f(x,y) = -\ln \sum_{X=0,Y=0}^{L-k,L-k} \prod_{x=X,y=Y}^{X+k-1,Y+k-1} [f(x,y)]^{w_{x,y}} \qquad (2.26)$$

where $w_{x,y}$ is a weight (by default equal to 1) provided for each location in the shape to roughly incorporate experimental observations about which areas nucleated better than others. Because $f(x,y) \geq 1$, a weight $w_{x,y} > 1$ will result in a more negative score, i.e. a prediction of faster nucleation, whenever the relevant tile is above the base concentration. Note that the contribution to the nucleation score is zero for window regions that extend beyond the boundaries of the shape. Also note that temperature, in the form of its influence on $G_{\rm se}$, does not come into play in this score, as bond energies can be factored out as a constant independent of position and pixel-to-tile map. Because temperature does influence the size of critical nuclei, our choice of k has implications on the range of temperatures for which the Window Nucleation Model will be reasonably well correlated with actual nucleation. The area K discussed in the main text as the size of a putative critical nucleus here corresponds to $K = k^2$.

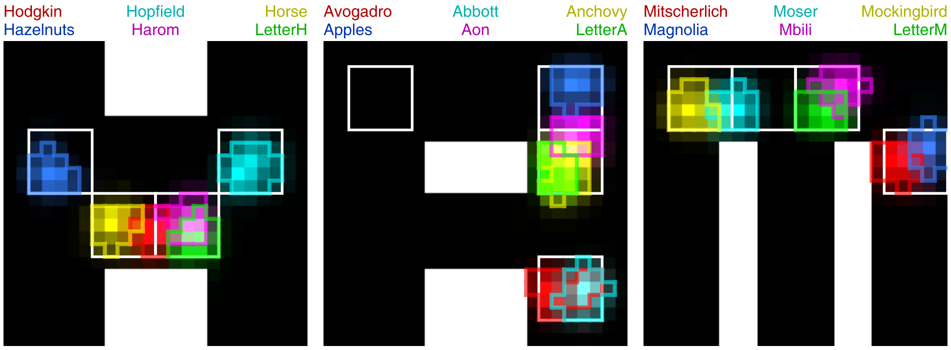

Figure S2.5: Nucleation heatmaps for the indicated training patterns are superimposed on their respective target shapes. The color of an (x, y) location is the RGB sum of adjusted heatmap greylevels (linearly proportional to the spatial nucleation rate $\eta^+_{x,y}$ for the given shape and pattern, but adjusted for visual clarity) times the given pattern's indicated color. Colored outlines indicate the smallest region accounting for 75% of nucleation for the given pattern. White square outlines indicate the preferred regions with weighting $w_{x,y} = 2$ during training.

For pattern recognition training, the window size was $k = 4$. This was chosen to be slightly smaller than the 5×5 flag size, which we knew could be sufficient for selective nucleation, because in the pattern recognition setup the high-concentration tiles need not be restricted to a checkerboard arrangement within the window; however, we make no claims of optimality here. The position-dependent weight was 2 when considering tiles in a target shape for a particular pattern within the 5×5 contiguous region of the following flags: H flags 2, 6, 8, and 10; A flags 1, 9, 10, and 12, and M flags 1, 5, 9, and 11, and was otherwise 1. When considering tiles in an off-target shape, all weights were 1. The goal of this weighting was to push high concentration regions for nucleation of the target shape into regions shown to work well in the flag-scan experiments (Fig. 4d), in the sense of having strong on-target nucleation and growth, but low off-target nucleation and growth.

The training and weighting was effective at locating pattern-specific nucleation to the preferred regions, as estimated by the Stochastic Greedy Model. Figure S2.5 show color-coded and superimposed nucleation heatmaps of $\eta^+_{x,y}$ for each training pattern on its target shape. (Individual nucleation heatmaps can be found in Section YYY, including heatmaps for nucleation on the off-target shapes.) Because there were 4 preferred regions for each shape, but 6 training patterns targeted to each shape, the strong effect of the position-dependent weights resulted in confining nucleation to the preferred regions, and thus forcing more than one shape to share the same nucleation region in some cases. Examination of how these patterns embed in the shapes (see Section 6.4), we see that although the same or overlapping nucleation regions are used, the highest-concentration tiles used by each pattern are generally different (c.f. Avogadro vs Abbott). This suggests that if all locations worked well for nucleation, there would be considerable potential for storing and recognizing more patterns per class in future improved designs that distribute nucleation throughout the shapes. (At this point we do not know whether the inferior nucleation locations were due to strand synthesis, sequence design, or other factors.)

2.6 A simple alternative pattern recognition training method

The training method discussed in Section 2.4 uses scores based on models of nucleation, together with an hill-climbing algorithm, to try to optimize the nucleation rate of the target shape for each training image compared to the nucleation rates of off-target shapes. When the method was developed, we were uncertain as to whether pattern recognition by nucleation, especially in this reservoir computing context, would be possible at all, and therefore we aimed for a method that used our most accurate nucleation models to attempt to ensure the highest discrimination ratio. It worked for our purposes, but the method is very expensive computationally and has no guarantees of finding the optimal pixel-to-tile map.

A kind reviewer suggested a simpler and faster algorithm, which we have evaluated and confirmed ought to be effective in many cases. The method is predicated on the assumption that the training patterns can be recognized based on just the co-occurrence of their few highest-intensity pixels. Specifically, for each training image the highest w^2 pixels will be assigned to tiles in a unique, contiguous $w \times w$ region of the target shape, if possible. Unlike the method used for our experimental implementation, this method does not rely on any model of nucleation, or exploration of the space of pixel-to-tile maps. In fact, the method is simple enough that the training could be done

by hand:

- For each training image, pixel locations are sorted by pixel value, from highest to lowest. The w^2 highest value pixel locations that are not already assigned to tiles are used for assignment.
- For each training image, all possible contiguous $w \times w$ regions in the target shape are checked, in random order, for whether the tiles in the region are all unassigned. When a region with no previously-assigned tiles is found, the unassigned high-value pixel locations in the training image are assigned, in left-to-right, top-to-bottom order, to the tiles in the region. If no suitable region is found after all possibilities are checked, regions with only one previously-assigned tile are searched for, then two, etc, until a region is found. When a region with some previously-assigned tiles is used, these tiles are skipped during assignment, and not all w^2 pixels are assigned at this point.
- Once all images have had pixels assigned to tiles with this process, any remaining unassigned pixels and tiles are assigned randomly.

The parameter w is chosen by the user to reflect the expected critical nucleus size for the concentrations and annealing speed to be used.

Unlike the nucleation-model-based training methods, this simple method has straightforward limits on the number of images that can be successfully trained if using the full complement of w^2 pixel-to-tile assignments per image is required, because no pixel or tile can be used to recognize more than one image and each trained image will have a unique intended nucleation region. Specifically, for images with P pixels and with shape s having N_s tiles, the number of training images is limited by P/w^2 (as each image assigns w^2 pixels) and the number of training images classified as shape s is limited by N_s/w^2 (because each such image assigns w^2 tiles). A more sophisticated algorithm could attempt to re-use pixel-to-tile assignments for multiple images and shapes.

We have no theoretical performance guarantee for this algorithm, and we assume that its effectiveness depends not only on the nature of the images but also the particularities of how tiles are arranged in the shapes. Analysis might be possible for random images and shapes with random arrangements of tiles. One would expect, for example, that even before the above hard limits are reached, the last-to-be-added images will have many of its highest-value pixels already assigned by the processing of previous images, so only lower-value pixels will be used in its assigned nucleation region. Furthermore, as shared tiles are assigned, their locations spread throughout other shapes will also mean that later images in the process will be less likely to have a full w^2 region of high concentration tiles assigned for nucleation. Thus the expected performance of pattern recognition will smoothly degrade as more images are added.

Surprisingly, however, this simple assignment method compares favorably to the model-based method in the capacity measurements of Extended Data Fig. E8. Note however that the algorithm is very sensitive to the choice of w with respect to the experimental conditions, or their approximation by the Stochastic Greedy Model with a specific choice of parameters. For this reason, we increased G_{se} to 5.5 for assessing the selectivity in Extended Data Fig. E8. Even so, the case of $w = 2$ is not plotted because accuracy never exceeds 70% even with just one image, as the "experimental conditions" here require larger critical nuclei. On the other side, for w larger than the experimentally relevant size, the number of images that can be recognized is bounded by the use of w^2 tiles and pixels per shape, as per the hard limits discussed above. Thus, unlike the naive hill-climbing optimization, this simpler assignment algorithm performs worse as w is increased beyond the ideal value.

As a caveat to the interpretation of the Extended Data Fig. E8 capacity measurements, it should be appreciated that the choice of a constant temperature ($G_{\text{se}} = 5.4$ for the WNM-trained pixel-to-tile maps and $G_{\text{se}} = 5.5$ for the alternate assignment method presented in this section) does not perfectly reflect the principles governing selectivity in an experiment where the temperature is ramped down slowly. In that scenario, the winning shape would be the one that first achieves a nucleate rate that is sufficiently high relative to the annealing rate. Future analysis of pattern recognition capacity should incorporate this consideration.

It is also worth noting that this simple algorithm does not perform well for the MNIST pattern recognition task discussed in Section 1.4, as that task involves balancing trade-offs for classifying a large number of similar patterns, so memorizing them individually is insufficient.

2.7 Training and testing image sources and processing

In our work here, we also placed restrictions on the images to be recognized. First, all images used for training and testing should make use of the same total concentration, so as to control for the strong effect that absolute concentration has on nucleation – we are interested in conditions where the *pattern* of relative concentrations is the deciding factor. Second, all images should have the same histogram of pixel values, so as to control for the fact that a combination of high concentration and low concentration tiles can be more effective than uniform concentrations with the same total. This second restriction is more stringent and implies the first. Neither is necessary in principle for pattern recognition by nucleation, but eliminate some possible confounds for interpreting this first foray into the phenomenon. Expanding our understanding of pattern recognition by nucleation into the full space of possible images could reveal novel properties; for example, we might expect dilutions of a concentration pattern to be classified equivalently for a suitably slow anneal until the first nucleation occurs.

The particulars of image processing in this work are as follows. To allow reasonable mixing of reagents by the acoustic liquid handler we used, which had a discrete volume step of 25 nL, pixel values for each image were binned into 10 bins. As described in the Methods, pixel values $0 \leq p_n \leq 1$ were mapped exponentially to tile concentrations, using $c_i = c\, e^{3\, p_n \ln 3}$, where base concentration $c = 16.67$ nM, c_i is a tile concentration and $i = \theta(n)$. Pixel value 0 corresponds to black and 1 to white. Because of pixel value binning, the pixel values were $x/9$ for integer $x \in [0, 9]$, which similarly resulted in 10 bins for tile concentrations, between 16.67 and 450 nM.

Images for the pattern recognition experiments came from a wide variety of sources. As described in Methods, each was cropped and rescaled to a 30×30 array, with grayscale levels adjusted to match a consistent histogram. We selected images that could be categorized into three classes based on the first letter of their names, with class labels and shapes 'H', 'A', and 'M' chosen to reflect 'Hopfield Associative Memory', the foundational inspiration for our perspective on multifarious self-assembly. Images were downloaded and processed in September, 2019. Quotes are from the given link or from Wikipedia.

Hodgkin. `https://www.gettyimages.com/detail/2672529`.
Image of Dorothy Mary Crowfoot Hodgkin licensed from Keystone / Getty Images. Hodgkin "was a Nobel Prize-winning British chemist who advanced the technique of X-ray crystallography to determine the structure of biomolecules, which became essential for structural biology." Our self-assembly work relies on the formation of geometrically ordered structures that would produce point-based diffraction patterns in the limit of large structures, much like classical homogeneous crystals. However, our structures have different DNA sequences at different sites and hence are not periodic if the sequence identity is taken into account. Our structures can be seen as a generalization of crystals to the multicomponent limit where the number of distinct species is comparable to the system size.

Hopfield. `https://alchetron.com/John-Hopfield`.
Permission for using this image obtained directly from John Joseph Hopfield and from Princeton University. Hopfield "is an American scientist most widely known for his invention of an associative neural network in 1982." Hopfield's Associative Memory inspired the concepts underlying this work, even though the our work exploits an inevitable physical process (nucleation) to perform pattern recognition without mimicking Hopfield neural networks element-by-element.

Horse. `https://pixabay.com/photos/horse-mold-thoroughbred-arabian-2063672`.
Pixabay's license is "free for commercial use, with no attribution required." The Arabian horse is "is also one of the oldest breeds, with archaeological evidence of horses in the Middle East that resemble modern Arabians dating back 4,500 years."

Hazelnuts. `https://pixabay.com/photos/hazelnut-nuts-legume-nut-healthy-3357096`.
Pixabay's license is "free for commercial use, with no attribution required." As of this writing, the oldest "evidence of large-scale Mesolithic nut processing, some 8,000 years old, was found in a midden pit on the island of Colonsay in Scotland."

Harom. `http://www.pymvpa.org/datadb/mnist.html`.
"Harom" is the word for "3" in Hungarian. The image is from the MNIST database.

H. `https://www.nist.gov/itl/products-and-services/emnist-dataset`.
The image of the letter "H" is from the EMNIST database.

Avogadro. `https://colenda.library.upenn.edu/catalog/81431-p34q7qz9q`.
Drawing of Lorenzo Romano Amedeo Carlo Avogadro by C. Sentier in 1856, from the Edgar Fahs Smith Image Collection at the University of Pennsylvania. Avogadro "was an Italian scientist, most noted for his contribution to molecular theory now known as Avogadro's law." His law tells us how many parallel pattern

recognition events ($\sim 10^{11}$) occurred in each macroscopic test tube sample (i.e. how many nuclei have formed at the time of 10% signal quenching), making us wonder how small a system could carry out the same task.

Abbott. https://www.frontiersin.org/journals/neuroscience.
Permission for using this image, downloaded from the Neuroscience journal Editorial Board list, was obtained directly from Laurence Frederick Abbott. Abbott is an American theoretical neuroscientist, known for his studies of neural adaptation and plasticity, including reservoir computing and competitive Hebbian learning in networks of spiking neurons. While we trained our molecular systems a la reservoir computing using a computer, our design using interaction mediating particles potentially allows learning interactions through a chemical Hebbian process in future work.

Anchovy. https://www.flickr.com/photos/noaaphotolib/5187467373/.
Photo from NOAA/NMFS/SEFSC Pascagoula Laboratory; Collection of Brandi Noble, NOAA/NMFS/SEFSC. Licensed under the Creative Commons Attribution 2.0 Generic license. The Dusky anchovy (Anchoa lyolepis) "is a species of anchovy native to the western Atlantic Ocean from New York to Brazil."

Apples. https://www.publicdomainpictures.net/en/view-image.php?image=17402&picture=green-apples.
Photo by Marina Shemesh, available under the CC0 Public Domain dedication. The Granny Smith "is an apple cultivar which originated in Australia in 1868. It is named after Maria Ann Smith, who propagated the cultivar from a chance seedling."

Aon. http://www.pymvpa.org/datadb/mnist.html.
"Aon" is the word for "1" in Scottish Gaelic. The image is from the MNIST database.

A. https://www.nist.gov/itl/products-and-services/emnist-dataset.
The image of the letter "A" is from the EMNIST database.

Mitscherlich. https://library.si.edu/image-gallery/73676.
Engraving of Eilhard Mitscherlich by William C. Sharpe, 1860, obtained from the Smithsonian Institute Libraries, which asserts no copyright. Mitscherlich "was a German chemist, who is perhaps best remembered today for his discovery of the phenomenon of crystallographic isomorphism in 1819 [...] His investigation, also in 1826, of the two crystalline modifications of sulfur threw much light on the fact that the two minerals calcite and aragonite have the same composition but different crystalline forms, a property which Mitscherlich called polymorphism." The multifarious S+H+A+M molecular mix, capable of three distinct geometric ordered assemblies, can be seen as a generalization of the crystal polymorph concept to the limit of a large number of distinct species.

Moser. https://www.flickr.com/photos/kavli-ntnu/37030995162.
Permission for using this photograph by Bård Ivar Basmo / Kavli Institute for Systems Neuroscience at the Norwegian University of Science and Technology, was obtained directly from May-Britt Moser. Moser, with "her then-husband, Edvard Moser, shared half of the 2014 Nobel Prize in Physiology or Medicine, awarded for work concerning the grid cells in the entorhinal cortex, as well as several additional space-representing cell types in the same circuit that make up the positioning system in the brain." The collective dynamics of the multifarious self-assembly model explored here is mathematically related to place cell network models that simultaneously encode multiple spatial memories through different colocalization of place fields.

Mockingbird. https://pixabay.com/photos/polyphonic-mockingbird-2232092.
Pixabay's license is "free for commercial use, with no attribution required." The northern mockingbird, commonly found in North America, "is known for its mimicking ability, as reflected by the meaning of its scientific name, 'many-tongued thrush'."

Magnolia. https://commons.wikimedia.org/wiki/File:Magnolia_flower_Duke_campus.jpg.
Licensed under the Creative Commons Attribution 3.0 Unported license and further permission granted by the photographer, David C. Richardson, emeritus professor of biochemistry at Duke University. The southern magnolia (Magnolia grandiflora) "is a tree of the family Magnoliaceae native to the southeastern United States."

Mbili. http://www.pymvpa.org/datadb/mnist.html.
"Mbili" is the word for "2" in Swahili. The image is from the MNIST database.

M. https://www.nist.gov/itl/products-and-services/emnist-dataset.
The image of the letter "M" is from the EMNIST database.

2.8 Mixes for pattern recognition experiments

Sample mixes were designed to be possible using a particular order of automated intermediate mixes, first creating mixes of tiles at each concentration value (discretized by the pixel value bins) for each pattern, then mixing these to obtain a full sample mix for each pattern. Absolute tile concentrations were set to have a target average tile concentration of 60 nM. This resulted in slightly different discrete tile concentration bins than were used in the nucleation model. Additionally, the discrete volume steps used by the acoustic liquid handler resulted in slightly different concentrations in experiments than the target concentrations, as shown in Table S2.1. The actual concentrations (nominally) dispensed by the liquid handling robot are different from the model concentrations by no more than 3.2 %.

Table S2.1: Tile concentrations for pattern recognition.

Factor	Model (nM)	Target (nM)	Actual (nM)
1.00	16.67	17.08	16.87
1.44	24.04	24.63	24.51
2.08	34.67	35.53	35.71
3.00	50.00	51.24	51.32
4.33	72.11	73.90	73.60
6.24	104.00	106.58	107.14
9.00	150.00	153.72	153.76
12.98	216.34	221.70	223.06
18.72	312.01	319.75	321.43
27.00	450.00	461.16	464.29

Section 3

Fluorescence readout, shape layouts, and simulated structures

To assess the progress of nucleation and growth in our system, we used a combination of atomic force microscopy (AFM) and real-time fluorescence, each of which comes with strengths and limitations. The obvious strength of AFM is that we can see individual assemblies and collect statistics on their morphologies, in some cases with single-tile resolution; limitations include that it can be time-consuming to prepare samples and take images, that analysis can be hard to automate, and that deposition onto mica introduces biases: in our case, we adjusted conditions such that single tiles and small assemblies did not stick well and were washed away. For these reasons we limited AFM to end-point measurements. The obvious strength of real-time fluorescence is that it reports bulk averages as a function of time, thus providing insight into kinetics; limitations include that exactly what the fluorescence level is reporting sometimes can be hard to discern, and that modifications required to enable fluorescent readout may alter the behavior of the system. Addressing and understanding these limitations, for our experimental system, required us to explore and characterize several designs for fluorescent readout.

Due to the wide availability of real-time quantitative PCR (qPCR) machines, with their ability to precisely control temperature while measuring multicolor fluorescence in real time on parallel samples, they have become a useful tool for quantitative studies of thermodynamics and kinetics in nucleic acid biophysics and molecular programming.[22,129,130] Ideally, the use of a qPCR machine would allow the quantitative measurement of the absolute or relative concentrations of free and bound tiles of multiple chosen types simultaneously throughout the course of self-assembly. Our particular aim was to simultaneously monitor four locations per shape, so as to be able to observe and distinguish both the timing of initial nucleation and the progress of further growth. However, there are several phenomena that present obstacles to converting from fluorescence measurements to absolute (or even relative) concentrations in our experimental conditions. Previously used methods for fluorescent monitoring of DNA self-assembly did not appear to be suitable for our needs. Self-assembly of 2D and 3D DNA nanostructures made of double-crossover and single-stranded tiles has been monitored using the SYBER Green I intercalating dye,[111,121] but this method generically assesses single-stranded versus double-stranded rather than being easily targetable to specific locations within a structure. Fluorescence resonance energy transfer (FRET) between fluorophore pairs covalently attached to specific strands can provide proximity-dependent signals,[98,114] but for multiplexing, each signal occupies more wavelength real estate than a single-fluorophore setup, and these studies monitored only a single position within the DNA object.

3.1 Initial fluorophore design

Inspired by dual-labeled fluorescent probes such as TaqMan probes and molecular beacons,[131] we initially used a single tile modified to contain a 5′ fluorophore and a 3′ quencher, as shown in Figure S3.1(a). When unbound, the modified strand was expected to allow free movement of the fluorophore and quencher without confining them to be near enough to cause significant quenching. When bound in a lattice, however, the the two ends would be on adjacent helices and oriented so as to be near each other. This constrained position would only result from a sufficiently large structure and not, for example, from dimers: thus quenching was expected to indicate the presence of a well-formed lattice structure around the labelled tile. By modifying only a single tile, we also hoped to minimize changes to nucleation and growth of the overall structure. We refer to a tile with a fluorophore and quencher as a 'label tile'.

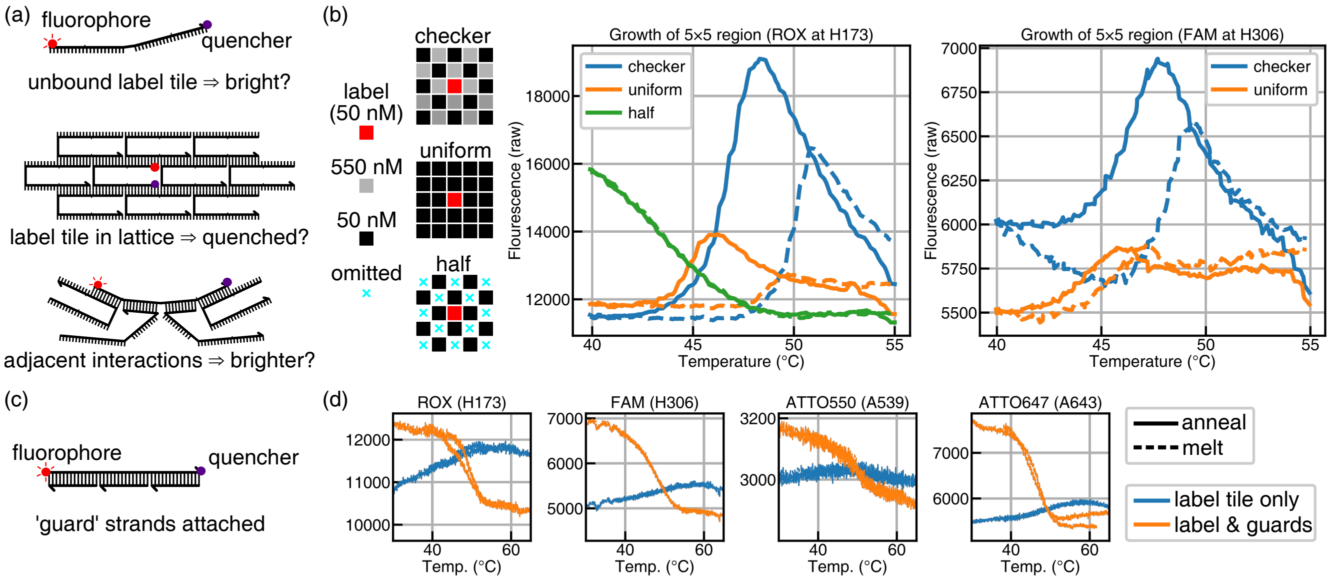

Figure S3.1: An initial design for a label tile consisting of a fluorophore and quencher on a single SST. **(a)** As a free unstructured strand, the fluorescence will not be fully quenched. **(b)** To examine the label tile's behaviour, we used three patterns around a 5×5 set of tiles from shape H, surrounding ROX on tile H173 or FAM on tile H306. The 'checker' pattern had high concentrations of tiles adjacent to the label tile, while the 'half' pattern omitted a checkerboard pattern of tiles such that the full 5×5 structure could not form. An anneal and melt of the patterns around a ROX label tile and FAM label tile shows a significant *increase* in fluorescence during the anneal. **(d)** To examine whether DNA binding to the label tile 'stretched' the strand, reducing self-quenching, we used 'guard' strands that each bound to a third of the tile. **(e)** For all four fluorophores, samples with guard strands showed a significant increase in fluorescence during the anneal around 50 °C.

Using this 'label tile' design in initial experiments, we found that while changes to fluorescence occurred near expected growth temperatures, the changes were not as simple as a transition from a bright unbound state, to a quenched bound state. Using 5×5 sets of tiles around label tiles to characterize their behaviour by growing small structures during annealing experiments, we found that fluorescence significantly increased when ramping from temperatures well above melting to temperatures around those favourable for growth, before quenching at lower temperatures, but only to levels slightly below those seen at high temperatures (Figure S3.1(b), 'checker' and 'uniform'). In contrast, samples missing half the tiles required for structure formation, but with adjacent tiles that could bind to the label tile, showed an increase in fluorescence as temperature decreased (Figure S3.1(b), 'half').

These results led us to suspect that, when unbound and single-stranded, the fluorophore and quencher on the label tile were both not constrained by the DNA to be close enough to quench fully, but were also not constrained to be far apart, and thus tended to interact and quench at an intermediate level. However, when some domains on the tile were bound to those on other tiles, whether in the course of nucleating a larger lattice structure or simply binding to complementary regions, the increased stiffness from the double-stranded regions did force the fluorophore and quencher apart, stopping this quenching and raising the fluorescence signal. Figure S3.1(a) shows an example intermediate structure that can occur in all the samples, but which will continue to assemble into a fully-quenched 5×5 square in all but the 'half' sample. This interpretation is consistent with the understood mechanism by which TaqMan probes, which typically have a baseline fluorescence several fold higher than molecular beacons that use a hairpin stem to bring fluorophore and quencher together, enhance their fluorescence upon binding to a target.[131]

To verify this interpretation, we used 14-nucleotide 'guard strands' complementary to the label tiles as shown in Figure S3.1(c), such that when added to a sample containing only the matching label tile, they would cause the tile to be double-stranded at low temperatures. For all our label tiles, this addition resulted in higher fluorescence at temperatures lower than the guard strands' ~ 50 °C predicted[132] melting point, while the label tiles by themselves showed relatively little temperature dependence (Figure S3.1(d)).

While it was possible to distinguish the formation of structures through the increased and decreased fluorescence during anneals, as in S3.1(b), the complexity and inconsistency of the label tile's behaviour during nucleation motivated a second design, which was used for all subsequent experimental results.

3.2 Final fluorophore design

To avoid fluorophore-quencher interactions outside of lattice formation, our second design, shown in Figure S3.2(a), placed the fluorophore and quencher on two separate tiles - a 'label pair' - with no complementary regions. In addition to avoiding the intra-tile interactions of the previous design, the two tiles in a label pair cannot form a dimer that might exhibit quenching, even at relatively low temperature. While normal SSTs in this configuration would not have terminal ends close enough for contact quenching, in this design, one of the two tiles had its orientation reversed, with a crossover in a lattice on the opposite side; as a result, in a well-formed lattice, the fluorophore and quencher are located on facing sides of adjacent helices. The same tile, when used without a fluorophore, does not use the reversed orientation.

Using this label design, formation of structures caused a monotonic corresponding decrease from an unquenched, high fluorescence, to a quenched, low fluorescence signal, subject to the temperature dependence of the fluorophore itself (data not shown). This behaviour was seen with reasonable consistency in preliminary multifarious growth experiments, and could be compared with AFM images of the resulting structures.

Given these results, we might hope to construct a formula f linking fluorescence to assembled (as opposed to free) tile concentration, e.g. $c_i^{assem} = f(c_i, F, T)$, for a particular fluorescent label tile i, fluorescence reading F, temperature T, and total tile concentration c_i. Doing so would be possible using suitable calibration experiments if, for example, free tiles and assembled tiles each had well-defined fluorescence levels at each temperature, independent of other DNA molecules in solution. (Here, we take 'assembled tiles' to mean those within assemblies of at least four strands that adopt the lattice conformation that brings fluorophore and quencher close to each other, while by an abuse of terminology we take 'free tiles' to mean those that are free or bound to others in locally linear or tree-like chains that don't fold the fluorophore and quencher tiles as in the lattice. It would be the concentration of this population that we could infer.)

In initial control experiments (Figure S3.2(c)), we found that strands that bind directly to the fluorophore tile substantially increase its fluorescence, as expected. But more surprisingly, this effect only moderately decreases at higher temperatures where the binding is predicted to be unfavorable, as well as in the presence of unrelated DNA with little expected binding. To characterize this behaviour further, we took melting curves of a 5×5 square of 50 nM tiles around the ROX label pair in H, along with a variable amount of an equal mixture of tiles unique to H, outside a 7×7 region around the same label pair (Figure S3.2(b)). Owing to the roughly checkerboard pattern of shared and unique tiles, this unique tile mix approximated a collection of unrelated, non-interacting DNA, which was not complementary to either the tiles in the 5×5 target assembly, or to other strands within the mixture.

As shown in Figure S3.2(d), increasing the total concentration of unrelated, non-specific DNA (i.e., $\sum_i c_i$ for strand concentrations c_i) in a sample increased fluorescence at all temperatures, in a seemingly consistent way unaffected by the quenching when the 5×5 region grew and melted. The effect was present even when all assemblies were clearly melted, above 65 °C, and appears to be roughly linear between 0 and 20 ţM.

Knowing that in the absence of Tween, PEG, carrier DNA, or the equivalent (which we did not use in these experiments), low concentrations of DNA can partially absorb onto the walls of test tubes and pipette tips, we explored the hypothesis that this was related to our observations – that somehow the excess strands were playing the role of carrier DNA. While we used coated tubes designed to minimize DNA adsorption (0.5 mL DNA LoBind tubes, Eppendorf), and pipette tips (Eppendorf LoRetention), we considered whether fluorescently-labeled DNA (and assembled structures containing the label pairs) could have been absorbed to the walls when excess strands were not present, thus yielding low fluorescence measurements, but released from the walls when excess strands were available to replace them, thus increasing the measured fluorescence. Quantitatively, the observed effected seemed too large for this mechanism, and further investigation also ruled it out as a significant factor.

However, strands with regions complementary to regions on the fluorophore tile also appear to affect fluorescence, to a greater extent than non-specific DNA, and this effect also persists even at temperatures where no binding would be expected to be stable.

To measure this effect, we took melting curves of samples with all tiles for constructing H at 50 nM, along with a 10x concentration checkerboard flag pattern, either surrounding the label pair, or in a position far from the label pair. Both types of samples had the same total DNA concentration, but the sample with the label pair inside the high-concentration pattern, thus having high concentration of tiles adjacent to the fluorophore and quencher, had a significantly higher concentration of strands with regions complementary to regions on the fluorophore and quencher strands. In an anneal and melt, shown in Figure S3.2(e), the sample with high concentration adjacent strands had a significantly higher fluorescence above the melting temperature of the structure, but when structures had formed, had similar fluorescence. The raw fluorescence level at low temperatures, regardless of whether the excess tiles were adjacent or not, was consistent with that of the sample in Figure S3.2(d) that had the most similar

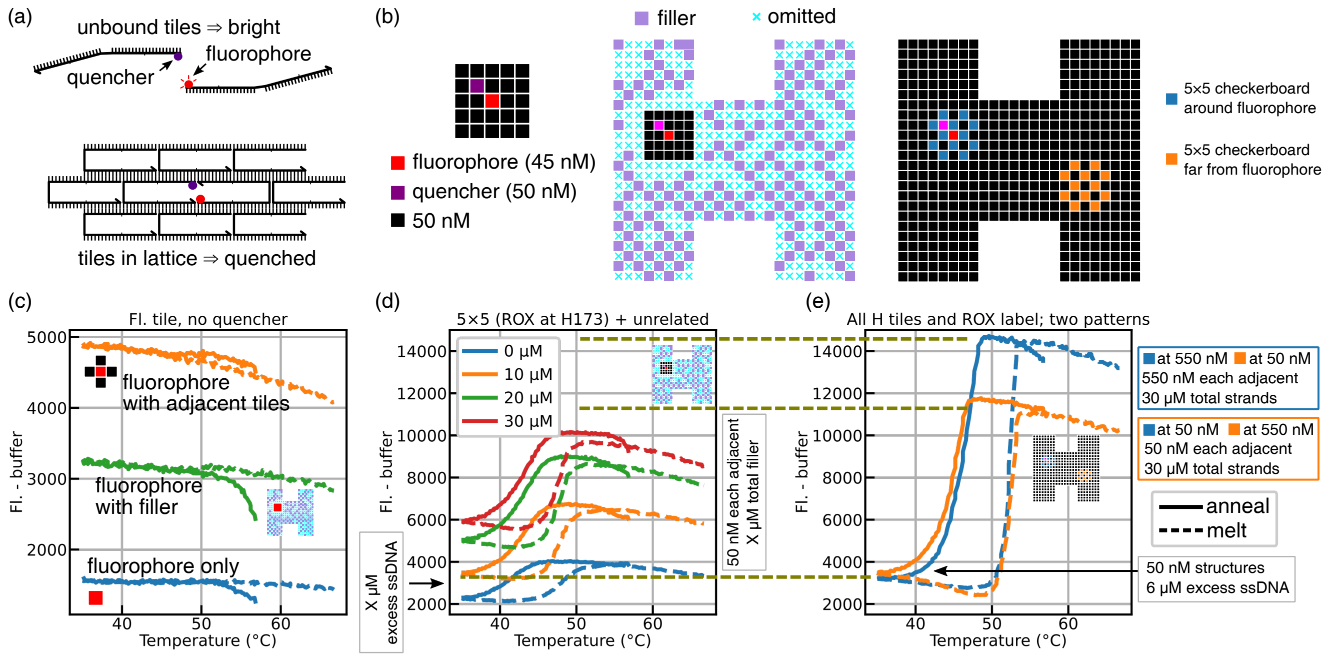

Figure S3.2: A two-tile label pair, separating the fluorophore and quencher, gives quenching from lattice growth without significant interference from intermediate states, but effects on fluorescence related to other strands complicate quantitative interpretation of fluorescence in terms of absolute tile and structure concentrations. **(a)** The label scheme uses two nearby tiles, along the same helices but not binding to each other directly, with one tile reversed in orientation, resulting in 5' modifications on the two tiles being constrained to be near to each other in a well-formed lattice, but not constrained in any potential complex of two or three tiles. **(b)** To characterize the label pair behaviour, we ran experiments with several combinations of components, based around a ROX fluorophore on tile H173 and a 5×5 surrounding area. A 'filler' mix included all tiles unique to H outside a 7×7 region around the fluorophore tile, intended to provide a mix of non-interacting tile-like DNA strands. Complete H structure samples used the same ROX fluorophore tile, with its adjacent quencher tile, and 5×5 area, but with one of two possible checkerboard patterns (blue or orange) of increased concentrations. **(c)** An anneal and melt with the (H173 with ROX) fluorophore tile and no quencher tile, including only the fluorophore tile, the fluorophore tile with 10 μM total filler concentration, and the fluorophore tile with each of the four tiles adjacent to it that each bind by one domain. We interpret the anomalously low fluorescence at the beginning of the anneal, which started by cooling from just below 60 °C, to the samples not yet having established equilibrium. **(d)** An anneal and melt of a 5×5 block with varying total concentration of filler tiles. The filler strands are expected to remain unbound at all temperatures. **(e)** An anneal and melt of a complete H structure, including the same label pair. At high temperature, the blue and orange samples are expected to have the same total concentration of strands, but different concentrations of tiles adjacent to the label. At low temperature, if structures have completely grown, the two samples are expected to have the same concentration of structures and of excess, unbound strands. For c-e, samples were held at 70 °C for 10 minutes, ramped to 57 °C at 1 °C/min, held at 57 °C for 20 minutes, ramped from 57 °C to 35 °C at 6 minutes per 0.2 °C, then ramped from 35 °C to 67 °C at 4 minutes per 0.2 °C. (Note that the extent of hysteresis depends on ramp speed and reflects the nucleation barrier kinetics and melting kinetics. We estimate a melting temperature for H, for 50 μM tiles, between 50 and 52 °C.)

	ROX	FAM	ATTO 550	ATTO 647N
H	H173, H148	H306, H281	H227, H252	H428, H445
A	A636, A647	A544, A527	A544, A528	A539, A550
M	M863, M855	M830, M836	M761, M754	M759, M767

Table S3.1: Fluorophore/quencher pair locations for each fluorophore and shape.

amount of excess DNA, suggesting a lack of sequence-specificity for fluorophores within assembled structures. This also rules out the possibility that the fluorescence increase was just due to unintentional excess of fluorophore tiles over non-labeled tiles (noting that label pair tiles were ordered purified, while all other tiles we used unpurified).

Our conclusions are as follows:

1. The mostly-quenched fluorescence levels of fluorophores within assembled structures, with a nearby quencher, increase similarly in the presence of unrelated or related single-stranded DNA in solution.
2. The mostly-unquenched fluorescence levels of fluorophores on free strands increase in the presence of unrelated single-stranded DNA, with further increases in the presence of related single-stranded DNA that has some sequence complementarity.
3. The free fluorophore tile's temperature dependence is small by comparison.
4. Accounting for these effects to obtain quantitative inference of tile and assembly concentrations from fluorescence data would be fraught.
5. In these experiments, we see that time/temperature of the formation and melting transitions is not very sensitive to the above factors influencing absolute fluorescence.

It seems likely that using FRET instead of direct fluorescence would alleviate some of these issues, at the cost of having fewer label pair tiles per sample. However, given the surprising influence of excess DNA even for fluorophore/quencher pairs within the assembled lattice context, it is not a foregone conclusion that FRET eliminate these confounds entirely.

As a separate matter, it is well-known that incorporation of fluorophores and quenchers can affect the thermodynamics of DNA hybridization and self-assembly reactions,[129] often being beneficial on the order of up to 2 kcal/mol. This is especially a concern for us, if the modified tiles are part of a critical nucleus that controls nucleation rates. Consistent with this, although also possibly influenced by the reverse orientation[133] of the fluorophore-labeled tile, the AFM-based counts for samples containing label pair tiles near the center of the nucleation region were often notably higher than those that used label tiles elsewhere but otherwise had the same tile concentration pattern (see the table in Section 5.3.1).

3.3 Shape layouts

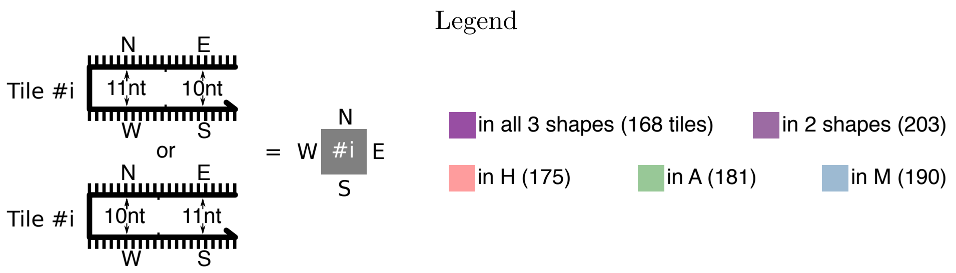

H: 480 tiles, 900 bonds, 175 unique tiles, 395 shared (168 all, 81 with A, 56 with M)

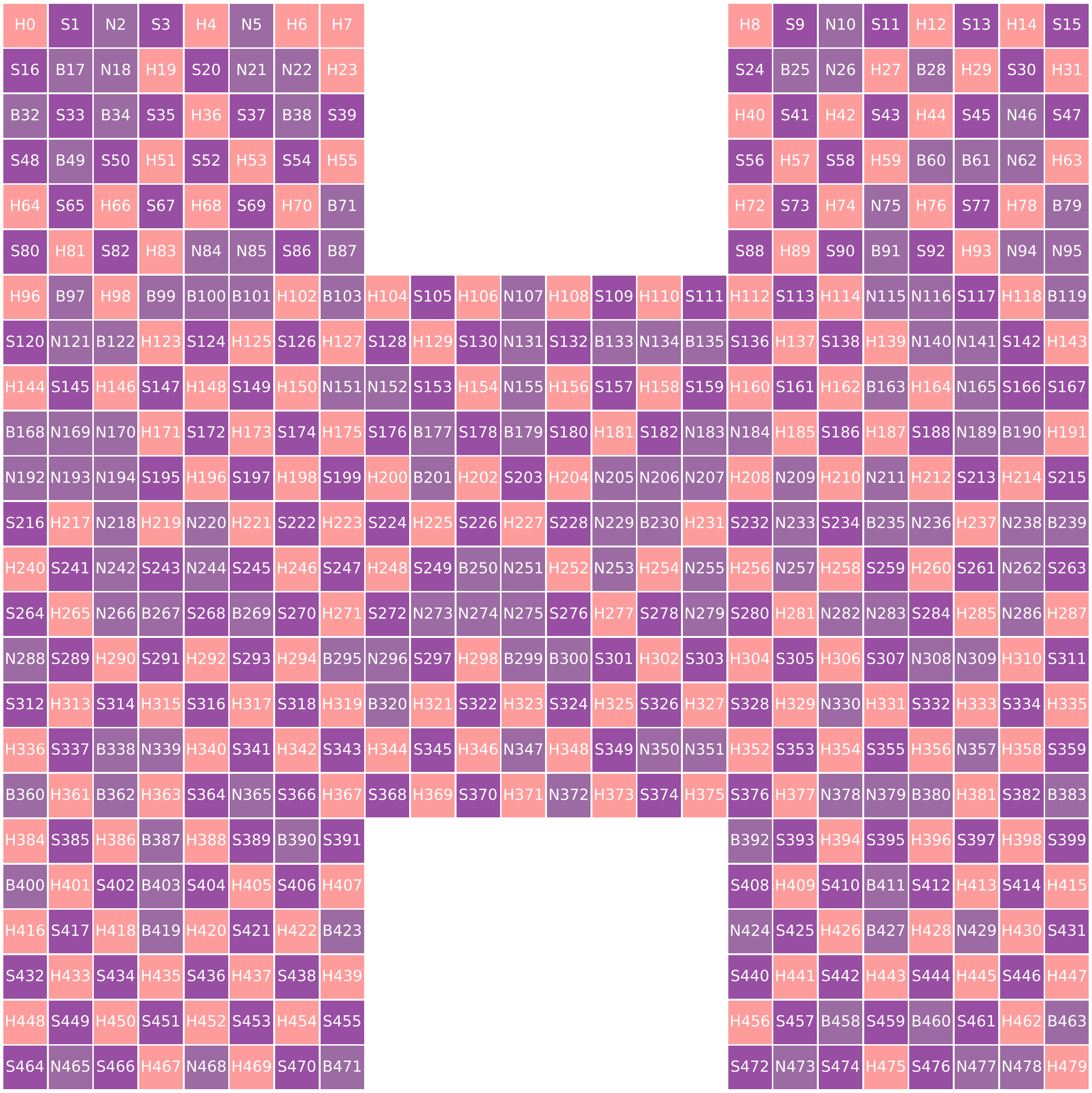

A: 496 tiles, 926 bonds, 181 unique tiles, 315 shared (168 all, 81 with H, 66 with M)

A480	A481	N10	S13	A482	N5	A483	I484	A485	I486	A487	S3	I488	S11	A489	A490	I491	S109	I492	I493	A494	S9	A495	S15
S120	I496	I497	I498	S301	A499	N351	A500	S345	I501	S303	A502	S126	A503	N115	A504	S213	A505	N251	A506	S305	A507	N282	A508
A509	S434	A510	S412	N229	S45	A511	I512	I513	S247	N308	S324	A514	N218	I515	S395	A516	I517	A518	S436	A519	S332	I520	I521
S80	N206	S50	A522	S188	N152	S90	I523	S54	A524	I525	I526	S278	A527	S451	A528	S222	A529	I530	A531	I532	A533	S382	A534
A535	S337	A536	I537	I538	S73	A539	S35	A540	S364	A541	N134	A542	S289	A543	S341	A544	S172	A545	N22	N296	S421	A546	S167
S24	A547	S343	A548	N207	A549	S457	A550	S203	A551	S410	A552	S406	A553	S138	A554	N62	A555	N309	A556	S124	A557	S314	A558
A559	S30	A560	S130	A561	I562	I563	N165	A564	S284	I565	S161	A566	S397	N169	S178	A567	S276	N350	S43	I568	N257	A569	S431
I570	A571	S385	A572	I573	A574	S293	A575	S476	A576	S466	I577	S474	N465	S368	A578	S186	A579	S241	A580	I581	A582	S132	A583
N192	I584	A585	I586	A587	S297	A588	S359									A589	I590	A591	N170	I592	S174	A593	S263
I594	A595	N151	A596	S234	A597	S249	A598									S312	N233	S322	A599	S366	I600	N255	I601
I602	S145	I603	N220	N21	S128	A604	I605									N424	S261	A606	S149	A607	I608	N379	S455
S440	A609	S449	A610	S199	A611	S270	N95									S408	A612	S20	A613	S402	A614	I615	A616
A617	S459	A618	S226	N262	S82	N116	S197	N2	N107	A619	S1	A620	S111	A621	S105	I622	N140	A623	S58	N121	S92	A624	S391
S216	A625	S33	A626	N75	I627	S291	A628	N339	N273	S349	A629	S159	N46	S417	A630	S228	A631	S318	A632	S259	A633	S376	A634
A635	S37	A636	S272	A637	S195	A638	N238	A639	S453	A640	S65	A641	N211	N131	N193	A642	S153	A643	S147	A644	S461	N189	I645
S432	A646	S245	A647	I648	A649	S280	A650	S326	A651	S232	A652	S182	A653	S180	N85	I654	A655	S353	I656	N18	A657	I658	A659
A660	S243	A661	S404	I662	S113	A663	N94	A664	S142	A665	S442	I666	S425	I667	N84	A668	N184	A669	N429	A670	S316	A671	I672
S16	A673	S41	A674	S446	A675	S52	N365	I676	A677	N372	A678	N478	N477	N468	A679	S438	S166	N357	I680	N253	A681	N286	I682
I683	S389	A684	S157	I685	S444	A686	S39									A687	N205	A688	N209	A689	N236	N141	S215
S88	A690	S393	A691	N274	A692	N330	I693									S56	A694	N155	A695	S355	I696	N266	A697
N288	N347	N279	S67	N183	N378	A698	I699									A700	N26	A701	S307	A702	S69	I703	S47
S264	N275	I704	A705	S117	N244	S86	A706									S48	A707	S334	A708	S136	A709	S414	A710
A711	S224	N194	I712	A713	S77	A714	S399									A715	S268	A716	S176	N242	S328	N283	S311
S464	A717	S470	A718	I719	A720	S374	A721									S472	A722	A723	A724	S370	N473	A725	A726

M: 480 tiles, 880 bonds, 190 unique tiles, 290 shared (168 all, 56 with H, 66 with A)

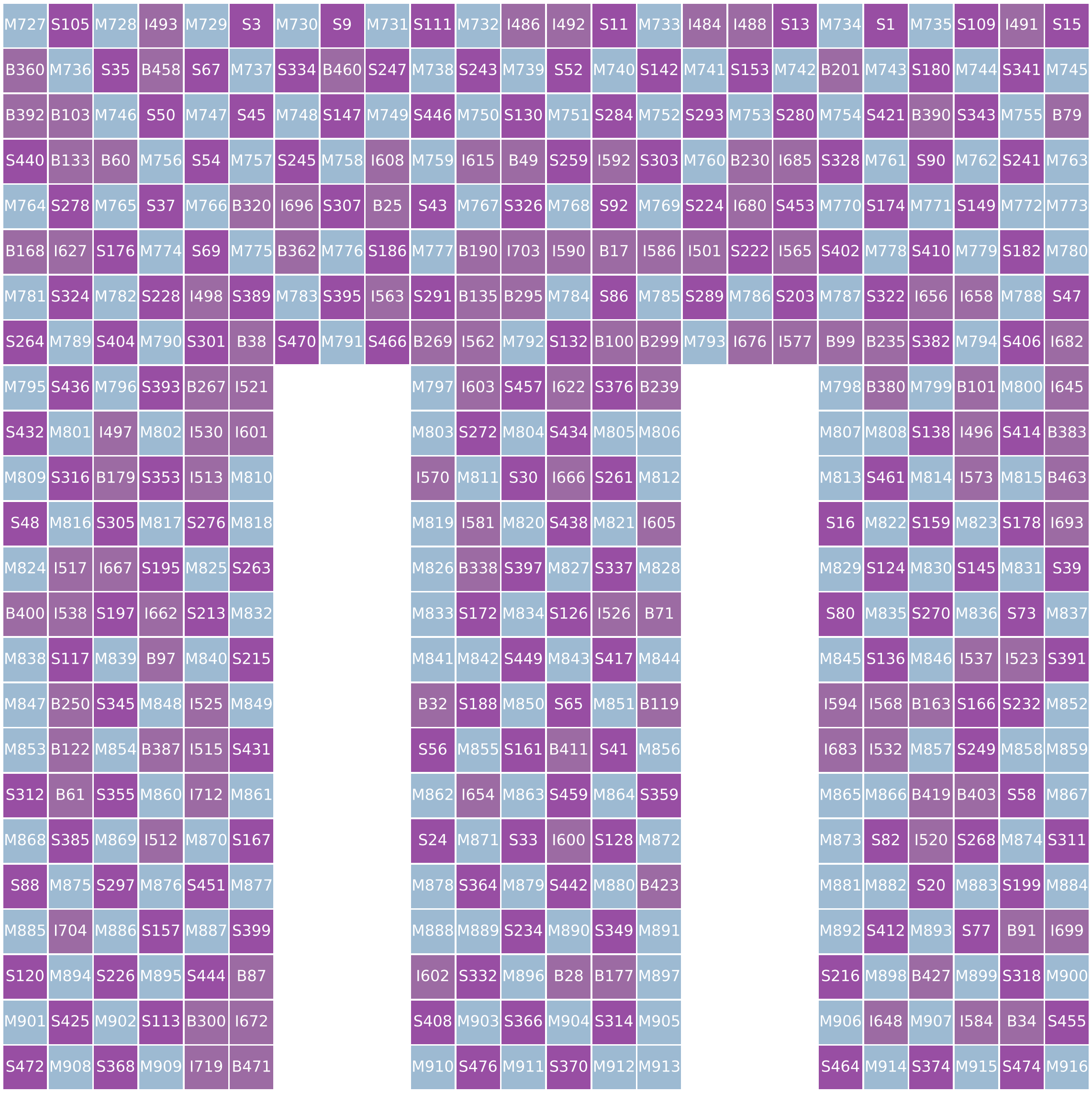

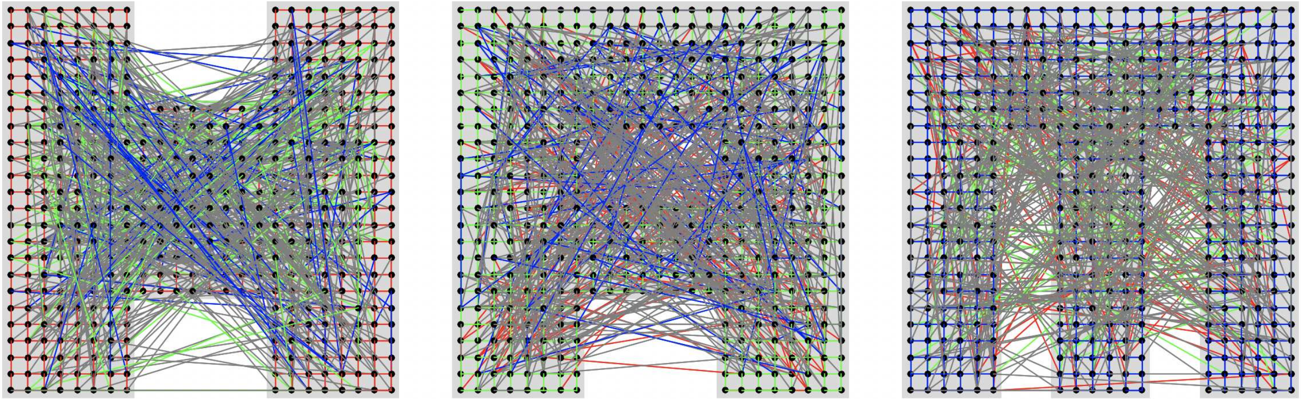

Figure S3.3: Programmed and non-programmed interactions between tiles. Tiles are shown arranged into each of the three intended shapes, with each block dot representing a tile. Each line between two tiles indicates that the tiles share complementary pair of domains on their North and South or East and West edges, and can thus interact. Red, green, and blue lines indicate the programmed interactions necessary to form the H, A, and M shapes, respectively; on the corresponding shape, these interactions are simply between adjacent tiles, while in other shapes, they link distant tiles. Gray lines indicate interactions that arise as a consequence of the transitivity of Watson-Crick binding (Figure S1.1).

3.4 Simulated structures

Stable configurations of the structures exhibited significant curvature, forming spirals of roughly 15 to 30 helices, consistent with the tile motif forming tubes of 4 to 20 helices in circumference[108] and being predicted to have relaxed curvature of approximately 30° per helix.[133] In trajectories, these spirals can change in size, for example, moving between the arrangements seen for H and for A in Figure S3.4b; it is likely that separate simulations would also result in spirals of different sizes. As each structure, from bottom-left to top-right, had 46 parallel helices, in many configurations these corners are at the center of spirals, which likely inhibits growth; numerous structures missing the corners were seen, as shown in Figures 2 and E3. Recall that the curling of the structure that takes place in the simulation, from an initial flat-but-complete configuration, does not reflect the dynamics expected in the experimental system: experimentally, the assembly would begin curling as it nucleates and grows, thus never experiencing the flat-but-complete configuration.

Structures were generated with oxDNA parameters of 1 M NaCl salt concentration, and a temperature of 20 °C (H and A) or 30 °C (M). H and M were run on oxdna.org,[93,94] while A was run on a local server using oxDNA 3.3. Relevant oxDNA files, trajectories, and movies are available at `https://www.dna.caltech.edu/SupplementaryMaterial/MultifariousSST/`.

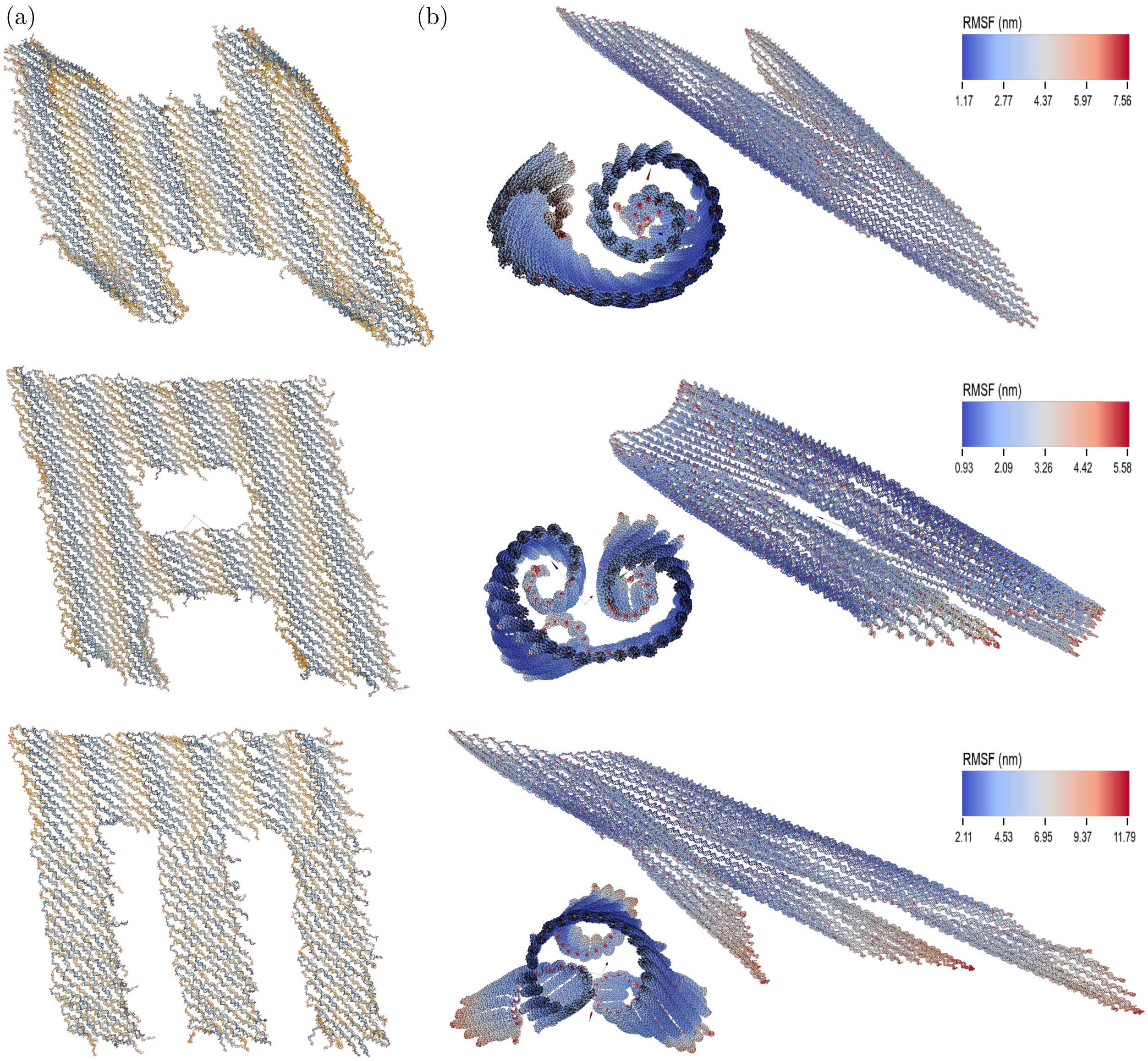

Figure S3.4: Simulated fully-assembled structures from coarse-grained molecular dynamics. **(a)** shows structures after a small number of relaxation steps. **(b)** shows mean structures, excluding configurations to reaching equilibrium, with colors corresponding to root mean square fluctuations. Two views are shown, one with DNA helix axes roughly parallelt to the plane, and one with helix axes roughly perpendicular to the plane. Images were generated with oxView.[134] We dont envision that flatter structures make any difference for nucleation, as the scale of curvature is considerably larger than that of expected critical nuclei. However, they could affect growth, changing the fluorescence readouts and assemblies visible in AFM images, for example, perhaps affecting the growth of corners of shapes. Our combination of AFM and fluorescence readouts can help investigate these issues in future work.

Bibliography

1. Hertz, J., Krogh, A. & Palmer, R. G. *Introduction to the Theory of Neural Computation* (CRC Press, 1991) (cit. on p. 7).
2. Bishop, C. M. *Pattern recognition and machine learning* (Springer, 2006) (cit. on p. 7).
3. Goodfellow, I., Bengio, Y. & Courville, A. *Deep Learning* (MIT press, 2016) (cit. on p. 7).
4. McCulloch, W. S. & Pitts, W. "A logical calculus of the ideas immanent in nervous activity". *The Bulletin of Mathematical Biophysics* **5,** 115–133 (1943) (cit. on p. 7).
5. Minsky, M. L. *Computation: Finite and Infinite Machines* (Prentice-Hall Englewood Cliffs, 1967) (cit. on p. 7).
6. Rosenblatt, F. *Principles of neurodynamics: Perceptrons and the theory of brain mechanisms* (Spartan books Washington, DC, 1962) (cit. on p. 7).
7. Mead, C. "Neuromorphic electronic systems". *Proceedings of the IEEE* **78,** 1629–1636 (1990) (cit. on p. 7).
8. Rössler, O. E. "A synthetic approach to exotic kinetics (with examples)". *Physics and Mathematics of the Nervous System,* 546–582 (1974) (cit. on p. 7).
9. Hjelmfelt, A., Weinberger, E. & Ross, J. "Chemical implementation of neural networks and Turing machines". *Proceedings of the National Academy of Sciences* **88,** 10983–10987 (1991) (cit. on p. 7).
10. Mjolsness, E., Sharp, D. H. & Reinitz, J. "A connectionist model of development". *Journal of Theoretical Biology* **152,** 429–453 (1991) (cit. on p. 7).
11. Buchler, N. E., Gerland, U. & Hwa, T. "On schemes of combinatorial transcription logic". *Proceedings of the National Academy of Sciences* **100,** 5136–5141 (2003) (cit. on p. 7).
12. Bray, D. "Protein molecules as computational elements in living cells". *Nature* **376,** 307–312 (1995) (cit. on pp. 7, 8).
13. Lewis, J. E. & Glass, L. "Steady states, limit cycles, and chaos in models of complex biological networks". *International Journal of Bifurcation and Chaos* **1,** 477–483 (1991) (cit. on p. 7).
14. Helikar, T., Konvalina, J., Heidel, J. & Rogers, J. A. "Emergent decision-making in biological signal transduction networks". *Proceedings of the National Academy of Sciences* **105,** 1913–1918 (2008) (cit. on p. 7).
15. Kim, J., Hopfield, J. J. & Winfree, E. "Neural network computation by in vitro transcriptional circuits". *Advances in Neural Information Processing Systems* **17,** 681–688 (2004) (cit. on pp. 7, 12, 23).
16. Kim, J., White, K. S. & Winfree, E. "Construction of an in vitro bistable circuit from synthetic transcriptional switches". *Molecular Systems Biology* **2,** 68 (2006) (cit. on pp. 7, 23).
17. Schaffter, S. W. *et al.* "Standardized excitable elements for scalable engineering of far-from-equilibrium chemical networks". *Nature Chemistry,* 1–9 (2022) (cit. on pp. 7, 23).
18. Montagne, K., Plasson, R., Sakai, Y., Fujii, T. & Rondelez, Y. "Programming an in vitro DNA oscillator using a molecular networking strategy". *Molecular Systems Biology* **7,** 466 (2011) (cit. on p. 7).
19. Genot, A. J., Fujii, T. & Rondelez, Y. "Computing with competition in biochemical networks". *Physical Review Letters* **109,** 208102 (2012) (cit. on p. 7).
20. Okumura, S. *et al.* "Nonlinear decision-making with enzymatic neural networks". *Nature* **610,** 496–501 (2022) (cit. on p. 7).
21. Qian, L., Winfree, E. & Bruck, J. "Neural network computation with DNA strand displacement cascades". *Nature* **475,** 368–372 (2011) (cit. on pp. 8, 12, 23).

22. Cherry, K. M. & Qian, L. "Scaling up molecular pattern recognition with DNA-based winner-take-all neural networks". *Nature* **559,** 370–376 (2018) (cit. on pp. 8, 12, 23, 49).

23. Van der Linden, A. J. *et al.* "DNA input classification by a riboregulator-based cell-free perceptron". *ACS Synthetic Biology* **11,** 1510–1520 (2022) (cit. on p. 8).

24. Pandi, A. *et al.* "Metabolic perceptrons for neural computing in biological systems". *Nature Communications* **10,** 3880 (2019) (cit. on pp. 8, 23).

25. Daniel, R., Rubens, J. R., Sarpeshkar, R. & Lu, T. K. "Synthetic analog computation in living cells". *Nature* **497,** 619–623 (2013) (cit. on pp. 8, 23).

26. Rizik, L., Danial, L., Habib, M., Weiss, R. & Daniel, R. "Synthetic neuromorphic computing in living cells". *Nature Communications* **13,** 5602 (2022) (cit. on pp. 8, 23).

27. Chen, Z., Linton, J. M., Zhu, R. & Elowitz, M. "A synthetic protein-level neural network in mammalian cells". *bioRxiv* (2022) (cit. on pp. 8, 23).

28. Schneidman, E., Berry, M. J., Segev, R. & Bialek, W. "Weak pairwise correlations imply strongly correlated network states in a neural population". *Nature* **440,** 1007–1012 (2006) (cit. on p. 8).

29. Laub, M. T. & Goulian, M. "Specificity in Two-Component Signal Transduction Pathways". *Annu. Rev. Genet.* **41,** 121–145 (Dec. 2007) (cit. on p. 8).

30. Huntley, M. H., Murugan, A. & Brenner, M. P. "Information capacity of specific interactions". en. *Proc. Natl. Acad. Sci. U. S. A.* **113,** 5841–5846 (May 2016) (cit. on p. 8).

31. Johnson, M. E. & Hummer, G. "Nonspecific binding limits the number of proteins in a cell and shapes their interaction networks". *Proceedings of the National Academy of Sciences* **108,** 603–608 (Jan. 2011) (cit. on p. 8).

32. Gao, A. *et al.* "Evolution of weak cooperative interactions for biological specificity". en. *Proc. Natl. Acad. Sci. U. S. A.* **115,** E11053–E11060 (Nov. 2018) (cit. on p. 8).

33. Zwicker, D., Murugan, A. & Brenner, M. P. "Receptor arrays optimized for natural odor statistics". *Proceedings of the National Academy of Sciences* (Jan. 2016) (cit. on p. 8).

34. Hallem, E. A. & Carlson, J. R. "Coding of odors by a receptor repertoire". *Cell* **125,** 143–160 (Jan. 2006) (cit. on p. 8).

35. Su, C. J. *et al.* "Ligand-receptor promiscuity enables cellular addressing". en. *Cell Syst* **13,** 408–425.e12 (May 2022) (cit. on p. 8).

36. Antebi, Y. E. *et al.* "Combinatorial Signal Perception in the BMP Pathway". en. *Cell* **170,** 1184–1196.e24 (Sept. 2017) (cit. on p. 8).

37. Murugan, A., Zeravcic, Z., Brenner, M. P. & Leibler, S. "Multifarious assembly mixtures: Systems allowing retrieval of diverse stored structures". *Proceedings of the National Academy of Sciences* **112,** 54–59 (2015) (cit. on pp. 9, 10, 13, 14, 19, 25).

38. Weibrecht, I. *et al.* "Proximity ligation assays: a recent addition to the proteomics toolbox". *Expert Review of Proteomics* **7,** 401–409 (2010) (cit. on p. 10).

39. Schaus, T. E., Woo, S., Xuan, F., Chen, X. & Yin, P. "A DNA nanoscope via auto-cycling proximity recording". *Nature Communications* **8,** 696 (2017) (cit. on p. 10).

40. Hochberg, G. K. A. *et al.* "A hydrophobic ratchet entrenches molecular complexes". *Nature* **588,** 503–508 (2020) (cit. on p. 10).

41. Sartori, P. & Leibler, S. "Lessons from equilibrium statistical physics regarding the assembly of protein complexes". *Proceedings of the National Academy of Sciences* **117,** 114–120 (2020) (cit. on p. 10).

42. Baum, E. "Building an associative memory vastly larger than the brain". *Science* **268,** 583–585 (1995) (cit. on p. 10).

43. Mills, A. P., Yurke, B. & Platzman, P. M. "Article for analog vector algebra computation". *Biosystems* **52,** 175–180 (1999) (cit. on p. 10).

44. Lakin, M. R. & Stefanovic, D. "Supervised learning in adaptive DNA strand displacement networks". *ACS Synthetic Biology* **5,** 885–897 (2016) (cit. on p. 10).

45. Nishimori, H., Whyte, W. & Sherrington, D. "Finite-dimensional neural networks storing structured patterns". *Physical Review E* **51,** 3628 (1995) (cit. on p. 12).

46. Koyama, S. "Storage capacity of two-dimensional neural networks". *Physical Review E* **65,** 016124 (2001) (cit. on p. 12).

47. Johnson, M. E. & Hummer, G. "Nonspecific binding limits the number of proteins in a cell and shapes their interaction networks". *Proceedings of the National Academy of Sciences* **108,** 603–608 (2011) (cit. on p. 12).

48. Whitelam, S. & Jack, R. L. "The statistical mechanics of dynamic pathways to self-assembly". *Annual Review of Physical Chemistry* **66,** 143–163 (2015) (cit. on pp. 12, 30, 36).

49. Murugan, A., Zou, J. & Brenner, M. P. "Undesired usage and the robust self-assembly of heterogeneous structures". *Nature Communications* **6,** 6203 (2015) (cit. on pp. 12, 25).

50. Jacobs, W. M. & Frenkel, D. "Self-assembly of structures with addressable complexity". *Journal of the American Chemical Society* **138,** 2457–2467 (2016) (cit. on pp. 12, 30).

51. Reinhardt, A. & Frenkel, D. "DNA brick self-assembly with an off-lattice potential". *Soft Matter* **12,** 6253–6260 (2016) (cit. on pp. 12, 30).

52. Moser, E. I., Kropff, E. & Moser, M.-B. "Place cells, grid cells, and the brain's spatial representation system". *Annual Review of Neuroscience* **31,** 69–89 (2008) (cit. on p. 14).

53. Moser, E. I., Moser, M.-B. & McNaughton, B. L. "Spatial representation in the hippocampal formation: A history". en. *Nature Neuroscience* **20,** 1448–1464 (2017) (cit. on p. 14).

54. Zhong, W., Schwab, D. J. & Murugan, A. "Associative pattern recognition through macro-molecular self-assembly". *Journal of Statistical Physics* **167,** 806–826 (cit. on pp. 14, 17).

55. Hopfield, J. J. "Neurodynamics of mental exploration". *Proceedings of the National Academy of Sciences* **107,** 1648–1653 (2010) (cit. on pp. 14, 17).

56. Burgess, N., Recce, M. & O'Keefe, J. "A model of hippocampal function". *Neural networks* **7,** 1065–1081 (1994) (cit. on p. 17).

57. Grason, G. M. "Perspective: Geometrically frustrated assemblies". *The Journal of Chemical Physics* **145,** 110901 (2016) (cit. on p. 17).

58. Berengut, J. F. *et al.* "Self-limiting polymerization of DNA origami subunits with strain accumulation". *ACS Nano* **14,** 17428–17441 (2020) (cit. on p. 17).

59. Bruck, J. "Harmonic analysis of polynomial threshold functions". *SIAM Journal on Discrete Mathematics* **3,** 168–177 (1990) (cit. on pp. 18, 19).

60. Aspnes, J., Beigel, R., Furst, M. & Rudich, S. "The expressive power of voting polynomials". *Combinatorica* **14,** 135–148 (1994) (cit. on p. 18).

61. Krause, M. & Pudlák, P. "Computing Boolean functions by polynomials and threshold circuits". *Computational Complexity* **7,** 346–370 (1998) (cit. on p. 18).

62. Hansen, K. A. & Podolskii, V. V. "Polynomial threshold functions and Boolean threshold circuits". *Information and Computation* **240,** 56–73 (2015) (cit. on p. 19).

63. Maass, W. "On the computational power of winner-take-all". *Neural Computation* **12,** 2519–2535 (2000) (cit. on p. 19).

64. Baldi, P. & Vershynin, R. "Polynomial threshold functions, hyperplane arrangements, and random tensors". *SIAM Journal on Mathematics of Data Science* **1,** 699–729 (2019) (cit. on p. 19).

65. LeCun, Y., Bottou, L., Bengio, Y. & Haffner, P. "Gradient-based learning applied to document recognition". *Proceedings of the IEEE* **86,** 2278–2324 (1998) (cit. on p. 19).

66. Koch, C. & Poggio, T. "Multiplying with Synapses and Neurons". *Single Neuron Computation,* 315–345 (1992) (cit. on p. 21).

67. Mel, B. W. "Information processing in dendritic trees". *Neural Computation* **6,** 1031–1085 (1994) (cit. on p. 21).

68. Dayan, P. & Abbott, L. F. *Theoretical Neuroscience: Computational and Mathematical Modeling of Neural Systems* (MIT press, 2005) (cit. on p. 21).

69. Tanaka, G. *et al.* "Recent advances in physical reservoir computing: A review". *Neural Networks* **115,** 100–123 (2019) (cit. on p. 21).

70. Seelig, G., Soloveichik, D., Zhang, D. Y. & Winfree, E. "Enzyme-free nucleic acid logic circuits". *science* **314,** 1585–1588 (2006) (cit. on p. 21).

71. Wang, B., Thachuk, C., Ellington, A. D., Winfree, E. & Soloveichik, D. "Effective design principles for leakless strand displacement systems". *Proceedings of the National Academy of Sciences* **115,** E12182–E12191 (2018) (cit. on pp. 21, 24).

72. Good, M. C., Zalatan, J. G. & Lim, W. A. "Scaffold proteins: hubs for controlling the flow of cellular information". *Science* **332,** 680–686 (2011) (cit. on p. 21).

73. Jaeger, H. "The "echo state" approach to analysing and training recurrent neural networks". *Bonn, Germany: German National Research Center for Information Technology GMD Technical Report* **148,** 13 (2001) (cit. on p. 22).

74. Maass, W., Natschläger, T. & Markram, H. "Real-time computing without stable states: A new framework for neural computation based on perturbations". *Neural Computation* **14,** 2531–2560 (2002) (cit. on p. 22).

75. Goudarzi, A., Lakin, M. R. & Stefanovic, D. *DNA reservoir computing: a novel molecular computing approach*. in *DNA Computing and Molecular Programming (LNCS 8141* (2013), 76–89 (cit. on p. 22).

76. Ong, L. L. *et al.* "Programmable self-assembly of three-dimensional nanostructures from 10,000 unique components". *Nature* **552,** 72–77 (2017) (cit. on p. 23).

77. Xiong, X. *et al.* "Molecular convolutional neural networks with DNA regulatory circuits". *Nature Machine Intelligence* **4,** 625–635 (2022) (cit. on p. 23).

78. Woods, D. *et al.* "Diverse and robust molecular algorithms using reprogrammable DNA self-assembly". *Nature* **567,** 366 (Mar. 2019) (cit. on pp. 23, 24, 29).

79. Wang, F. *et al.* "Implementing digital computing with DNA-based switching circuits". *Nature Communications* **11,** 121 (2020) (cit. on p. 24).

80. Roostalu, J. & Surrey, T. "Microtubule nucleation: beyond the template". *Nature Reviews Molecular Cell Biology* **18,** 702–710 (2017) (cit. on p. 24).

81. Rothemund, P. W. K. & Winfree, E. *The program-size complexity of self-assembled squares*. in *Proceedings of the thirty-second annual ACM Symposium on Theory of Computing (STOC)* (2000), 459–468 (cit. on p. 24).

82. Rothemund, P. W. K., Papadakis, N. & Winfree, E. "Algorithmic self-assembly of DNA Sierpinski triangles". *PLoS Biology* **2,** e424 (2004) (cit. on pp. 24, 27, 28).

83. Fujibayashi, K., Hariadi, R., Park, S. H., Winfree, E. & Murata, S. "Toward reliable algorithmic self-assembly of DNA tiles: A fixed-width cellular automaton pattern". *Nano Letters* **8,** 1791–1797 (2008) (cit. on pp. 24, 27).

84. Barish, R. D., Schulman, R., Rothemund, P. W. K. & Winfree, E. "An information-bearing seed for nucleating algorithmic self-assembly". *Proceedings of the National Academy of Sciences* **106,** 6054–6059 (2009) (cit. on p. 24).

85. Mitscherlich, E. "Sur la relation qui existe entre la forme cristalline et les proportions chimiques. I. Mémoire sur les arsenates et les phosphates". *Annales de Chimie et de Physique* **19,** 350–419. `https://gallica.bnf.fr/ark:/12148/bpt6k6571078m/f356.item` (1821) (cit. on p. 25).

86. Hodgkin, D. C. "The X-ray analysis of complicated molecules". *Science* **150,** 979–988. `http://www.jstor.org/stable/1717892` (1965) (cit. on p. 25).

87. Jacobs, W. M., Reinhardt, A. & Frenkel, D. "Rational design of self-assembly pathways for complex multicomponent structures". *Proceedings of the National Academy of Sciences* **112,** 6313–6318 (2015) (cit. on pp. 25, 30).

88. Dunn, K. E. *et al.* "Guiding the folding pathway of DNA origami". *Nature* **525,** 82–86 (2015) (cit. on p. 25).

89. Pan, T. & Sosnick, T. "RNA folding during transcription". *Annual Review of Biophysics and Biomolecular Structure* **35,** 161–175 (2006) (cit. on p. 25).

90. Dirks, R. M. & Pierce, N. A. "Triggered amplification by hybridization chain reaction". *Proceedings of the National Academy of Sciences* **101,** 15275–15278 (2004) (cit. on p. 25).

91. Yin, P., Choi, H. M., Calvert, C. R. & Pierce, N. A. "Programming biomolecular self-assembly pathways". *Nature* **451,** 318–322 (2008) (cit. on p. 25).

92. Maffeo, C., Yoo, J. & Aksimentiev, A. "De novo reconstruction of DNA origami structures through atomistic molecular dynamics simulation". *Nucleic Acids Research* **44,** 3013–3019 (2016) (cit. on p. 26).

93. Sengar, A., Ouldridge, T. E., Henrich, O., Rovigatti, L. & Šulc, P. "A primer on the oxDNA model of DNA: When to use it, how to simulate it and how to interpret the results". *Frontiers in Molecular Biosciences* **8** (2021) (cit. on pp. 26, 57).

94. Poppleton, E., Romero, R., Mallya, A., Rovigatti, L. & Šulc, P. "OxDNA.Org: A public webserver for coarse-grained simulations of DNA and RNA nanostructures". *Nucleic Acids Research* **49,** W491–W498 (cit. on pp. 26, 57).

95. Fonseca, P. *et al.* "Multi-scale coarse-graining for the study of assembly pathways in DNA-brick self-assembly". *The Journal of Chemical Physics* **148,** 134910 (cit. on pp. 26, 28, 29).

96. Winfree, E. *Simulations of computing by self-assembly.* Tech. rep. CaltechCSTR:1998.22 (Pasadena, CA, 1998) (cit. on p. 26).

97. Evans, C. G., Hariadi, R. F. & Winfree, E. "Direct atomic force microscopy observation of DNA tile crystal growth at the single-molecule level". *Journal of the American Chemical Society* **134,** 10485–10492 (2012) (cit. on pp. 26, 28).

98. Jiang, S., Hong, F., Hu, H., Yan, H. & Liu, Y. "Understanding the elementary Steps in DNA tile-based self-assembly". *ACS Nano* **11,** 9370–9381 (2017) (cit. on pp. 26, 28, 29, 49).

99. Schulman, R. & Winfree, E. "Programmable control of nucleation for algorithmic self-assembly". *SIAM Journal on Computing* **39,** 1581–1616 (2009) (cit. on pp. 26, 30, 31).

100. Evans, C. G. & Winfree, E. "Physical principles for DNA tile self-assembly". *Chemical Society Reviews* **46,** 3808–3829 (2017) (cit. on pp. 26, 28, 31).

101. Gillespie, D. T. "Stochastic simulation of chemical kinetics". *Annual Review of Physical Chemistry* **58,** 35–55 (2007) (cit. on p. 28).

102. Gibson, M. A. & Bruck, J. "Efficient exact stochastic simulation of chemical systems with many species and many channels". *The Journal of Physical Chemistry A* **104,** 1876–1889 (2000) (cit. on p. 28).

103. Evans, C. G., Schulman, R. & Winfree, E. *The Xgrow Simulator.* 1996–2022. `https://github.com/DNA-and-Natural-Algorithms-Group/xgrow` (cit. on p. 28).

104. Schulman, R., Wright, C. & Winfree, E. "Increasing redundancy exponentially reduces error rates during algorithmic self-assembly". *ACS Nano* **9,** 5760–5771 (2015) (cit. on p. 28).

105. Fujibayashi, K. & Murata, S. "Precise simulation model for DNA tile self-assembly". *IEEE Transactions on Nanotechnology* **8,** 361–368 (2009) (cit. on p. 28).

106. Rothemund, P. W. K. *et al.* "Design and characterization of programmable DNA nanotubes". *Journal of the American Chemical Society* **126,** 16344–16352 (2004) (cit. on p. 28).

107. Schulman, R. & Winfree, E. "Synthesis of crystals with a programmable kinetic barrier to nucleation". *Proceedings of the National Academy of Sciences* **104,** 15236–15241 (2007) (cit. on pp. 28, 30).

108. Yin, P. *et al.* "Programming DNA tube circumferences". *Science* **321,** 824–826 (Aug. 8, 2008) (cit. on pp. 28, 57).

109. Winfree, E., Liu, F., Wenzler, L. A. & Seeman, N. C. "Design and self-assembly of two-dimensional DNA crystals". *Nature* **394,** 539–544 (1998) (cit. on p. 28).

110. Wei, B., Dai, M. & Yin, P. "Complex shapes self-assembled from single-stranded DNA tiles". *Nature* **485,** 623–626 (May 2012) (cit. on p. 28).

111. Wang, W. *et al.* "Self-assembly of fully addressable DNA nanostructures from double crossover tiles". *Nucleic acids research* **44,** 7989–7996 (2016) (cit. on pp. 28, 49).

112. Zheng, J. *et al.* "From molecular to macroscopic via the rational design of a self-assembled 3D DNA crystal". *Nature* **461,** 74–77 (2009) (cit. on p. 28).

113. Ke, Y., Ong, L. L., Shih, W. M. & Yin, P. "Three-Dimensional Structures Self-Assembled from DNA Bricks". *Science* **338,** 1177–1183 (2012) (cit. on p. 28).

114. Ke, Y. *et al.* "DNA brick crystals with prescribed depths". *Nature Chemistry* **6,** 994–1002 (2014) (cit. on pp. 28, 49).

115. Fu, T. J. & Seeman, N. C. "DNA double-crossover molecules". *Biochemistry* **32,** 3211–3220 (1993) (cit. on p. 28).

116. Doye, J. P. *et al.* "Coarse-graining DNA for simulations of DNA nanotechnology". *Physical Chemistry Chemical Physics* **15,** 20395–20414 (2013) (cit. on p. 29).

117. SantaLucia Jr, J. & Hicks, D. "The thermodynamics of DNA structural motifs". *Annual Review of Biophysics and Biomolecular Structure* **33,** 415–440 (2004) (cit. on p. 29).

118. Evans, C. G. *stickydesign v0.8.2.* 2019. `https://github.com/DNA-and-Natural-Algorithms-Group/stickydesign` (cit. on p. 29).

119. Frenkel, D. & Smit, B. *Understanding Molecular Simulation: From Algorithms to Applications* (2002) (cit. on p. 30).

120. Reinhardt, A. & Frenkel, D. "Numerical evidence for nucleated self-assembly of DNA brick structures". *Physical review letters* **112,** 238103 (2014) (cit. on p. 30).

121. Sajfutdinow, M., Jacobs, W. M., Reinhardt, A., Schneider, C. & Smith, D. M. "Direct observation and rational design of nucleation behavior in addressable self-assembly". *Proceedings of the National Academy of Sciences* **115,** E5877–E5886 (2018) (cit. on pp. 30, 49).

122. Minev, D., Wintersinger, C. M., Ershova, A. & Shih, W. M. "Robust nucleation control via crisscross polymerization of highly coordinated DNA slats". *Nature Communications* **12,** 1–9 (2021) (cit. on p. 30).

123. Wintersinger, C. M. *et al.* "Multi-micron crisscross structures grown from DNA-origami slats". *Nature Nanotechnology* **18,** 281–289 (2023) (cit. on p. 30).

124. Bolhuis, P. G., Chandler, D., Dellago, C. & Geissler, P. L. "Transition path sampling: Throwing ropes over rough mountain passes, in the dark". *Annual Review of Physical Chemistry* **53.** PMID: 11972010, 291–318 (2002) (cit. on p. 32).

125. Allen, R. J., Valeriani, C. & ten Wolde, P. R. "Forward flux sampling for rare event simulations". *Journal of Physics: Condensed Matter* **21,** 463102 (2009) (cit. on p. 32).

126. Pan, A. C. & Chandler, D. "Dynamics of nucleation in the Ising model". *The Journal of Physical Chemistry B* **108,** 19681–19686 (2004) (cit. on p. 32).

127. Oxtoby, D. W. "Homogeneous nucleation: Theory and experiment". *Journal of Physics: Condensed Matter* **4,** 7627 (1992) (cit. on p. 36).

128. Soloveichik, D. *CRN Simulator (version 2.1).* 2022. `http://users.ece.utexas.edu/~soloveichik/crnsimulator.html` (cit. on p. 41).

129. You, Y., Tataurov, A. V. & Owczarzy, R. "Measuring thermodynamic details of DNA hybridization using fluorescence". *Biopolymers* **95,** 472–486 (2011) (cit. on pp. 49, 53).

130. Padirac, A., Fujii, T. & Rondelez, Y. "Quencher-free multiplexed monitoring of DNA reaction circuits". *Nucleic Acids Research* **40,** e118–e118 (2012) (cit. on p. 49).

131. Huang, Q. *et al.* "Multiplex fluorescence melting curve analysis for mutation detection with dual-labeled, self-quenched probes". *PloS ONE* **6,** e19206 (2011) (cit. on pp. 49, 50).

132. Zadeh, J. N. *et al.* "NUPACK: Analysis and design of nucleic acid systems". *Journal of Computational Chemistry* **32,** 170–173 (2011) (cit. on p. 50).

133. Wei, B. *et al.* "Design space for complex DNA structures". *Journal of the American Chemical Society* **135,** 18080–18088 (2013) (cit. on pp. 53, 57).

134. Bohlin, J. *et al.* "Design and simulation of DNA, RNA and hybrid protein-nucleic acid nanostructures with oxView". *Nature Protocols* **17,** 1762–1788 (8 Aug. 2022) (cit. on p. 58).

Section 4

Sequences

4.1 Unmodified strands

Tile names in this table are of the form [LETTER][TILE NUMBER], where the tile number starts from 0 (and does not depend on the letter), and the letter denotes what shapes the tile appears in. S tiles appear in all three shapes, H/A/M tiles appear only in the corresponding shape, and I/B/N tiles appear in two shapes: A+M, H+M, and H+A, respectively, akin to the IUPAC naming for degenerate DNA bases, where the letter is the letter that follows the excluded shape in the alphabet. Spaces in sequences correspond to divisions between glues. Glues are listed from 5' to 3'. They do not indicate complementarity: as we do not consider tile rotations, a glue in position 1 will bind to 3, and position 2 to position 4. Glues with the same names in these two positions (1/3 or 2/4) will be reverse complements of each other: for example, glue 3 of H1 is the reverse complement of glue 1 of H0. The exception to this is for positions that are "null" glues, which are simply 10nt or 11nt all-T regions, on tiles that will form the edges of shapes. On diagrams where the H/A/M shapes are upright, such as in Section 3.3, the glue positions in these sequences will correspond with East, North, West, and South directions, respectively. Null glue names refer to the length in nucleotides (as tiles alternate between having 10/11/11/10 nt domains and 11/10/10/11 nt domains), and, for consistency reasons, a rotated cardinal direction.

Name	Sequence ($5' \rightarrow 3'$)	Gl. 1/E	Gl. 2/N	Gl. 3/W	Gl. 4/S
H0	TGGGAATATCT TTTTTTTTTT TTTTTTTTTT TTCATGTTGTT	e1	nullT10W	nullT10N	e2
S1	AACTTCTCGA TTTTTTTTTTT AGATATTCCCA ATCACCCAAA	e3	nullT11W	e1	e4
N2	AGGTTAAGAGA TTTTTTTTTT TCGAGAAGTT TAAAAGGCAAA	e5	nullT10W	e3	e6
S3	TCACATACGT TTTTTTTTTTT TCTCTTAACCT AATCGATCCA	e7	nullT11W	e5	e8
H4	TTAGTATCGGT TTTTTTTTTT ACGTATGTGA TACATGATGGA	e9	nullT10W	e7	e10
N5	ACTGCTTCTT TTTTTTTTTTT ACCGATACTAA AATCGTTCCT	e11	nullT11W	e9	e12
H6	TTGTTCATTGT TTTTTTTTTT AAGAAGCAGT TGGTGAGATTA	e13	nullT10W	e11	e14
H7	TTTTTTTTTT TTTTTTTTTTT ACAATGAACAA TTCTCGACAT	nullT10S	nullT11W	e13	e15
H8	TACGGTATTGA TTTTTTTTTT TTTTTTTTTT AGGAGTTTACT	e16	nullT10W	nullT10N	e17
S9	TAGCATCACA TTTTTTTTTTT TCAATACCGTA ACTAGCACAA	e18	nullT11W	e16	e19
N10	AAGATACGAGA TTTTTTTTTT TGTGATGCTA TTTCATAGGGA	e20	nullT10W	e18	e21
S11	TTTCGCTCTA TTTTTTTTTTT TCTCGTATCTT AAAGAACCCA	e22	nullT11W	e20	e23
H12	AAGATACGAGA TTTTTTTTTT TAGAGCGAAA TGTGATAGACA	e20	nullT10W	e22	e25
S13	AACGTTACCT TTTTTTTTTTT TCTCGTATCTT AGCCTTTCTT	e26	nullT11W	e20	e27
H14	TTTCGGATAGA TTTTTTTTTT AGGTAACGTT TTAGTAGGTGT	e28	nullT10W	e26	e29
S15	TTTTTTTTTT TTTTTTTTTTT TCTATCCGAAA ACTCAACACA	nullT10S	nullT11W	e28	e30
S16	TTACACCGAT AACAACATGAA TTTTTTTTTTT TCACCAAACT	e31	e2	nullT11N	e32
B17	TGCGATATTTT TTTGGGTGAT ATCGGTGTAA AGCTAGTGTTA	e33	e4	e31	e34
N18	TTTTCCCTGA TTTGCCTTTTA AAAATATCGCA TATCAACGCT	e35	e6	e33	e36
H19	AAGGGATTAGT TGGATCGATT TCAGGGAAAA ATTGTTTGACA	e37	e8	e35	e38
S20	AAACTCCGAA TCCATCATGTA ACTAATCCCTT TTGTACCACA	e39	e10	e37	e40
N21	TGGATGATACT AGGAACGATT TTCGGAGTTT TCAAGAAGGTA	e41	e12	e39	e42
N22	TCTTTCACGT TAATCTCACCA AGTATCATCCA AAAACGCAAT	e43	e14	e41	e44
H23	TTTTTTTTTTT ATGTCGAGAA ACGTGAAAGA TGAGGTATGAT	nullT11S	e15	e43	e45
S24	ACAGCCATTA AGTAAACTCCT TTTTTTTTTTT TTCAACTCGT	e46	e17	nullT11N	e47

**Name**	**Sequence** ($5' \to 3'$)	**Gl. 1/E**	**Gl. 2/N**	**Gl. 3/W**	**Gl. 4/S**
B25	TGCAGTTTTTA TTGTGCTAGT TAATGGCTGT ATTAGTGGGTA	e48	e19	e46	e49
N26	TTCATCGCTA TCCCTATGAAA TAAAAACTGCA AGTCAAACCT	e50	e21	e48	e51
H27	TGGCAAAATAA TGGGTTCTTT TAGCGATGAA AGTATTACGGA	e52	e23	e50	e53
B28	ACTTGTTCCT TGTCTATCACA TTATTTTGCCA ACCACTTCTT	e54	e25	e52	e55
H29	AGTAGTGAAGT AAGAAAGGCT AGGAACAAGT ATAGACAGGAA	e56	e27	e54	e57
S30	TTCTACGCAT ACACCTACTAA ACTTCACTACT ATCACCACAA	e58	e29	e56	e59
H31	TTTTTTTTTTT TGTGTTGAGT ATGCGTAGAA TAGGAAGACAA	nullT11S	e30	e58	e60
B32	TTGAGGGATAT AGTTTGGTGA TTTTTTTTTT TCGAGAGATAA	e61	e32	nullT10N	e62
S33	TTCGTACACA TAACACTAGCT ATATCCCTCAA TGCTCATCAT	e63	e34	e61	e64
B34	TGGCTATTAGA AGCGTTGATA TGTGTACGAA ACGGAAATTTT	e65	e36	e63	e66
S35	ACCTCACATT TGTCAAACAAT TCTAATAGCCA ACCCATTTCA	e67	e38	e65	e68
H36	ATGGGATTAGA TGTGGTACAA AATGTGAGGT TTTTTAAGCGT	e69	e40	e67	e70
S37	ACCCTTCAAT TACCTTCTTGA TCTAATCCCAT AATCACCAGT	e71	e42	e69	e72
B38	AGTAGACTGAA ATTGCGTTTT ATTGAAGGGT TAATACGAGGA	e73	e44	e71	e74
S39	TTTTTTTTTT ATCATACCTCA TTCAGTCTACT TATGCACTCA	nullT10S	e45	e73	e75
H40	TGAGGACTATT ACGAGTTGAA TTTTTTTTTT TCGAGAGATAA	e76	e47	nullT10N	e62
S41	TGCATATCCA TACCCACTAAT AATAGTCCTCA TTAGCAACCA	e78	e49	e76	e79
H42	TGCAGTTTTTA AGGTTTGACT TGGATATGCA AAAATTAGCGT	e48	e51	e78	e81
S43	TTGACACCAT TCCGTAATACT TAAAAACTGCA TTACCCAACA	e82	e53	e48	e83
H44	AAGTTAGGGTA AAGAAGTGGT ATGGTGTCAA TGGTATTCAGA	e84	e55	e82	e85
S45	ACCCTTCTTT TTCCTGTCTAT TACCCTAACTT ATTGCCACTA	e86	e57	e84	e87
N46	AGGGATGTAAT TTGTGGTGAT AAAGAAGGGT ATATGGTAGCT	e88	e59	e86	e89
S47	TTTTTTTTTT TTGTCTTCCTA ATTACATCCCT ATTCACCGTA	nullT10S	e60	e88	e90
S48	ATGTCCACTT TTATCTCTCGA TTTTTTTTTTT TCGATCCAAT	e91	e62	nullT11N	e92
B49	AATAGGGTACA ATGATGAGCA AAGTGGACAT AGGATATAGCA	e93	e64	e91	e94
S50	ATTGACCCTT AAAATTTCCGT TGTACCCTATT ATTACAGCCA	e95	e66	e93	e96
H51	TGGTAGTAACT TGAAATGGGT AAGGGTCAAT ATAGAGTTGGA	e97	e68	e95	e98
S52	AACTTCAGCT ACGCTTAAAAA AGTTACTACCA ATCACGCTTA	e99	e70	e97	e100
H53	TGGAAGAGTAT ACTGGTGATT AGCTGAAGTT AAATTGGAACA	e101	e72	e99	e102
S54	AAACCAGTCT TCCTCGTATTA ATACTCTTCCA ATTACTGCCA	e103	e74	e101	e104
H55	TTTTTTTTTTT TGAGTGCATA AGACTGGTTT ATTTTTGACGT	nullT11S	e75	e103	e105
S56	TTACCGACAT TTATCTCTCGA TTTTTTTTTTT ATCCATCGAA	e106	e62	nullT11N	e107
H57	AGAATTAGGGA TGGTTGCTAA ATGTCGGTAA TAGAGAATCGA	e108	e79	e106	e109
S58	ACTGAACCTT ACGCTAATTTT TCCCTAATTCT ACTCGTAACA	e110	e81	e108	e111
H59	ATCTTGGAGTA TGTTGGGTAA AAGGTTCAGT TTTTGGTTCAA	e112	e83	e110	e113
B60	AGCCTACAAA TCTGAATACCA TACTCCAAGAT AATCCTTGCT	e114	e85	e112	e115
B61	ACAAGGATAGA TAGTGGCAAT TTTGTAGGCT TAGGGTAACAT	e116	e87	e114	e117
N62	AAGTCACCAA AGCTACCATAT TCTATCCTTGT TTCTTCACGT	e118	e89	e116	e119
H63	TTTTTTTTTTT TACGGTGAAT TTGGTGACTT ATGCAAAGATT	nullT11S	e90	e118	e120
H64	AAGTACTGAGA ATTGGATCGA TTTTTTTTTT AGTAGGAAGAA	e121	e92	nullT10N	e122
S65	ACTGCCATAA TGCTATATCCT TCTCAGTACTT ATGCATACCA	e123	e94	e121	e124
H66	TTATCTGTGGA TGGCTGTAAT TTATGGCAGT AGACTTAGGAA	e125	e96	e123	e126
S67	TACGTTCCAA TCCAACTCTAT TCCACAGATAA ACATCTGCTT	e127	e98	e125	e128
H68	ATACTGGATGA TAAGCGTGAT TTGGAACGTA AATAGTCTGGT	e129	e100	e127	e130
S69	AACCTCGAAA TGTTCCAATTT TCATCCAGTAT AGCCTTTCTT	e131	e102	e129	e27
H70	TGTTTGAACTT TGGCAGTAAT TTTCGAGGTT AGCTAGTGTTA	e133	e104	e131	e34
B71	TTTTTTTTTT ACGTCAAAAAT AAGTTCAAACA ATCTTCTGCA	nullT10S	e105	e133	e135
H72	AGAACTAAGGA TTCGATGGAT TTTTTTTTTT AACTATGAGGA	e136	e107	nullT10N	e137
S73	TAGCCAACAA TCGATTCTCTA TCCTTAGTTCT TCGACTTCTT	e138	e109	e136	e139
H74	ACGAGATAGAA TGTTACGAGT TTGTTGGCTA AATGTGGAAAA	e140	e111	e138	e141
N75	AACGTCTTCT TTGAACCAAAA TTCTATCTCGT TCCAATCCAA	e142	e113	e140	e143
H76	AGTGATTAGGA AGCAAGGATT AGAAGACGTT AGGTTCTAGTT	e144	e115	e142	e145
S77	AGCTTCATCA ATGTTACCCTA TCCTAATCACT ATGCACTACT	e146	e117	e144	e147
H78	TGGACAAATTT ACGTGAAGAA TGATGAAGCT ATGGGATCATA	e148	e119	e146	e149
B79	TTTTTTTTTT AATCTTTGCAT AAATTTGTCCA TAACACCCAA	nullT10S	e120	e148	e150
S80	ACATCCATGA TTCTTCCTACT TTTTTTTTTTT ATTCACCCTT	e151	e122	nullT11N	e152
H81	TAGAGGAAAGT TGGTATGCAT TCATGGATGT AGAACTATGGT	e153	e124	e151	e154
S82	AACCCTCTTT TTCCTAAGTCT ACTTTCCTCTA ACTAACGACA	e155	e126	e153	e156
H83	ACAGTAGGATT AAGCAGATGT AAAGAGGGTT ATTATCAGGGT	e157	e128	e155	e158

Name	Sequence (5′ → 3′)	Gl. 1/E	Gl. 2/N	Gl. 3/W	Gl. 4/S
N84	AATCACAGCT ACCAGACTATT AATCCTACTGT TCCGATCAAA	e159	e130	e157	e160
N85	AGGGTTCTAAT AAGAAAGGCT AGCTGTGATT AATAGTCTGGT	e161	e27	e159	e130
S86	TTCGCATCTA TAACACTAGCT ATTAGAACCCT TCCGATCAAA	e163	e34	e161	e160
B87	TTTTTTTTTTT TGCAGAAGAT TAGATGCGAA AGTGAACAAAA	nullT11S	e135	e163	e165
S88	ACACAGAACA TCCTCATAGTT TTTTTTTTTTT TCATAGCCAA	e166	e137	nullT11N	e167
H89	AGAGTTTAGGA AAGAAGTCGA TGTTCTGTGT AGATCAGTGTA	e168	e139	e166	e169
S90	AGCTTCATCA TTTTCCACATT TCCTAAACTCT TAGCAAACCA	e146	e141	e168	e171
B91	TGGAGATACAT TTGGATTGGA TGATGAAGCT TCTAAGTGTGA	e172	e143	e146	e173
S92	AAACCCAGAT AACTAGAACCT ATGTATCTCCA ATCACCCAAA	e174	e145	e172	e4
H93	TATCGGAAGTA AGTAGTGCAT ATCTGGGTTT AGCAAAAGAAA	e176	e147	e174	e177
N94	TGCTCCATTA TATGATCCCAT TACTTCCGATA AAACCTCCTT	e178	e149	e176	e179
N95	TTTTTTTTTTT TTGGGTGTTA TAATGGAGCA ACTTTAGAGGT	nullT11S	e150	e178	e180
H96	AGCTTAGATGA AAGGGTGAAT TTTTTTTTTT AGAGCTATGAT	e181	e152	nullT10N	e182
B97	TCTTGCTTCT ACCATAGTTCT TCATCTAAGCT TTCTGACCAA	e183	e154	e181	e184
H98	TTAGGATGACA TGTCGTTAGT AGAAGCAAGA TGGAGTAAGAT	e185	e156	e183	e186
B99	TTCCCAAAGA ACCCTGATAAT TGTCATCCTAA ATCGAACCTT	e187	e158	e185	e188
B100	AGTTGATAGGT TTTGATCGGA TCTTTGGGAA TTTAAATGCGA	e189	e160	e187	e190
B101	TTTACACCGA ACCAGACTATT ACCTATCAACT TTCGACACTT	e191	e130	e189	e192
H102	TATATGGTCGT TTTGATCGGA TCGGTGTAAA AGAGTTAGGAA	e193	e160	e191	e194
B103	TGACAACCAT TTTTGTTCACT ACGACCATATA TCATTGCCTA	e195	e165	e193	e196
H104	AGATGTGATCT TTTTTTTTTT ATGGTTGTCA ATGATTGCAAT	e197	nullT10W	e195	e198
S105	ACCTCACATT TTTTTTTTTTT AGATCACATCT AACTCCGATT	e67	nullT11W	e197	e200
H106	AGGTTAAGAGA TTTTTTTTTT AATGTGAGGT AATTTTTCGGT	e5	nullT10W	e67	e202
N107	ACCAGCAATA TTTTTTTTTTT TCTCTTAACCT ACACCATCAT	e203	nullT11W	e5	e204
H108	TTTCGGATAGA TTTTTTTTTT TATTGCTGGT AAAAGTTGTGT	e28	nullT10W	e203	e206
S109	TTTCCCTTGA TTTTTTTTTTT TCTATCCGAAA TCATTGCCTA	e207	nullT11W	e28	e196
H110	AGTTTGTTTGT TTTTTTTTTT TCAAGGGAAA TATATGACGGA	e209	nullT10W	e207	e210
S111	AAACTTCCCA TTTTTTTTTTT ACAAACAAACT ATCACCACAA	e211	nullT11W	e209	e59
H112	TTCTATGTGGA TTGGCTATGA TGGGAAGTTT AGATTATCGGA	e213	e167	e211	e214
S113	ATCCACCATT TACACTGATCT TCCACATAGAA TCATTGCTCT	e215	e169	e213	e216
H114	TCGTTTAGAGA TGGTTTGCTA AATGGTGGAT TCTTAGAAGGT	e217	e171	e215	e218
N115	AACCCTCTTT TCACACTTAGA TCTCTAAACGA ACTGTCACAT	e155	e173	e217	e220
N116	AGAACTAAGGA TTTGGGTGAT AAAGAGGGTT AAAGAGAGGAA	e136	e4	e155	e222
S117	ATATGCACCA TTTCTTTTGCT TCCTTAGTTCT ATGACCCAAT	e223	e177	e136	e224
H118	AAGTCGAGTAT AAGGAGGTTT TGGTGCATAT AGAGTAGTGTT	e225	e179	e223	e226
B119	TTTTTTTTTT ACCTCTAAAGT ATACTCGACTT TGAACCAACT	nullT10S	e180	e225	e227
S120	ACTGAACCTT ATCATAGCTCT TTTTTTTTTTT TCTACCTCCT	e110	e182	nullT11N	e229
N121	TGGAGATACAT TTGGTCAGAA AAGGTTCAGT TAAGAAGGTCA	e172	e184	e110	e231
B122	ACTCGCAATA ATCTTACTCCA ATGTATCTCCA ATTGCCACTA	e232	e186	e172	e87
H123	AGAACAGAGTA AAGGTTCGAT TATTGCGAGT TCTGGGATTAT	e234	e188	e232	e235
S124	TTGACATCCA TCGCATTTAAA TACTCTGTTCT ACTTGCCTAA	e236	e190	e234	e237
H125	TTGAGAGTAGT AAGTGTCGAA TGGATGTCAA ACAATGGAAAA	e238	e192	e236	e239
S126	TCGATTCCTT TTCCTAACTCT ACTACTCTCAA ACTGCTACAT	e240	e194	e238	e241
H127	TGGATGATACT TAGGCAATGA AAGGAATCGA TTCGGTATAGA	e41	e196	e240	e243
S128	ACGTCCATAA ATTGCAATCAT AGTATCATCCA ATTGCCACTA	e244	e198	e41	e87
H129	TGTTGTTGATT AATCGGAGTT TTATGGACGT AGAGTTAGGAA	e246	e200	e244	e194
S130	AATCCGAACT ACCGAAAAATT AATCAACAACA TGCTCATCAT	e248	e202	e246	e64
N131	ACTGAGGATAA ATGATGGTGT AGTTCGGATT TAATAGTCGGA	e250	e204	e248	e251
S132	TTCCCAAAGA ACACAACTTTT TTATCCTCAGT TCAAGAACCA	e187	e206	e250	e253
B133	ATCTTGGAGTA TAGGCAATGA TCTTTGGGAA TATAGACGTGT	e112	e196	e187	e255
N134	ACCTGACATT TCCGTCATATA TACTCCAAGAT AGAAACACCT	e256	e210	e112	e257
B135	AACGTGAAATT TTGTGGTGAT AATGTCAGGT ATGTAGTAGCA	e258	e59	e256	e259
S136	AATCAGCCTT TCCGATAATCT AATTTCACGTT ACTTGCCTAA	e260	e214	e258	e237
H137	TAGTCAGTTGA AGAGCAATGA AAGGCTGATT TTAGTTCAGGA	e262	e216	e260	e263
S138	ACTGAACCTT ACCTTCTAAGA TCAACTGACTA TTTCCGTCTT	e110	e218	e262	e265
H139	TGTAGATGAGT ATGTGACAGT AAGGTTCAGT TAGGGATGAAA	e266	e220	e110	e267
N140	ATCGTAACCA TTCCTCTCTTT ACTCATCTACA ATCTCAGCAT	e268	e222	e266	e269
N141	AGGAAGGAATA ATTGGGTCAT TGGTTACGAT TGAAGTAAGGA	e270	e224	e268	e271
S142	TCTTTCACGA AACACTACTCT TATTCCTTCCT AGCTCTCATT	e272	e226	e270	e273

Name	Sequence (5′ → 3′)	Gl. 1/E	Gl. 2/N	Gl. 3/W	Gl. 4/S
H143	TTTTTTTTTTT AGTTGGTTCA TCGTGAAAGA ATTGAAGCAAA	nullT11S	e227	e272	e274
H144	AGAGATGACTT AGGAGGTAGA TTTTTTTTTT TAGTTAGTCGT	e275	e229	nullT10N	e276
S145	TCACCCATTT TGACCTTCTTA AAGTCATCTCT TTTCCGTCTT	e277	e231	e275	e265
H146	ACTTGTAGGAT TAGTGGCAAT AAATGGGTGA AGGAAAGACTA	e279	e87	e277	e280
S147	TAACTGCACA ATAATCCCAGA ATCCTACAAGT TCACAACGTA	e281	e235	e279	e282
H148	ATGTGAGTAGT TTAGGCAAGT TGTGCAGTTA AAATTGGAACA	e283	e237	e281	e102
S149	ATATCGCACA TTTTCCATTGT ACTACTCACAT AATCTCGACA	e285	e239	e283	e286
H150	TGTATGAAGGA ATGTAGCAGT TGTGCGATAT AAAGGCTAGTA	e287	e241	e285	e288
N151	ACACACAAGA TCTATACCGAA TCCTTCATACA AGTTCACACA	e289	e243	e287	e290
N152	AGAGTTTAGGA TAGTGGCAAT TCTTGTGTGT TAGAGAATCGA	e168	e87	e289	e109
S153	TGTACCCAAA TTCCTAACTCT TCCTAAACTCT TCATGCCTTA	e293	e194	e168	e294
H154	ATGTAGGACTT ATGATGAGCA TTTGGGTACA TGGAGACTAAT	e295	e64	e293	e296
N155	ACATGTCACA TCCGACTATTA AAGTCCTACAT TCATCACCAT	e297	e251	e295	e298
H156	ATGGATGTCTA TGGTTCTTGA TGTGACATGT TAATAGTCGGA	e299	e253	e297	e251
S157	TTCCAACAGA ACACGTCTATA TAGACATCCAT TTCAACCCAT	e301	e255	e299	e302
H158	ATGTAGGGTAA AGGTGTTTCT TCTGTTGGAA AAGAGAGTGTA	e303	e257	e301	e304
S159	ACCCTTCTTT TGCTACTACAT TTACCCTACAT ACCATCCAAA	e86	e259	e303	e306
H160	TAGAAGGCATA TTAGGCAAGT AAAGAAGGGT AGCAAAGAAAT	e307	e237	e86	e308
S161	TTCGCTCTAA TCCTGAACTAA TATGCCTTCTA AGCACTTACA	e309	e263	e307	e310
H162	TGGATAGCTTA AAGACGGAAA TTAGAGCGAA ATTAGTGGGTA	e311	e265	e309	e49
B163	ACGATCATCA TTTCATCCCTA TAAGCTATCCA TTCCTGACAA	e313	e267	e311	e314
H164	AGGTATCGTAT ATGCTGAGAT TGATGATCGT AAAATGCAAGA	e315	e269	e313	e316
N165	ACGATCATCA TCCTTACTTCA ATACGATACCT TCAAGACACA	e313	e271	e315	e318
S166	TAAAATTGGCA AATGAGAGCT TGATGATCGT AATGTCTAGGA	e319	e273	e313	e320
S167	TTTTTTTTTT TTTGCTTCAAT TGCCAATTTTA TCAATCAGCT	nullT10S	e274	e319	e321
B168	AACGTCTTCT ACGACTAACTA TTTTTTTTTTT TCACCGTTAT	e142	e276	nullT11N	e323
N169	TTAGGTAGCAT AAGACGGAAA AGAAGACGTT ATAGTGAGGAA	e324	e265	e142	e325
N170	ACAACTTCGA TAGTCTTTCCT ATGCTACCTAA TTACCACCAA	e326	e280	e324	e327
H171	AATGCAGATTT TACGTTGTGA TCGAAGTTGT TTTCATTTGGT	e328	e282	e326	e329
S172	ACACTCCAAA TGTTCCAATTT AAATCTGCATT ATTGTCCCTT	e330	e102	e328	e331
H173	TTTTGATTCGT TGTCGAGATT TTTGGAGTGT ACTTTAGAGGT	e332	e286	e330	e180
S174	ACCATAACGA TACTAGCCTTT ACGAATCAAAA AGCATCAACT	e334	e288	e332	e335
H175	AGGTATCGTAT TGTGTGAACT TCGTTATGGT TCAAGAAGGTA	e315	e290	e334	e42
S176	ACTTGTTCCT TCGATTCTCTA ATACGATACCT AAATCCTGCT	e54	e109	e315	e339
B177	TTAGGTAGCAT TAAGGCATGA AGGAACAAGT AGGTTAGACAT	e324	e294	e54	e341
S178	AAACCTGCTA ATTAGTCTCCA ATGCTACCTAA ATTACGCTCT	e342	e296	e324	e343
B179	TAGGAACAGAA ATGGTGATGA TAGCAGGTTT ACAGGAAGATA	e344	e298	e342	e345
S180	AATCACAGCT TCCGACTATTA TTCTGTTCCTA AGCCTCATTT	e159	e251	e344	e347
H181	TGGTAAGTACT ATGGGTTGAA AGCTGTGATT AATGTCTAGGA	e348	e302	e159	e320
S182	TACGTTCCAA TACACTCTCTT AGTACTTACCA TCGACTTTCA	e127	e304	e348	e351
N183	AGAAGTCAGAT TTTGGATGGT TTGGAACGTA AGCAAAAGAAA	e352	e306	e127	e177
N184	ATACACACGT ATTTCTTTGCT ATCTGACTTCT AGCTCTCATT	e354	e308	e352	e273
H185	ATGGTTTTGTT TGTAAGTGCT ACGTGTGTAT TGAAAGAGGTA	e356	e310	e354	e357
S186	ACGTATCACA TACCCACTAAT AACAAAACCAT ACCAAGCTAA	e358	e49	e356	e359
H187	TTGAGGGATAT TTGTCAGGAA TGTGATACGT ATATGGTAGCT	e61	e314	e358	e89
S188	ACACACAAGA TCTTGCATTTT ATATCCCTCAA TTAACAGCCA	e289	e316	e61	e363
N189	AGGAGTAAACT TGTGTCTTGA TCTTGTGTGT AGTTGGTAGTA	e364	e318	e289	e365
B190	AACCTCGAAA TCCTAGACATT AGTTTACTCCT ATCACCACAA	e131	e320	e364	e59
H191	TTTTTTTTTTT AGCTGATTGA TTTCGAGGTT AGAATCAGGAT	nullT11S	e321	e131	e368
N192	ACTGAGGATAA ATAACGGTGA TTTTTTTTTT AAAAGTGAACA	e250	e323	nullT10N	e370
N193	AGCCATCTTT TTCCTCACTAT TTATCCTCAGT AGCCTTTCTT	e371	e325	e250	e27
N194	ACAGTAGGATT TTGGTGGTAA AAAGATGGCT TGTAGGAATCT	e157	e327	e371	e374
S195	AACACCTCAT ACCAAATGAAA AATCCTACTGT TGCCATATCA	e375	e329	e157	e376
H196	AGAACTAAGGA AAGGGACAAT ATGAGGTGTT TATGACTGTGA	e136	e331	e375	e378
S197	AACTTCTCGA ACCTCTAAAGT TCCTTAGTTCT AAGACCAACA	e3	e180	e136	e380
H198	AGGAGTTACTT AGTTGATGCT TCGAGAAGTT TGAAAGAGGTA	e381	e335	e3	e357
S199	TCGCTTTCTA TACCTTCTTGA AAGTAACTCCT TCCAATCCAA	e383	e42	e381	e143
H200	TATGGAGTTCT AGCAGGATTT TAGAAAGCGA AGAAGTAGACA	e385	e339	e383	e386
B201	TCCACGTTAT ATGTCTAACCT AGAACTCCATA AACCAGAACT	e387	e341	e385	e388

Name	Sequence ($5' \to 3'$)	Gl. 1/E	Gl. 2/N	Gl. 3/W	Gl. 4/S
H202	AGGGATTTAGT AGAGCGTAAT ATAACGTGGA AAAGCATTGAT	e389	e343	e387	e390
S203	ACGAACCATA TATCTTCCTGT ACTAAATCCCT AGCACTTACA	e391	e345	e389	e310
H204	TAACTGGAAGA AAATGAGGCT TATGGTTCGT AAAAATGGACA	e393	e347	e391	e394
N205	ACATCCATGA TCCTAGACATT TCTTCCAGTTA ATTCACTCGA	e151	e320	e393	e396
N206	AATAGGGTACA TGAAAGTCGA TCATGGATGT TTCTGTAGTGA	e93	e351	e151	e398
N207	AAAAAACGCA TTTCTTTTGCT TGTACCCTATT TTCCTTCACA	e399	e177	e93	e400
H208	AGTAATCTGGT AATGAGAGCT TGCGTTTTTT ATTGTTTGACA	e401	e273	e399	e38
N209	ATCACGACTT TACCTCTTTCA ACCAGATTACT TTGCACCTTA	e403	e357	e401	e404
H210	TCGAGAGATTA TTAGCTTGGT AAGTCGTGAT TTTACTGGGTA	e405	e359	e403	e406
N211	AATCCGAACT AGCTACCATAT TAATCTCTCGA TTCTGCTCAA	e248	e89	e405	e408
H212	TTCTATGTGGA TGGCTGTTAA AGTTCGGATT TTATGTTTGCA	e213	e363	e248	e410
S213	ATTGCCTACA TACTACCAACT TCCACATAGAA TCGTCTTCAT	e411	e365	e213	e412
H214	AGGAAGGAATA TTGTGGTGAT TGTAGGCAAT TGATTAGGAGT	e270	e59	e411	e414
S215	TTTTTTTTTT ATCCTGATTCT TATTCCTTCCT AACGTCATCT	nullT10S	e368	e270	e415
S216	TTGTTCCCTT TGTTCACTTTT TTTTTTTTTTT ATCTTCCGAA	e416	e370	nullT11N	e417
H217	TAAATGGGAGA AAGAAAGGCT AAGGGAACAA AGATCTGAGTT	e418	e27	e416	e419
N218	ATCTGCTTCA AGATTCCTACA TCTCCCATTTA ACTTGCCTAA	e420	e374	e418	e237
H219	TTGGTTTGAAT TGATATGGCA TGAAGCAGAT ATAGCAGGATA	e422	e376	e420	e423
N220	AAACTCCGAA TCACAGTCATA ATTCAAACCAA ACATCGCTAA	e39	e378	e422	e425
H221	TTTTGATTCGT TGTTGGTCTT TTCGGAGTTT ATATAGAGGCA	e332	e380	e39	e427
S222	TTTCGTCTCA TACCTCTTTCA ACGAATCAAAA TAAGCCACAA	e428	e357	e332	e429
H223	ACTATGTGAGT TTGGATTGGA TGAGACGAAA ATTACGAGAGA	e430	e143	e428	e431
S224	AGCCATCTTT TGTCTACTTCT ACTCACATAGT TAACACAGCA	e371	e386	e430	e433
H225	TAGCATGAGTA AGTTCTGGTT AAAGATGGCT AATGTCTAGGA	e434	e388	e371	e320
S226	ACACGACTAA ATCAATGCTTT TACTCATGCTA ATGACCCAAT	e436	e390	e434	e224
H227	TAGTGGTTGTA TGTAAGTGCT TTAGTCGTGT TTTTTAAGCGT	e438	e310	e436	e70
S228	TTTACTGCCT TGTCCATTTTT TACAACCACTA ACATACACGT	e440	e394	e438	e441
N229	AAGTTAGGGTA TCGAGTGAAT AGGCAGTAAA AAAATGCAAGA	e84	e396	e440	e316
B230	TTCCAACAGA TCACTACAGAA TACCCTAACTT ACGTTAACCT	e301	e398	e84	e445
H231	TAAAATTGGCA TGTGAAGGAA TCTGTTGGAA TACATGGAAGA	e319	e400	e301	e447
S232	AGCCTACAAA TGTCAAACAAT TGCCAATTTTA ACCAAACAGA	e114	e38	e319	e449
N233	AGAGAATGAGT TAAGGTGCAA TTTGTAGGCT TAGTTGTGAGA	e450	e404	e114	e451
S234	TTCCCAAAGA TACCCAGTAAA ACTCATTCTCT AATCGTTCCT	e187	e406	e450	e12
B235	AGTGTTTTGAA TTGAGCAGAA TCTTTGGGAA TAAGAAGGTCA	e454	e408	e187	e231
N236	ATCGTAACCA TGCAAACATAA TTCAAAACACT ACATAACCGA	e268	e410	e454	e457
H237	TAGCTAATGGA ATGAAGACGA TGGTTACGAT TAGTTGTGAGA	e458	e412	e268	e451
N238	TACCGTCATT ACTCCTAATCA TCCATTAGCTA TCCAATCCAA	e460	e414	e458	e143
B239	TTTTTTTTTTT AGATGACGTT AATGACGGTA AGAGTTATGGA	nullT11S	e415	e460	e462
H240	AGAGTGATGAT TTCGGAAGAT TTTTTTTTTT TTGTTGCATAA	e463	e417	nullT10N	e464
S241	ACTTGTTCCT AACTCAGATCT ATCATCACTCT ATCGACCTTT	e54	e419	e463	e466
N242	TGAGTATCTGT TTAGGCAAGT AGGAACAAGT TGAAGTAAGGA	e467	e237	e54	e271
S243	ATATGCACCA TATCCTGCTAT ACAGATACTCA ATTCACTCGA	e223	e423	e467	e396
N244	AGGGTTCTAAT TTAGCGATGT TGGTGCATAT TAGGGTAACAT	e161	e425	e223	e117
S245	ACAACTCACT TGCCTCTATAT ATTAGAACCCT ACATAACCGA	e473	e427	e161	e457
H246	AGTCGATGATA TTGTGGCTTA AGTGAGTTGT ATGAGTTAGGT	e475	e429	e473	e476
S247	ACAGCCATTA TCTCTCGTAAT TATCATCGACT AATCTCCGAA	e46	e431	e475	e478
H248	ATGGATTAGGA TGCTGTGTTA TAATGGCTGT ACAATTGGAAA	e479	e433	e46	e480
S249	TCAGCATCTT TCCTAGACATT TCCTAATCCAT ACACCATCAT	e481	e320	e479	e204
B250	TTTTGTTCAGT ATTGGGTCAT AAGATGCTGA TGGAGTAAGAT	e483	e224	e481	e186
N251	AATTCCCAGT ACGCTTAAAAA ACTGAACAAAA ATAACGCTCT	e485	e70	e483	e486
H252	TTGGGATATCT ACGTGTATGT ACTGGGAATT TAATACGAGGA	e487	e441	e485	e74
N253	ATACTGCCAA TCTTGCATTTT AGATATCCCAA TCTTGTCACA	e489	e316	e487	e490
H254	TGGATGATACT AGGTTAACGT TTGGCAGTAT TATAGACGTGT	e41	e445	e489	e255
N255	ACTTTCTCGT TCTTCCATGTA AGTATCATCCA ATCGCTACAA	e493	e447	e41	e494
H256	TGGATAGCTTA TCTGTTTGGT ACGAGAAAGT ATATAGGACGT	e311	e449	e493	e496
N257	TTCATCCCAA TCTCACAACTA TAAGCTATCCA ATCCAACCAT	e497	e451	e311	e498
H258	AATAGGGTACA AGGAACGATT TTGGGATGAA TGGAATAGTCA	e93	e12	e497	e500
S259	ACAACTTCGA TGACCTTCTTA TGTACCCTATT AGTTCCAACA	e326	e231	e93	e502
H260	AGTAGATACGT TCGGTTATGT TCGAAGTTGT AAGGTAGTCAT	e503	e457	e326	e504

**Name**	**Sequence** ($5' \to 3'$)	**Gl. 1/E**	**Gl. 2/N**	**Gl. 3/W**	**Gl. 4/S**
S261	ACACGACTAA TCTCACAACTA ACGTATCTACT AAAGCACTCT	e436	e451	e503	e506
N262	TAGAGGAAAGT TTGGATTGGA TTAGTCGTGT TTTTGGTTCAA	e153	e143	e436	e113
S263	TTTTTTTTTT TCCATAACTCT ACTTTCCTCTA ATTCCAACCT	nullT10S	e462	e153	e509
S264	TTCACCACTT TTATGCAACAA TTTTTTTTTT TCATAGCCAA	e510	e464	nullT11N	e167
H265	TGGCTTTTTTA AAAGGTCGAT AAGTGGTGAA ATTACGAGAGA	e512	e466	e510	e431
N266	AAACCCATGA TCCTTACTTCA TAAAAAAGCCA AAACTGTCCA	e514	e271	e512	e515
B267	ATTAATTGCGA TCGAGTGAAT TCATGGGTTT AAAGAGAGGAA	e516	e396	e514	e222
S268	ATCGACATCA ATGTTACCCTA TCGCAATTAAT TCTGACAACA	e518	e117	e516	e519
B269	AGGGTTAGAAT TCGGTTATGT TGATGTCGAT ACAGGAAGATA	e520	e457	e518	e345
S270	TGCTCCATTA ACCTAACTCAT ATTCTAACCCT ATCACCCAAA	e178	e476	e520	e4
H271	TATGACAGAGT TTCGGAGATT TAATGGAGCA ATAGGACATGA	e524	e478	e178	e525
S272	TCGATCAACA TTTCCAATTGT ACTCTGTCATA TATCGCTCAA	e526	e480	e524	e527
N273	AGACTAATGGA ATGATGGTGT TGTTGATCGA TGGAGTAAGAT	e528	e204	e526	e186
N274	TTCACCACTT ATCTTACTCCA TCCATTAGTCT ACCATCCAAA	e510	e186	e528	e306
N275	AGTCTAAAGGA AGAGCGTTAT AAGTGGTGAA AGAAGTAGACA	e532	e486	e510	e386
S276	AACGCAATTT TCCTCGTATTA TCCTTTAGACT ACTAGCACAA	e534	e74	e532	e19
H277	TGTTTGAACTT TGTGACAAGA AAATTGCGTT AGGAGCATATA	e133	e490	e534	e537
S278	ACATTCACCT ACACGTCTATA AAGTTCAAACA ACTAACGACA	e538	e255	e133	e156
N279	TTATCTGTGGA TTGTAGCGAT AGGTGAATGT TATTGAGGCTA	e125	e494	e538	e541
S280	ACCAACTTGA ACGTCCTATAT TCCACAGATAA ATCCAACGTA	e542	e496	e125	e543
H281	ACAGGTTAGAT ATGGTTGGAT TCAAGTTGGT ACAGGAAGATA	e544	e498	e542	e345
N282	TACAACGTCA TGACTATTCCA ATCTAACCTGT AATCCGTTCT	e546	e500	e544	e547
N283	TAAGAGTGTGA TGTTGGAACT TGACGTTGTA TTAAGGGAGAA	e548	e502	e546	e549
S284	TTTCGTCTCA ATGACTACCTT TCACACTCTTA ACGATCTCAA	e428	e504	e548	e551
H285	TAGGAAAGTCA AGAGTGCTTT TGAGACGAAA TTAGATACGGA	e552	e506	e428	e553
N286	TAACAACCGT TTGAACCAAAA TGACTTTCCTA ATGACCCAAT	e554	e113	e552	e224
H287	TTTTTTTTTTT AGGTTGGAAT ACGGTTGTTA ATCAGGATGTA	nullT11S	e509	e554	e556
N288	TTCGAAATGTT TTGGCTATGA TTTTTTTTTT TTGTTGCATAA	e557	e167	nullT10N	e464
S289	AACCTGAACA TCTCTCGTAAT AACATTTCGAA AAATCCGACT	e559	e431	e557	e560
H290	AGGTATCGTAT TGGACAGTTT TGTTCAGGTT AGGTTAGACAT	e315	e515	e559	e341
S291	ACCTGACATT TTCCTCTCTTT ATACGATACCT ACATAACCGA	e256	e222	e315	e457
H292	ACGTTGATTTT TGTTGTCAGA AATGTCAGGT TTAAAGAGGGA	e565	e519	e256	e566
S293	AACTCGCTAA TATCTTCCTGT AAAATCAACGT TTCTTCTGCT	e567	e345	e565	e568
H294	AACGTGAAATT TTTGGGTGAT TTAGCGAGTT TCTAAGTGTGA	e258	e4	e567	e173
B295	TCTTTCACGT TCATGTCCTAT AATTTCACGTT ACTTGACACA	e43	e525	e258	e572
N296	ATTTGCTGATT TTGAGCGATA ACGTGAAAGA TTTAAATGCGA	e573	e527	e43	e190
S297	TTCAGCTCAA ATCTTACTCCA AATCAGCAAAT ATTTCAGCCT	e575	e186	e573	e576
H298	AGTTGATAGGT TTTGGATGGT TTGAGCTGAA TGAGTAGAACA	e189	e306	e575	e578
B299	ATCCACCATT TGTCTACTTCT ACCTATCAACT AACGTCATCT	e215	e386	e189	e415
B300	ACATATGGAGT TTGTGCTAGT AATGGTGGAT TTTCATTTGGT	e581	e19	e215	e329
S301	ACCCTTCAAT TATATGCTCCT ACTCCATATGT ATTCACTCGA	e71	e537	e581	e396
H302	TTTTGATTCGT TGTCGTTAGT ATTGAAGGGT AGGATATAGCA	e332	e156	e71	e94
S303	AACTGCTCTT TAGCCTCAATA ACGAATCAAAA ACGATCTCAA	e587	e541	e332	e551
H304	AGTAGTGACAT TACGTTGGAT AAGAGCAGTT AAGAAAAGCAA	e589	e543	e587	e590
S305	TCCACTCAAA TATCTTCCTGT ATGTCACTACT AGCCTCATTT	e591	e345	e589	e347
H306	TGGCTTTTTTA AGAACGGATT TTTGAGTGGA AGTGAGTAAGA	e512	e547	e591	e594
S307	ACAGCCATTA TTCTCCCTTAA TAAAAAAGCCA TCAACCTGTT	e46	e549	e512	e596
N308	TCGGAATTAGA TTGAGATCGT TAATGGCTGT AGATAGCGTAT	e597	e551	e46	e598
N309	AGACAACCTT TCCGTATCTAA TCTAATTCCGA AAACTGTCCA	e599	e553	e597	e515
H310	TAAGAGTGTGA ATTGGGTCAT AAGGTTGTCT TAAAATGTGCA	e548	e224	e599	e602
S311	TTTTTTTTTT TACATCCTGAT TCACACTCTTA ACGATCAACT	nullT10S	e556	e548	e603
S312	AGCCTACAAA TTATGCAACAA TTTTTTTTTT TCAAGAACCA	e114	e464	nullT11N	e253
H313	AGACGGTAATA AGTCGGATTT TTTGTAGGCT TTCTGTAGTGA	e606	e560	e114	e398
S314	TTACGTCCAA ATGTCTAACCT TATTACCGTCT ACGATCTCAA	e608	e341	e606	e551
H315	ATGAAAATGCT TCGGTTATGT TTGGACGTAA TAAAAGGCAAA	e610	e457	e608	e6
S316	AAACCTGCTA TCCCTCTTTAA AGCATTTTCAT TCACAAACCT	e342	e566	e610	e613
H317	AAAAGGGTCTA AGCAGAAGAA TAGCAGGTTT AGTTGGTAGTA	e614	e568	e342	e365
S318	TTAAGCACCT TCACACTTAGA TAGACCCTTTT TATCAACGCT	e616	e173	e614	e36
H319	TCGTAGTATGT TGTGTCAAGT AGGTGCTTAA AGAGAAGATGT	e618	e572	e616	e619

Name	Sequence (5′ → 3′)	Gl. 1/E	Gl. 2/N	Gl. 3/W	Gl. 4/S
B320	AGACCACTTT TCGCATTTAAA ACATACTACGA TACACAAGCT	e620	e190	e618	e621
H321	AGAGAATGAGT AGGCTGAAAT AAAGTGGTCT ATTAGTCTGGA	e450	e576	e620	e623
S322	ACCAGCATAA TGTTCTACTCA ACTCATTCTCT TTCTGCTCAA	e624	e578	e450	e408
H323	TCGGAATTAGA AGATGACGTT TTATGCTGGT ATTATCGGAGA	e597	e415	e624	e627
S324	TCGCATTCTA ACCAAATGAAA TCTAATTCCGA AAGTTCCCAT	e628	e329	e597	e629
H325	TAGATGTACGT TCGAGTGAAT TAGAATGCGA TTAGAGTGACA	e630	e396	e628	e631
S326	AATACCACGA TGCTATATCCT ACGTACATCTA AAACTGTCCA	e632	e94	e630	e515
H327	TGAGTATCTGT TTGAGATCGT TCGTGGTATT TAGGAGAAACT	e467	e551	e632	e635
S328	TACAACGTCA TTGCTTTTCTT ACAGATACTCA ACCAAGCTAA	e546	e590	e467	e359
H329	AGAAGGATGAT AAATGAGGCT TGACGTTGTA AGGTTTAGGTA	e638	e347	e546	e639
N330	AAACCTGCTA TCTTACTCACT ATCATCCTTCT TTCACCTGTT	e342	e594	e638	e641
H331	AGAGATGACTT AACAGGTTGA TAGCAGGTTT AGAGTTGAGTA	e275	e596	e342	e643
S332	AACCCTCTTT ATACGCTATCT AAGTCATCTCT AAGACCACAT	e155	e598	e275	e645
H333	TTTAGTGGAGA TGGACAGTTT AAAGAGGGTT ACTAGATGGAA	e646	e515	e155	e647
S334	ACGACCAATA TGCACATTTTA TCTCCACTAAA AACGTACCTT	e648	e602	e646	e649
H335	TTTTTTTTTTT AGTTGATCGT TATTGGTCGT ATAGGACAAGT	nullT11S	e603	e648	e650
H336	TTCGGATAGAA TGGTTCTTGA TTTTTTTTTT TGGGTGTATAT	e651	e253	nullT10N	e652
S337	ACCTTCAGTT TCACTACAGAA TTCTATCCGAA AAGTTCCCAT	e653	e398	e651	e629
B338	TGTAGAGGATT TTGAGATCGT AACTGAAGGT AAATTGGAACA	e655	e551	e653	e102
N339	TCGATCAACA TTTGCCTTTTA AATCCTCTACA ACACTTCCAT	e526	e6	e655	e658
H340	AGGACTAATGT AGGTTTGTGA TGTTGATCGA TAAAACGTGTT	e659	e613	e526	e660
S341	ACCAACAAGA TACTACCAACT ACATTAGTCCT AAACCTCCTT	e661	e365	e659	e179
H342	TGGAAGAGTAT AGCGTTGATA TCTTGTTGGT AGGTTCTAGTT	e101	e36	e661	e145
S343	AGCACCTTTA ACATCTTCTCT ATACTCTTCCA TTCCAGTTCA	e665	e619	e101	e666
H344	TTTTGTTCAGT AGCTTGTGTA TAAAGGTGCT ATAGTGAGGAA	e483	e621	e665	e325
S345	TACACGAACA TCCAGACTAAT ACTGAACAAAA TCCCAGAAAA	e669	e623	e483	e670
H346	TTCGAAATGTT TTGAGCAGAA TGTTCGTGTA TGAAGTAAGGA	e557	e408	e669	e271
N347	ACATTCACCT TCTCCGATAAT AACATTTCGAA ATAACGCTCT	e538	e627	e557	e486
H348	AGACTAATGGA ATGGGAACTT AGGTGAATGT ATTGTAGGACT	e528	e629	e538	e676
S349	AACGCAATTT TGTCACTCTAA TCCATTAGTCT TCATGCCTTA	e534	e631	e528	e294
N350	TGCAGTTTTTA TGGACAGTTT AAATTGCGTT AGATCTGAGTT	e48	e515	e534	e419
N351	TTTACCGACA AGTTTCTCCTA TAAAAACTGCA TCCCAATTGA	e681	e635	e48	e682
H352	TAGGAACAGAA TTAGCTTGGT TGTCGGTAAA TTTAAATGCGA	e344	e359	e681	e190
S353	ACCAGCATAA TACCTAAACCT TTCTGTTCCTA ATTCTGCACT	e624	e639	e344	e686
H354	ACAAGGATAGA AACAGGTGAA TTATGCTGGT TGGGTACTTTA	e116	e641	e624	e688
S355	AGACCACTTT TACTCAACTCT TCTATCCTTGT ATCGCTACAA	e620	e643	e116	e494
H356	TAAAATTGGCA ATGTGGTCTT AAAGTGGTCT TAAGAAGGTCA	e319	e645	e620	e231
N357	AGCCATCTTT TTCCATCTAGT TGCCAATTTTA ACAAGTCCAT	e371	e647	e319	e694
H358	AGTTGTAGTCT AAGGTACGTT AAAGATGGCT TACATGATGGA	e695	e649	e371	e10
S359	TTTTTTTTTT ACTTGTCCTAT AGACTACAACT AGAAACCCAA	nullT10S	e650	e695	e697
B360	TCAATCTCGT ATATACACCCA TTTTTTTTTTT TCAAGACACA	e698	e652	nullT11N	e318
H361	ATTGCAATGAT ATGGGAACTT ACGAGATTGA TAGGGTAACAT	e700	e629	e698	e117
B362	TCCTATCCCT TGTTCCAATTT ATCATTGCAAT ACACACTCAA	e702	e102	e700	e703
H363	AGGTAATGGTA ATGGAAGTGT AGGGATAGGA TGCATATAGGT	e704	e658	e702	e705
S364	AACTTCAGCT AACACGTTTTA TACCATTACCT TAACTGCCAA	e99	e660	e704	e707
N365	AACTGATAGGA AAGGAGGTTT AGCTGAAGTT TGAGGTATGAT	e708	e179	e99	e45
S366	TTCGTACACA AACTAGAACCT TCCTATCAGTT AGCCTCATTT	e63	e145	e708	e347
H367	TTAAGGACTGA TGAACTGGAA TGTGTACGAA AGAATATGGGT	e712	e666	e63	e713
S368	ATTCGCTACT TTCCTCACTAT TCAGTCCTTAA TTTTTTTTTT	e714	e325	e712	nullT10E
H369	TGAGGATGTTA TTTTCTGGGA AGTAGCGAAT TTTTTTTTTTT	e715	e670	e714	nullT11E
S370	TCACACAGTT TCCTTACTTCA TAACATCCTCA TTTTTTTTTT	e716	e271	e715	nullT10E
H371	AGTTCTGAGAT AGAGCGTTAT AACTGTGTGA TTTTTTTTTTT	e717	e486	e716	nullT11E
N372	ACACATCCAT AGTCCTACAAT ATCTCAGAACT TTTTTTTTTT	e718	e676	e717	nullT10E
H373	AAAGAGGAGAT TAAGGCATGA ATGGATGTGT TTTTTTTTTTT	e719	e294	e718	nullT11E
S374	TCCATCGAAA AACTCAGATCT ATCTCCTCTTT TTTTTTTTTT	e720	e419	e719	nullT10E
H375	TGTAGATGAGT TCAATTGGGA TTTCGATGGA TTTTTTTTTTT	e266	e682	e720	nullT11E
S376	TACCGTCATT TCGCATTTAAA ACTCATCTACA TCAAGACACA	e460	e190	e266	e318
H377	AGAAGTCAGAT AGTGCAGAAT AATGACGGTA TAAGTGACTGA	e352	e686	e460	e725
N378	ACCCTTTGAT TAAAGTACCCA ATCTGACTTCT ACATCGCTAA	e726	e688	e352	e425

Name	Sequence (5′ → 3′)	Gl. 1/E	Gl. 2/N	Gl. 3/W	Gl. 4/S
N379	TGGCTATTAGA TTGTAGCGAT ATCAAAGGGT AGAAAGGAAGA	e65	e494	e726	e729
B380	ATCGCAACTA TGACCTTCTTA TCTAATAGCCA ATCGTCCAAT	e730	e231	e65	e731
H381	AGTGTTTTGAA ATGGACTTGT TAGTTGCGAT TGAGAGAAAGA	e454	e694	e730	e733
S382	TTCTCTTGCT TCCATCATGTA TTCAAAACACT AACACTCGAT	e734	e10	e454	e735
B383	TTTTTTTTTTT TTGGGTTTCT AGCAAGAGAA AGGTCATTAGT	nullT11S	e697	e734	e736
H384	AGTCTAAAGGT TGTGTCTTGA TTTTTTTTTT AGTGTAAGAGA	e737	e318	nullT10N	e738
S385	TTCACGAACT ATGTTACCCTA ACCTTTAGACT ACTCAACGTA	e739	e117	e737	e740
H386	TCGGTAGATTA TTGAGTGTGT AGTTCGTGAA ATGGTAGATCA	e741	e703	e739	e742
B387	ATCTGCTTCA ACCTATATGCA TAATCTACCGA ACACCATCAT	e420	e705	e741	e204
H388	ACATATGGAGT TTGGCAGTTA TGAAGCAGAT TTAGAGTGTCT	e581	e707	e420	e746
S389	AAACCCAAGA ATCATACCTCA ACTCCATATGT AAAACGCAAT	e747	e45	e581	e44
B390	TGGAAGAGTAT AAATGAGGCT TCTTGGGTTT AATGTGGAAAA	e101	e347	e747	e141
S391	TTTTTTTTTT ACCCATATTCT ATACTCTTCCA AGAACCCAAA	nullT10S	e713	e101	e751
B392	TATATGGTCGT TGTGTCTTGA TTTTTTTTTT TATCGGAGAAA	e193	e318	nullT10N	e753
S393	AAACCCATGA TCAGTCACTTA ACGACCATATA ATCGCTACAA	e514	e725	e193	e494
H394	TATGGAGCTAT TTAGCGATGT TCATGGGTTT AGGAAATAGGA	e756	e425	e514	e757
S395	TGATCCACAT TCTTCCTTTCT ATAGCTCCATA ATGCATACCA	e758	e729	e756	e124
H396	TGTAGAGGATT ATTGGACGAT ATGTGGATCA AGGAGCATATA	e655	e731	e758	e537
S397	AACGTCTTCT TCTTTCTCTCA AATCCTCTACA TTCGATCACT	e142	e733	e655	e763
H398	TTTTTGTCTGT ATCGAGTGTT AGAAGACGTT ATAGGACATGA	e764	e735	e142	e525
S399	TTTTTTTTTT ACTAATGACCT ACAGACAAAAA ATCTTCTGCA	nullT10S	e736	e764	e135
B400	AGCTTCATCA TCTCTTACACT TTTTTTTTTTT TTTCCTTCCA	e146	e738	nullT11N	e768
H401	TAGAAGGCATA TACGTTGAGT TGATGAAGCT TATATGACGGA	e307	e740	e146	e210
S402	ATTCCGTACA TGATCTACCAT TATGCCTTCTA TTCTGACCAA	e771	e742	e307	e184
B403	AGAATTAGGGA ATGATGGTGT TGTACGGAAT TAGGGTAACAT	e108	e204	e771	e117
S404	AACTTCTCGA AGACACTCTAA TCCCTAATTCT AACACAGACA	e3	e746	e108	e776
H405	AGTGGATCTTA ATTGCGTTTT TCGAGAAGTT TGTGACAGATA	e777	e44	e3	e778
S406	TAACAACCGT TTTTCCACATT TAAGATCCACT ACATTCCACT	e554	e141	e777	e780
H407	TTTTTTTTTTT TTTGGGTTCT ACGGTTGTTA TTGACGAAAAT	nullT11S	e751	e554	e781
S408	ACCATGTCAT TTTCTCCGATA TTTTTTTTTTT TCAAGAACCA	e782	e753	nullT11N	e253
H409	AGGTGAAGTAT TTGTAGCGAT ATGACATGGT ATGACAGTAGT	e784	e494	e782	e785
S410	TTCGCTCTAA TCCTATTTCCT ATACTTCACCT TCACAACGTA	e309	e757	e784	e282
B411	TGAGGACTATT TGGTATGCAT TTAGAGCGAA TAGAGGAACAT	e76	e124	e309	e789
S412	TTTACTGCCT TATATGCTCCT AATAGTCCTCA TCATGACACA	e440	e537	e76	e791
H413	AGGGATTCATA AGTGATCGAA AGGCAGTAAA TACATGATGGA	e792	e763	e440	e10
S414	TTCTCTTGCT TCATGTCCTAT TATGAATCCCT AGTTCCAACA	e734	e525	e792	e502
H415	TTTTTTTTTTT TGCAGAAGAT AGCAAGAGAA ATTACAGAGGT	nullT11S	e135	e734	e796
H416	AGGGATGTAAT TGGAAGGAAA TTTTTTTTTT AGAGTGGATAA	e88	e768	nullT10N	e798
S417	TGCTACACAA TCCGTCATATA ATTACATCCCT ACACCATCAT	e799	e210	e88	e204
H418	AAGTGAGGATA TTGGTCAGAA TTGTGTAGCA AAAAATGGACA	e801	e184	e799	e394
B419	ATTCCGTACA ATGTTACCCTA TATCCTCACTT AATCCGTTCT	e771	e117	e801	e547
H420	ATTTGCTGATT TGTCTGTGTT TGTACGGAAT TTAAGGAAGGT	e573	e776	e771	e806
S421	AAACCCAAGA TATCTGTCACA AATCAGCAAAT ACAAAGTCCA	e747	e778	e573	e808
H422	AGTATGTCTGA AGTGGAATGT TCTTGGGTTT TCAGTTTAGGA	e809	e780	e747	e810
B423	TTTTTTTTTT ATTTTCGTCAA TCAGACATACT AAAGACACCT	nullT10S	e781	e809	e811
N424	AGTAGATACGT TGGTTCTTGA TTTTTTTTTT TATCGGAGAAA	e503	e253	nullT10N	e753
S425	ACCACTGATT ACTACTGTCAT ACGTATCTACT AAACGCAAAT	e814	e785	e503	e815
H426	TGGGATCTATT TACGTTGTGA AATCAGTGGT TATACGAAGGT	e816	e282	e814	e817
B427	TACACGTCAT ATGTTCCTCTA AATAGATCCCA ATCGACTCAA	e818	e789	e816	e819
H428	AGGTAAGAGAA TGTGTCATGA ATGACGTGTA AGGTCTTGATA	e820	e791	e818	e821
N429	AGCTCAATCA TCCATCATGTA TTCTCTTACCT ACGTTAACCT	e822	e10	e820	e445
H430	TATGGAGCTAT TGTTGGAACT TGATTGAGCT AGAACTATGGT	e756	e502	e822	e154
S431	TTTTTTTTTT ACCTCTGTAAT ATAGCTCCATA TCAAACGACT	nullT10S	e796	e756	e826
S432	TATTCTCGCA TTATCCACTCT TTTTTTTTTTT ACAGCATACT	e827	e798	nullT11N	e828
H433	AGGATGTACTT ATGATGGTGT TGCGAGAATA ACAATTGGAAA	e829	e204	e827	e480
S434	ACCAGCTTTA TGTCCATTTTT AAGTACATCCT TCGACTTTCA	e831	e394	e829	e351
H435	AACAGAGAGAT AGAACGGATT TAAAGCTGGT TATTCGAGGTA	e833	e547	e831	e834
S436	ATTCGACCAT ACCTTCCTTAA ATCTCTCTGTT AGCCATACAT	e835	e806	e833	e836
H437	AGGTATGGAAT TGGACTTTGT ATGGTCGAAT TGGAGTAAGAT	e837	e808	e835	e186

Name	Sequence (5′ → 3′)	Gl. 1/E	Gl. 2/N	Gl. 3/W	Gl. 4/S
S438	ACGATCATCA TCCTAAACTGA ATTCCATACCT ATTCTCAGCT	e313	e810	e837	e840
H439	TTTTTTTTTTT AGGTGTCTTT TGATGATCGT ATTTTGCAGAT	nullT11S	e811	e313	e841
S440	TTCCCAAAGA TTTCTCCGATA TTTTTTTTTTT ACGTTCACTA	e187	e753	nullT11N	e843
H441	TTTGACTGTTT ATTTGCGTTT TCTTTGGGAA TTTTCGATTGT	e844	e815	e187	e845
S442	TTCTACGCAT ACCTTCGTATA AAACAGTCAAA TACAAGCCAA	e58	e817	e844	e847
H443	TGAGTATCTGT TTGAGTCGAT ATGCGTAGAA TAGAGGAACAT	e467	e819	e58	e789
S444	TTCGCATCTA TATCAAGACCT ACAGATACTCA ACTAGCACAA	e163	e821	e467	e19
H445	AGATGGAGATT AGGTTAACGT TAGATGCGAA TAGGTTTCAGA	e852	e445	e163	e853
S446	TAACTCGCTT ACCATAGTTCT AATCTCCATCT ATCCAACGTA	e854	e154	e852	e543
H447	TTTTTTTTTTT AGTCGTTTGA AAGCGAGTTA AGGTCATTAGT	nullT11S	e826	e854	e736
H448	ATCTTAGGGAA AGTATGCTGT TTTTTTTTTT AGAGTAAGGTT	e857	e828	nullT10N	e858
S449	TTTCCAGTCA TTTCCAATTGT TTCCCTAAGAT TTCGATCACT	e859	e480	e857	e763
H450	AGGAGTGTATT TGAAAGTCGA TGACTGGAAA ACAGGAAGATA	e861	e351	e859	e345
S451	ATTTCTCCGT TACCTCGAATA AATACACTCCT TCCACAAGTT	e863	e834	e861	e864
H452	TTGGGATATCT ATGTATGGCT ACGGAGAAAT ACTTTAGAGGT	e487	e836	e863	e180
S453	ATCGCTAACA ATCTTACTCCA AGATATCCCAA TCACAACGTA	e867	e186	e487	e282
H454	TGGCTATTAGA AGCTGAGAAT TGTTAGCGAT TGTAGGAATCT	e65	e840	e867	e374
S455	TTTTTTTTTT ATCTGCAAAAT TCTAATAGCCA ATTCACCGTA	nullT10S	e841	e65	e90
H456	TTGGTTTGAAT TAGTGAACGT TTTTTTTTTT AGGCTAAGTAT	e422	e843	nullT10N	e873
S457	ACCTCACATT ACAATCGAAAA ATTCAAACCAA ACCAAGCTAA	e67	e845	e422	e359
B458	TTATCTGTGGA TTGGCTTGTA AATGTGAGGT ACGGAAATTTT	e125	e847	e67	e66
S459	ACGACCAATA ATGTTCCTCTA TCCACAGATAA AGCATCAACT	e648	e789	e125	e335
B460	AGTCGATGATA TTGTGCTAGT TATTGGTCGT TCTGGGATTAT	e475	e19	e648	e235
S461	ACACACAAGA TCTGAAACCTA TATCATCGACT AAACGCAAAT	e289	e853	e475	e815
H462	TGATAGGACTT TACGTTGGAT TCTTGTGTGT TCAGTTTAGGA	e884	e543	e289	e810
B463	TTTTTTTTTT ACTAATGACCT AAGTCCTATCA TATGCACTCA	nullT10S	e736	e884	e75
S464	ACAATCCGAT AACCTTACTCT TTTTTTTTTTT TTTTTTTTTT	e887	e858	nullT11N	nullT10E
N465	TTAAGGACTGA AGTGATCGAA ATCGGATTGT TTTTTTTTTTT	e712	e763	e887	nullT11E
S466	ATCGACATCA TATCTTCCTGT TCAGTCCTTAA TTTTTTTTTT	e518	e345	e712	nullT10E
H467	ATGGATCTGAT AACTTGTGGA TGATGTCGAT TTTTTTTTTTT	e890	e864	e518	nullT11E
N468	TACCAAAGCA ACCTCTAAAGT ATCAGATCCAT TTTTTTTTTT	e891	e180	e890	nullT10E
H469	AGTAGACTGAA TACGTTGTGA TGCTTTGGTA TTTTTTTTTTT	e73	e282	e891	nullT11E
S470	TTACCACGAA AGATTCCTACA TTCAGTCTACT TTTTTTTTTT	e893	e374	e73	nullT10E
B471	TTTTTTTTTTT TACGGTGAAT TTCGTGGTAA TTTTTTTTTTT	nullT11S	e90	e893	nullT11E
S472	TCACACAGTT ATACTTAGCCT TTTTTTTTTTT TTTTTTTTTT	e716	e873	nullT11N	nullT10E
N473	TTAGGATGACA TTAGCTTGGT AACTGTGTGA TTTTTTTTTTT	e185	e359	e716	nullT11E
S474	ACAATCCGAT AAAATTTCCGT TGTCATCCTAA TTTTTTTTTT	e887	e66	e185	nullT10E
H475	ATTCGTAGAGA AGTTGATGCT ATCGGATTGT TTTTTTTTTTT	e897	e335	e887	nullT11E
S476	ATCTCATGCA ATAATCCCAGA TCTCTACGAAT TTTTTTTTTT	e898	e235	e897	nullT10E
N477	ATGGATCTGAT ATTTGCGTTT TGCATGAGAT TTTTTTTTTTT	e890	e815	e898	nullT11E
N478	ATCTCATGCA TCCTAAACTGA ATCAGATCCAT TTTTTTTTTT	e898	e810	e890	nullT10E
H479	TTTTTTTTTTT TGAGTGCATA TGCATGAGAT TTTTTTTTTTT	nullT11S	e75	e898	nullT11E
A480	ACAAGGGATAT TTTTTTTTTT TTTTTTTTTT AGAGCTATGAT	e901	nullT10W	nullT10N	e182
A481	TAGCATCACA TTTTTTTTTTT ATATCCCTTGT TTCGACACTT	e18	nullT11W	e901	e192
A482	TTAGTATCGGT TTTTTTTTTT AGGTAACGTT AGGAGCATATA	e9	nullT10W	e26	e537
A483	AGATGTCAGAT TTTTTTTTTT AAGAAGCAGT TAGGAGAAACT	e913	nullT10W	e11	e635
I484	TCACATACGT TTTTTTTTTTT ATCTGACATCT TTCCATTCGA	e7	nullT11W	e913	e916
A485	TTAGGGAATCT TTTTTTTTTT ACGTATGTGA ATTAGTCTGGA	e917	nullT10W	e7	e623
I486	TTTCCCTTGA TTTTTTTTTTT AGATTCCCTAA TAACACAGCA	e207	nullT11W	e917	e433
A487	AGGTTAAGAGA TTTTTTTTTT TCAAGGGAAA TATTGAGGCTA	e5	nullT10W	e207	e541
I488	AAGATACGAGA TTTTTTTTTT ACGTATGTGA AGAGTTAGGAA	e20	nullT10W	e7	e194
A489	TTAAGCTAGGT TTTTTTTTTT TAGAGCGAAA TCTAAGTGTGA	e929	nullT10W	e22	e173
A490	TTTCCCTTGA TTTTTTTTTTT ACCTAGCTTAA TACCATGCTT	e207	nullT11W	e929	e932
I491	TTTCGGATAGA TTTTTTTTTT TCAAGGGAAA AGTTGGTAGTA	e28	nullT10W	e207	e365
I492	AAGATACGAGA TTTTTTTTTT TCAAGGGAAA TTTTTAAGCGT	e20	nullT10W	e207	e70
I493	AACAAGTCCT TTTTTTTTTTT TCTCGTATCTT TACAAGCCAA	e939	nullT11W	e20	e847
A494	TACGGTATTGA TTTTTTTTTT AGGACTTGTT ACAGGAAGATA	e16	nullT10W	e939	e345
A495	TTTCGGATAGA TTTTTTTTTT TGTGATGCTA TGGAATAGTCA	e28	nullT10W	e18	e500
I496	AGGGATTCATA AAGTGTCGAA AAGGTTCAGT AAAAATGGACA	e792	e192	e110	e394

**Name**	**Sequence** ($5' \to 3'$)	**Gl. 1/E**	**Gl. 2/N**	**Gl. 3/W**	**Gl. 4/S**
I497	TTTACTGCCT TCCCTATGAAA TATGAATCCCT TCATCACCAT	e440	e21	e792	e298
I498	ACATATGGAGT AAGAAAGGCT AGGCAGTAAA AGGAGCATATA	e581	e27	e440	e537
A499	TGCAGTTTTTA AGGAACGATT ATTGAAGGGT ATAGACAGGAA	e48	e12	e71	e57
A500	TTTTGTTCAGT TCGAATGGAA TGTCGGTAAA TTTGTCAATGT	e483	e916	e681	e963
I501	TTTTGATTCGT TGCTGTGTTA TGTTCGTGTA ATTACGAGAGA	e332	e433	e669	e431
A502	TTGAGAGTAGT TGGATCGATT AAGAGCAGTT TTTCATTTGGT	e238	e8	e587	e329
A503	TCGTTTAGAGA TGGGTTCTTT AAGGAATCGA TGTAGGAATCT	e217	e23	e240	e374
A504	TTCTATGTGGA AAGCATGGTA AAAGAGGGTT AGAAAGGAAGA	e213	e932	e155	e729
A505	TTTTGTTCAGT TAGGCAATGA TGTAGGCAAT AGTAATCAGGA	e483	e196	e411	e983
A506	AGTAGTGACAT TTGGCTTGTA ACTGGGAATT TTAAGGAAGGT	e589	e847	e485	e806
A507	ACAGGTTAGAT TTGTGCTAGT TTTGAGTGGA AGATAGCGTAT	e544	e19	e591	e598
A508	TTTTTTTTTTT TGTGTTGAGT TGACGTTGTA TAATACGAGGA	nullT11S	e30	e546	e74
A509	AGGATGTACTT AGGAGGTAGA TTTTTTTTTT AGTAGGAAGAA	e829	e229	nullT10N	e122
A510	TGAGGACTATT ATGGTGATGA TAAAGCTGGT ACGGAAATTTT	e76	e298	e831	e66
A511	AGCAGGTATTA TCAATTGGGA AAAGAAGGGT AATGTGGAAAA	e1007	e682	e86	e141
I512	ACCAGCATAA ACATTGACAAA TAATACCTGCT TCGACTTCTT	e624	e963	e1007	e139
I513	AGTCGATGATA TTTTCTGGGA TTATGCTGGT TAATACGAGGA	e475	e670	e624	e74
A514	TAAATGGGAGA ATGTAGCAGT TAGAATGCGA TATAGACGTGT	e418	e241	e628	e255
I515	TATGGAGCTAT ATGTGACAGT TGAAGCAGAT TATTCGAGGTA	e756	e220	e420	e834
A516	TTATTTTGCGT ATGAAGACGA ATGTGGATCA TGAAAGAGGTA	e1027	e412	e758	e357
I517	ACCACTGATT TCCTGATTACT ACGCAAAATAA TTAACAGCCA	e814	e983	e1027	e363
A518	AACAGAGAGAT AGAGCGTTAT AATCAGTGGT AAAGAGAGGAA	e833	e486	e814	e222
A519	AGAGATGACTT AAATGAGGCT ATGGTCGAAT AATCGGAAAAA	e275	e347	e835	e1036
I520	ATTAATTGCGA AGAACGGATT AAAGAGGGTT TACATGATGGA	e516	e547	e155	e10
I521	TTTTTTTTTT TCCTCGTATTA TCGCAATTAAT ATTCCAACCT	nullT10S	e74	e516	e509
A522	TTGAGGGATAT TGTGTCATGA AAGGGTCAAT AGTACGAGTAT	e61	e791	e95	e1049
I523	TGGAAGAGTAT AAGAAGTCGA TGATGAAGCT ATTGTTTGACA	e101	e139	e146	e38
A524	TAGAGATGCTT TTCGGAGATT AGACTGGTTT TAAAACGTGTT	e1060	e478	e103	e660
I525	TCGATTCCTT ATACGCTATCT AAGCATCTCTA ACTGTCACAT	e240	e598	e1060	e220
I526	TGTTTGAACTT ATGGGAACTT AAGGAATCGA TATATGACGGA	e133	e629	e240	e210
A527	AGGAGTGTATT TTAGGCAAGT AGGTGAATGT ATTACGAGAGA	e861	e237	e538	e431
A528	TTTTGATTCGT TGGTATGCAT ACGGAGAAAT AGTTGGTAGTA	e332	e124	e863	e365
A529	AGGAAGATTCT TGGCTGTTAA TGAGACGAAA AAATTGGAACA	e1076	e363	e428	e102
I530	ACTTTCTCGT TTCCTCTCTTT AGAATCTTCCT TCCCAGAAAA	e493	e222	e1076	e670
A531	ACATATGGAGT ATGTATGGCT ACGAGAAAGT TGGTGAGATTA	e581	e836	e493	e14
I532	ATTCCATCGT TTTTTCCGATT ACTCCATATGT TATCGCTCAA	e1082	e1036	e581	e527
A533	AGTGTTTTGAA ATGTGGTCTT ACGATGGAAT TGTGACAGATA	e454	e645	e1082	e778
A534	TTTTTTTTTTT AGGTTGGAAT AGCAAGAGAA ATTGAAGCAAA	nullT11S	e509	e734	e274
A535	TTCGGATAGAA AAGGGTGAAT TTTTTTTTTT AGGAGTTTACT	e651	e152	nullT10N	e17
A536	AGGTACGATTA TGGCTGTAAT AACTGAAGGT AGAGAAGATGT	e1093	e96	e653	e619
I537	AGCTTCATCA ATACTCGTACT TAATCGTACCT AGCTCTCATT	e146	e1049	e1093	e273
I538	AGAACTAAGGA TGGCTGTTAA TGATGAAGCT AGCAAAAGAAA	e136	e363	e146	e177
A539	TGGCTATTAGA TGGTTTGCTA TTGTTGGCTA TTTTCGATTGT	e65	e171	e138	e845
A540	AGGTAATGGTA TGGCAGTAAT AATGTGAGGT ACAGGAAGATA	e704	e104	e67	e345
A541	ATCTTGGAGTA ATGTGACAGT AGCTGAAGTT AGGAAATAGGA	e112	e220	e99	e757
A542	TTCGAAATGTT TGTCGTTAGT AATGTCAGGT AATGTGGAAAA	e557	e156	e256	e141
A543	AGGACTAATGT AACTTGTGGA TGTTCAGGTT TCTTAGAAGGT	e659	e864	e559	e218
A544	AATGCAGATTT TTGTGGCTTA TCTTGTTGGT ATATGGTAGCT	e328	e429	e661	e89
A545	TGGATGATACT TTTTCTGGGA TTTGGAGTGT TTAGATACGGA	e41	e670	e330	e553
A546	TAAAATTGGCA ATCGAGTGTT TCTTGGGTTT AGGTTAGACAT	e319	e735	e747	e341
A547	TGGAAGAGTAT ATGGGAACTT TAATGGCTGT TTAGTAGGTGT	e101	e629	e46	e29
A548	AATAGGGTACA AATGAGAGCT TAAAGGTGCT AATTTTTCGGT	e93	e273	e665	e202
A549	TTGGTTTGAAT AAGAAGTCGA TGCGTTTTTT ATGTAGTAGCA	e422	e139	e399	e259
A550	AGGGATTTAGT TGAAATGGGT AATGTGAGGT TGAAGTAAGGA	e389	e68	e67	e271
A551	AGGTGAAGTAT TTGGCAGTTA TATGGTTCGT AAGGTAGTCAT	e784	e707	e391	e504
A552	AGTGGATCTTA AGGTGTTTCT TTAGAGCGAA TTAGTTCAGGA	e777	e257	e309	e263
A553	TAGTCAGTTGA AGTCGGATTT ACGGTTGTTA TGAGAGAAAGA	e262	e560	e554	e733
A554	ACAAGGATAGA AAGGAGGTTT AAGGTTCAGT TGGAGACTAAT	e116	e179	e110	e296
A555	TCGGAATTAGA AAGGGACAAT TTGGTGACTT TAATACGAGGA	e597	e331	e118	e74

Name	Sequence (5′ → 3′)	Gl. 1/E	Gl. 2/N	Gl. 3/W	Gl. 4/S
A556	AGAACAGAGTA ATTGCGTTTT AAGGTTGTCT AGTATTACGGA	e234	e44	e599	e53
A557	AGACGGTAATA TGGACTTTGT TGGATGTCAA TAGTTGTGAGA	e606	e808	e236	e451
A558	TTTTTTTTTTT AGCTGATTGA TTGGACGTAA ATTACAGAGGT	nullT11S	e321	e608	e796
A559	AGTAGTGAAGT ACGAGTTGAA TTTTTTTTTT ACTTGGTAGAT	e56	e47	nullT10N	e1184
A560	TGTTGTTGATT TGAACTGGAA ATGCGTAGAA TAGGGTAACAT	e246	e666	e58	e117
A561	AGGGTTAGAAT TGTGAAGGAA AGTTCGGATT AAAAATGGACA	e520	e400	e248	e394
I562	TGATCCACAT TGCTACTACAT ATTCTAACCCT AGTTCACACA	e758	e259	e520	e290
I563	AGGTATCGTAT TTAGCTTGGT ATGTGGATCA ACAGGAAGATA	e315	e359	e758	e345
A564	TAAGAGTGTGA TGTAAGTGCT TGATGATCGT TCTGGGATTAT	e548	e310	e313	e235
I565	TAGAAGGCATA TACGTTGTGA TGAGACGAAA ACAGGAAGATA	e307	e282	e428	e345
A566	TGTAGAGGATT AGTGGAATGT TTAGAGCGAA ACGGAAATTTT	e655	e780	e309	e66
A567	AGTCTAAAGGA ACGTGAAGAA TAGCAGGTTT ATTAGTGGGTA	e532	e119	e342	e49
I568	TGGATAGCTTA TTAGGCAAGT ATGGTGTCAA AATCGGAAAAA	e311	e237	e82	e1036
A569	TATGGAGCTAT TTGAGATCGT TTGGGATGAA AAAAGTTGTGT	e756	e551	e497	e206
I570	AAGCTCTTCA ATCTACCAAGT TTTTTTTTTTT TCACCGTTAT	e1230	e1184	nullT11N	e323
A571	AGTCTAAAGGT TTGTGGTGAT TGAAGAGCTT TTTTGCATGAT	e737	e59	e1230	e1233
A572	AAGAGGTAGAA ATGATGAGCA AGTTCGTGAA ATAGTCTTGGA	e1236	e64	e739	e1237
I573	AACCATGTCT TGTCCATTTTT TTCTACCTCTT ACCCAATGAT	e1238	e394	e1236	e1239
A574	ACGTTGATTTT TGTGTGAACT AGACATGGTT TGGAGTAAGAT	e565	e290	e1238	e186
A575	ATTCGTAGAGA TGTGTCTTGA TTAGCGAGTT ATAGGACAAGT	e897	e318	e567	e650
A576	TTAAGGACTGA TTGAGATCGT TGCATGAGAT TTTTTTTTTTT	e712	e551	e898	nullT11E
I577	TTAGGATGACA TGTAAGTGCT TGATGTCGAT TTTTTTTTTTT	e185	e310	e518	nullT11E
A578	ATGGTTTTGTT AGAGCGTAAT AGTAGCGAAT TTTTTTTTTTT	e356	e343	e714	nullT11E
A579	AGAGTGATGAT TTGTGCTAGT TGTGATACGT ATTGATGCAAA	e463	e19	e358	e1257
A580	AGAAGTACAGA TGTTGGGTAA AGGAACAAGT AGGAAAGACTA	e1260	e83	e54	e280
I581	ACCCTTTGAA TTTTTCCGATT TCTGTACTTCT ACGATCTCAA	e1262	e1036	e1260	e551
A582	ACTGAGGATAA ATGGTTGGAT TTCAAAGGGT AAAGGCTAGTA	e250	e498	e1262	e288
A583	TTTTTTTTTTT AGTCGTTTGA TCTTTGGGAA AGAGTTATGGA	nullT11S	e826	e187	e462
I584	TTCGTACACA ATCATGCAAAA TTATCCTCAGT TATCTGCACA	e63	e1233	e250	e1272
A585	TGCGATATTTT TACGTTGAGT TGTGTACGAA TTCGGTATAGA	e33	e740	e63	e243
I586	TACACGAACA TCCAAGACTAT AAAATATCGCA AAGCCATCTT	e669	e1237	e33	e1276
A587	ATTTGCTGATT ATCATTGGGT TGTTCGTGTA TTTACTGGGTA	e573	e1239	e669	e406
A588	AGTTGTAGTCT AGCAGAAGAA TTGAGCTGAA AATGTCTAGGA	e695	e568	e575	e320
A589	AGGGATGTAAT TTAGCTTGGT TTTTTTTTTT TTGTTGCATAA	e88	e359	nullT10N	e464
I590	TTACACCGAT TTTGCATCAAT ATTACATCCCT TTGCACCTTA	e31	e1257	e88	e404
A591	TTAGGTAGCAT AAAGGTCGAT ATCGGTGTAA TGAGTAGAACA	e324	e466	e31	e578
I592	TTTTGATTCGT TTGAGATCGT TCGAAGTTGT AGGTTCTAGTT	e332	e551	e326	e145
A593	TAGAGGAAAGT TGGTTCTTGA TCGTTATGGT TACATGGAAGA	e153	e253	e334	e447
I594	TTGACACCAT TGTTCACTTTT TTTTTTTTTTT TCACCAAACT	e82	e370	nullT11N	e32
A595	TGTATGAAGGA TGTGCAGATA ATGGTGTCAA TAAGAAGGTCA	e287	e1272	e82	e231
A596	AGAGAATGAGT AAGATGGCTT TCTTGTGTGT TATGACTGTGA	e450	e1276	e289	e378
A597	ATGGATTAGGA AGGCTGAAAT TCTTTGGGAA ATGATTGCAAT	e479	e576	e187	e198
A598	TTTTTTTTTTT TTGGGTTTCT AAGATGCTGA ACTAAAGGAGT	nullT11S	e697	e481	e1313
A599	AACTGATAGGA TTGGTGGTAA TTATGCTGGT ACAATGGAAAA	e708	e327	e624	e239
I600	TGGATGATACT AGTTGATGCT TGTGTACGAA TATACGAAGGT	e41	e335	e63	e817
I601	TTTTTTTTTTT AGGTTGGAAT ACGAGAAAGT ATTTTGCAGAT	nullT11S	e509	e493	e841
I602	AGAGATGACTT AGTTTGGTGA TTTTTTTTTT TATCGGAGAAA	e275	e32	nullT10N	e753
I603	TTGGTTTGAAT TGTGTGAACT AAATGGGTGA ACAATTGGAAA	e422	e290	e277	e480
A604	TTGACATTGTT ATGATGGTGT TTATGGACGT ATGAGTTAGGT	e1341	e204	e244	e476
I605	TTTTTTTTTT ACTCCTTTAGT AACAATGTCAA TAACACCCAA	nullT10S	e1313	e1341	e150
A606	ATGTGAGTAGT TTGAGCAGAA TTAGTCGTGT TACATGATGGA	e283	e408	e436	e10
A607	AGGATCTGATT AAATGAGGCT TGTGCGATAT ATGGTAGATCA	e1352	e347	e285	e742
I608	ACCCTTTGAT ACCTTCGTATA AATCAGATCCT ACTAGCACAA	e726	e817	e1352	e19
A609	ATCTTAGGGAA AAGACGGAAA TCTTTGGGAA TAGAGGAACAT	e857	e265	e187	e789
A610	AGGAGTTACTT TTAGCGATGT TGACTGGAAA AAAGCATTGAT	e381	e425	e859	e390
A611	AGGGTTAGAAT TAGTGGCAAT TAGAAAGCGA AGACTTAGGAA	e520	e87	e383	e126
A612	AAGGGATTAGT AGAGTGCTTT ATGACATGGT AAAGAGAGGAA	e37	e506	e782	e222
A613	TAGAAGGCATA TGTCGAGATT TTCGGAGTTT AAAATTAGCGT	e307	e286	e39	e81
A614	TGGTCATAGAT TTGTGCTAGT TGTACGGAAT AGGTTCTAGTT	e1384	e19	e771	e145

Name	Sequence (5′ → 3′)	Gl. 1/E	Gl. 2/N	Gl. 3/W	Gl. 4/S
I615	ATGTCCACTT TCTTCCTTTCT ATCTATGACCA ATGCCACTTA	e91	e729	e1384	e1387
A616	TTTTTTTTTTT TACGGTGAAT AAGTGGACAT AGAATATGGGT	nullT11S	e90	e91	e713
A617	TTATCTGTGGA TAGTGAACGT TTTTTTTTTT AAAAGTGAACA	e125	e843	nullT10N	e370
A618	TAGCATGAGTA AGTGATCGAA TATTGGTCGT AGCTAGTGTTA	e434	e763	e648	e34
A619	TGGGAATATCT TTTTTTTTTT TATTGCTGGT TTAGAGTGACA	e1	nullT10W	e203	e631
A620	AGTTTGTTTGT TTTTTTTTTT TCGAGAAGTT ATGTAGTAGCA	e209	nullT10W	e3	e259
A621	AGATGTGATCT TTTTTTTTTT TGGGAAGTTT TATATGACGGA	e197	nullT10W	e211	e210
I622	TGTAGATGAGT TGGTTCTTGA AATGTGAGGT AAAAATGGACA	e266	e253	e67	e394
A623	AGAATTAGGGA TGTGGTACAA TGGTTACGAT TCTAAGTGTGA	e108	e40	e268	e173
A624	TGGAAGAGTAT TAAGTGGCAT ATCTGGGTTT TTTAAATGCGA	e101	e1387	e174	e190
A625	TTGAGGGATAT AGTTGATGCT AAGGGAACAA TCAAGAAGGTA	e61	e335	e416	e42
A626	ACGAGATAGAA ATTGGGTCAT TGTGTACGAA ACAATTGGAAA	e140	e224	e63	e480
I627	AGGTATCGTAT TGTCGTTAGT AGAAGACGTT TTTCATTTGGT	e315	e156	e142	e329
A628	TGTAGAGGATT TGTTGGTCTT AATGTCAGGT TGATTAGGAGT	e655	e380	e256	e414
A629	ATGTAGGGTAA TTTGGGTGAT AAATTGCGTT AGGATATAGCA	e303	e4	e534	e94
A630	TAGTGGTTGTA AATCGGAGTT TTGTGTAGCA ATAGTGAGGAA	e438	e200	e799	e325
A631	AAAAGGGTCTA ATGCTGAGAT AGGCAGTAAA AGAGTTAGGAA	e614	e269	e440	e194
A632	AATAGGGTACA TGTTACGAGT AGGTGCTTAA TCTGGGATTAT	e93	e111	e616	e235
A633	TGTAGATGAGT TTTGGGTGAT TCGAAGTTGT TAGGTTTCAGA	e266	e4	e326	e853
A634	TTTTTTTTTTT TTTGGGTTCT AATGACGGTA AGAATCAGGAT	nullT11S	e751	e460	e368
A635	ATGGGATTAGA TTCGGAAGAT TTTTTTTTTT AGAGTGGATAA	e69	e417	nullT10N	e798
A636	TATGACAGAGT ATGATGAGCA ATTGAAGGGT ATATAGAGGCA	e524	e64	e71	e427
A637	ACAGTAGGATT TTGGATTGGA TGTTGATCGA TTTAGGACGTA	e157	e143	e526	e1492
A638	TAGCTAATGGA TCGGTTATGT ATGAGGTGTT ATATAGGACGT	e458	e457	e375	e496
A639	TTGGGATATCT ATGGAAGTGT AATGACGGTA AGGATATAGCA	e487	e658	e460	e94
A640	AAGTACTGAGA TAAGGCATGA TGTTAGCGAT ATTGTTTGACA	e121	e294	e867	e38
A641	TCGAGAGATTA TTTGGATGGT TTATGGCAGT AAGAGAGTGTA	e405	e306	e123	e304
A642	AGAGTTTAGGA ACGTGTATGT AAAGATGGCT ATCAAGGTAGA	e168	e441	e371	e1516
A643	ACTTGTAGGAT AGCGTTGATA TTTGGGTACA AGGTTTAGGTA	e279	e36	e293	e639
A644	AGTCGATGATA TGTTGGAACT TGTGCAGTTA TAAAAGGCAAA	e475	e502	e281	e6
I645	TTTTTTTTTT ATCCTGATTCT AGTTTACTCCT AGAAACCCAA	nullT10S	e368	e364	e697
A646	AGGGTTCTAAT ACTGGTGATT TGCGAGAATA ATAGCAGGATA	e161	e72	e827	e423
A647	AGTGTGAGATA TTGAGCGATA AGTGAGTTGT TTAGAGTGTCT	e1536	e527	e473	e746
I648	ACGACACATA TACGTCCTAAA TATCTCACACT TGCCATATCA	e1538	e1492	e1536	e376
A649	TTATCTGTGGA TGATATGGCA TATGTGTCGT AGATCAGTGTA	e125	e376	e1538	e169
A650	TAGATGTACGT TTGGATTGGA TCAAGTTGGT ATGGGATCATA	e630	e143	e542	e149
A651	TAAAATTGGCA TACGTTGTGA TCGTGGTATT AGAGTAGTGTT	e319	e282	e632	e226
A652	TGGTAAGTACT TGGTATGCAT TTTGTAGGCT TATACGAAGGT	e348	e124	e114	e817
A653	TAGGAACAGAA TTGAGCAGAA TTGGAACGTA ATGACAGTAGT	e344	e408	e127	e785
I654	AACATACCGA TCTACCTTGAT ATTAGAACCCT ATTCATCCGT	e1562	e1516	e161	e1563
A655	TAGGAACAGAA TAAGGCATGA TCGGTATGTT AGCAAAGAAAT	e344	e294	e1562	e308
I656	TGCGATATTTT TACGTTGTGA TTATGCTGGT TACATGATGGA	e33	e282	e624	e10
A657	TGCGATATTTT ATTTGCGTTT TCAGGGAAAA TTAAAGAGGGA	e33	e815	e35	e566
I658	AACTCCCTTT TACTACCAACT AAAATATCGCA TACCCACAAA	e1574	e365	e33	e1575
A659	TTTTTTTTTTT TTGGGTTTCT AAAGGGAGTT AGTGAACAAAA	nullT11S	e697	e1574	e165
A660	TGAGTATCTGT AGTATGCTGT TTTTTTTTTT TTCATGTTGTT	e467	e828	nullT10N	e2
A661	AGAATTAGGGA TCGGTTATGT TGGTGCATAT ATTAGTGGGTA	e108	e457	e223	e49
I662	TTCTATGTGGA TGATATGGCA TCGAGAAGTT AGAACTATGGT	e213	e376	e3	e154
A663	TATCGGAAGTA TACGTTGGAT AATGGTGGAT TTTTTAAGCGT	e176	e543	e215	e70
A664	AGGAAGGAATA TGGACAGTTT TAATGGAGCA ATAGGTGACAT	e270	e515	e178	e1594
A665	TTTGACTGTTT TCTGTTTGGT TCGTGAAAGA ATTGTAGGACT	e844	e449	e272	e676
I666	AGTAGATACGT TGAAAGTCGA ATGCGTAGAA TCAGTTTAGGA	e503	e351	e58	e810
I667	ACAGTAGGATT AAATGAGGCT AATCAGTGGT ACTTTAGAGGT	e157	e347	e814	e180
A668	AGAAGTCAGAT ACGGATGAAT AGCTGTGATT TCAGTTTAGGA	e352	e1563	e159	e810
A669	AGGTAAGAGAA AGTGCAGAAT ACGTGTGTAT ACTAGATGGAA	e820	e686	e354	e647
A670	ATGAAAATGCT AGCGTTGATA TGATTGAGCT AAAATGCAAGA	e610	e36	e822	e316
A671	ACATATGGAGT TTTGTGGGTA TAGCAGGTTT TTTTGGTTCAA	e581	e1575	e342	e113
I672	TTTTTTTTTT TTTTGTTCACT ACTCCATATGT ATTCACCGTA	nullT10S	e165	e581	e90
A673	TGAGGACTATT TCGAGTGAAT ATCGGTGTAA TGAGGTATGAT	e76	e396	e31	e45

Name	Sequence ($5' \rightarrow 3'$)	Gl. 1/E	Gl. 2/N	Gl. 3/W	Gl. 4/S
A674	AGATGGAGATT TGTCTGTGTT TGGATATGCA TATAGACGTGT	e852	e776	e78	e255
A675	TGGTAGTAACT AGAGCAATGA AAGCGAGTTA AGGTCTTGATA	e97	e216	e854	e821
I676	ATCGACATCA ATGTCACCTAT TCCTATCAGTT TTTTTTTTTT	e518	e1594	e708	nullT10E
A677	AGTTCTGAGAT AATGAGAGCT TGATGTCGAT TTTTTTTTTTT	e717	e273	e518	nullT11E
A678	ATGGATCTGAT TTGGCTTGTA ATGGATGTGT TTTTTTTTTTT	e890	e847	e718	nullT11E
A679	AGGTATGGAAT TTTGATCGGA TGCTTTGGTA TTTTTTTTTTT	e837	e160	e891	nullT11E
I680	TTGGGATATCT AGGTTAACGT AAAGATGGCT TGAAAGAGGTA	e487	e445	e371	e357
A681	TAGGAAAGTCA AGGTTTGTGA TTGGCAGTAT TTATGTTTGCA	e552	e613	e489	e410
I682	TTTTTTTTTTT TACGGTGAAT ACGGTTGTTA AGAATCAGGAT	nullT11S	e90	e554	e368
I683	ACATATGGAGT AGTTTGGTGA TTTTTTTTTT AACTATGAGGA	e581	e32	nullT10N	e137
A684	ATGGATGTCTA TGGTTGCTAA TCTTGGGTTT TAAGTGACTGA	e299	e79	e747	e725
I685	TGAGTATCTGT TACGTTGGAT TCTGTTGGAA TGGAGTAAGAT	e467	e543	e301	e186
A686	AGTAGACTGAA TAAGCGTGAT TAGATGCGAA AGTGAGTAAGA	e73	e100	e163	e594
A687	TAACTGGAAGA AGCTGAGAAT TTTTTTTTTT TCGAGAGATAA	e393	e840	nullT10N	e62
A688	AGTAATCTGGT ATGGACTTGT TCATGGATGT TAATAGTCGGA	e401	e694	e151	e251
A689	AGTGTTTTGAA TGTGACAAGA AAGTCGTGAT AGAGTTGAGTA	e454	e490	e403	e643
A690	TATATGGTCGT ATTGCGTTTT TGTTCTGTGT ATTATCGGAGA	e193	e44	e166	e627
A691	AGACTAATGGA ATGGGTTGAA TCATGGGTTT ATAGAGTTGGA	e528	e302	e514	e98
A692	AGAAGGATGAT TTGTGCTAGT AAGTGGTGAA TGGGTACTTTA	e638	e19	e510	e688
I693	TTTTTTTTTTT TGAGTGCATA TAGCAGGTTT TGAGGTATGAT	nullT11S	e75	e342	e45
A694	ATGTAGGACTT TCGAGTGAAT ATGTCGGTAA TTTCATAGGGA	e295	e396	e106	e21
A695	ACAAGGATAGA TAAGGTGCAA TGTGACATGT TTAAGGGAGAA	e116	e404	e297	e549
I696	TGGCTTTTTTA TCGGTTATGT AAAGTGGTCT AAATTGGAACA	e512	e457	e620	e102
A697	TTTTTTTTTTT AGATGACGTT TCATGGGTTT TAGGAAGACAA	nullT11S	e415	e514	e60
A698	TGGAGATACAT AACAGGTGAA ATCAAAGGGT AGCTAGTGTTA	e172	e641	e726	e34
I699	TTTTTTTTTT ATCATACCTCA ATGTATCTCCA TCCAATCACA	nullT10S	e45	e172	e1737
A700	TGCAGTTTTTA TTCGATGGAT TTTTTTTTTT TCGAGAGATAA	e48	e107	nullT10N	e62
A701	TGGCTTTTTTA ATGGTGATGA TAGCGATGAA TAAAATGTGCA	e512	e298	e50	e602
A702	ATACTGGATGA TTGTAGCGAT TAATGGCTGT AGATTATCGGA	e129	e494	e46	e214
I703	AGGGATGTAAT TGGACAGTTT TTTCGAGGTT ATAGGACATGA	e88	e515	e131	e525
I704	ACCCTCAAAA TAGCCTCAATA TCCTTTAGACT TTACCACCAA	e1757	e541	e532	e327
A705	AGAACTAAGGA AAGCAGATGT TTTTGAGGGT TATTCGAGGTA	e136	e128	e1757	e834
A706	TTTTTTTTTTT TGTGATTGGA TAGATGCGAA AGGTCATTAGT	nullT11S	e1737	e163	e736
A707	TTTAGTGGAGA AGGTTTGACT AAGTGGACAT TAGGGTAACAT	e646	e51	e91	e117
A708	AACGTGAAATT AACAGGTTGA TATTGGTCGT TAGAGAATCGA	e258	e596	e648	e109
A709	AGGGATTCATA AAGAAAGGCT AAGGCTGATT AAGAAAAGCAA	e792	e27	e260	e590
A710	TTTTTTTTTTT TACGGTGAAT AGCAAGAGAA ATCAGGATGTA	nullT11S	e90	e734	e556
A711	ACTATGTGAGT TTGGCTATGA TTTTTTTTTT AGAGTAAGGTT	e430	e167	nullT10N	e858
I712	ACTTACTGCA TACCTCGAATA AATCCTACTGT TCTTCAGCAT	e1789	e834	e157	e1790
A713	AGTGATTAGGA ATTGGGTCAT TGCAGTAAGT TTTCATTTGGT	e144	e224	e1789	e329
A714	TTTTTGTCTGT TTTGATCGGA TGATGAAGCT AGATCTGAGTT	e764	e160	e146	e419
A715	ATTAATTGCGA ATTGGATCGA TTTTTTTTTT AGGCTAAGTAT	e516	e92	nullT10N	e873
A716	AGGTATCGTAT AAGGTACGTT TGATGTCGAT TTCTAGGAAGT	e315	e649	e518	e1803
A717	AGTAGACTGAA TGCTGTGTTA ATCGGATTGT TTTTTTTTTTT	e73	e433	e887	nullT11E
A718	TTGCGATAAAA ATGCTGAAGA TTCGTGGTAA TTTTTTTTTTT	e1816	e1790	e893	nullT11E
I719	TTACCACGAA ACCAAATGAAA TTTTATCGCAA TTTTTTTTTT	e893	e329	e1816	nullT10E
A720	AAAGAGGAGAT AGTAGTGCAT TTCGTGGTAA TTTTTTTTTTT	e719	e147	e893	nullT11E
A721	TTTTTTTTTTT TGCAGAAGAT TTTCGATGGA TTTTTTTTTTT	nullT11S	e135	e720	nullT11E
A722	AGGGAAGTATT TGTTGTCAGA AACTGTGTGA TTTTTTTTTTT	e1821	e519	e716	nullT11E
A723	TGCCTTACAA ACTTCCTAGAA AATACTTCCCT TTTTTTTTTT	e1822	e1803	e1821	nullT10E
A724	TGAGGATGTTA AGCAGGATTT TTGTAAGGCA TTTTTTTTTTT	e715	e339	e1822	nullT11E
A725	ATCACCGAAT TTCTCCCTTAA TGTCATCCTAA TTTTTTTTTT	e1826	e549	e185	nullT10E
A726	TTTTTTTTTTT AGTTGATCGT ATTCGGTGAT TTTTTTTTTTT	nullT11S	e603	e1826	nullT11E
M727	AGATGTGATCT TTTTTTTTTT TTTTTTTTTT TGGGTGTATAT	e197	nullT10W	nullT10N	e652
M728	AAGATACGAGA TTTTTTTTTT AATGTGAGGT ATTGTTTGACA	e20	nullT10W	e67	e38
M729	AGGTTAAGAGA TTTTTTTTTT AGGACTTGTT ATAGAGTTGGA	e5	nullT10W	e939	e98
M730	TACGGTATTGA TTTTTTTTTT ACGTATGTGA TAAAATGTGCA	e16	nullT10W	e7	e602
M731	AGTTTGTTTGT TTTTTTTTTT TGTGATGCTA ATTACGAGAGA	e209	nullT10W	e18	e431
M732	TTAGGGAATCT TTTTTTTTTT TGGGAAGTTT ATAGCAGGATA	e917	nullT10W	e211	e423

Name	Sequence (5′ → 3′)	Gl. 1/E	Gl. 2/N	Gl. 3/W	Gl. 4/S
M733	AGATGTCAGAT TTTTTTTTTT TAGAGCGAAA AGAGTAGTGTT	e913	nullT10W	e22	e226
M734	TGGGAATATCT TTTTTTTTTT AGGTAACGTT AGGTTAGACAT	e1	nullT10W	e26	e341
M735	TTTCGGATAGA TTTTTTTTTT TCGAGAAGTT TAATAGTCGGA	e28	nullT10W	e3	e251
M736	TGGCTATTAGA AATCGGAGTT ACGAGATTGA AGTGAACAAAA	e65	e200	e698	e165
M737	TTTAGTGGAGA TGGATCGATT TTGGAACGTA ATAGACAGGAA	e646	e8	e127	e57
M738	TGAGTATCTGT TTGTGGTGAT TAATGGCTGT AGAACTATGGT	e467	e59	e46	e154
M739	TGGTAGTAACT TGCTGTGTTA TGGTGCATAT AATTTTTCGGT	e97	e433	e223	e202
M740	AGGAAGGAATA TGGGTTCTTT AGCTGAAGTT AAGGTAGTCAT	e270	e23	e99	e504
M741	AGAGTTTAGGA TCGAATGGAA TCGTGAAAGA ACAGGAAGATA	e168	e916	e272	e345
M742	TATGGAGTTCT AAGAAAGGCT TTTGGGTACA ATATAGGACGT	e385	e27	e293	e496
M743	TAGGAACAGAA TTTGGGTGAT ATAACGTGGA TGTGACAGATA	e344	e4	e387	e778
M744	AGGACTAATGT TAGGCAATGA AGCTGTGATT AGAGAAGATGT	e659	e196	e159	e619
M745	TTTTTTTTTTT TGTGTTGAGT TCTTGTTGGT ATGCAAAGATT	nullT11S	e30	e661	e120
M746	AATAGGGTACA TGAAATGGGT ATGGTTGTCA TGGTATTCAGA	e93	e68	e195	e85
M747	AAGTTAGGGTA AAGCAGATGT AAGGGTCAAT TAATACGAGGA	e84	e128	e95	e74
M748	ACTTGTAGGAT AAGGTACGTT AAAGAAGGGT ATATAGAGGCA	e279	e649	e86	e427
M749	AGATGGAGATT TTCGGAGATT TGTGCAGTTA TATACGAAGGT	e852	e478	e281	e817
M750	TGTTGTTGATT TCGAGTGAAT AAGCGAGTTA AGAAAGGAAGA	e246	e396	e854	e729
M751	TAAGAGTGTGA TAAGCGTGAT AGTTCGGATT TAAGAAGGTCA	e548	e100	e248	e231
M752	ACGTTGATTTT AATGAGAGCT TGAGACGAAA TATTGAGGCTA	e565	e273	e428	e541
M753	TTATCTGTGGA TAAGGCATGA TTAGCGAGTT TTCTGTAGTGA	e125	e294	e567	e398
M754	ATTTGCTGATT AGTTCTGGTT TCAAGTTGGT AAGAAAAGCAA	e573	e388	e542	e590
M755	TGGACAAATTT AAGGAGGTTT TAAAGGTGCT AGATCTGAGTT	e148	e179	e665	e419
M756	TGGAAGAGTAT TGGCTGTAAT TTTGTAGGCT TCAAGAAGGTA	e101	e96	e114	e42
M757	AGGGTTCTAAT TAGTGGCAAT AGACTGGTTT TTTAAATGCGA	e161	e87	e103	e190
M758	AGGATCTGATT TACGTTGTGA AGTGAGTTGT TTAAGGGAGAA	e1352	e282	e473	e549
M759	TGGTCATAGAT TACGTTGGAT ATCAAAGGGT AGTATTACGGA	e1384	e543	e726	e53
M760	AAGTTAGGGTA AGCAGAAGAA AAGAGCAGTT AGAAGTAGACA	e84	e568	e587	e386
M761	AGAGTTTAGGA TGGACTTTGT TGACGTTGTA AAAGGCTAGTA	e168	e808	e546	e288
M762	AGAGTGATGAT TGAACTGGAA TGATGAAGCT ACAATGGAAAA	e463	e666	e146	e239
M763	TTTTTTTTTTT TTGGGTGTTA AGGAACAAGT TGAGGATTACA	nullT11S	e150	e54	e2014
M764	TGTTTGAACTT TAGTGAACGT TTTTTTTTTT TAGTTAGTCGT	e133	e843	nullT10N	e276
M765	ATGGGATTAGA AGCAAGGATT AGGTGAATGT TAGAGAATCGA	e69	e115	e538	e109
M766	TCGTAGTATGT TGGCAGTAAT ATTGAAGGGT AAATTGGAACA	e618	e104	e71	e102
M767	TAGATGTACGT TAAGTGGCAT ATGGTGTCAA AATGTCTAGGA	e630	e1387	e82	e320
M768	TGGAGATACAT TGTTGGAACT TCGTGGTATT ATTGATGCAAA	e172	e502	e632	e1257
M769	ACTATGTGAGT TTGAGATCGT ATCTGGGTTT ATAGTCTTGGA	e430	e551	e174	e1237
M770	TTTTGATTCGT TTAGCTTGGT TGTTAGCGAT ATGGTAGATCA	e332	e359	e867	e742
M771	ATGTGAGTAGT TGGTTTGCTA TCGTTATGGT AGGAAATAGGA	e283	e171	e334	e757
M772	TGAGATTGGTA AAAGGTCGAT TGTGCGATAT AAGAGAGTGTA	e2059	e466	e285	e304
M773	TTTTTTTTTT TGTAATCCTCA TACCAATCTCA ATCACATCGT	nullT10S	e2014	e2059	e2061
M774	ATACTGGATGA ACTGGTGATT AGGAACAAGT AAAAATGGACA	e129	e72	e54	e394
M775	ATTGCAATGAT AGCTTGTGTA TTTCGAGGTT TGAGGTATGAT	e700	e621	e131	e45
M776	ATGGTTTTGTT AACAGGTTGA AGGGATAGGA AGAAAGGAAGA	e356	e596	e702	e729
M777	AGGAGTAAACT TGTTGGGTAA TGTGATACGT AAAGAGAGGAA	e364	e83	e358	e222
M778	AGGTGAAGTAT AGTTGATGCT TGTACGGAAT TGAGTAGAACA	e784	e335	e771	e578
M779	TGGTAAGTACT TGTCGAGATT TTAGAGCGAA AGTTGGTAGTA	e348	e286	e309	e365
M780	TTTTTTTTTTT ACGATGTGAT TTGGAACGTA TAGGAAGACAA	nullT11S	e2061	e127	e60
M781	TCGGAATTAGA ATAACGGTGA TTTTTTTTTT TTGTTGCATAA	e597	e323	nullT10N	e464
M782	TAGTGGTTGTA AGCAGGATTT TAGAATGCGA TTAGAGTGTCT	e438	e339	e628	e746
M783	TATGGAGCTAT TTGAGTGTGT TCTTGGGTTT TGTAGGAATCT	e756	e703	e747	e374
M784	AGGGTTCTAAT TAAGGTGCAA ACGTGAAAGA AAAAGTTGTGT	e161	e404	e43	e206
M785	TTCGAAATGTT AAGATGGCTT TAGATGCGAA AGAAGTAGACA	e557	e1276	e163	e386
M786	AGGGATTTAGT TTGTGGCTTA TGTTCAGGTT ATAGGTGACAT	e389	e429	e559	e1594
M787	AGAGAATGAGT TTGGTCAGAA TATGGTTCGT ATTATCAGGGT	e450	e184	e391	e158
M788	AGGGATGTAAT TGAAAGTCGA AAAGGGAGTT AATGTGGAAAA	e88	e351	e1574	e141
M789	AGAATTAGGGA ATGGGAACTT AAGTGGTGAA TTAAGGAAGGT	e108	e629	e510	e806
M790	ACATATGGAGT ACGTGTATGT TCGAGAAGTT TAAGTGACTGA	e581	e441	e3	e725
M791	TTAAGGACTGA TGGTATGCAT TTCGTGGTAA TTTTTTTTTTT	e712	e124	e893	nullT11E

Name	Sequence (5′ → 3′)	Gl. 1/E	Gl. 2/N	Gl. 3/W	Gl. 4/S
M792	ACTGAGGATAA TGTGTCAAGT ATGTGGATCA TTTTCGATTGT	e250	e572	e758	e845
M793	AACTGATAGGA AGTCGGATTT AATGGTGGAT TTTTTTTTTTT	e708	e560	e215	nullT11E
M794	AGTGGATCTTA TTTGTGGGTA AGCAAGAGAA AATAGTCTGGT	e777	e1575	e734	e130
M795	AACAGAGAGAT TTGGCTATGA TTTTTTTTTT AGAGTGGATAA	e833	e167	nullT10N	e798
M796	TATATGGTCGT TGTCTGTGTT ATGGTCGAAT TTTCATAGGGA	e193	e776	e835	e21
M797	TCACCCATTT TATCTTCCTGT TTTTTTTTTTT TACCACAACA	e277	e345	nullT11N	e2209
M798	TGGCTATTAGA AAGGTTCGAT TTTTTTTTTT TTAGTCTTGGA	e65	e188	nullT10N	e2220
M799	AGTTGATAGGT ATCGAGTGTT TAGTTGCGAT TCTTAGAAGGT	e189	e735	e730	e218
M800	AGGAGTAAACT AGTGGAATGT TCGGTGTAAA ATAGGACATGA	e364	e780	e191	e525
M801	AGGGATTCATA ATGTATGGCT TGCGAGAATA TTAAAGAGGGA	e792	e836	e827	e566
M802	AGGAAGATTCT TTGTAGCGAT AGGCAGTAAA AGGTTTAGGTA	e1076	e494	e440	e639
M803	TATGACAGAGT TGTTGTGGTA TTTTTTTTTT ACTTGGTAGAT	e524	e2209	nullT10N	e1184
M804	AGGATGTACTT TTAGCTTGGT TGTTGATCGA TTAGTAGGTGT	e829	e359	e526	e29
M805	TGTGGAGATAT TGTGTCTTGA TAAAGCTGGT TAGTTGTGAGA	e2249	e318	e831	e451
M806	TTTTTTTTTT TCCATAACTCT ATATCTCCACA TCACTTGTCA	nullT10S	e462	e2249	e2251
M807	AACAACTCGA TCCAAGACTAA TTTTTTTTTTT TTACAGCCTT	e2252	e2220	nullT11N	e2253
M808	TAGTCAGTTGA ATTGGACGAT TCGAGTTGTT TAGGTTTCAGA	e262	e731	e2252	e853
M809	ATGAAAATGCT AGTATGCTGT TTTTTTTTTT TCGAGAGATAA	e610	e828	nullT10N	e62
M810	TTTTTTTTTT ATCTGCAAAAT TATCATCGACT ATCACACGAT	nullT10S	e841	e475	e2273
M811	AGTAGTGAAGT TTGAGCGATA TGAAGAGCTT AATCGGAAAAA	e56	e527	e1230	e1036
M812	TTTTTTTTTTT TGACAAGTGA TTAGTCGTGT ACTAAAGGAGT	nullT11S	e2251	e436	e1313
M813	AGTCGATGATA AAGGCTGTAA TTTTTTTTTT TTCATGTTGTT	e475	e2253	nullT10N	e2
M814	AAGAGGTAGAA AAGACGGAAA TCTTGTGTGT ATGTAGTAGCA	e1236	e265	e289	e259
M815	TGATAGGACTT TGTTGGAACT AGACATGGTT TGGAGACTAAT	e884	e502	e1238	e296
M816	AGTAGTGACAT AGGTTTGTGA AAGTGGACAT AGTAATCAGGA	e589	e613	e91	e983
M817	AGTCTAAAGGA AGTGCAGAAT TTTGAGTGGA TTTCATTTGGT	e532	e686	e591	e329
M818	TTTTTTTTTTT ATCGTGTGAT AAATTGCGTT AGAGTTATGGA	nullT11S	e2273	e534	e462
M819	AGAAGTACAGA ATAACGGTGA TTTTTTTTTT ATTCGAGTAGT	e1260	e323	nullT10N	e2308
M820	AGGTATGGAAT TTGTGGTGAT TTCAAAGGGT TGAGAGAAAGA	e837	e59	e1262	e733
M821	TTGACATTGTT AGAGTGCTTT TGATGATCGT TTCTGTAGTGA	e1341	e506	e313	e398
M822	ATGTAGGGTAA ATTTGCGTTT ATCGGTGTAA TTTAAATGCGA	e303	e815	e31	e190
M823	TTAGGTAGCAT ATCATTGGGT AAAGAAGGGT TAAGAAGGTCA	e324	e1239	e86	e231
M824	TTATTTTGCGT ATTGGATCGA TTTTTTTTTT AGTGTAAGAGA	e1027	e92	nullT10N	e738
M825	TAGAGGAAAGT TTGTGCTAGT ATGAGGTGTT AGTTGGTAGTA	e153	e19	e375	e365
M826	ACCTTCAGTT ACTACTCGAAT TTTTTTTTTTT AACAATCCCT	e653	e2308	nullT11N	e2341
M827	TTCGGATAGAA AGCTGAGAAT AGAAGACGTT AGAGTTAGGAA	e651	e840	e142	e194
M828	TTTTTTTTTTT TTGGGTGTTA AACTGAAGGT ATTTTTGACGT	nullT11S	e150	e653	e105
M829	AGAACAGAGTA AGTTTGGTGA TTTTTTTTTT AGTAGGAAGAA	e234	e32	nullT10N	e122
M830	AGAGATGACTT TTTGGATGGT TGGATGTCAA ATGAGTTAGGT	e275	e306	e236	e476
M831	AGTAGACTGAA AGAGCGTAAT AAATGGGTGA TAGAGAATCGA	e73	e343	e277	e109
M832	TTTTTTTTTTT AGGTTGGAAT TGTAGGCAAT AGAATCAGGAT	nullT11S	e509	e411	e368
M833	AATGCAGATTT AGGGATTGTT TTTTTTTTTT ATCGGTAGTAA	e328	e2341	nullT10N	e2374
M834	TTGAGAGTAGT AGTGATCGAA TTTGGAGTGT ACAATTGGAAA	e238	e763	e330	e480
M835	AGGGTTAGAAT TTAGGCAAGT TCATGGATGT AGATTATCGGA	e520	e237	e151	e214
M836	AGAACTAAGGA AAGACGGAAA TAATGGAGCA AGTACGAGTAT	e136	e265	e178	e1049
M837	TTTTTTTTTTT TGAGTGCATA TTGTTGGCTA AGAATATGGGT	nullT11S	e75	e138	e713
M838	AGAACTAAGGA TGGAAGGAAA TTTTTTTTTT TGTTTGATGTT	e136	e768	nullT10N	e2396
M839	AGCTTAGATGA TGTTGGTCTT TGGTGCATAT ATTAGTCTGGA	e181	e380	e223	e623
M840	AGGAAGGAATA ATGAAGACGA AGAAGCAAGA AGATAGCGTAT	e270	e412	e183	e598
M841	AACAGAACCT TTACTACCGAT TTTTTTTTTTT TCACCAAACT	e2406	e2374	nullT11N	e32
M842	ATCTTAGGGAA AAGGGACAAT AGGTTCTGTT AAAATGCAAGA	e857	e331	e2406	e316
M843	AGGGATGTAAT ATGTAGCAGT TGACTGGAAA AGGATATAGCA	e88	e241	e859	e94
M844	TTTTTTTTTTT TGCAGAAGAT TTGTGTAGCA ACTTTAGAGGT	nullT11S	e135	e799	e180
M845	AACGTGAAATT AAGGGTGAAT TTTTTTTTTT AAAAGTGAACA	e258	e152	nullT10N	e370
M846	AGGTACGATTA TTTGGGTGAT AAGGCTGATT TAGGGATGAAA	e1093	e4	e260	e267
M847	TCAGCATCTT AACATCAAACA TTTTTTTTTTT ACCCATGAAA	e481	e2396	nullT11N	e2429
M848	TAGAGATGCTT TTGGTCAGAA TGTTCGTGTA TGCATATAGGT	e1060	e184	e669	e705
M849	TTTTTTTTTTT AGATGACGTT AAGGAATCGA ATTACAGAGGT	nullT11S	e415	e240	e796
M850	AAGTACTGAGA AGTGATCGAA TCTTGTGTGT TTAGTTCAGGA	e121	e763	e289	e263

Name	Sequence (5′ → 3′)	Gl. 1/E	Gl. 2/N	Gl. 3/W	Gl. 4/S
M851	AAGTCGAGTAT ATGATGGTGT TTATGGCAGT ATTAGTGGGTA	e225	e204	e123	e49
M852	TTTTTTTTTTT TTTGGGTTCT TTTGTAGGCT TGATAGTGCTA	nullT11S	e751	e114	e2460
M853	TGGAGATACAT TTTCATGGGT TTTTTTTTTT TTGTTGCATAA	e172	e2429	nullT10N	e464
M854	TCGGTAGATTA TTTTCTGGGA TATTGCGAGT AGAGTTGAGTA	e741	e670	e232	e643
M855	TAGAAGGCATA TGGCTGTTAA ATGTCGGTAA ATCAAGGTAGA	e307	e363	e106	e1516
M856	TTTTTTTTTTT AGTTGGTTCA TGGATATGCA ATAGGACAAGT	nullT11S	e227	e78	e650
M857	ATGGATTAGGA TTGTCAGGAA ACGATGGAAT TAGGGTAACAT	e479	e314	e1082	e117
M858	AGATAGTGGTT TCTGTTTGGT AAGATGCTGA AAAATTAGCGT	e2491	e449	e481	e81
M859	TTTTTTTTTT TAGCACTATCA AACCACTATCT TGCTAACCAT	nullT10S	e2460	e2491	e2493
M860	ACAGTAGGATT ATGATGGTGT AAAGTGGTCT TTTGTCAATGT	e157	e204	e620	e963
M861	TTTTTTTTTTT AGTCGTTTGA TGCAGTAAGT ATTGAAGCAAA	nullT11S	e826	e1789	e274
M862	AGGGTTCTAAT TTCGATGGAT TTTTTTTTTT AGGAGTTTACT	e161	e107	nullT10N	e17
M863	TTATCTGTGGA TGTAAGTGCT TCGGTATGTT AGCTAGTGTTA	e125	e310	e1562	e34
M864	AGTTGTAGTCT TGGTTGCTAA TATTGGTCGT ATGATTGCAAT	e695	e79	e648	e198
M865	AACTCCAACT TCCTCATAGTT TTTTTTTTTTT TACCAATGCA	e2516	e137	nullT11N	e2517
M866	AAGTGAGGATA TTGAGCGATA AGTTGGAGTT AGACTTAGGAA	e801	e527	e2516	e126
M867	TTTTTTTTTTT ATGGTTAGCA AAGGTTCAGT ATCAGGATGTA	nullT11S	e2493	e110	e556
M868	AGTCTAAAGGT TGGTTCTTGA TTTTTTTTTT AACTATGAGGA	e737	e253	nullT10N	e137
M869	AGCAGGTATTA TTGTAGCGAT AGTTCGTGAA TGGAGTAAGAT	e1007	e494	e739	e186
M870	TAAAATTGGCA ATGCTGAAGA TTATGCTGGT TATTCGAGGTA	e319	e1790	e624	e834
M871	TTGAGGGATAT ACGGATGAAT TAATGGCTGT TAAAACGTGTT	e61	e1563	e46	e660
M872	TTTTTTTTTTT TTGGGTTTCT TTATGGACGT TTGACGAAAAT	nullT11S	e697	e244	e781
M873	TAGAGGAAAGT TGCATTGGTA TTTTTTTTTT AGGAGAATTCT	e153	e2517	nullT10N	e2550
M874	TAAGAGTGTGA TGTTACGAGT TGATGTCGAT TCAAGAAGGTA	e548	e111	e518	e42
M875	ATTTGCTGATT TACGTTGAGT TGTTCTGTGT TATTGAGGCTA	e573	e740	e166	e541
M876	AGGAGTGTATT AAGAAGTCGA TTGAGCTGAA TATAGACGTGT	e861	e139	e575	e255
M877	TTTTTTTTTTT AGCTGATTGA ACGGAGAAAT AGGTCATTAGT	nullT11S	e321	e863	e736
M878	AGGTAATGGTA ACGAGTTGAA TTTTTTTTTT TAGCTGAGTAA	e704	e47	nullT10N	e2572
M879	TTTGACTGTTT ATGATGAGCA AGCTGAAGTT TTTACTGGGTA	e844	e64	e99	e406
M880	AGTATGTCTGA TAGTGGCAAT ATGCGTAGAA TTAGAGTGACA	e809	e87	e58	e631
M881	TGCCAACTTA AGAATTCTCCT TTTTTTTTTTT TAGCATCCAA	e2582	e2550	nullT11N	e2583
M882	AAGGGATTAGT TGTCGTTAGT TAAGTTGGCA AGGAGCATATA	e37	e156	e2582	e537
M883	AGGAGTTACTT TGTTGTCAGA TTCGGAGTTT TAGGGTAACAT	e381	e519	e39	e117
M884	TTTTTTTTTTT AGTTGATCGT TAGAAAGCGA TGAGGTATGAT	nullT11S	e603	e383	e45
M885	AGTCTAAAGGA TTGGCTATGA TTTTTTTTTT AGAGCTATGAT	e532	e167	nullT10N	e182
M886	ATGGATGTCTA AGGCTGAAAT TTTTGAGGGT AAAGCATTGAT	e299	e576	e1757	e390
M887	TTTTTGTCTGT AACTTGTGGA TCTGTTGGAA AGGTCTTGATA	e764	e864	e301	e821
M888	TGCTACAACT TTACTCAGCTA TTTTTTTTTTT TCACCAAACT	e2604	e2572	nullT11N	e32
M889	AGAGAATGAGT TTGGCAGTTA AGTTGTAGCA AGATAGCGTAT	e450	e707	e2604	e598
M890	AGACTAATGGA TTGGCTTGTA TCTTTGGGAA TGTGATAGACA	e528	e847	e187	e25
M891	TTTTTTTTTTT AGGTGTCTTT AAATTGCGTT TGATTTGTTGT	nullT11S	e811	e534	e2614
M892	TGAGGACTATT TTGGATGCTA TTTTTTTTTT AAAAGTGAACA	e76	e2583	nullT10N	e370
M893	AGTGATTAGGA TGTGGTACAA AGGCAGTAAA TAGAGGAACAT	e144	e40	e440	e789
M894	TAGCATGAGTA TTGGTGGTAA AAGGTTCAGT ATGACAGTAGT	e434	e327	e110	e785
M895	TGAGTATCTGT ATGGGTTGAA TTAGTCGTGT AGATCAGTGTA	e467	e302	e436	e169
M896	TGGCAAAATAA AGGAACGATT AAAGAGGGTT AGGTTCTAGTT	e52	e12	e155	e145
M897	TTTTTTTTTT ACAACAAATCA ATGCTACCTAA ACACTTCCAA	nullT10S	e2614	e324	e2647
M898	TGGGATCTATT TGTGTCATGA AAGGGAACAA TTTAGGACGTA	e816	e791	e416	e1492
M899	AAAAGGGTCTA AGTAGTGCAT ATGACGTGTA TTTTGCATGAT	e614	e147	e818	e1233
M900	TTTTTTTTTTT TGTGATTGGA AGGTGCTTAA ATTTTGCAGAT	nullT11S	e1737	e616	e841
M901	AGTAGATACGT AGGAGGTAGA TTTTTTTTTT AGGCTAAGTAT	e503	e229	nullT10N	e873
M902	TTCTATGTGGA ATTGGGTCAT AATCAGTGGT ATAGTGAGGAA	e213	e224	e814	e325
M903	AACTGATAGGA ATGTGGTCTT ATGACATGGT TCTGGGATTAT	e708	e645	e782	e235
M904	AGACGGTAATA AAGAAGTGGT TGTGTACGAA TGAAGTAAGGA	e606	e55	e63	e271
M905	TTTTTTTTTTT TTGGAAGTGT TTGGACGTAA TCAAAATGTGT	nullT11S	e2647	e608	e2680
M906	AGTGTGAGATA TTCGGAAGAT TTTTTTTTTT AGAGTAAGGTT	e1536	e417	nullT10N	e858
M907	ACTGAGGATAA TTGAGTCGAT TATGTGTCGT AGATCTGAGTT	e250	e819	e1538	e419
M908	TTAAGGACTGA ATTTGCGTTT AACTGTGTGA TTTTTTTTTTT	e712	e815	e716	nullT11E
M909	TTGCGATAAAA AGAGCAATGA AGTAGCGAAT TTTTTTTTTTT	e1816	e216	e714	nullT11E

**Name**	**Sequence** ($5' \to 3'$)	**Gl. 1/E**	**Gl. 2/N**	**Gl. 3/W**	**Gl. 4/S**
M910	ATTCGTAGAGA TGGTTCTTGA TTTTTTTTTT TTTTTTTTTTT	e897	e253	nullT10N	nullT11E
M911	TGAGGATGTTA AAATGAGGCT TGCATGAGAT TTTTTTTTTTT	e715	e347	e898	nullT11E
M912	AGGTTAAGACT TTGAGATCGT AACTGTGTGA TTTTTTTTTTT	e2701	e551	e716	nullT11E
M913	TTTTTTTTTT ACACATTTTGA AGTCTTAACCT TTTTTTTTTT	nullT10S	e2680	e2701	nullT10E
M914	AAAGAGGAGAT TGATATGGCA ATCGGATTGT TTTTTTTTTTT	e719	e376	e887	nullT11E
M915	TTAGGATGACA TGTGCAGATA TTTCGATGGA TTTTTTTTTTT	e185	e1272	e720	nullT11E
M916	TTTTTTTTTTT TACGGTGAAT ATCGGATTGT TTTTTTTTTTT	nullT11S	e90	e887	nullT11E

4.2 Fluorophore- and quencher-modified strands

The sequences below use Integrated DNA Technologies' (IDT) code format for DNA modifications.

Name	Tile	Sequence
H-ROX	H173	/56-ROXN/TTTGGAGTGT ACTTTAGAGGT TTTTGATTCGT TGTCGAGATT
	H148	/5IAbRQ/ATGTGAGTAGT TTAGGCAAGT TGTGCAGTTA AAATTGGAACA
H-FAM	H306	/56-FAM/TTTGAGTGGA AGTGAGTAAGA TGGCTTTTTTA AGAACGGATT
	H281	/5IABkFQ/ACAGGTTAGAT ATGGTTGGAT TCAAGTTGGT ACAGGAAGATA
H-five	H227	/5ATTO550N/TAGTGGTTGTA TGTAAGTGCT TTAGTCGTGT TTTTTAAGCGT
	H252	/5IAbRQ/ACTGGGAATT TAATACGAGGA TTGGGATATCT ACGTGTATGT
H-six	H428	/5ATTO647NN/AGGTAAGAGAA TGTGTCATGA ATGACGTGTA AGGTCTTGATA
	H445	/5IAbRQ/TAGATGCGAA TAGGTTTCAGA AGATGGAGATT AGGTTAACGT
A-ROX	A636	/56-ROXN/TATGACAGAGT ATGATGAGCA ATTGAAGGGT ATATAGAGGCA
	A647	/5IAbRQ/AGTGAGTTGT TTAGAGTGTCT AGTGTGAGATA TTGAGCGATA
A-FAM	A544	/56-FAM/TCTTGTTGGT ATATGGTAGCT AATGCAGATTT TTGTGGCTTA
	A528	/5IABkFQ/TTTTGATTCGT TGGTATGCAT ACGGAGAAAT AGTTGGTAGTA
A-five	A539	/5ATTO550N/TGGCTATTAGA TGGTTTGCTA TTGTTGGCTA TTTTCGATTGT
	A550	/5IAbRQ/AATGTGAGGT TGAAGTAAGGA AGGGATTTAGT TGAAATGGGT
A-six	A643	/5ATTO647NN/TTTGGGTACA AGGTTTAGGTA ACTTGTAGGAT AGCGTTGATA
	A631	/5IAbRQ/AAAAGGGTCTA ATGCTGAGAT AGGCAGTAAA AGAGTTAGGAA
M-ROX	M863	/56-ROXN/TCGGTATGTT AGCTAGTGTTA TTATCTGTGGA TGTAAGTGCT
	M855	/5IAbRQ/TAGAAGGCATA TGGCTGTTAA ATGTCGGTAA ATCAAGGTAGA
M-FAM	M830	/56-FAM/AGAGATGACTT TTTGGATGGT TGGATGTCAA ATGAGTTAGGT
	M836	/5IABkFQ/TAATGGAGCA AGTACGAGTAT AGAACTAAGGA AAGACGGAAA
M-five	M761	/5ATTO550N/TGACGTTGTA AAAGGCTAGTA AGAGTTTAGGA TGGACTTTGT
	M754	/5IAbRQ/ATTTGCTGATT AGTTCTGGTT TCAAGTTGGT AAGAAAAGCAA
M-six	M759	/5ATTO647NN/TGGTCATAGAT TACGTTGGAT ATCAAAGGGT AGTATTACGGA
	M767	/5IAbRQ/ATGGTGTCAA AATGTCTAGGA TAGATGTACGT TAAGTGGCAT

Section 5

Flag patterns

5.1 Protocols

5.1.1 Flag patterns, constant temperature

1. 85 °C for 3 minutes, no fluorescence measurement.
2. 71 °C to 55 °C, in steps of -2.0 °C every 4 minutes, taking a measurement at the end of each hold (36 minutes in total).
3. 53 °C to 47.2 °C, in steps of -0.2 °C every 12 minutes, taking a measurement at the end of each hold (6 hours in total).
4. A 47 °C hold, taking a measurement every 12 minutes (51 hours in total).
5. 47 °C to 40.5 °C, in steps of -0.5 °C every 13 minutes, taking a measurement at the end of each hold (3 hours 2 minutes in total).

5.1.2 Flag patterns, ramp

1. 71 °C for 4 minutes, no fluorescence measurement.
2. 71 °C to 55 °C, in steps of -2.0 °C every 4 minutes, taking a measurement at the end of each hold (36 minutes in total).
3. 53 °C to 48.2 °C, in steps of -0.2 °C every 12 minutes, taking a measurement at the end of each hold (5 hours in total).
4. 48 °C to 46.1 °C, in steps of -0.1 °C every 5 hours, taking a measurement every 30 minutes (100 hours in total).
5. 46 °C to 39.5 °C, in steps of -0.5 °C every 13 minutes, taking a measurement at the end of each hold (3 hours 2 minutes in total).

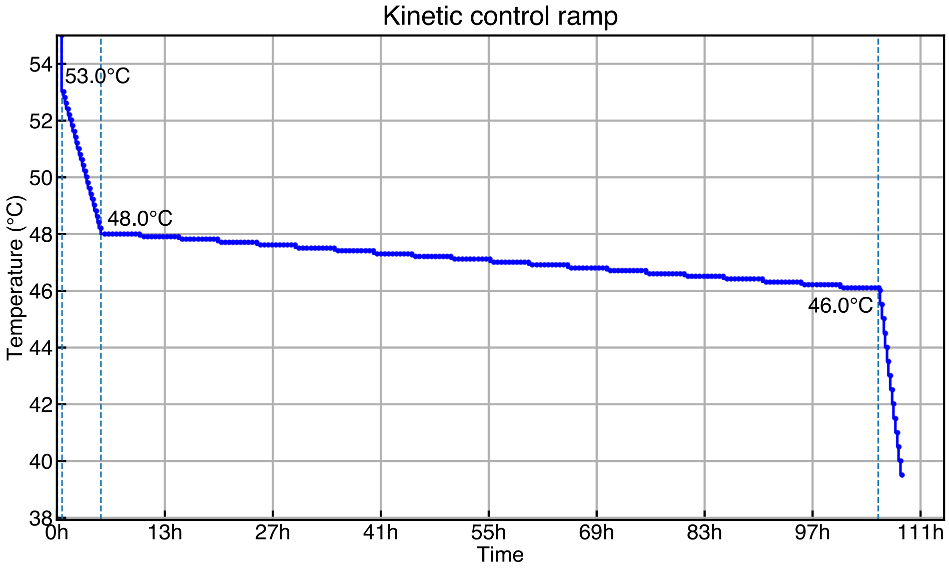

5.2 Nucleation model summaries

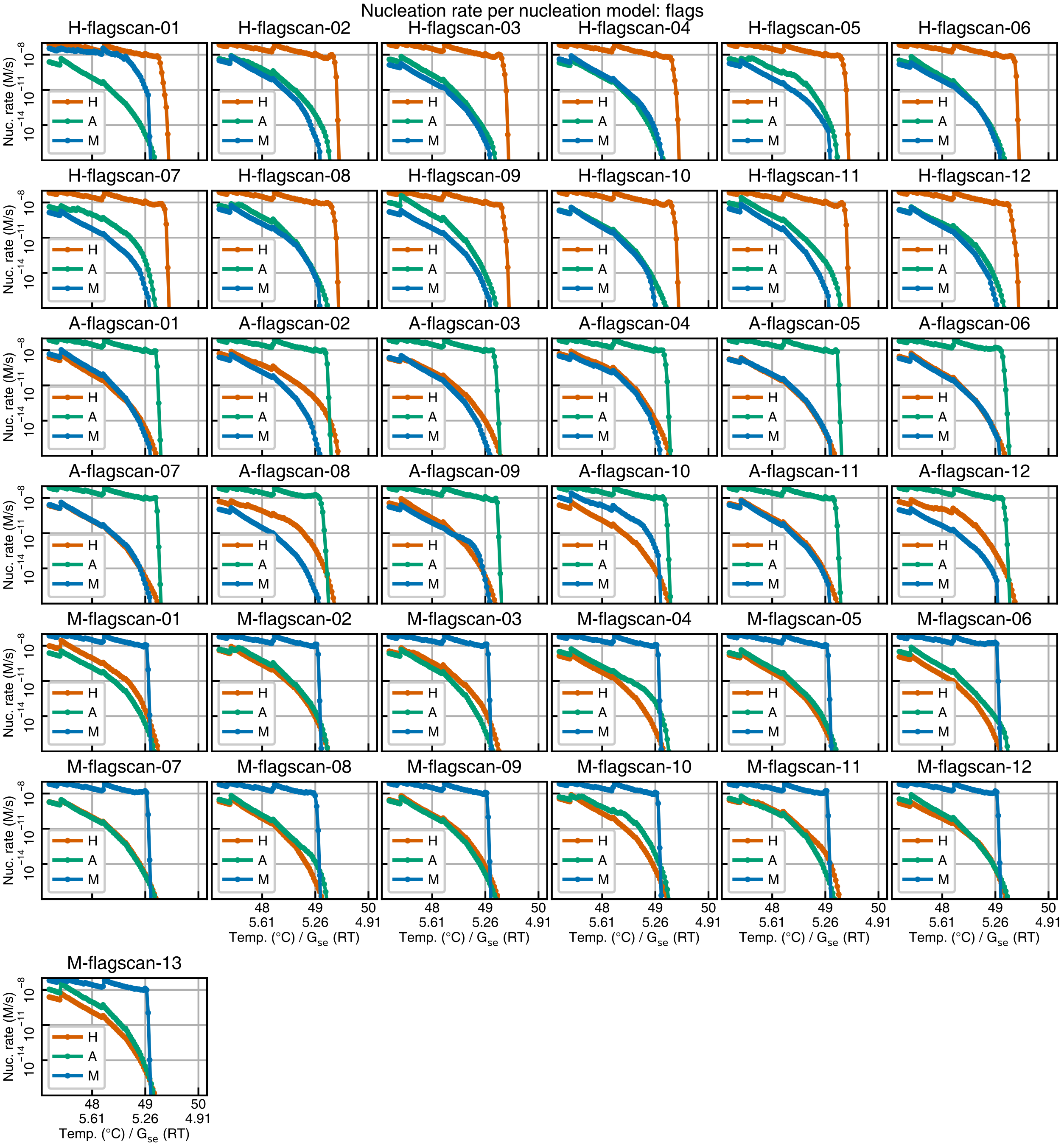

5.3 Individual results

5.3.1 H flag 1

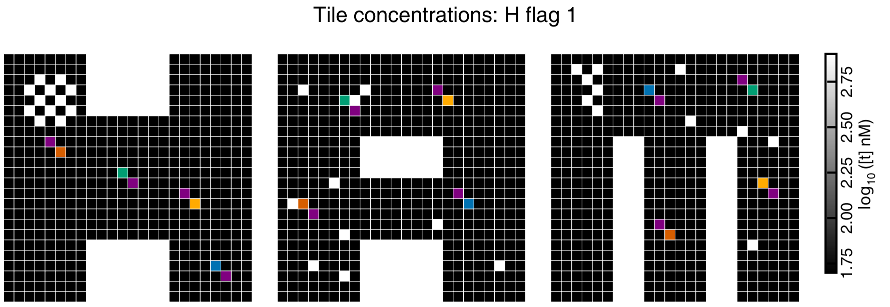

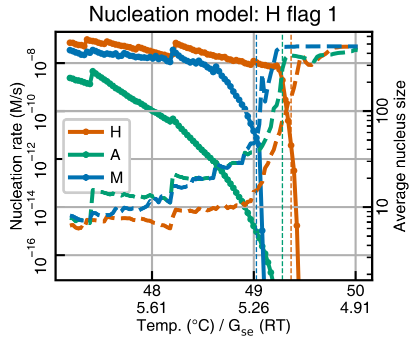

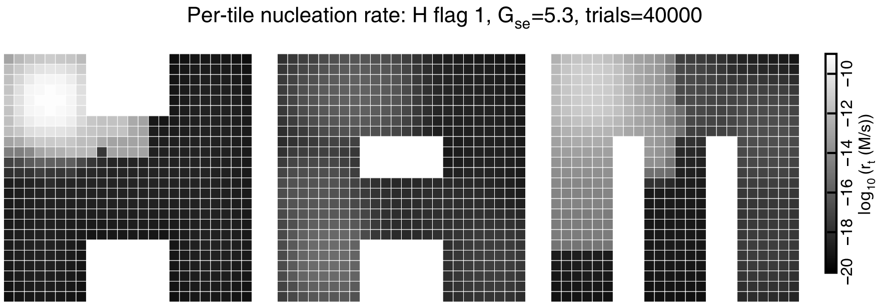

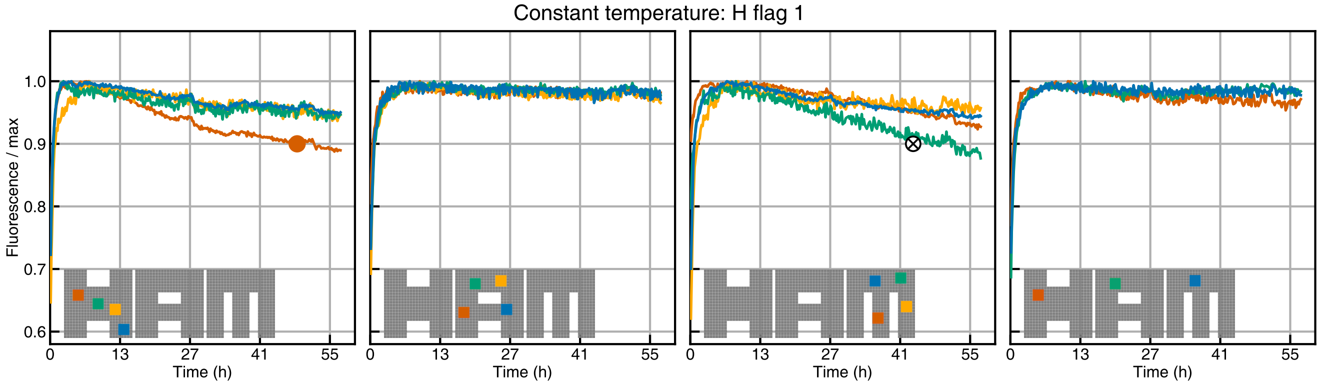

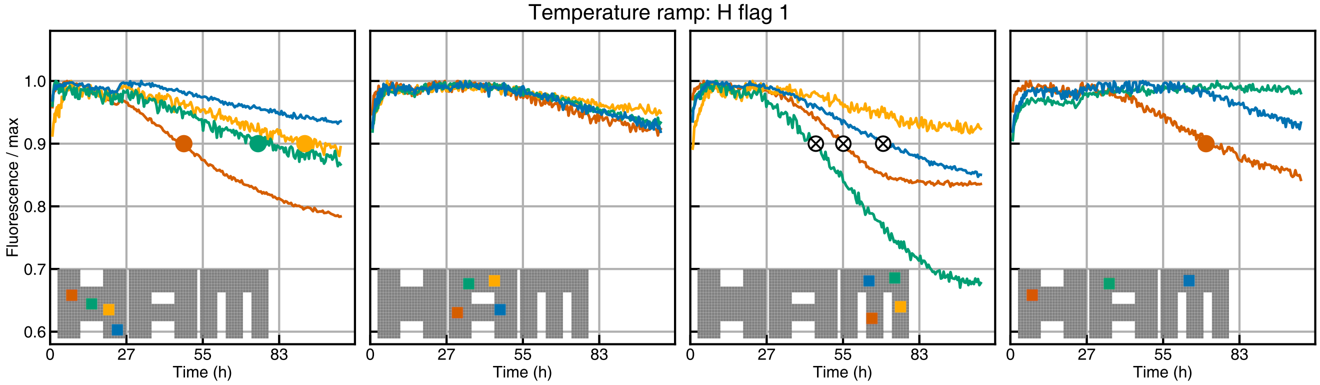

5.3.2 H flag 2

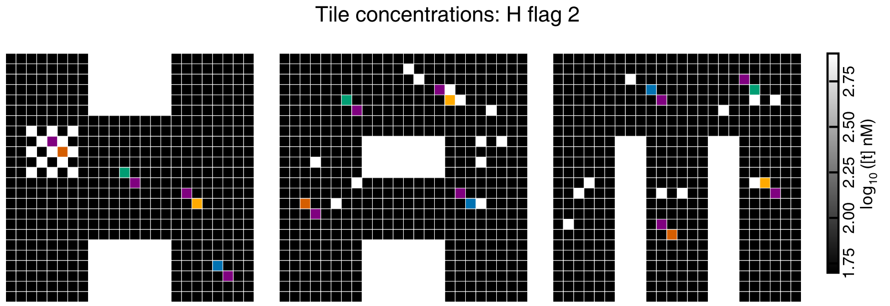
Tile concentrations: H flag 2
1.75 2.00 2.25 2.50 2.75
$\log_{10}$([t] nM)

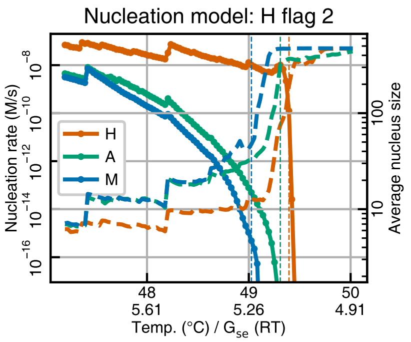
Nucleation model: H flag 2
Nucleation rate (M/s)
10^{-8} 10^{-10} 10^{-12} 10^{-14} 10^{-16}
H
A
M
Average nucleus size
10 100
48 49 50
5.61 5.26 4.91
Temp. (°C) / G_{se} (RT)

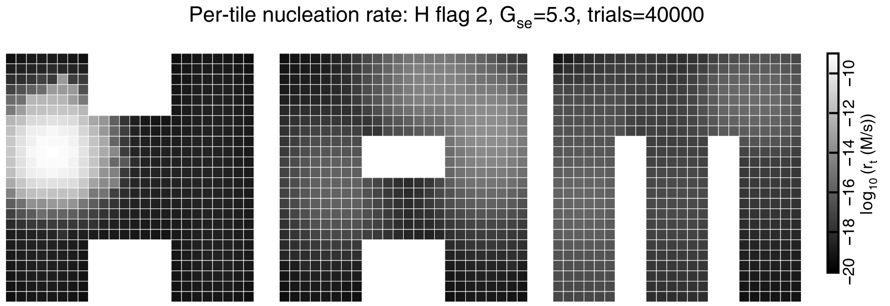
Per-tile nucleation rate: H flag 2, G_{se}=5.3, trials=40000
−20 −18 −16 −14 −12 −10
$\log_{10}$ (r_t (M/s))

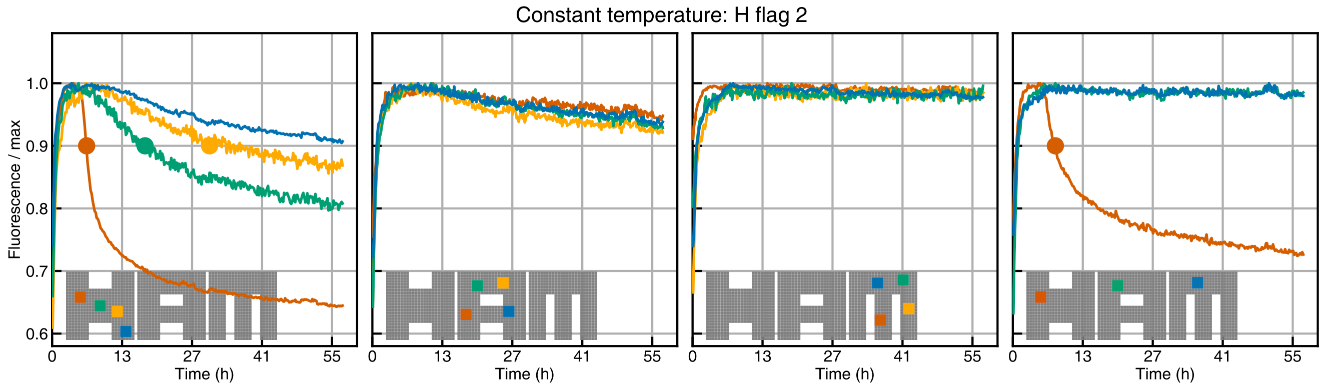
Constant temperature: H flag 2
Fluorescence / max
1.0 0.9 0.8 0.7 0.6
0 13 27 41 55
Time (h)

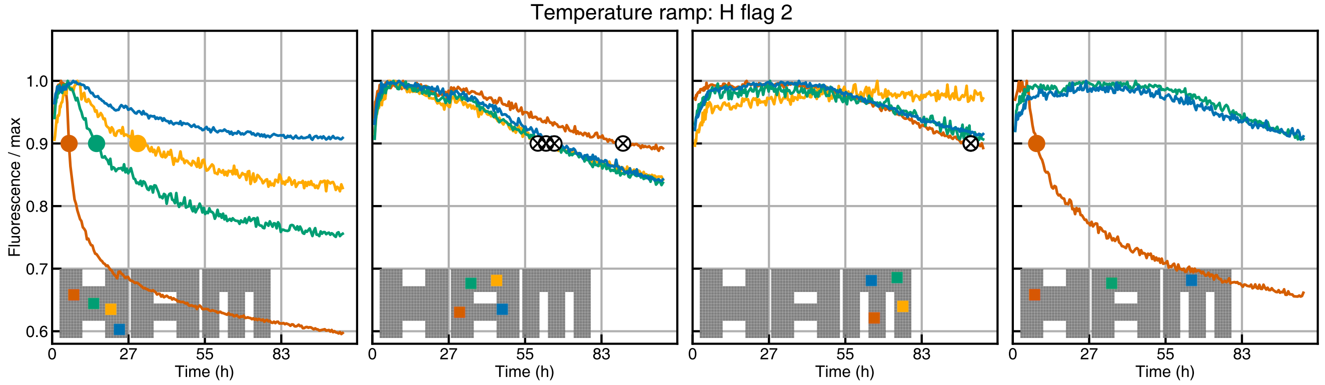
Temperature ramp: H flag 2
Fluorescence / max
1.0 0.9 0.8 0.7 0.6
0 27 55 83
Time (h)

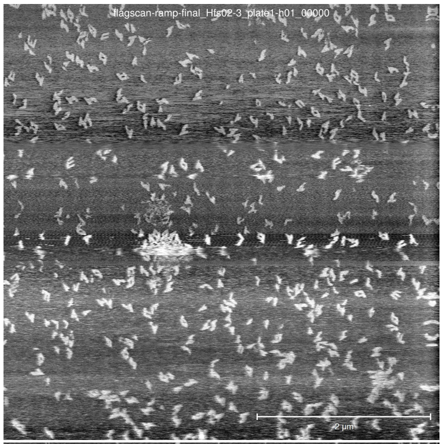
flagscan-ramp-final_Hfs02-3_plate1-h01_00000
2 µm

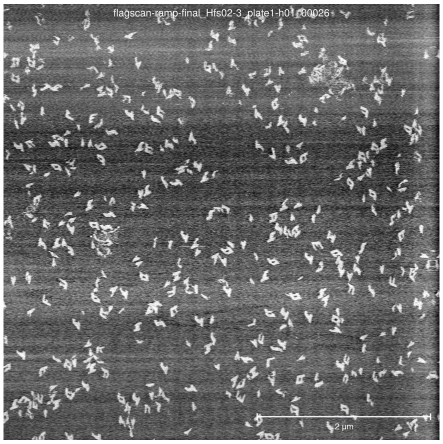
flagscan-ramp-final_Hfs02-3_plate1-h01_00026
2 µm

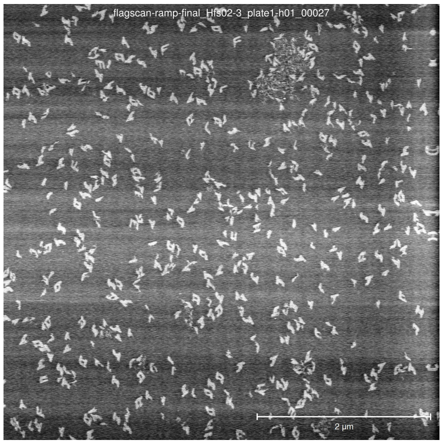
flagscan-ramp-final_Hfs02-3_plate1-h01_00027
2 µm

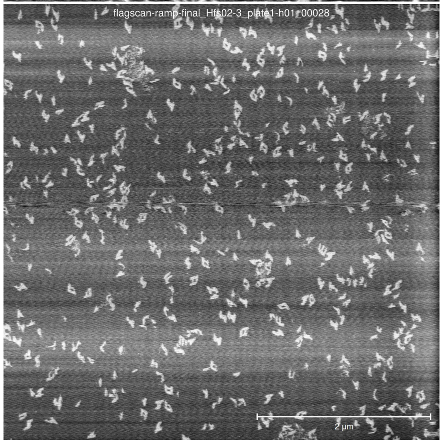
flagscan-ramp-final_Hfs02-3_plate1-h01_00028
2 µm

5.3.3 H flag 3

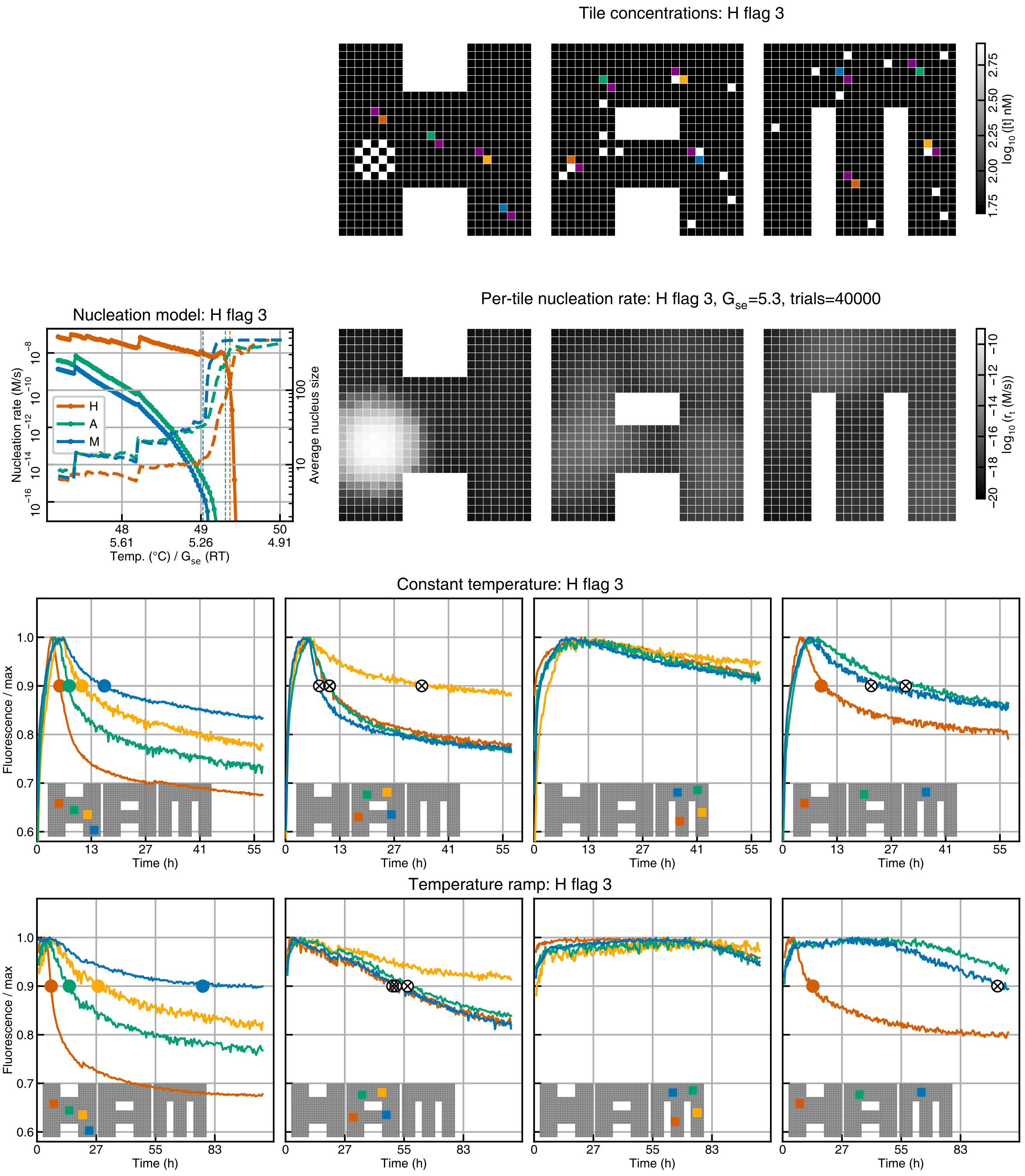

Tile concentrations: H flag 3
$\log_{10}$([t] nM)
1.75 2.00 2.25 2.50 2.75
Nucleation model: H flag 3
Nucleation rate (M/s)
Average nucleus size
H
A
M
48 49 50
5.61 5.26 4.91
Temp. (°C) / G_{se} (RT)
Per-tile nucleation rate: H flag 3, G_{se}=5.3, trials=40000
$\log_{10}$ (r_t (M/s))
−20 −18 −16 −14 −12 −10
Constant temperature: H flag 3
Fluorescence / max
Time (h)
0 13 27 41 55
Temperature ramp: H flag 3
Fluorescence / max
Time (h)
0 27 55 83

5.3.4 H flag 4

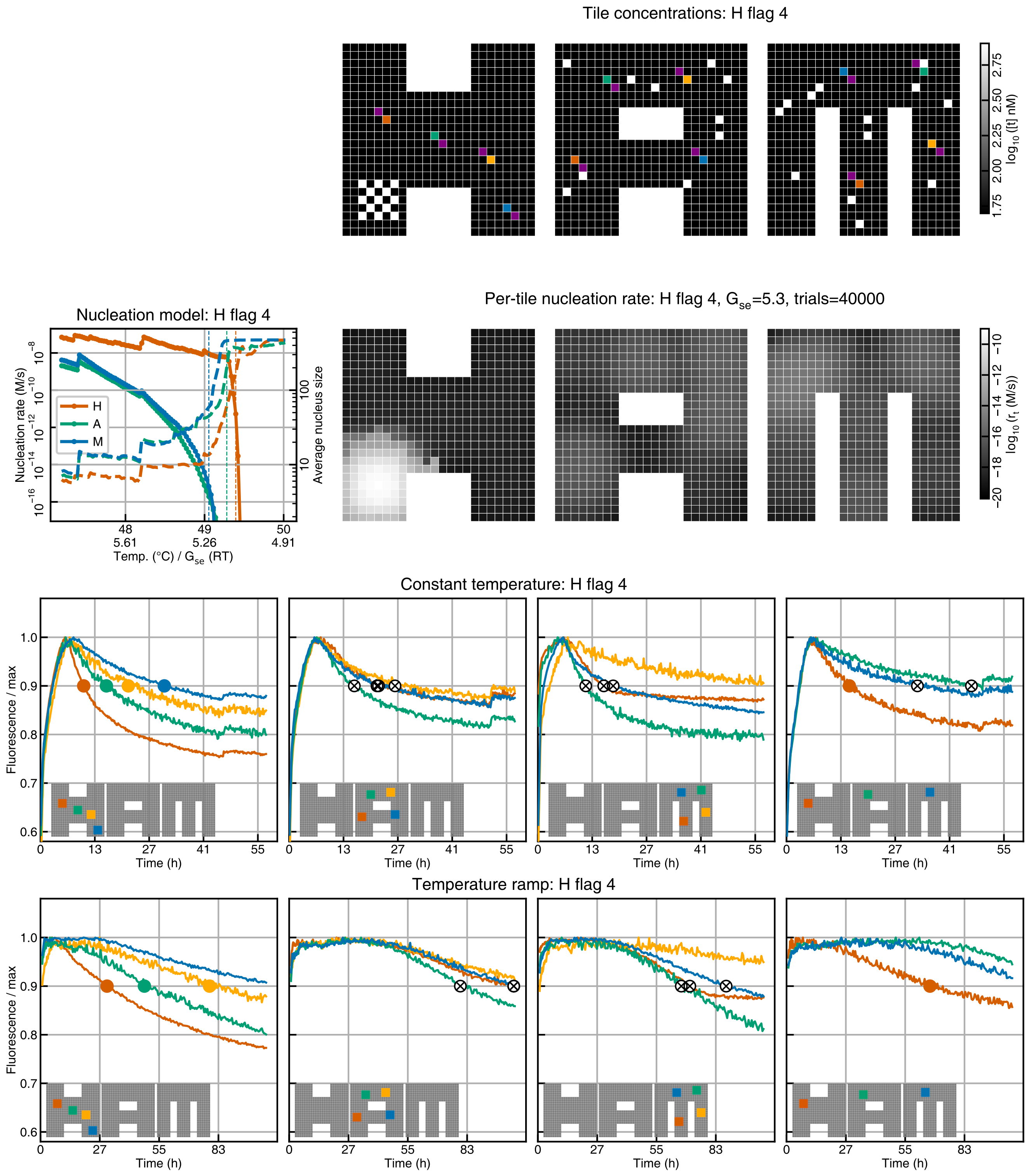

Tile concentrations: H flag 4
$\log_{10}([t]$ nM)
Nucleation model: H flag 4
Nucleation rate (M/s)
Average nucleus size
H
A
M
Temp. (°C) / G_{se} (RT)
Per-tile nucleation rate: H flag 4, G_{se}=5.3, trials=40000
$\log_{10}(r_t$ (M/s))
Constant temperature: H flag 4
Fluorescence / max
Time (h)
Temperature ramp: H flag 4
Fluorescence / max
Time (h)

5.3.5 H flag 5

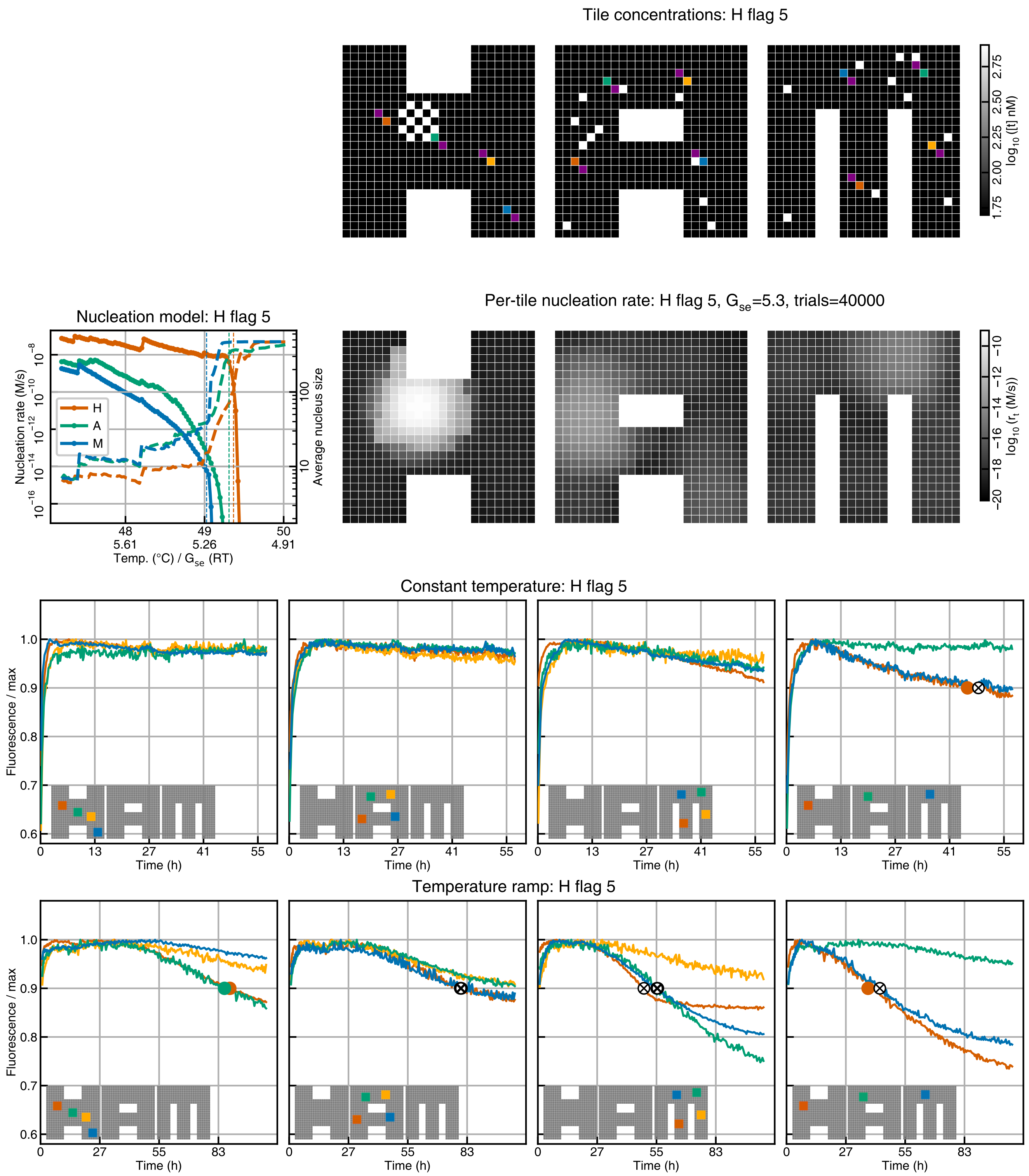
Tile concentrations: H flag 5
log10 ([t] nM)
1.75 2.00 2.25 2.50 2.75
Nucleation model: H flag 5
Nucleation rate (M/s)
Average nucleus size
H
A
M
48 49 50
5.61 5.26 4.91
Temp. (°C) / Gse (RT)
Per-tile nucleation rate: H flag 5, Gse=5.3, trials=40000
log10 (rt (M/s))
−20 −18 −16 −14 −12 −10
Constant temperature: H flag 5
Fluorescence / max
Time (h)
0 13 27 41 55
Temperature ramp: H flag 5
Fluorescence / max
Time (h)
0 27 55 83

5.3.6 H flag 6

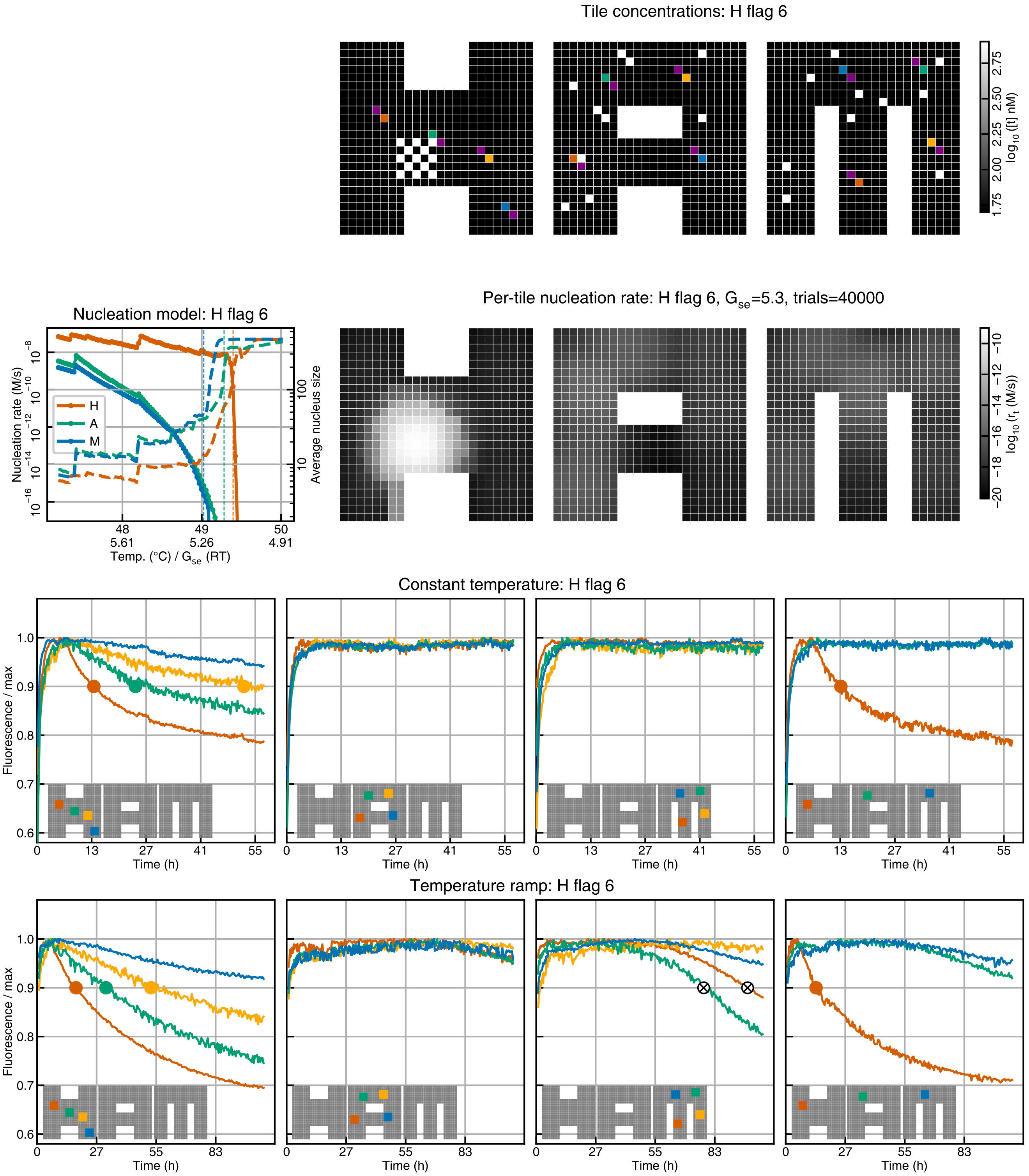
Tile concentrations: H flag 6
log10 ([t] nM)
1.75 2.00 2.25 2.50 2.75
Nucleation model: H flag 6
Nucleation rate (M/s)
Average nucleus size
H
A
M
48 49 50
5.61 5.26 4.91
Temp. (°C) / G_{se} (RT)
Per-tile nucleation rate: H flag 6, G_{se}=5.3, trials=40000
$\log_{10}(r_t$ (M/s))
Constant temperature: H flag 6
Fluorescence / max
Time (h)
Temperature ramp: H flag 6
Fluorescence / max
Time (h)

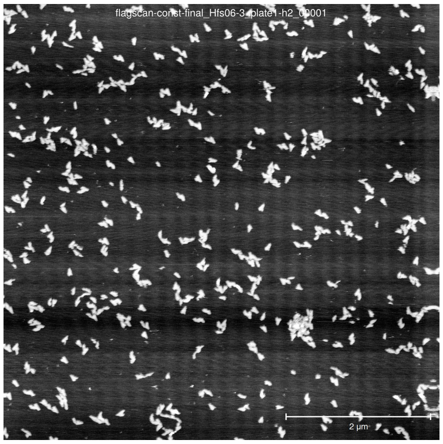
flagscan-const-final_Hfs06-3_plate1-h2_00001
2 µm

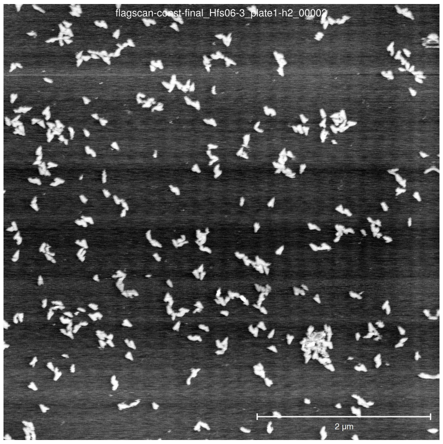
flagscan-const-final_Hfs06-3_plate1-h2_00002
2 µm

5.3.7 H flag 7

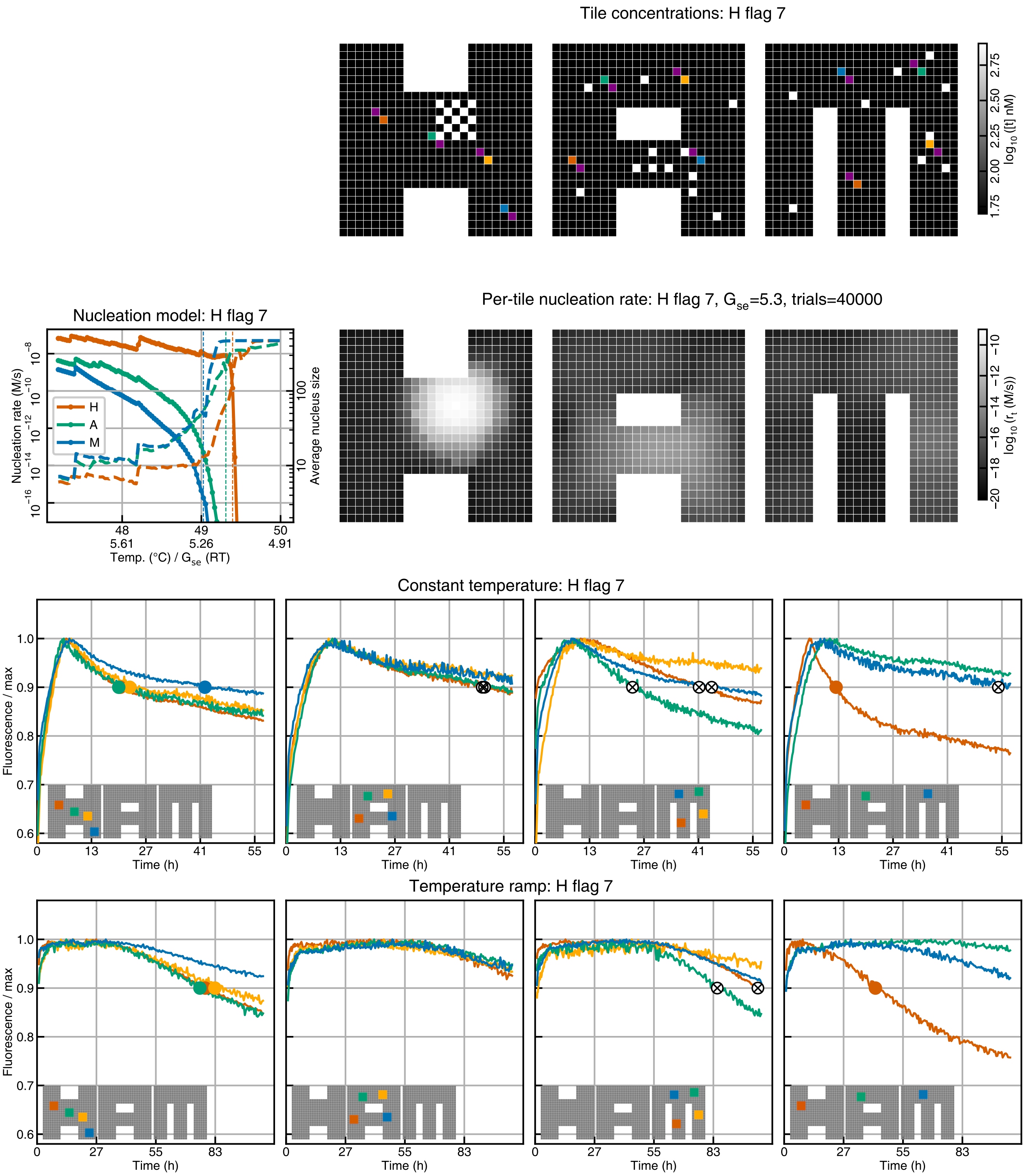
Tile concentrations: H flag 7
log10([t] nM)
1.75 2.00 2.25 2.50 2.75
Nucleation model: H flag 7
Nucleation rate (M/s)
Average nucleus size
H
A
M
48 49 50
5.61 5.26 4.91
Temp. (°C) / G_se (RT)
Per-tile nucleation rate: H flag 7, G_se=5.3, trials=40000
log10 (r_t (M/s))
−20 −18 −16 −14 −12 −10
Constant temperature: H flag 7
Fluorescence / max
Time (h)
Temperature ramp: H flag 7
Fluorescence / max
Time (h)

5.3.8 H flag 8

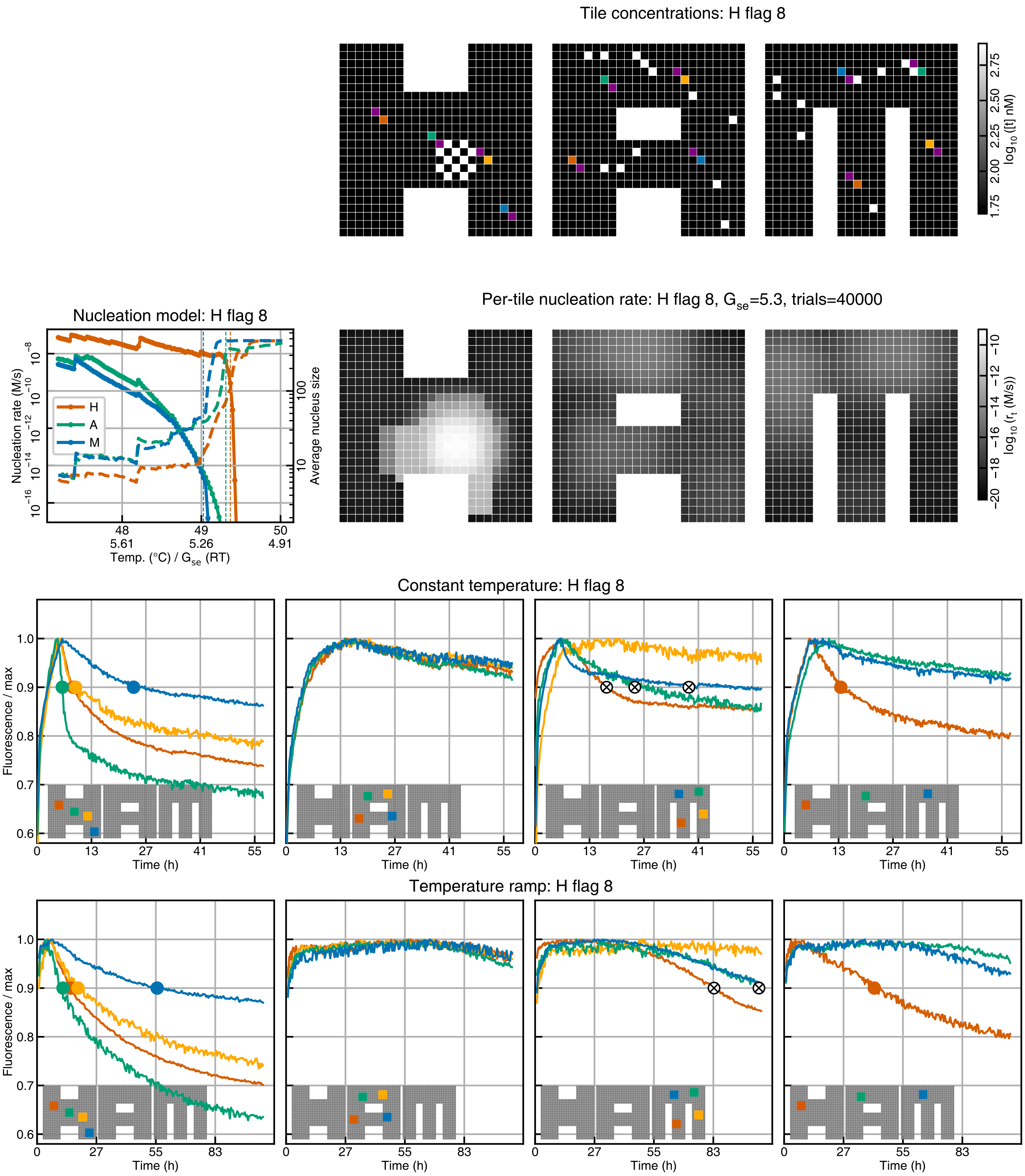
Tile concentrations: H flag 8
$\log_{10}$([t] nM)
1.75 2.00 2.25 2.50 2.75
Nucleation model: H flag 8
Nucleation rate (M/s)
Average nucleus size
H
A
M
48 49 50
5.61 5.26 4.91
Temp. (°C) / G_{se} (RT)
Per-tile nucleation rate: H flag 8, G_{se}=5.3, trials=40000
$\log_{10}$ (r_t (M/s))
Constant temperature: H flag 8
Fluorescence / max
Time (h)
Temperature ramp: H flag 8
Fluorescence / max
Time (h)

5.3.9 H flag 9

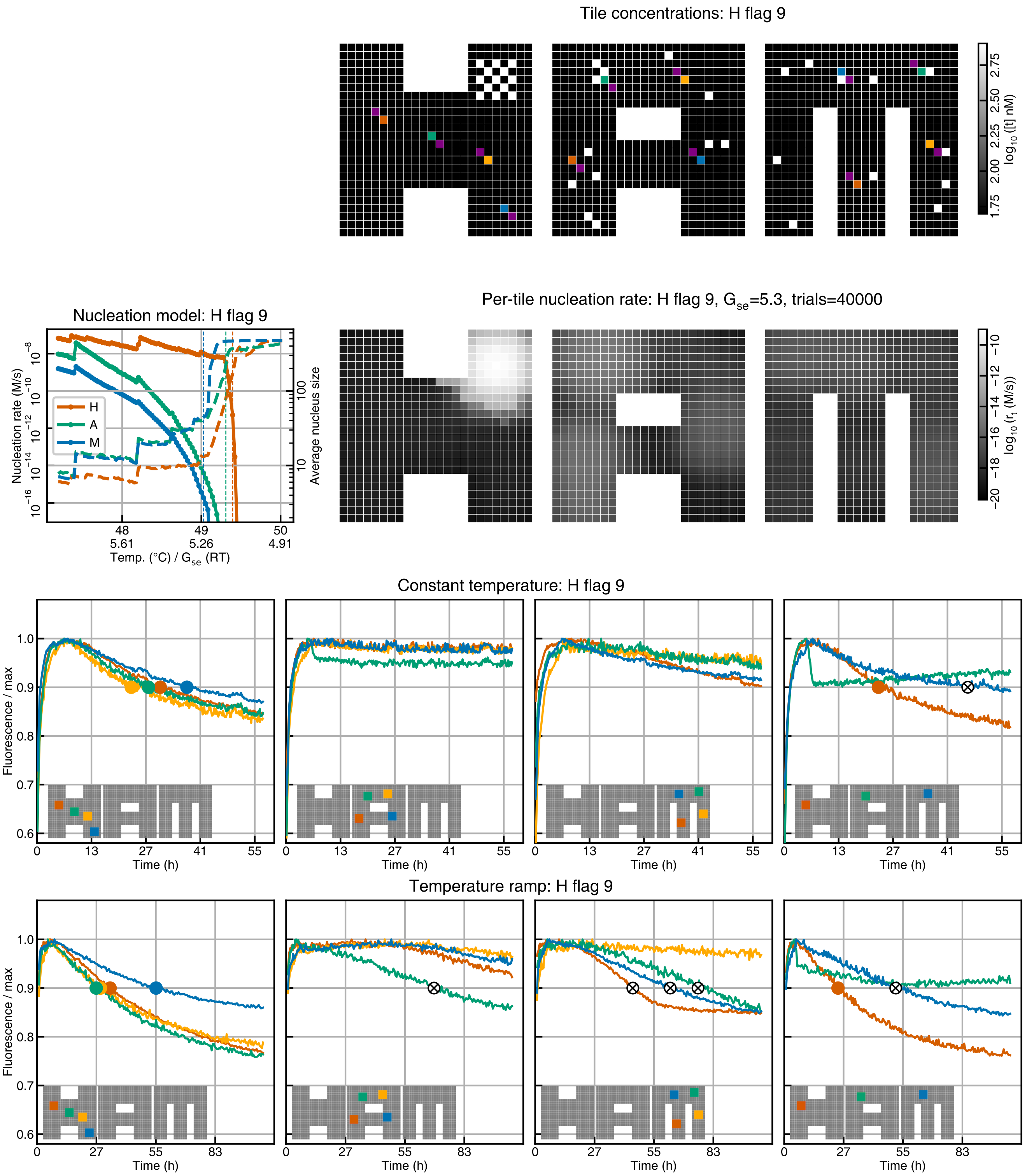
Tile concentrations: H flag 9
log$_{10}$([t] nM)
1.75 2.00 2.25 2.50 2.75
Nucleation model: H flag 9
Nucleation rate (M/s)
Average nucleus size
H
A
M
Temp. (°C) / G$_{se}$ (RT)
48 49 50
5.61 5.26 4.91
Per-tile nucleation rate: H flag 9, G$_{se}$=5.3, trials=40000
log$_{10}$ (r$_t$ (M/s))
−20 −18 −16 −14 −12 −10
Constant temperature: H flag 9
Fluorescence / max
Time (h)
0 13 27 41 55
Temperature ramp: H flag 9
Fluorescence / max
Time (h)
0 27 55 83

5.3.10 H flag 10

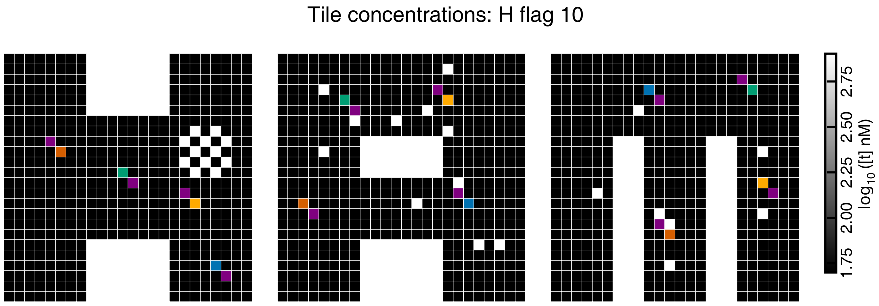

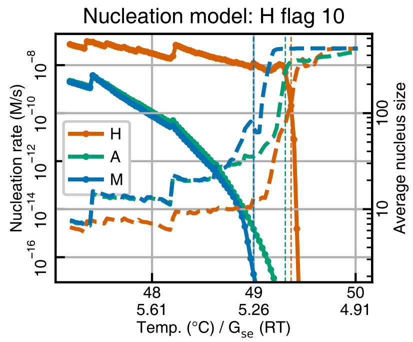

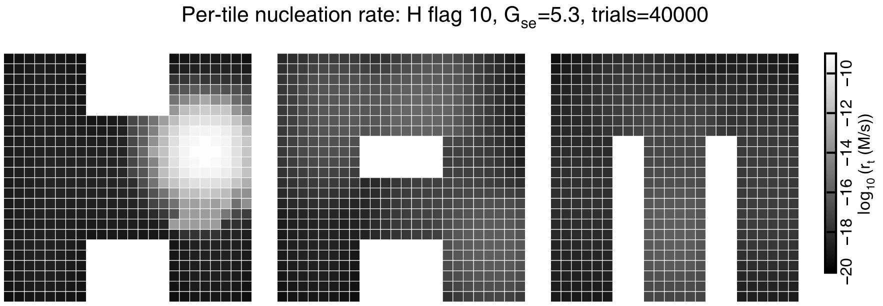

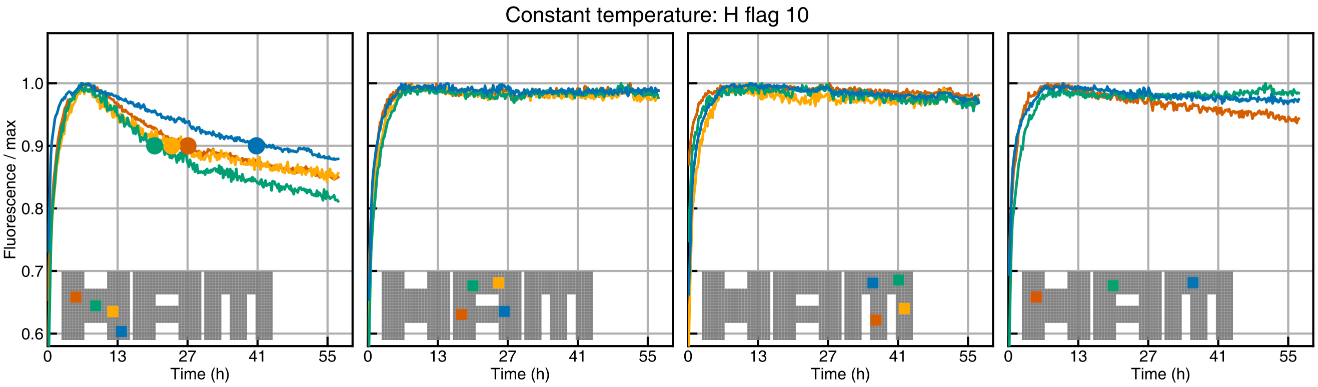

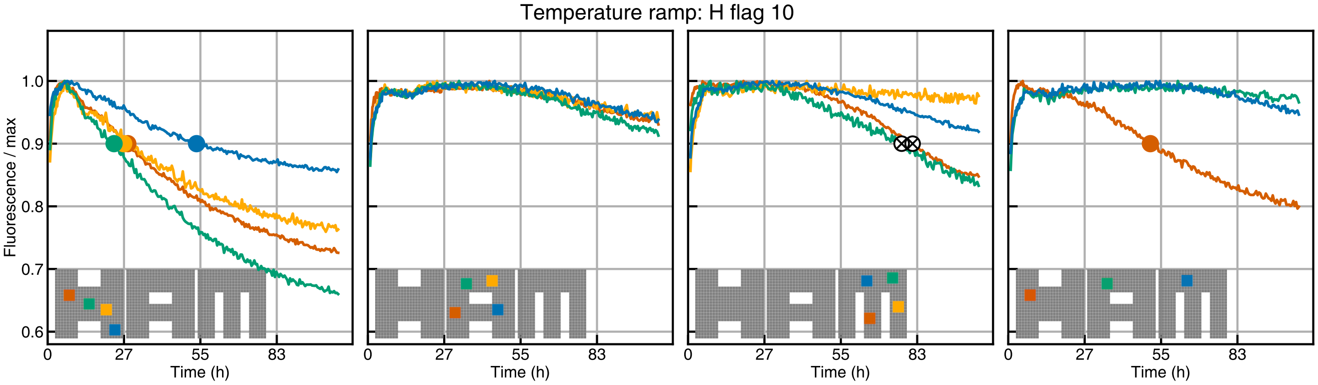

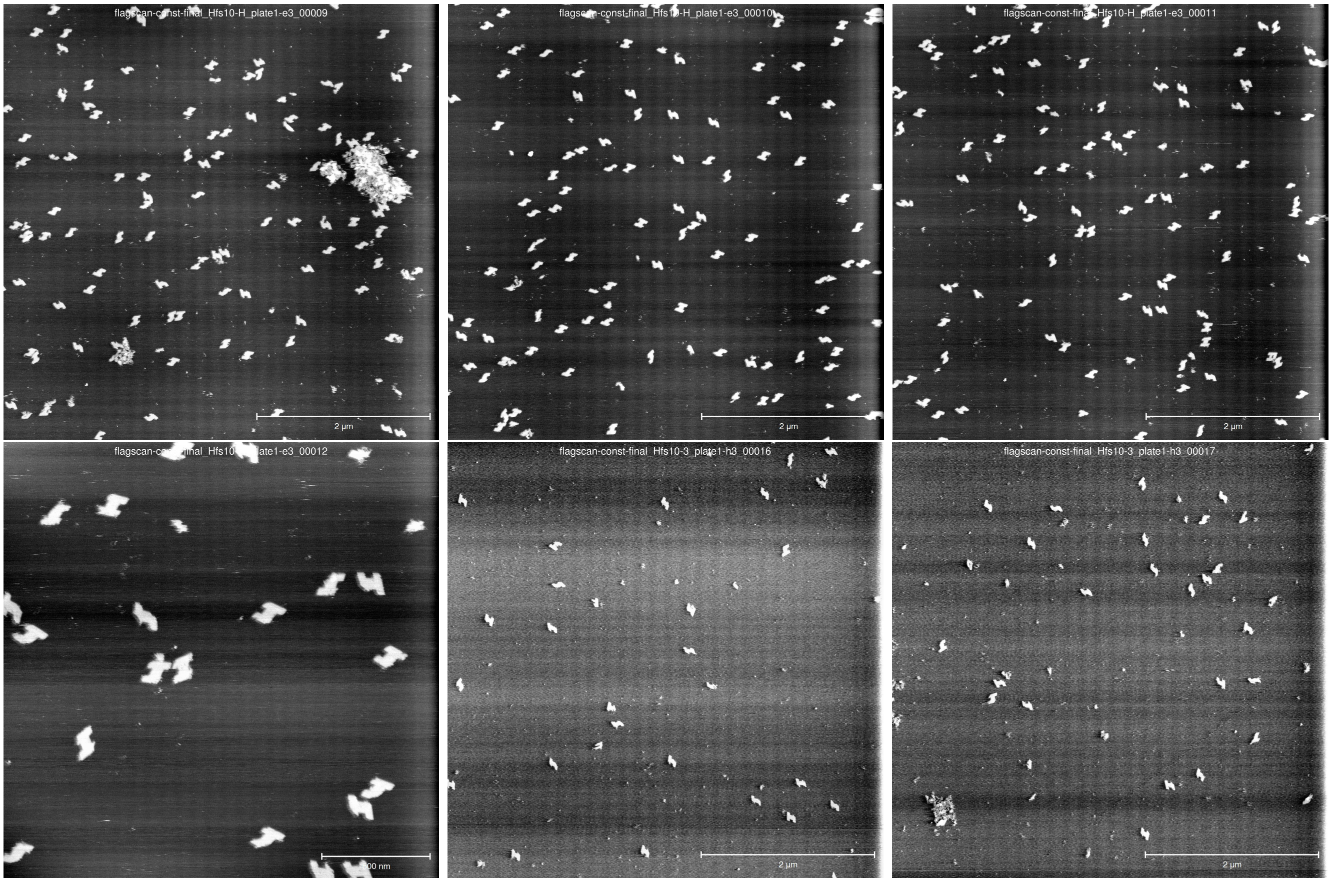
flagscan-const-final_Hfs10-H_plate1-e3_00009
2 µm
2 µm
2 µm
flagscan-const-final_Hfs10-3_plate1-h3_00016
2 µm
flagscan-const-final_Hfs10-3_plate1-h3_00017
2 µm

5.3.11 H flag 11

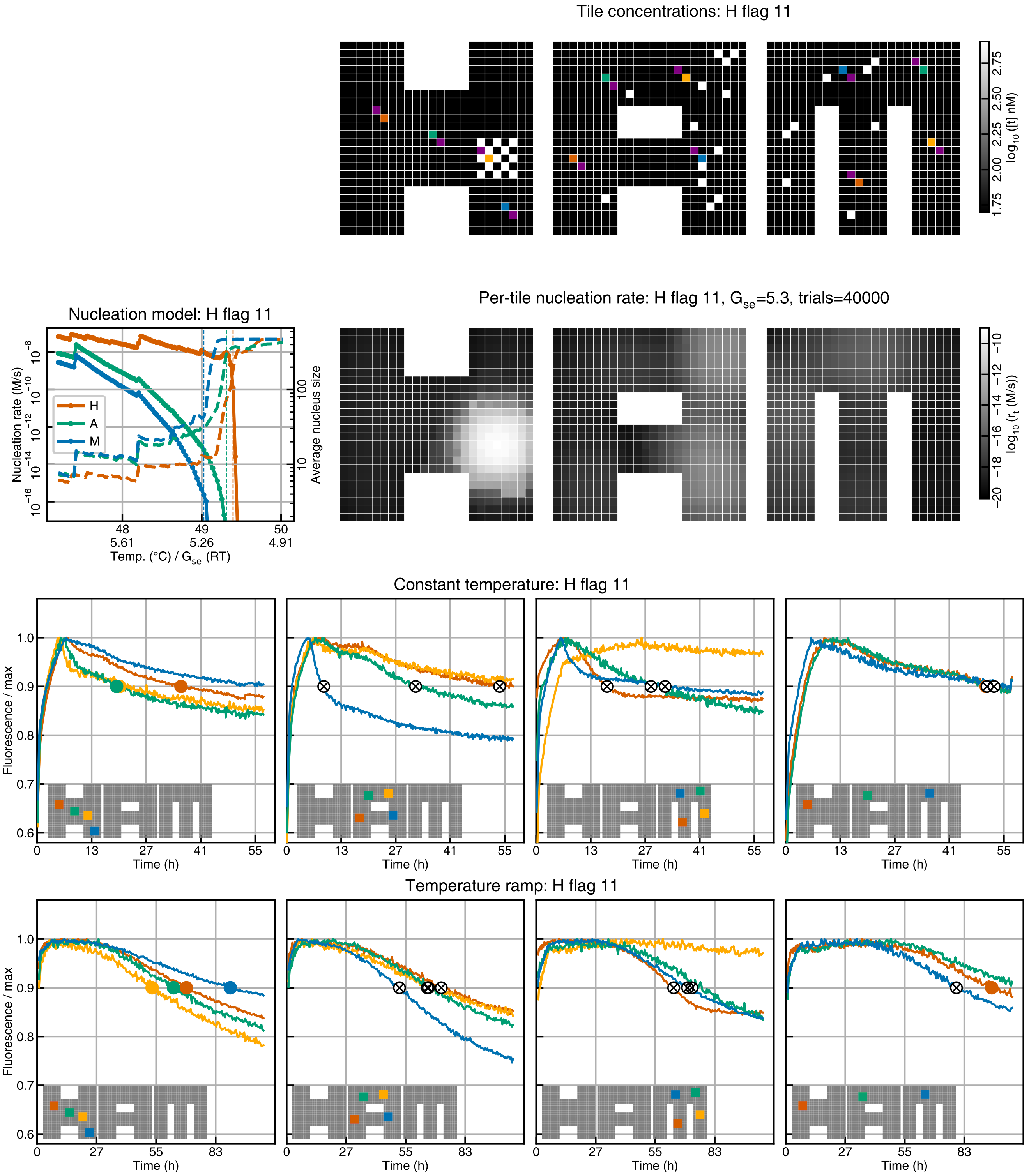

5.3.12 H flag 12

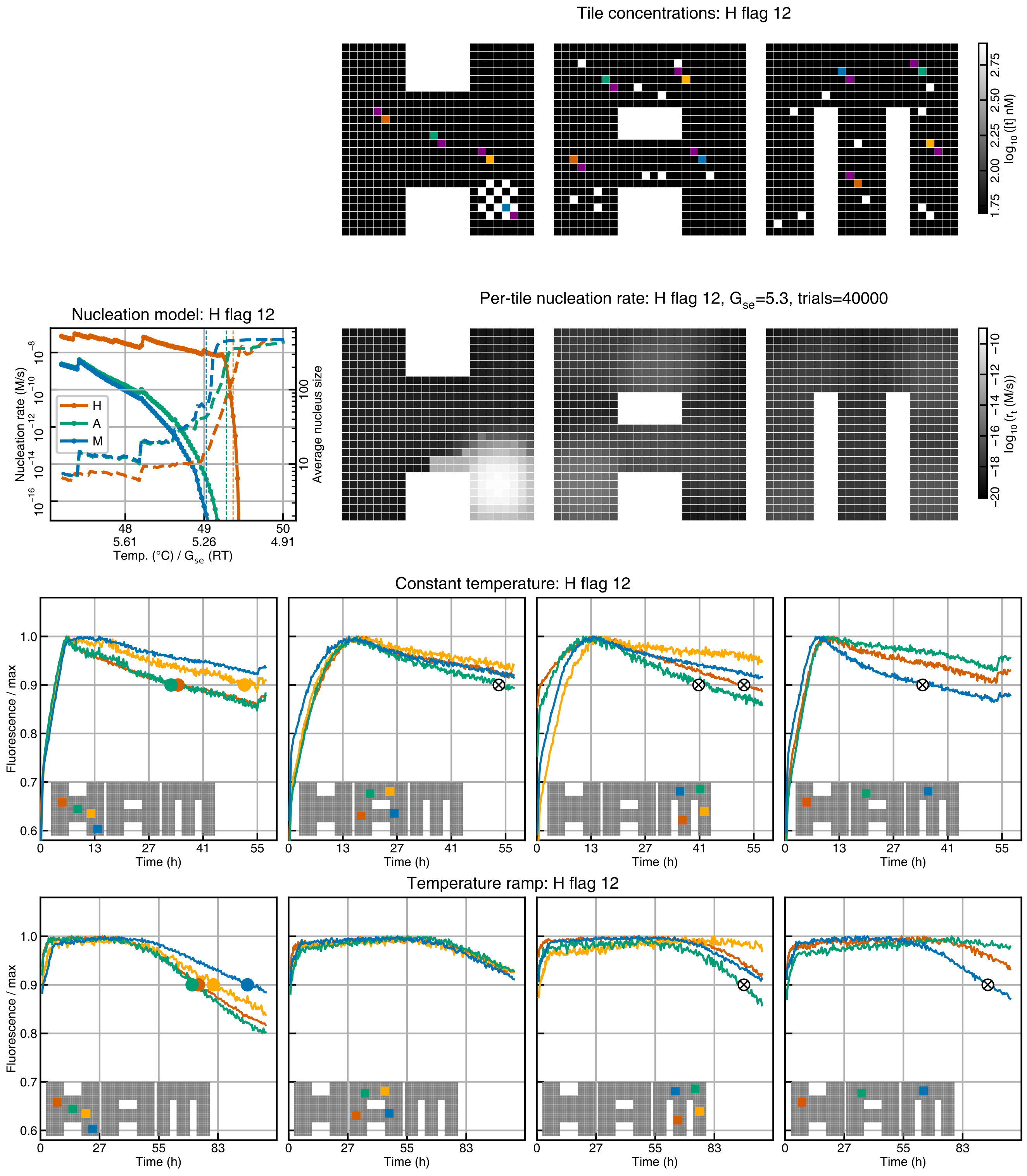

5.3.13 A flag 1

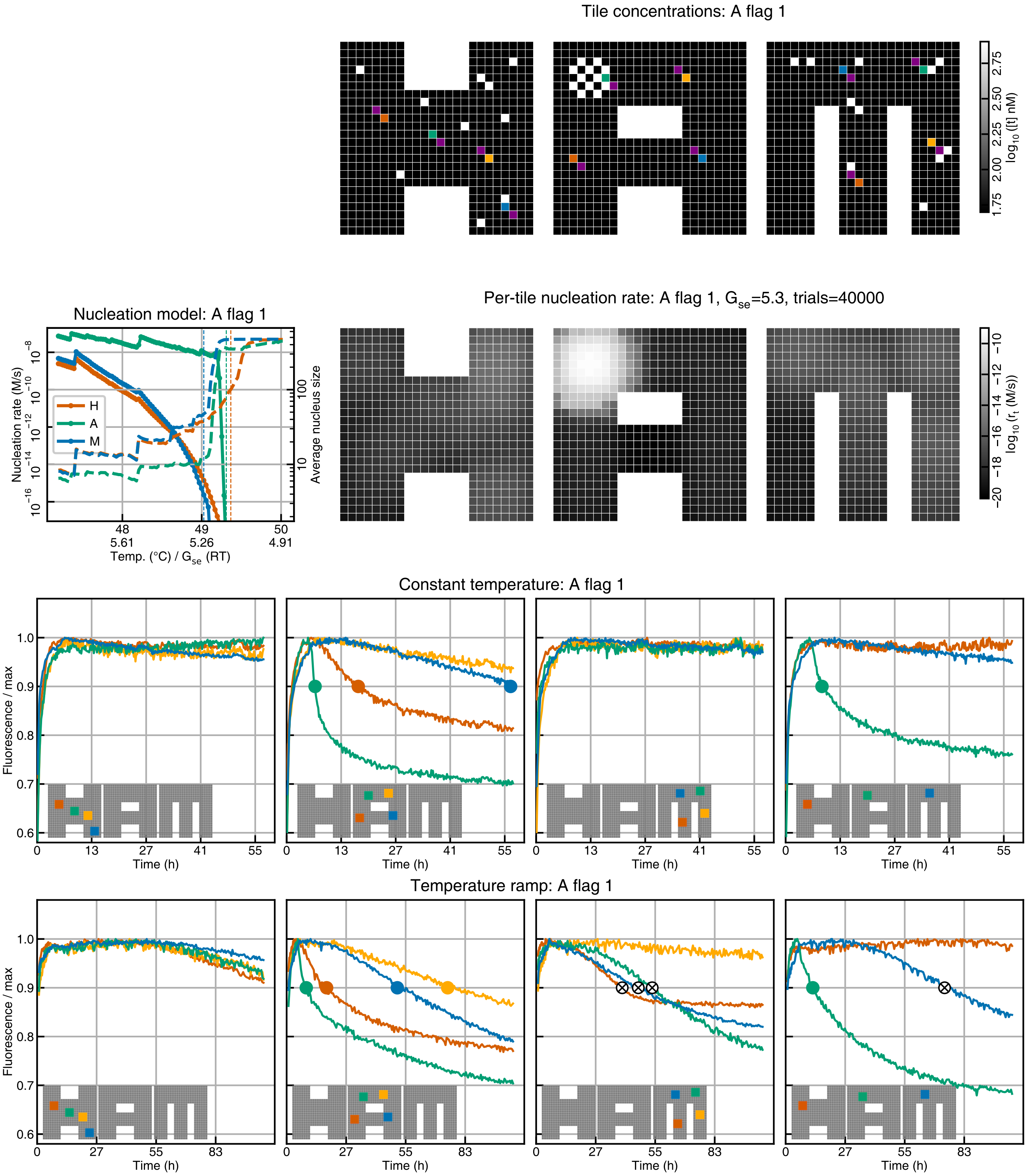
Tile concentrations: A flag 1
$\log_{10}([t]$ nM)
1.75
2.00
2.25
2.50
2.75
Nucleation model: A flag 1
Nucleation rate (M/s)
10^{-8}
10^{-10}
10^{-12}
10^{-14}
10^{-16}
H
A
M
Average nucleus size
100
10
48
49
50
5.61
5.26
4.91
Temp. (°C) / G_{se} (RT)
Per-tile nucleation rate: A flag 1, G_{se}=5.3, trials=40000
$\log_{10}(r_t$ (M/s))
−20
−18
−16
−14
−12
−10
Constant temperature: A flag 1
Fluorescence / max
1.0
0.9
0.8
0.7
0.6
0
13
27
41
55
Time (h)
Temperature ramp: A flag 1
Fluorescence / max
1.0
0.9
0.8
0.7
0.6
0
27
55
83
Time (h)

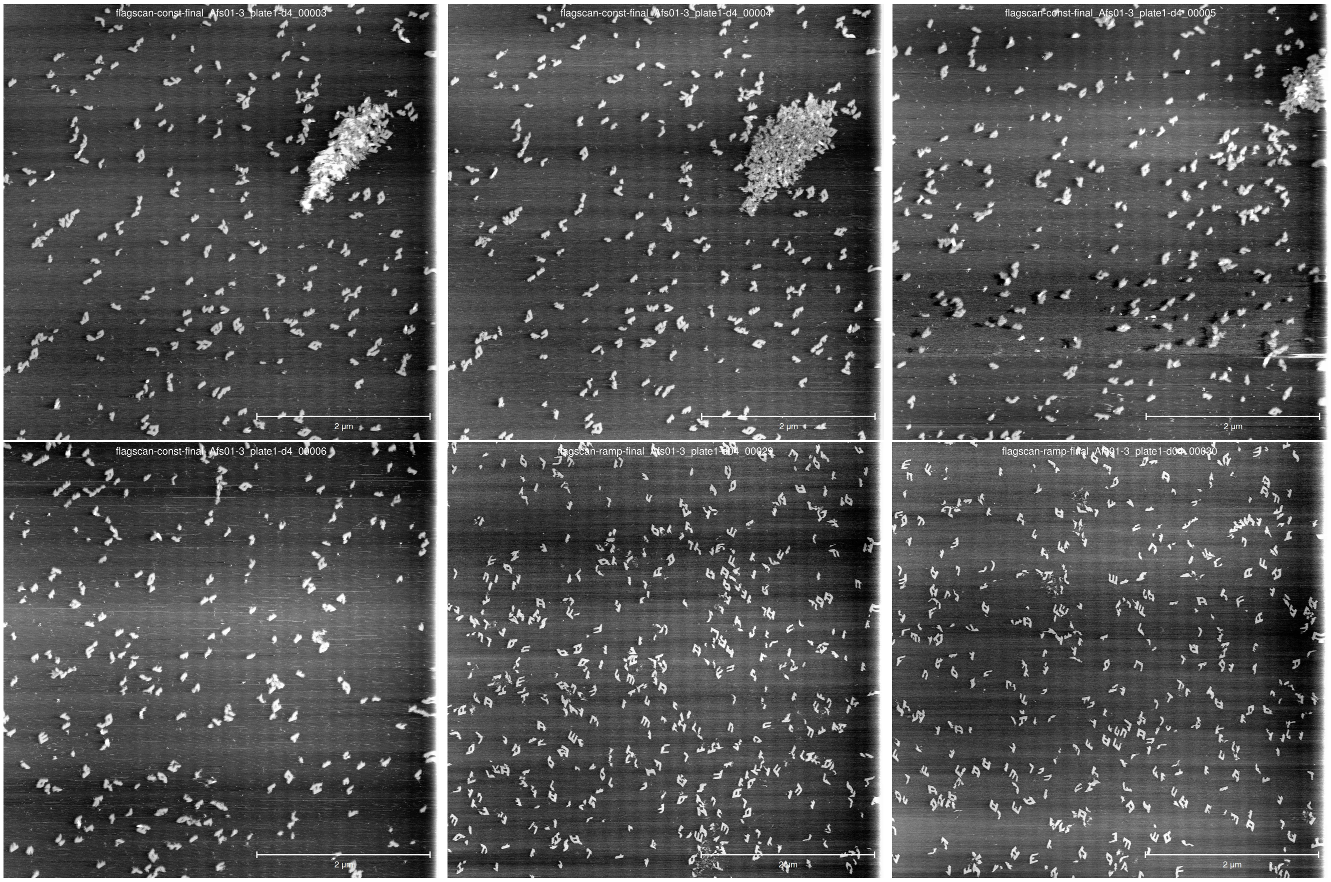
flagscan-const-final_Afs01-3_plate1-d4_00003
flagscan-const-final_Afs01-3_plate1-d4_00004
flagscan-const-final_Afs01-3_plate1-d4_00005
flagscan-const-final_Afs01-3_plate1-d4_00006
2 µm

5.3.14 A flag 2

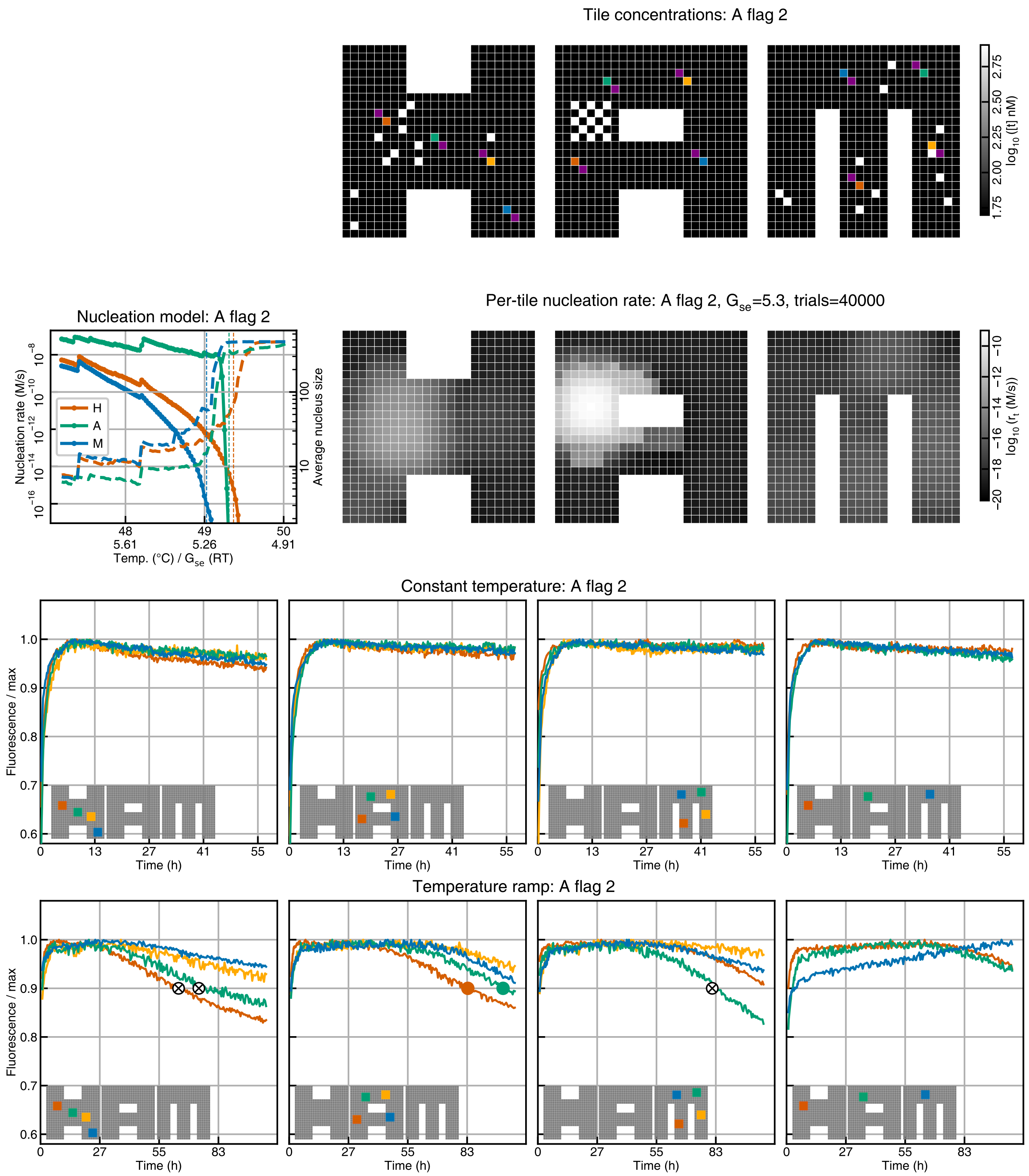
Tile concentrations: A flag 2
$\log_{10}$ ([t] nM)
1.75 2.00 2.25 2.50 2.75
Nucleation model: A flag 2
Nucleation rate (M/s)
Average nucleus size
H
A
M
48 49 50
5.61 5.26 4.91
Temp. (°C) / G_{se} (RT)
Per-tile nucleation rate: A flag 2, G_{se}=5.3, trials=40000
$\log_{10}$ (r_t (M/s))
−20 −18 −16 −14 −12 −10
Constant temperature: A flag 2
Fluorescence / max
Time (h)
0 13 27 41 55
Temperature ramp: A flag 2
Fluorescence / max
Time (h)
0 27 55 83

5.3.15 A flag 3

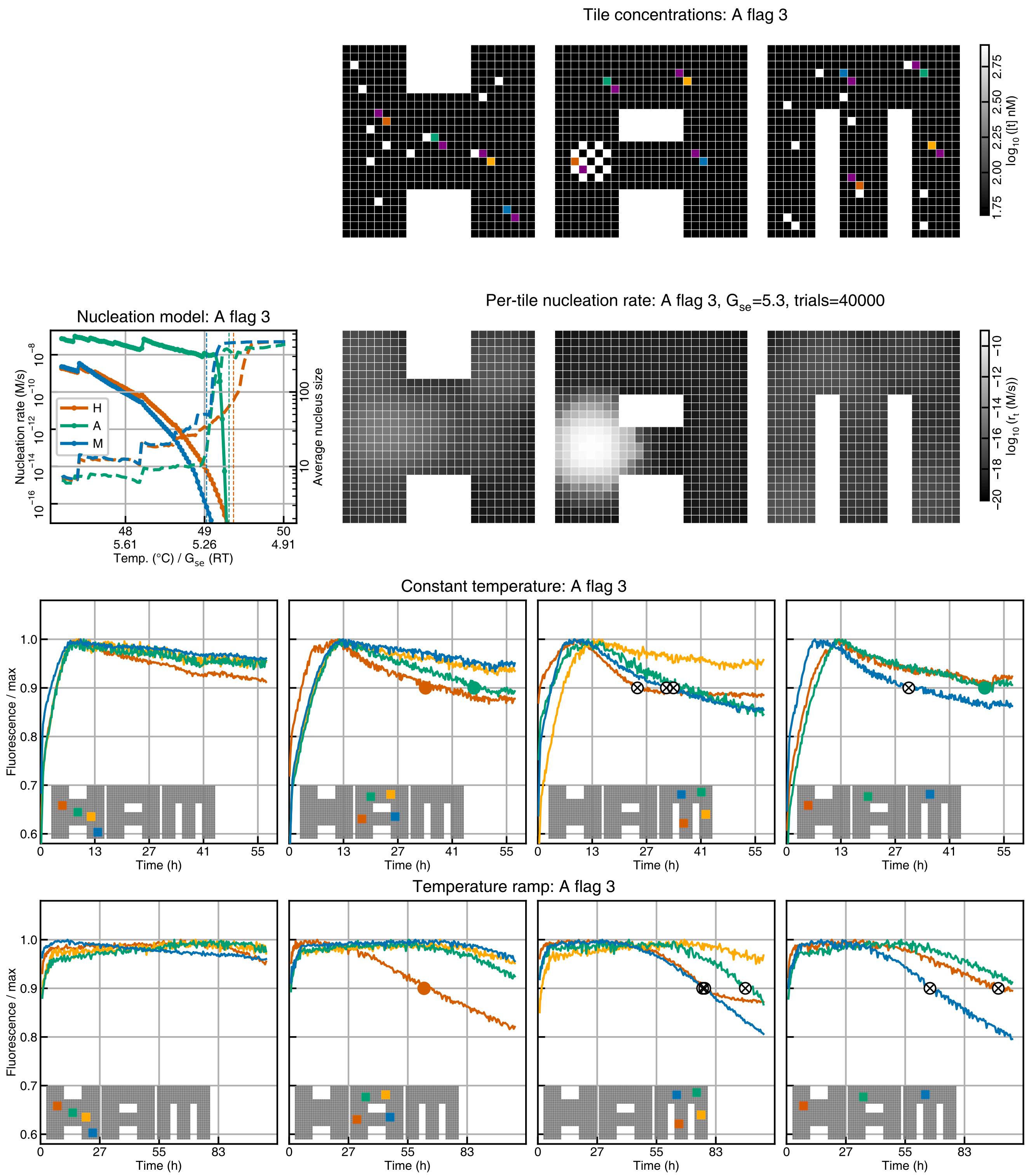

Tile concentrations: A flag 3
$\log_{10}$([t] nM)
1.75 2.00 2.25 2.50 2.75
Nucleation model: A flag 3
Nucleation rate (M/s)
Average nucleus size
H
A
M
Temp. (°C) / G_{se} (RT)
48 49 50
5.61 5.26 4.91
Per-tile nucleation rate: A flag 3, G_{se}=5.3, trials=40000
$\log_{10}$ (r_t (M/s))
−20 −18 −16 −14 −12 −10
Constant temperature: A flag 3
Fluorescence / max
Time (h)
Temperature ramp: A flag 3
Fluorescence / max
Time (h)

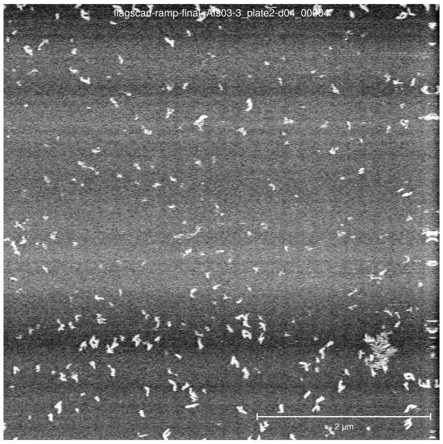
flagscan-ramp-final_Afs03-3_plate2-d04_00004
2 µm

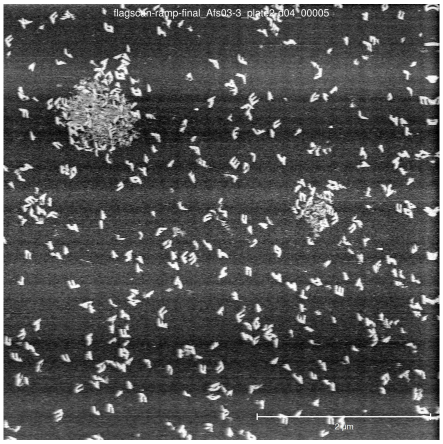
flagscan-ramp-final_Afs03-3_plate2-d04_00005
2 µm

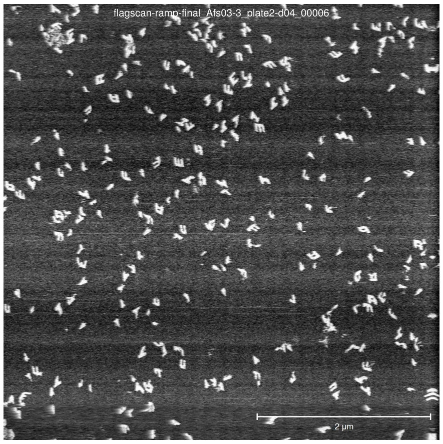
flagscan-ramp-final_Afs03-3_plate2-d04_00006
2 µm

5.3.16 A flag 4

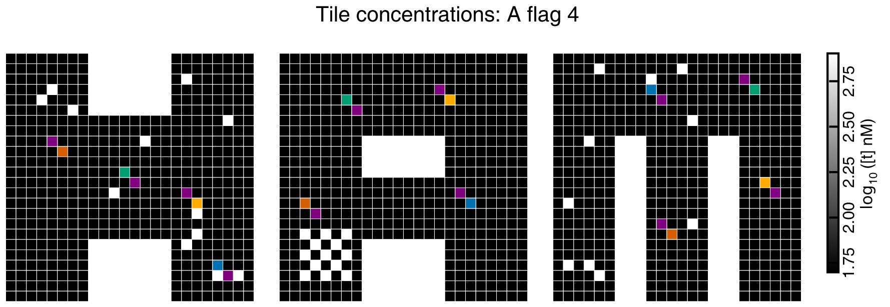
Tile concentrations: A flag 4
$\log_{10}([t]\ nM)$
1.75
2.00
2.25
2.50
2.75

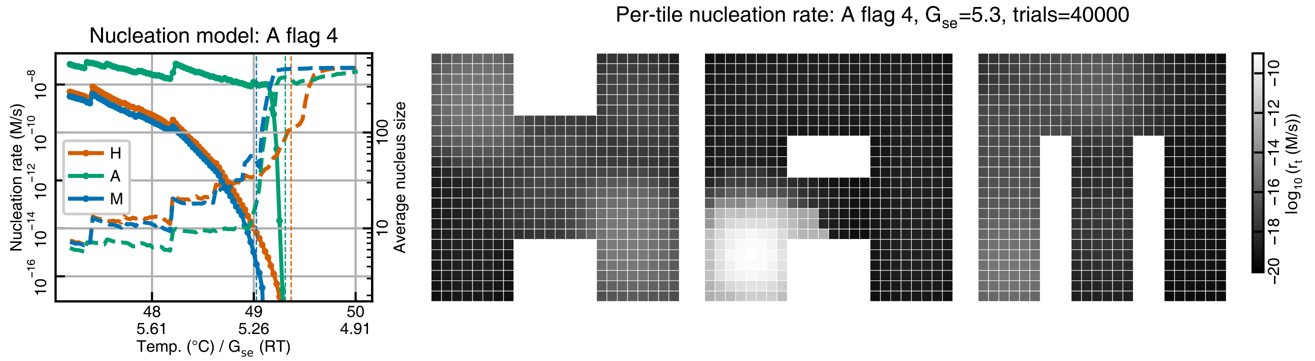
Nucleation model: A flag 4
Nucleation rate (M/s)
Average nucleus size
H
A
M
48
5.61
49
5.26
50
4.91
Temp. (°C) / G_{se} (RT)
Per-tile nucleation rate: A flag 4, G_{se}=5.3, trials=40000
$\log_{10}(r_t\ (M/s))$

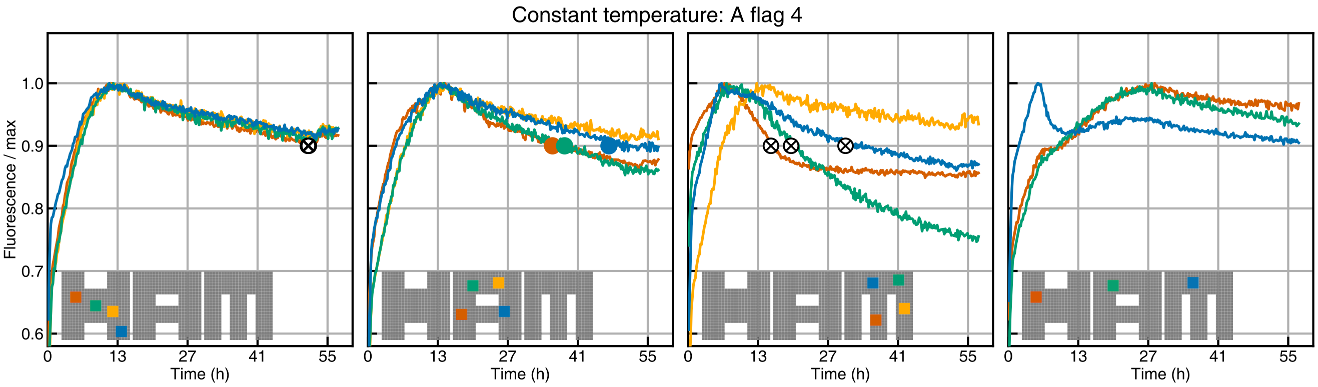
Constant temperature: A flag 4
Fluorescence / max
Time (h)

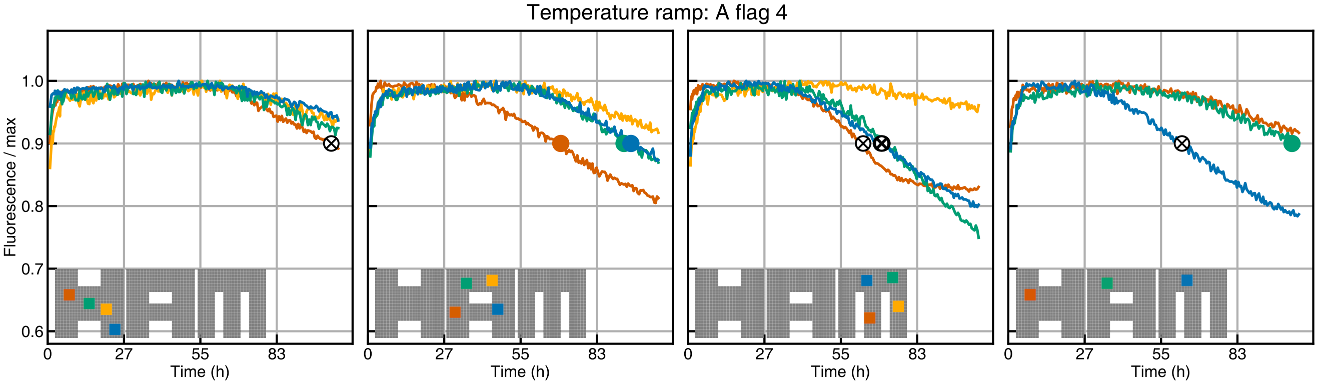
Temperature ramp: A flag 4
Fluorescence / max
Time (h)

5.3.17 A flag 5

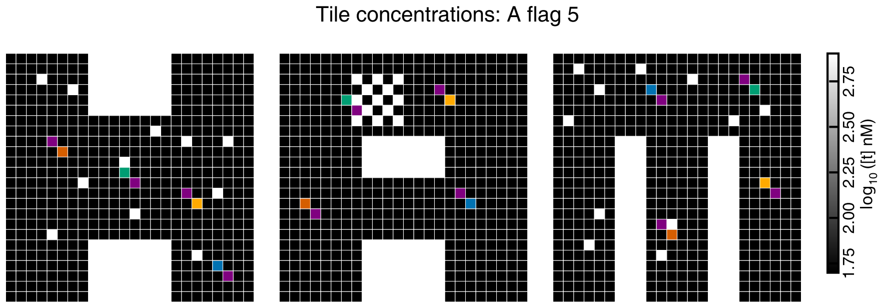
Tile concentrations: A flag 5
1.75 2.00 2.25 2.50 2.75
$\log_{10}$([t] nM)

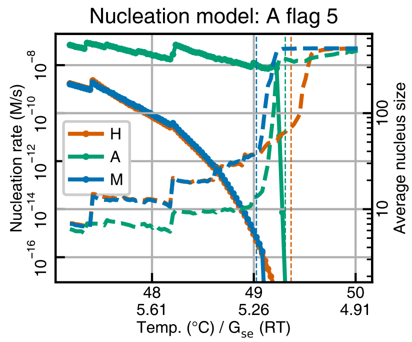
Nucleation model: A flag 5
Nucleation rate (M/s)
Average nucleus size
H
A
M
48 49 50
5.61 5.26 4.91
Temp. (°C) / G_{se} (RT)

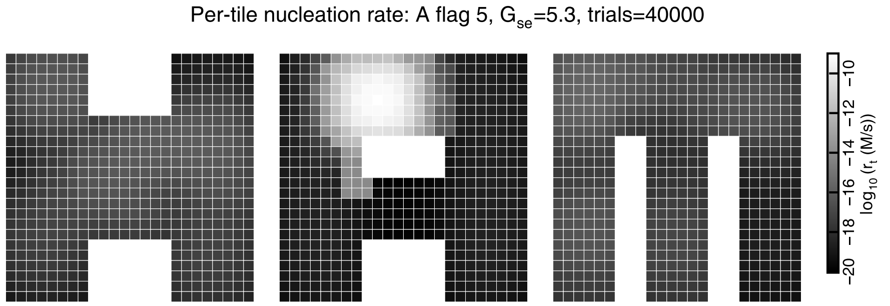
Per-tile nucleation rate: A flag 5, G_{se}=5.3, trials=40000
−20 −18 −16 −14 −12 −10
$\log_{10}$ (r_t (M/s))

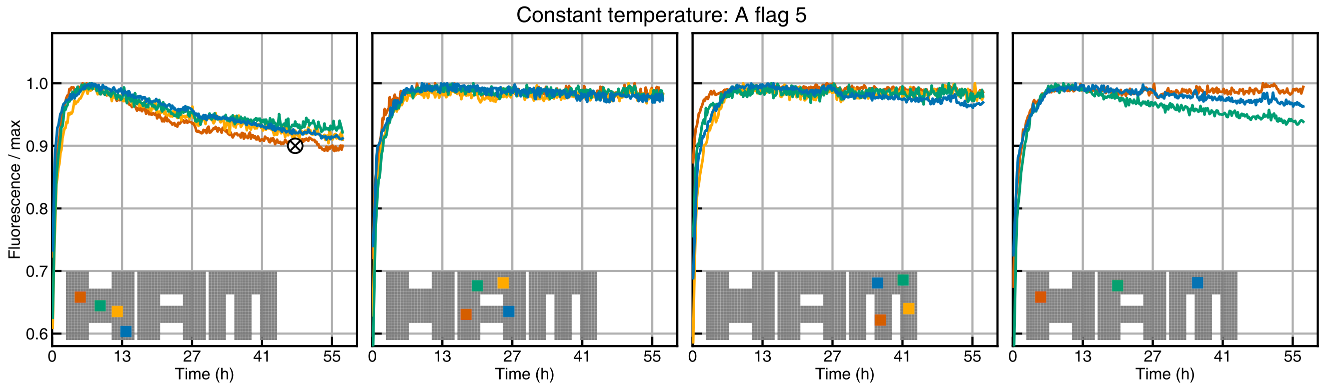
Constant temperature: A flag 5
Fluorescence / max
Time (h)

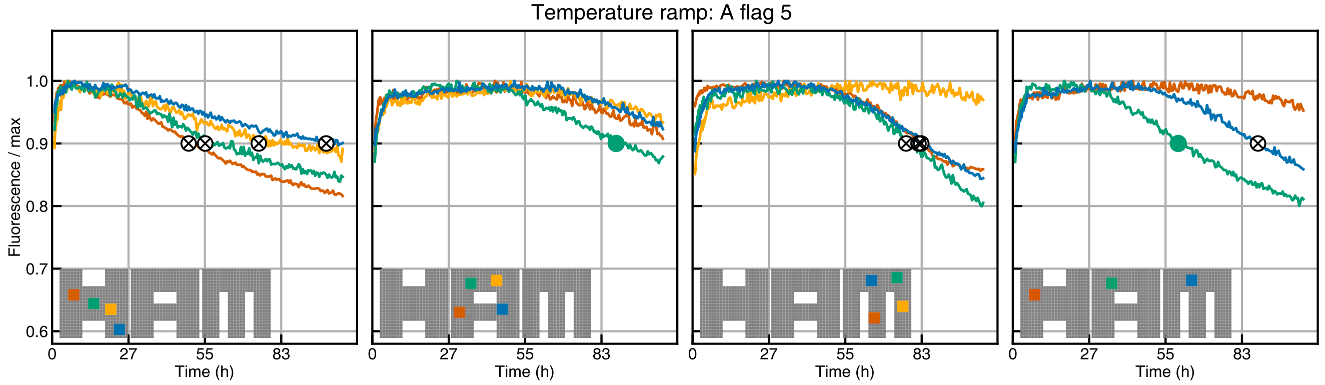
Temperature ramp: A flag 5
Fluorescence / max
Time (h)

5.3.18 A flag 6

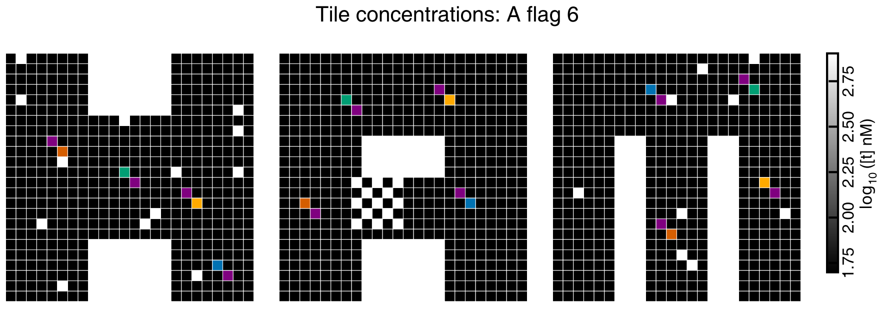
Tile concentrations: A flag 6
log_{10}([t] nM)
1.75
2.00
2.25
2.50
2.75

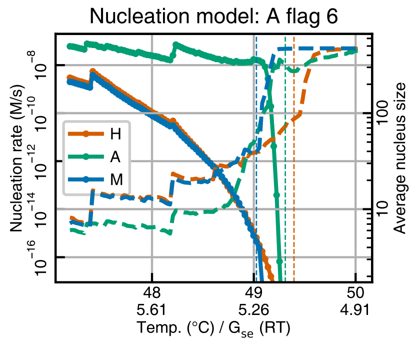
Nucleation model: A flag 6
Nucleation rate (M/s)
Average nucleus size
H
A
M
Temp. (°C) / G_{se} (RT)

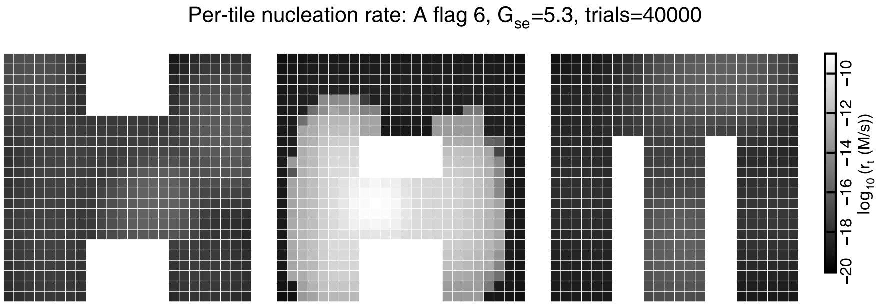
Per-tile nucleation rate: A flag 6, G_{se}=5.3, trials=40000
log_{10} (r_t (M/s))

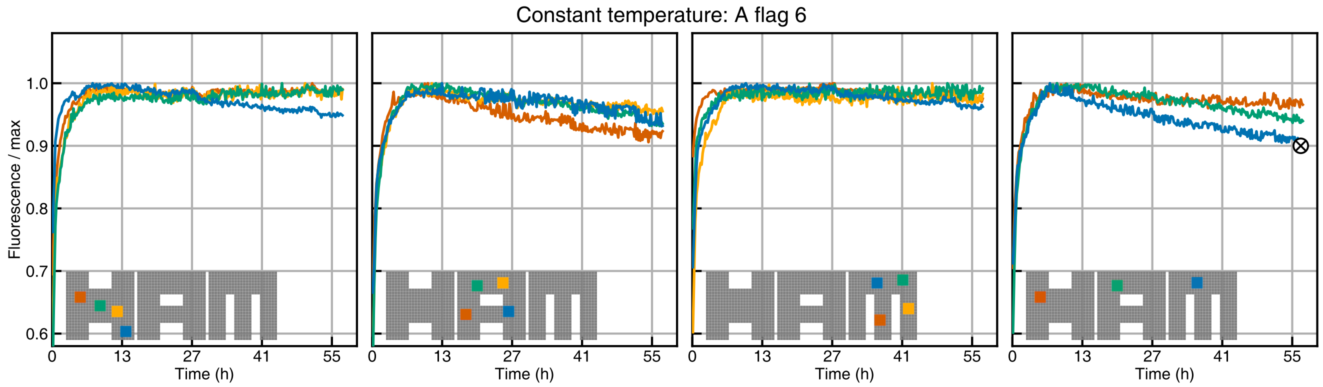
Constant temperature: A flag 6
Fluorescence / max
Time (h)

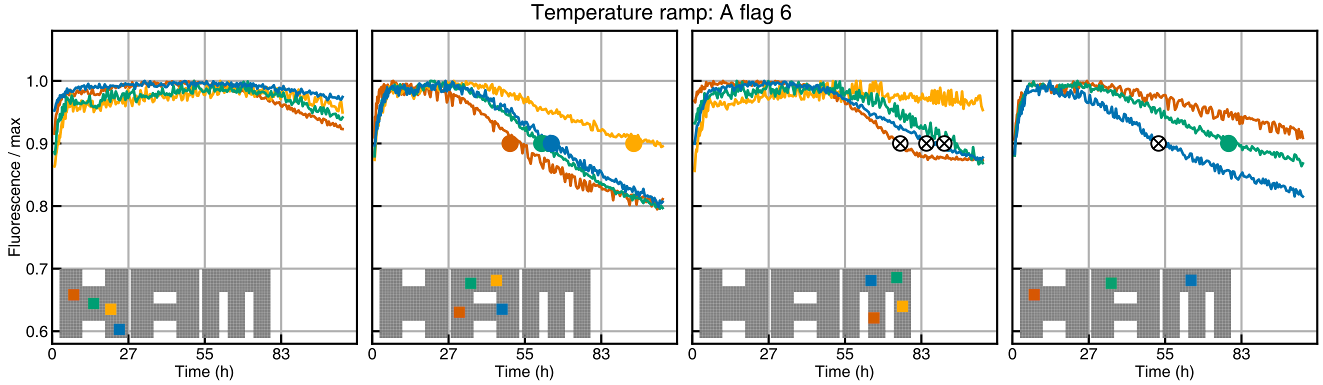
Temperature ramp: A flag 6
Fluorescence / max
Time (h)

5.3.19 A flag 7

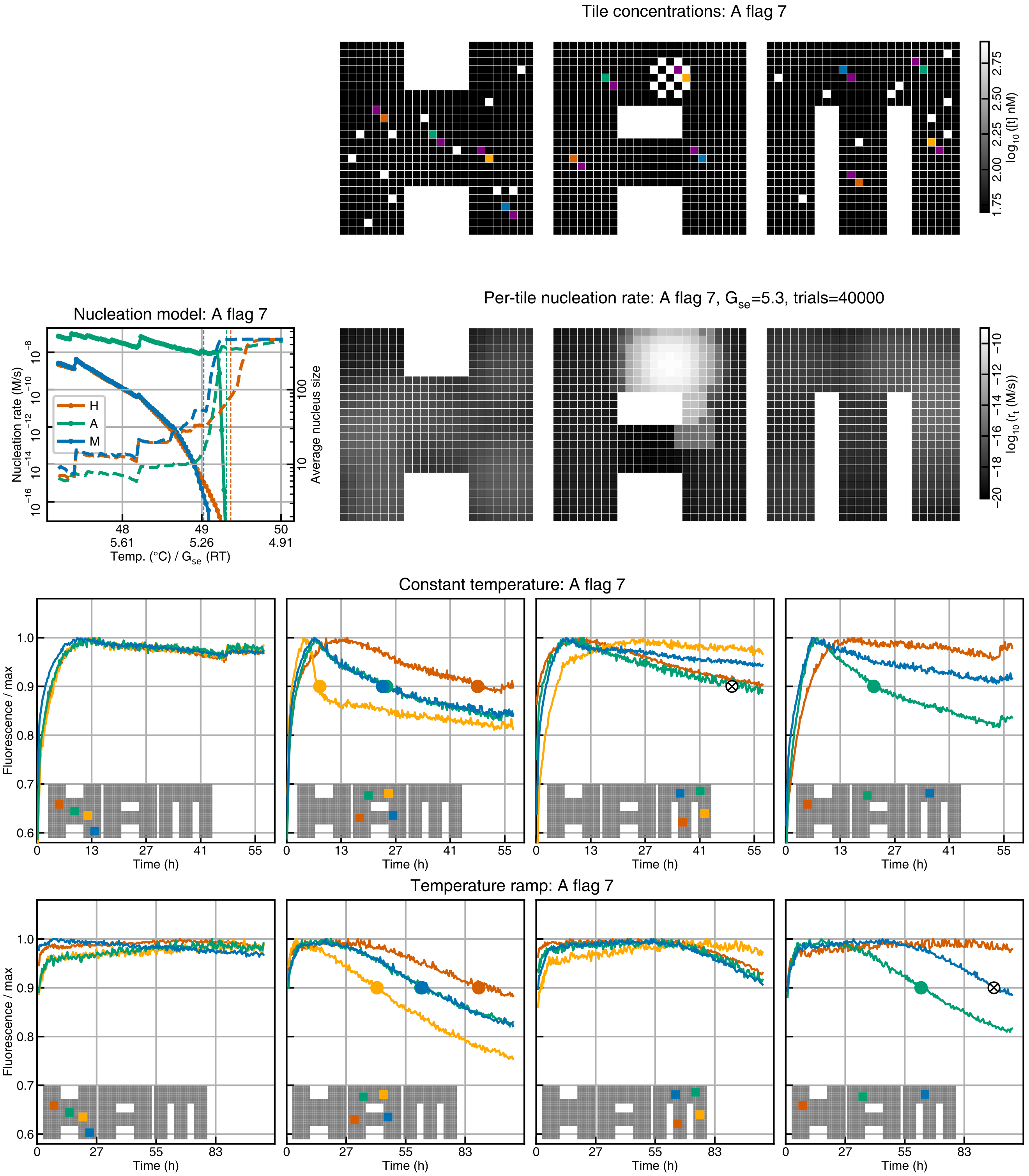
Tile concentrations: A flag 7
log10 ([t] nM)
1.75 2.00 2.25 2.50 2.75
Nucleation model: A flag 7
Nucleation rate (M/s)
Average nucleus size
H
A
M
Temp. (°C) / Gse (RT)
48 49 50
5.61 5.26 4.91
Per-tile nucleation rate: A flag 7, Gse=5.3, trials=40000
log10 (rt (M/s))
−20 −18 −16 −14 −12 −10
Constant temperature: A flag 7
Fluorescence / max
Time (h)
0 13 27 41 55
Temperature ramp: A flag 7
Fluorescence / max
Time (h)
0 27 55 83

5.3.20 A flag 8

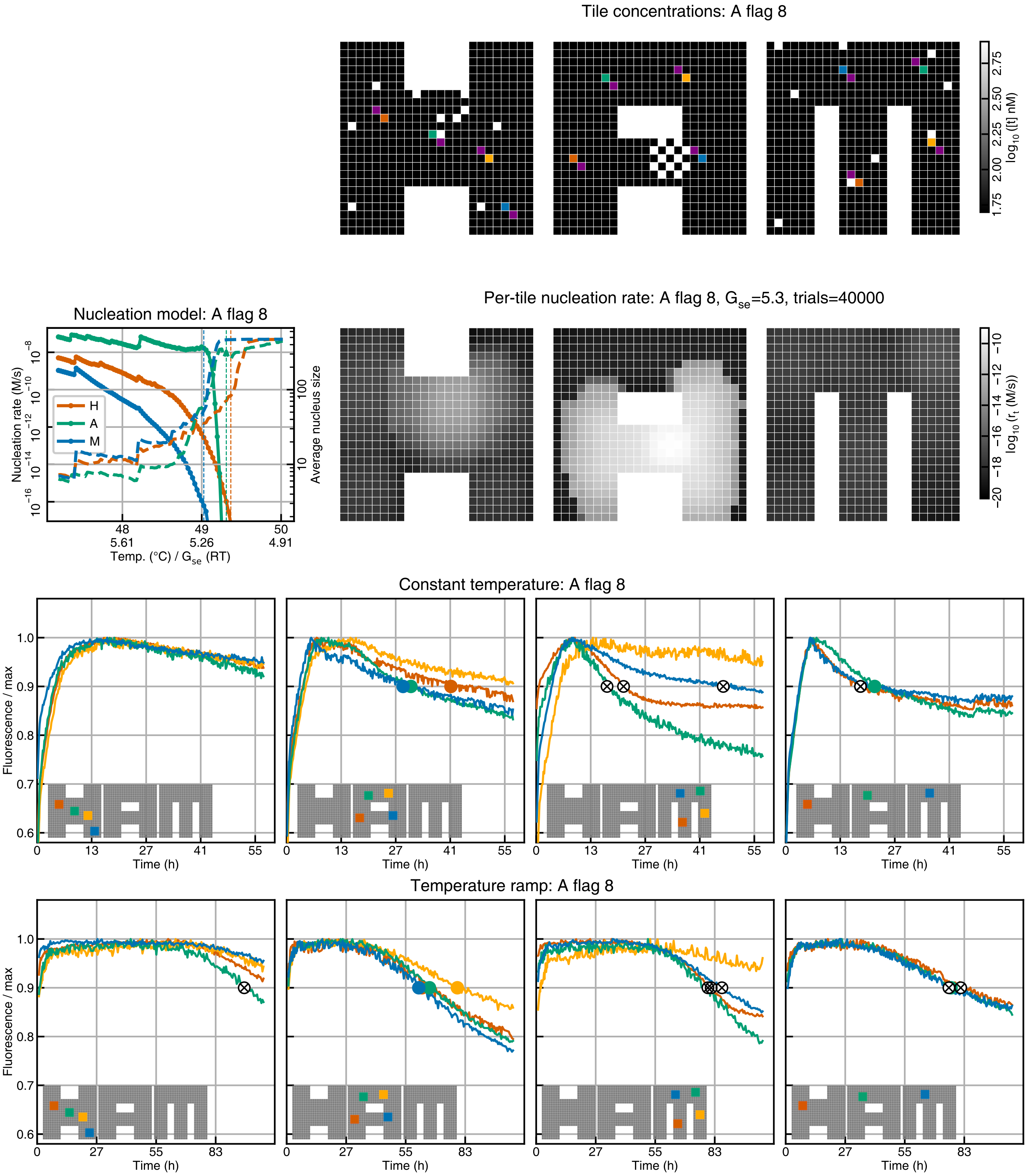
Tile concentrations: A flag 8
$\log_{10}$([t] nM)
Nucleation model: A flag 8
Nucleation rate (M/s)
Average nucleus size
H
A
M
Temp. (°C) / G_{se} (RT)
Per-tile nucleation rate: A flag 8, G_{se}=5.3, trials=40000
$\log_{10}$ (r_t (M/s))
Constant temperature: A flag 8
Fluorescence / max
Time (h)
Temperature ramp: A flag 8
Fluorescence / max
Time (h)

5.3.21 A flag 9

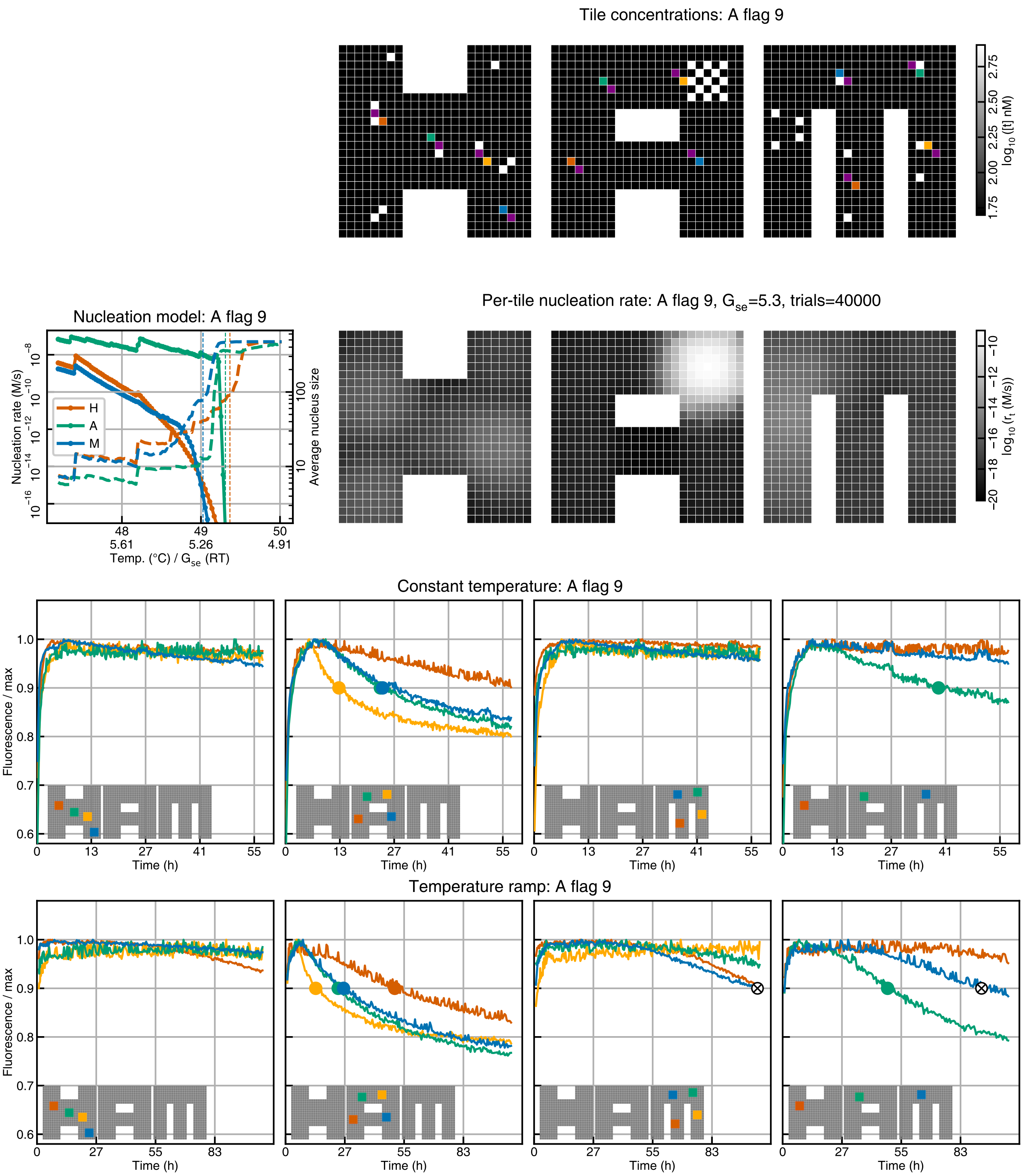
Tile concentrations: A flag 9
log10([t] nM)
1.75 2.00 2.25 2.50 2.75
Nucleation model: A flag 9
Nucleation rate (M/s)
Average nucleus size
H
A
M
Temp. (°C) / Gse (RT)
Per-tile nucleation rate: A flag 9, Gse=5.3, trials=40000
log10 (rt (M/s))
Constant temperature: A flag 9
Fluorescence / max
Time (h)
Temperature ramp: A flag 9

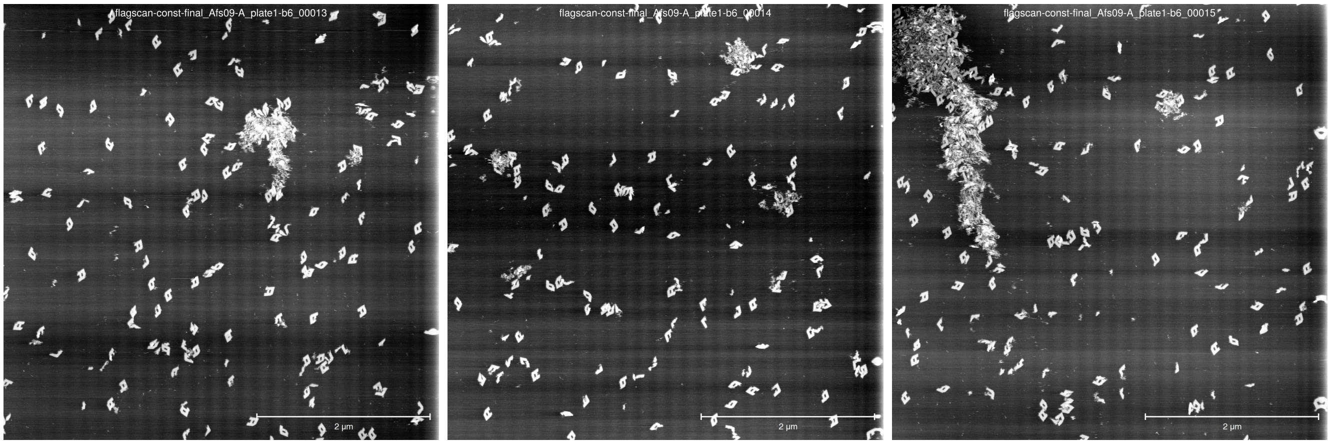
flagscan-const-final_Afs09-A_plate1-b6_00013
2 µm
flagscan-const-final_Afs09-A_plate1-b6_00014
2 µm
flagscan-const-final_Afs09-A_plate1-b6_00015
2 µm

5.3.22 A flag 10

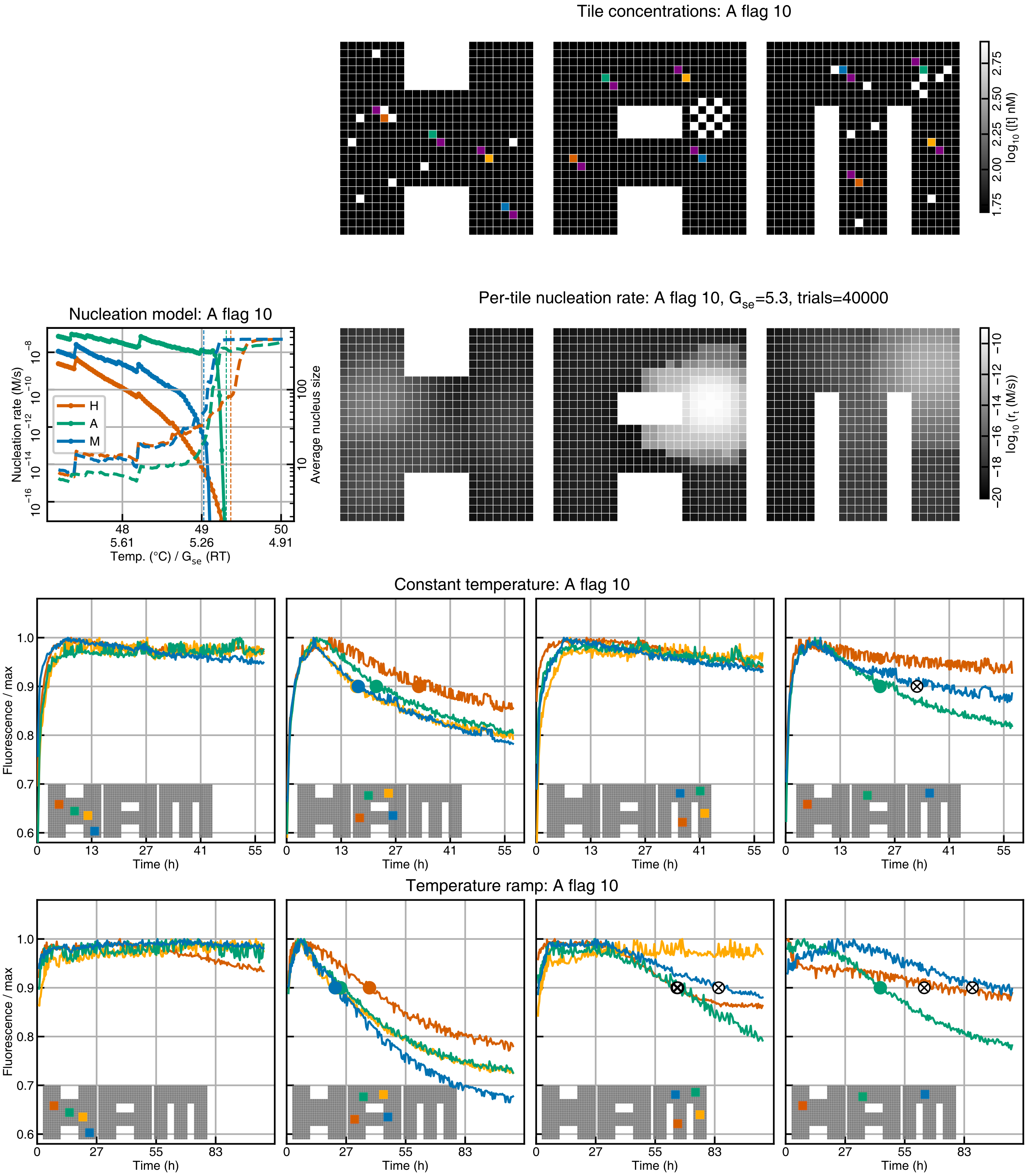

5.3.23 A flag 11

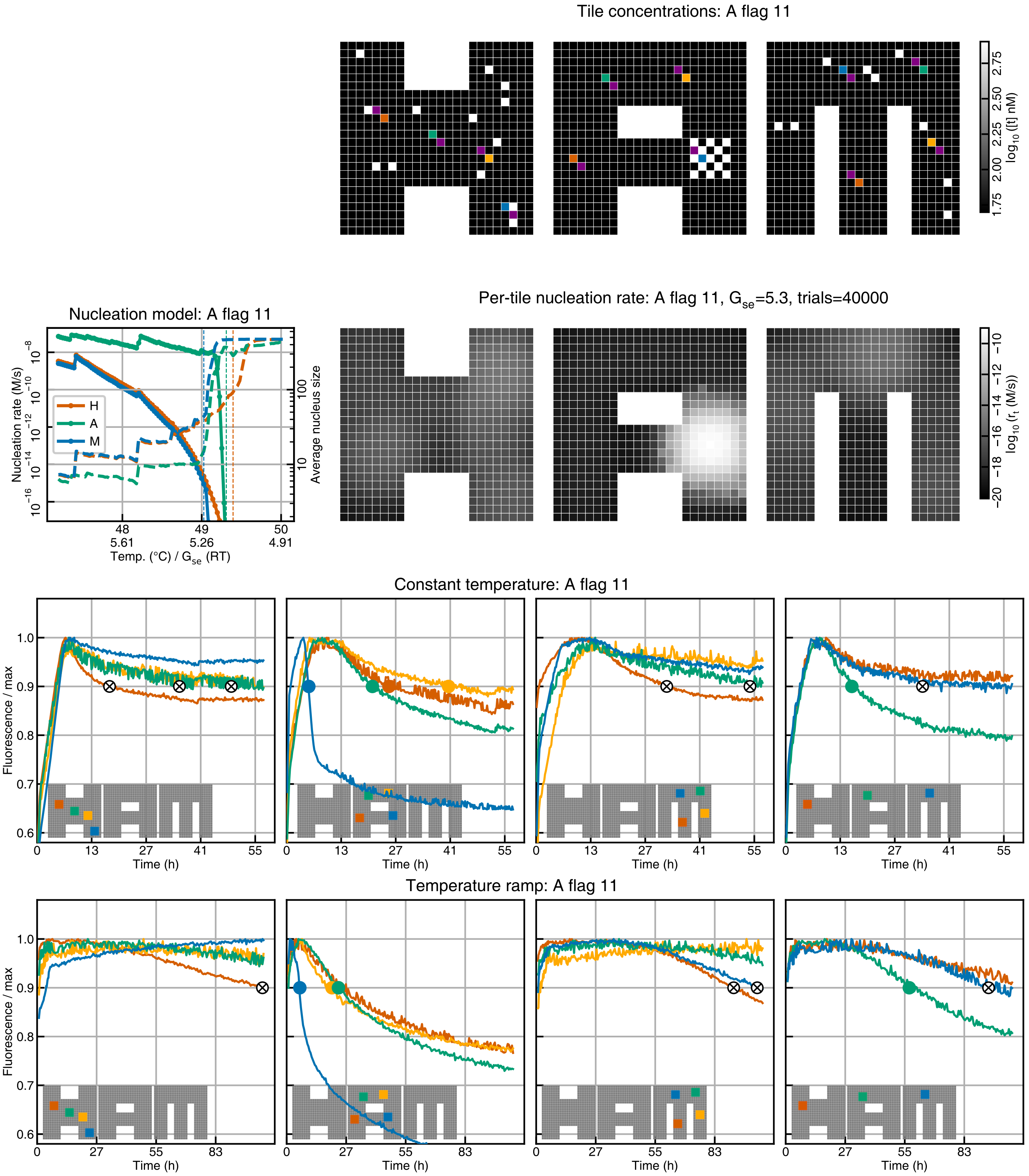

5.3.24 A flag 12

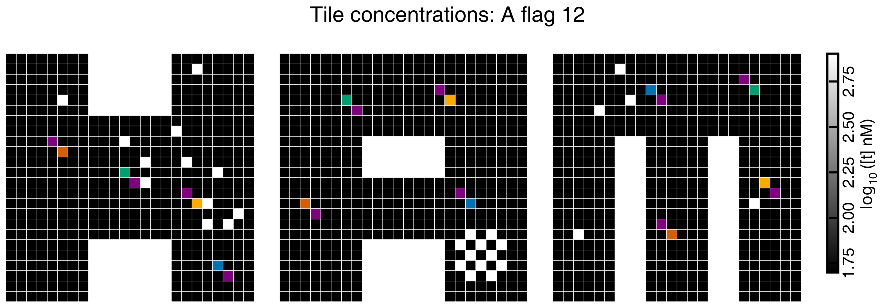

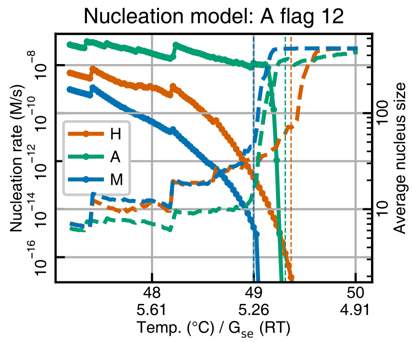

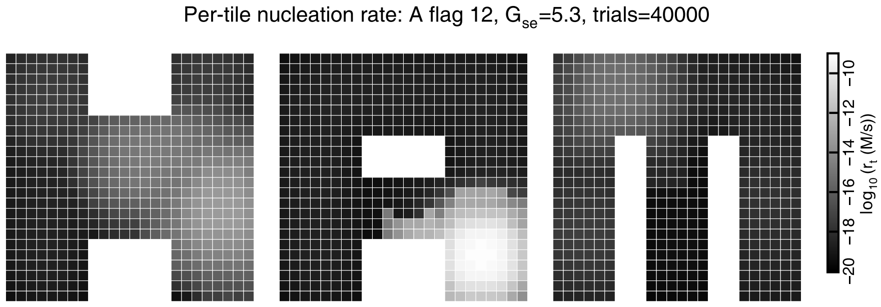

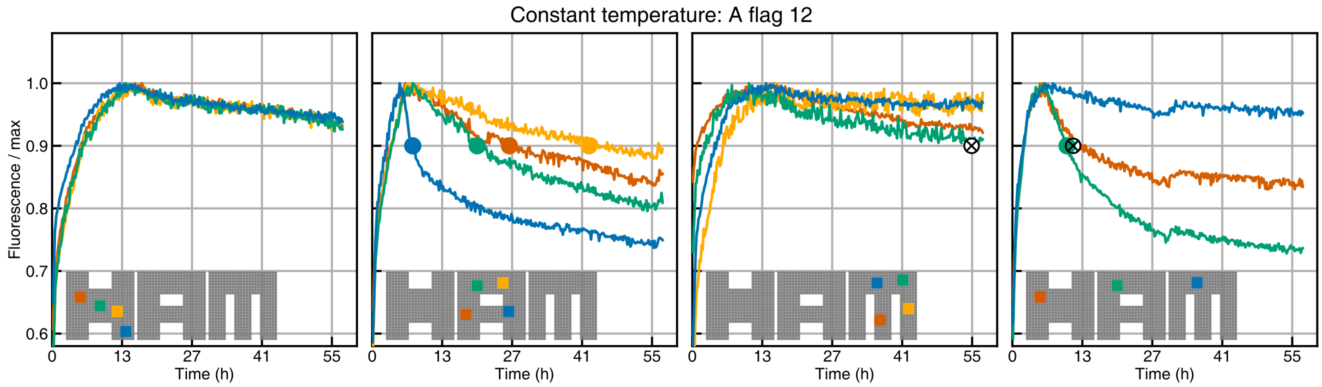

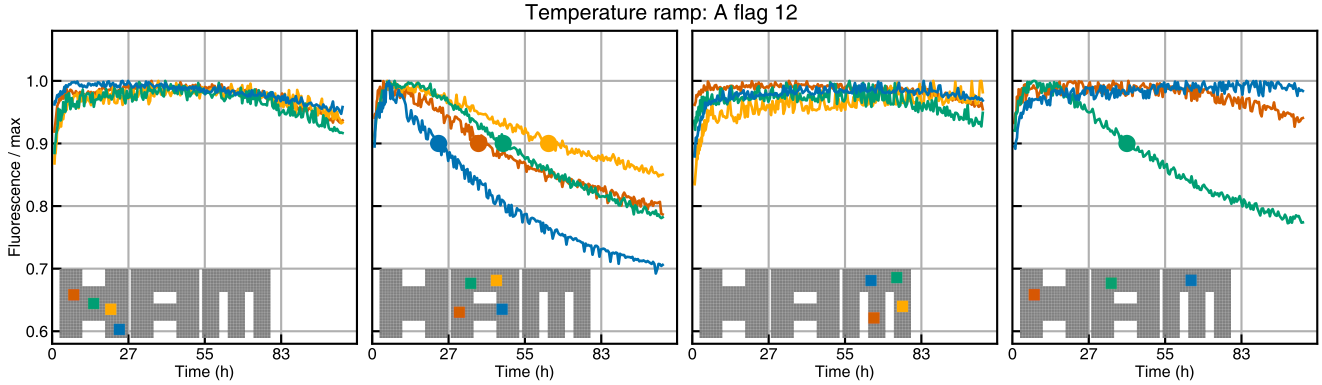

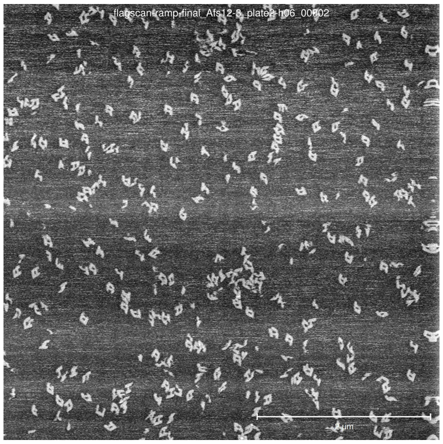
flagscan-ramp-final_Afs12-3_plate2-h06_00002
2 µm

5.3.25 M flag 1

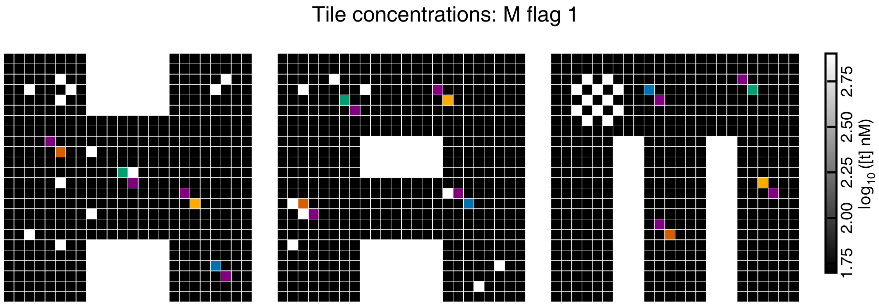
Tile concentrations: M flag 1
1.75
2.00
2.25
2.50
2.75
$\log_{10}$([t] nM)

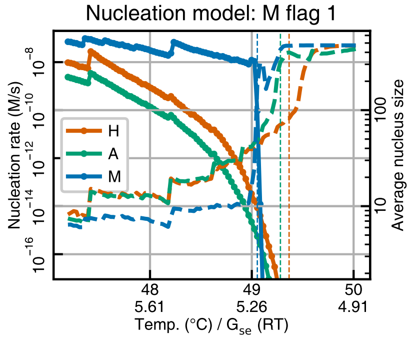
Nucleation model: M flag 1
Nucleation rate (M/s)
Average nucleus size
H
A
M
48
49
50
5.61
5.26
4.91
Temp. (°C) / G_{se} (RT)

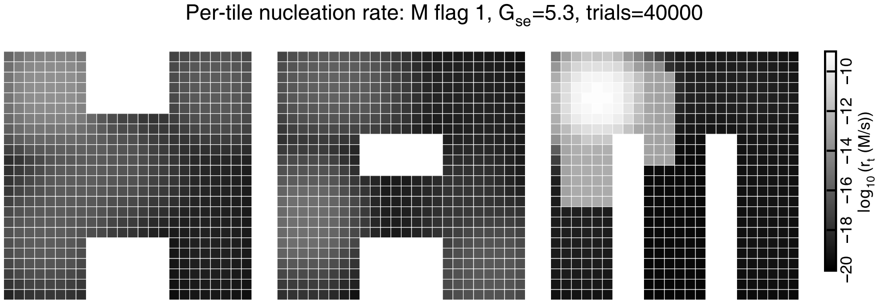
Per-tile nucleation rate: M flag 1, G_{se}=5.3, trials=40000
$\log_{10}$ (r_t (M/s))

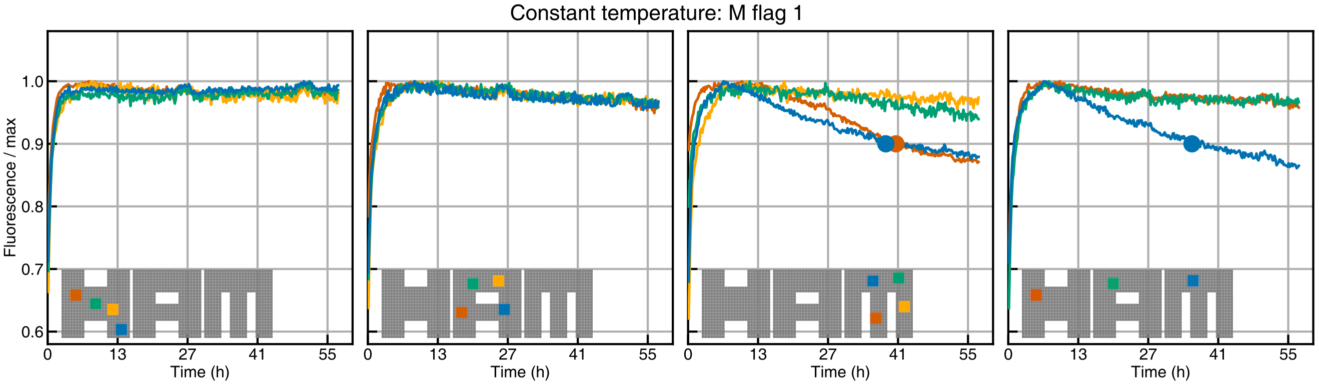
Constant temperature: M flag 1
Fluorescence / max
Time (h)

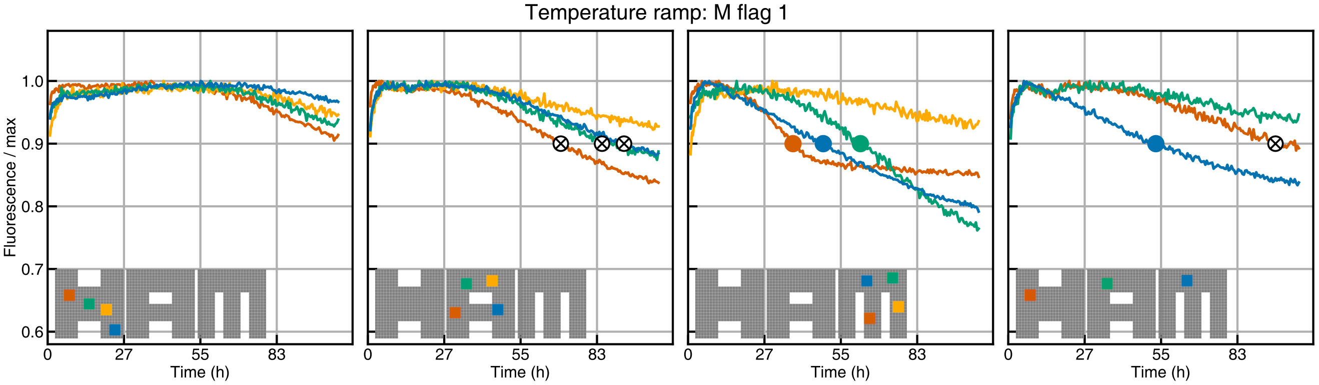
Temperature ramp: M flag 1
Fluorescence / max
Time (h)

5.3.26 M flag 2

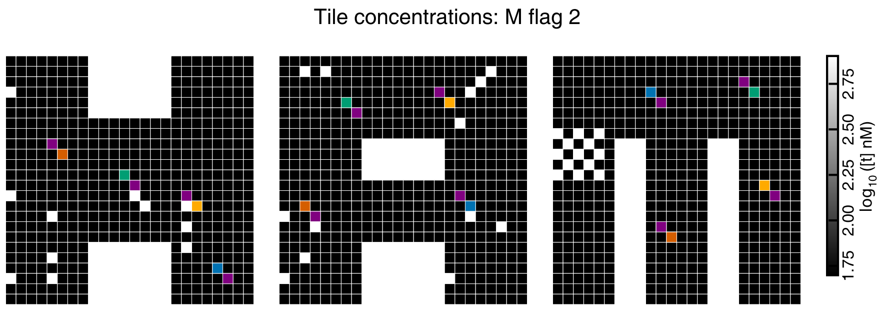
Tile concentrations: M flag 2
1.75 2.00 2.25 2.50 2.75
$\log_{10}$ ([t] nM)

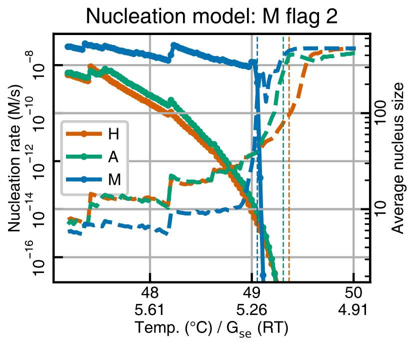
Nucleation model: M flag 2
Nucleation rate (M/s)
10^{-8} 10^{-10} 10^{-12} 10^{-14} 10^{-16}
H
A
M
Average nucleus size
100
10
48 49 50
5.61 5.26 4.91
Temp. (°C) / G_{se} (RT)

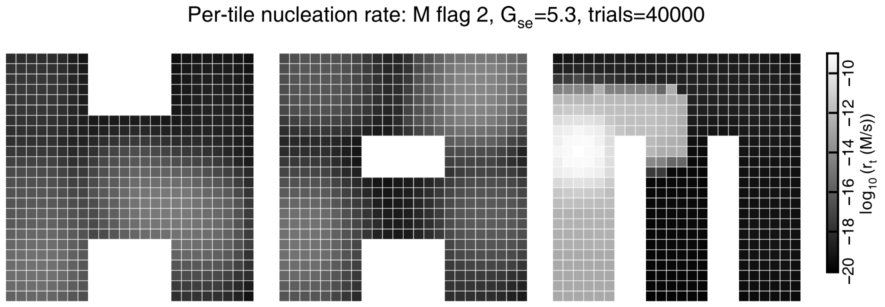
Per-tile nucleation rate: M flag 2, G_{se}=5.3, trials=40000
−20 −18 −16 −14 −12 −10
$\log_{10}$ (r_t (M/s))

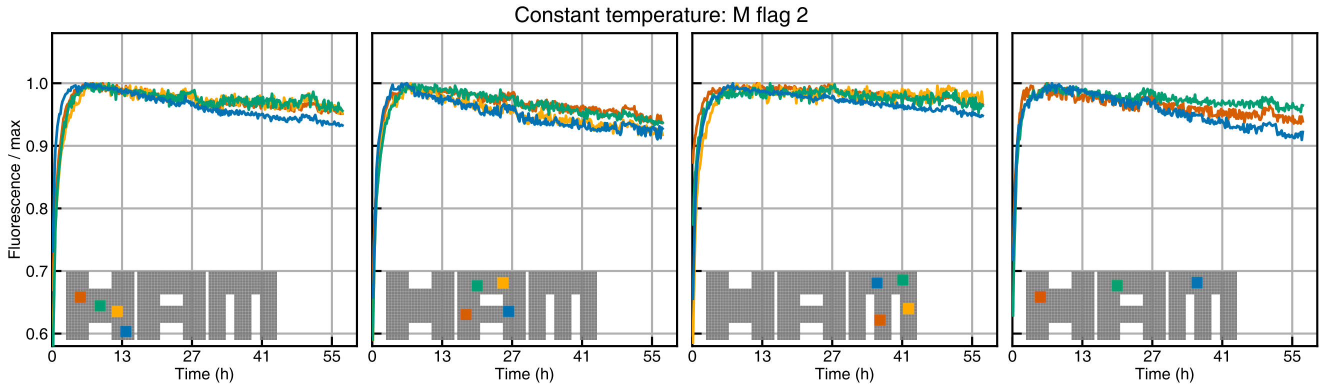
Constant temperature: M flag 2
Fluorescence / max
1.0 0.9 0.8 0.7 0.6
0 13 27 41 55
Time (h)

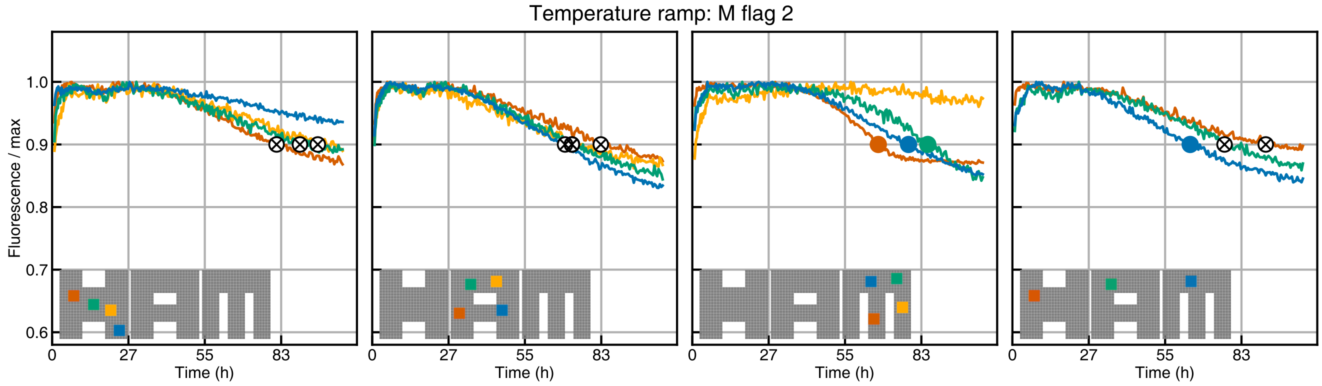
Temperature ramp: M flag 2
Fluorescence / max
1.0 0.9 0.8 0.7 0.6
0 27 55 83
Time (h)

5.3.27 M flag 3

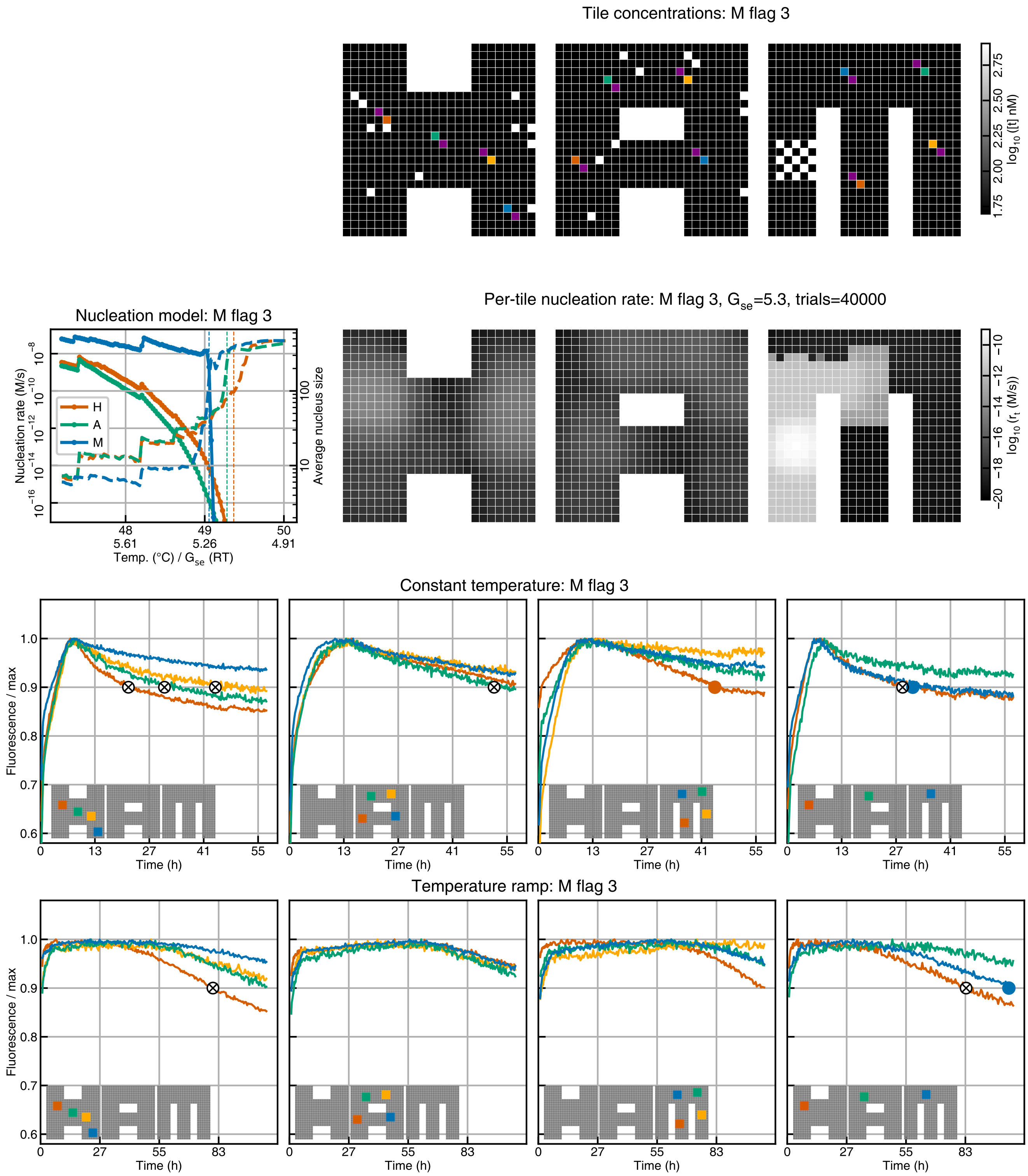
Tile concentrations: M flag 3
log10([t] nM)
1.75 2.00 2.25 2.50 2.75
Nucleation model: M flag 3
Nucleation rate (M/s)
Average nucleus size
H
A
M
48 49 50
5.61 5.26 4.91
Temp. (°C) / Gse (RT)
Per-tile nucleation rate: M flag 3, Gse=5.3, trials=40000
log10 (rt (M/s))
−20 −18 −16 −14 −12 −10
Constant temperature: M flag 3
Fluorescence / max
Time (h)
0 13 27 41 55
Temperature ramp: M flag 3
Fluorescence / max
Time (h)
0 27 55 83

5.3.28 M flag 4

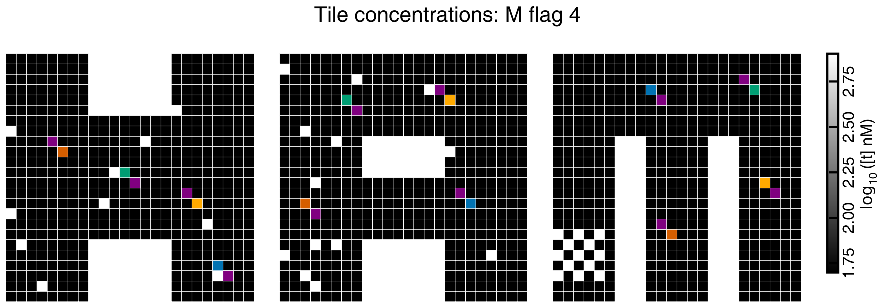
Tile concentrations: M flag 4
$\log_{10}([t]$ nM)
1.75 2.00 2.25 2.50 2.75

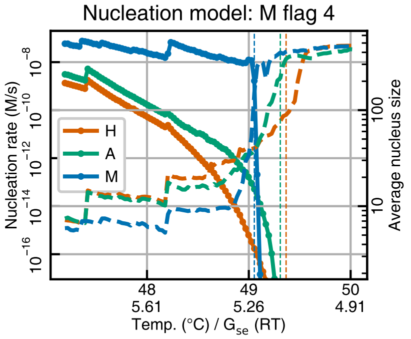
Nucleation model: M flag 4
Nucleation rate (M/s)
Average nucleus size
H
A
M
Temp. (°C) / G_{se} (RT)
48 49 50
5.61 5.26 4.91

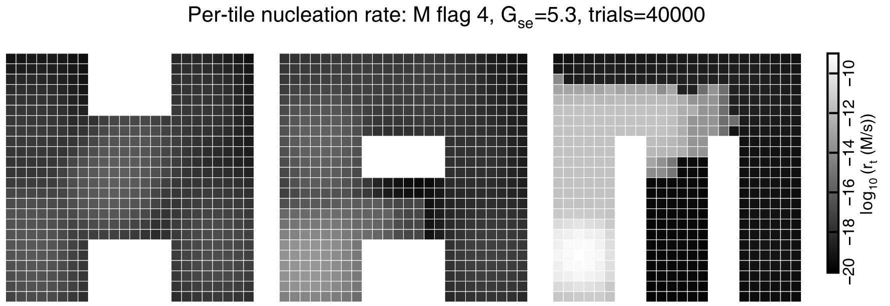
Per-tile nucleation rate: M flag 4, G_{se}=5.3, trials=40000
$\log_{10}(r_t$ (M/s))
−20 −18 −16 −14 −12 −10

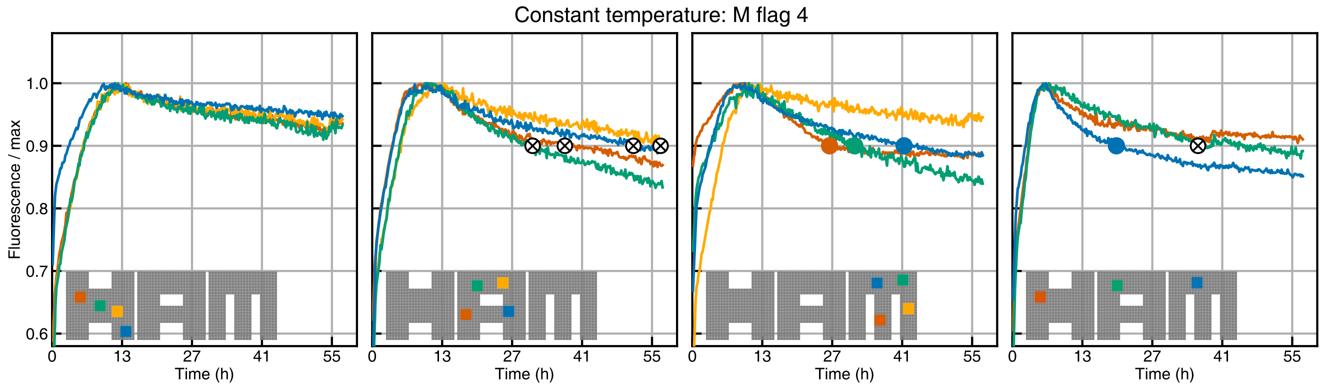
Constant temperature: M flag 4
Fluorescence / max
Time (h)
0 13 27 41 55

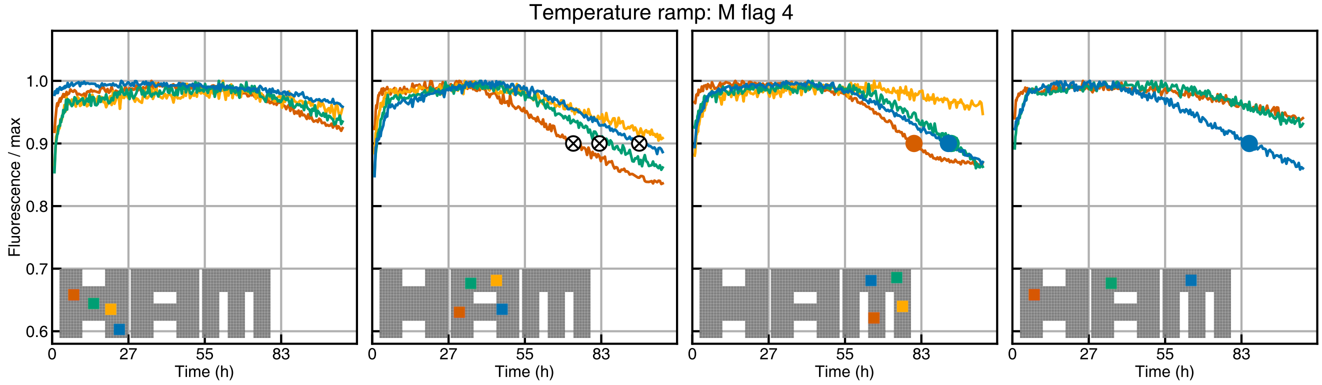
Temperature ramp: M flag 4
Fluorescence / max
Time (h)
0 27 55 83

5.3.29 M flag 5

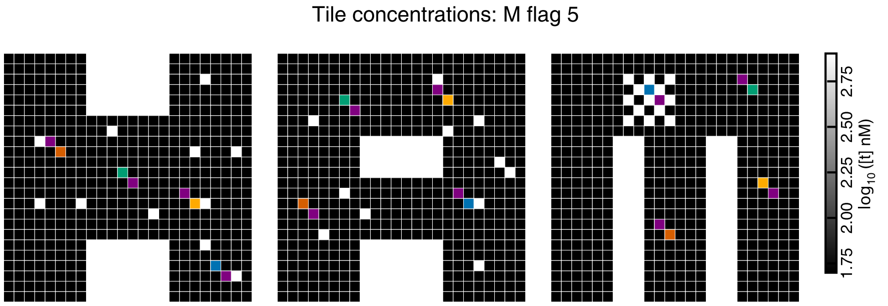
Tile concentrations: M flag 5
log$_{10}$([t] nM)
1.75 2.00 2.25 2.50 2.75

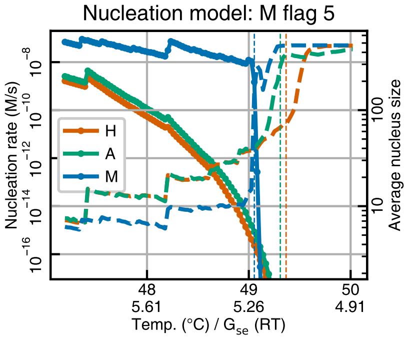
Nucleation model: M flag 5
Nucleation rate (M/s)
Average nucleus size
H
A
M
Temp. (°C) / G$_{se}$ (RT)
48 49 50
5.61 5.26 4.91

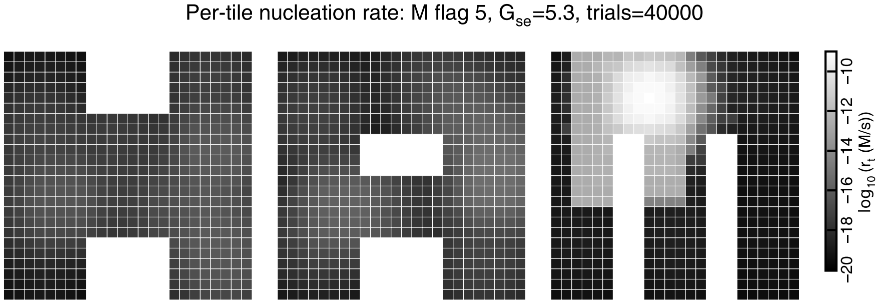
Per-tile nucleation rate: M flag 5, G$_{se}$=5.3, trials=40000
log$_{10}$ (r$_t$ (M/s))
−20 −18 −16 −14 −12 −10

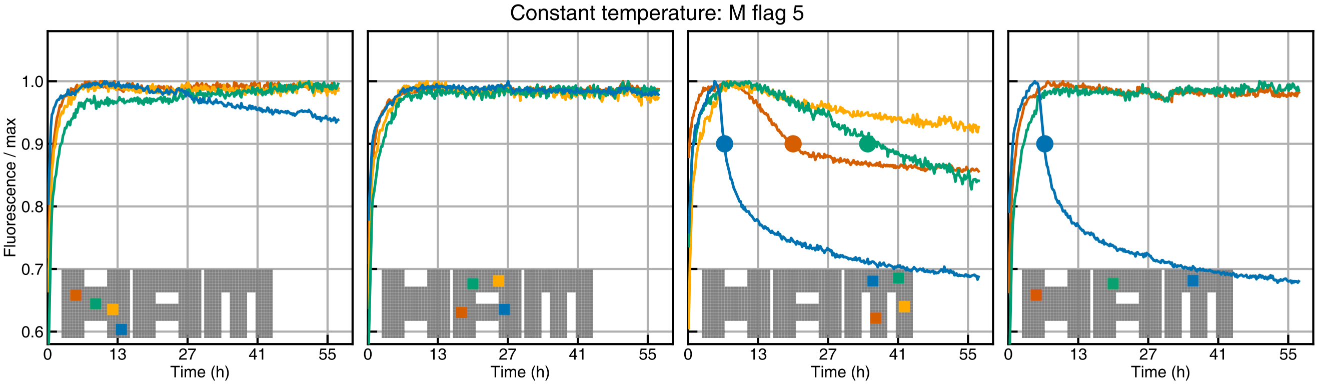
Constant temperature: M flag 5
Fluorescence / max
Time (h)
0 13 27 41 55

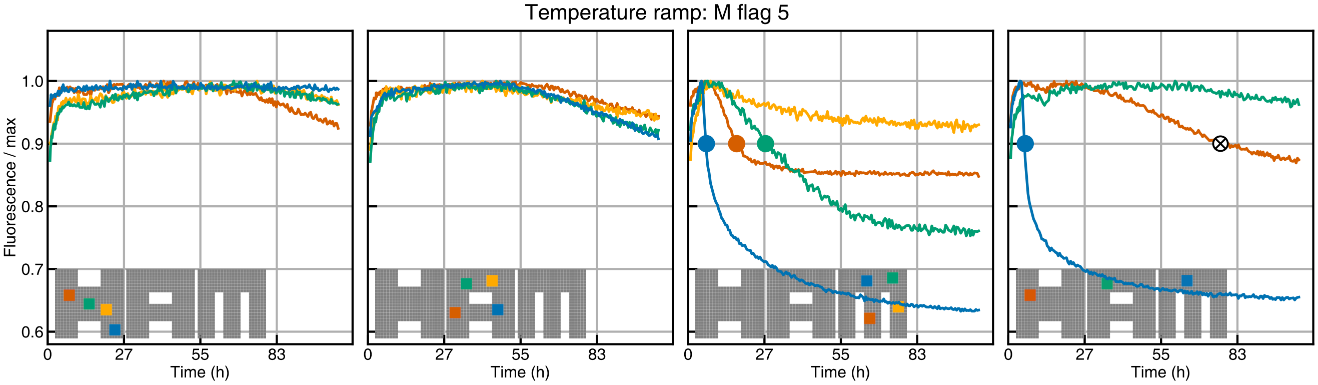
Temperature ramp: M flag 5
Fluorescence / max
Time (h)
0 27 55 83

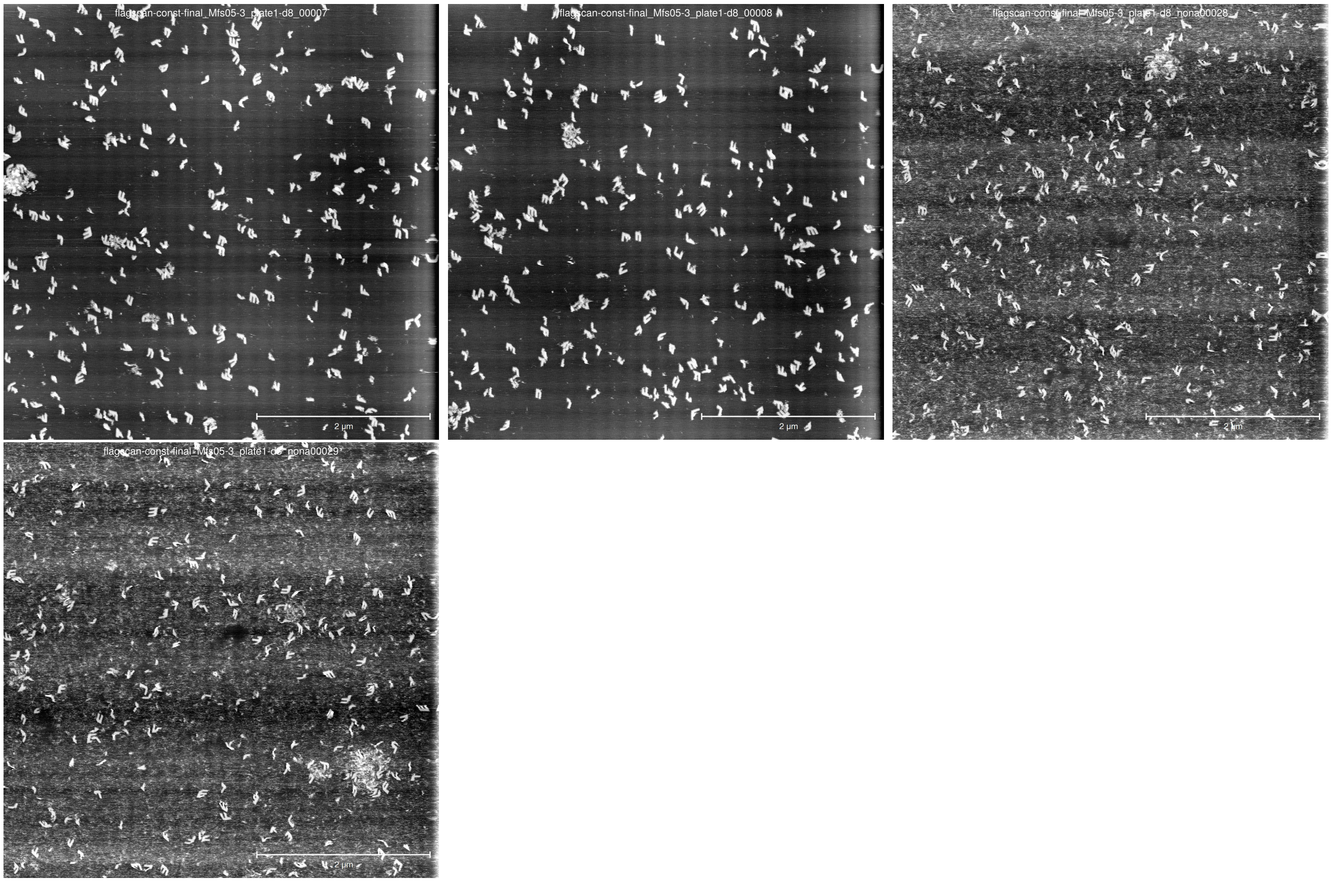
flagscan-const-final_Mfs05-3_plate1-d8_00008
2 µm
2 µm
2 µm
2 µm

5.3.30 M flag 6

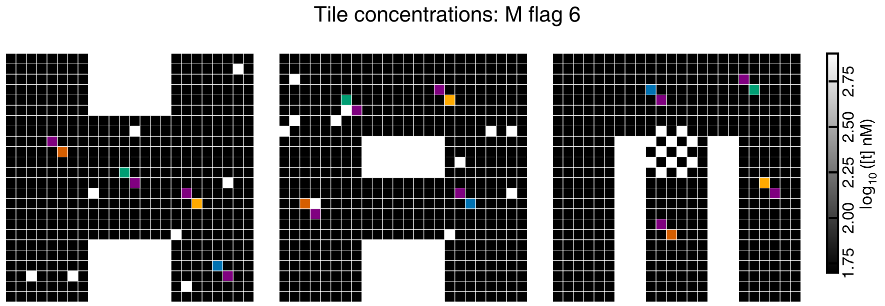
Tile concentrations: M flag 6
1.75
2.00
2.25
2.50
2.75
log10([t] nM)

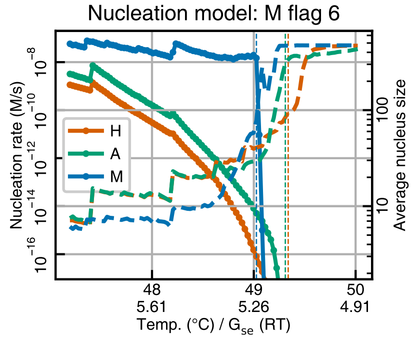
Nucleation model: M flag 6
H
A
M
Nucleation rate (M/s)
Average nucleus size
48
49
50
5.61
5.26
4.91
Temp. (°C) / Gse (RT)

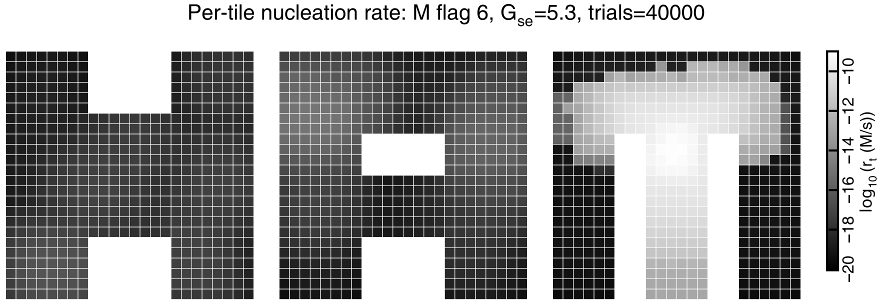
Per-tile nucleation rate: M flag 6, Gse=5.3, trials=40000
−20
−18
−16
−14
−12
−10
log10 (rt (M/s))

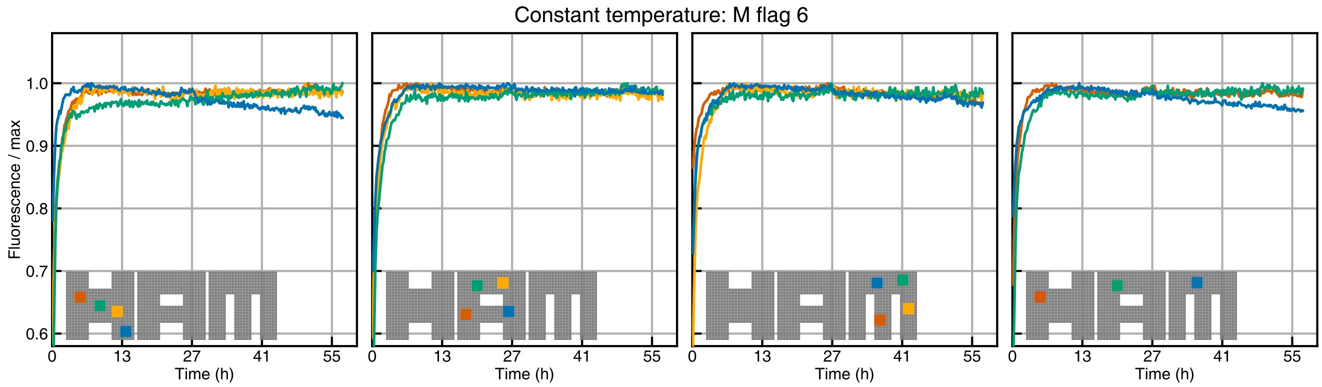
Constant temperature: M flag 6
Fluorescence / max
1.0
0.9
0.8
0.7
0.6
0
13
27
41
55
Time (h)

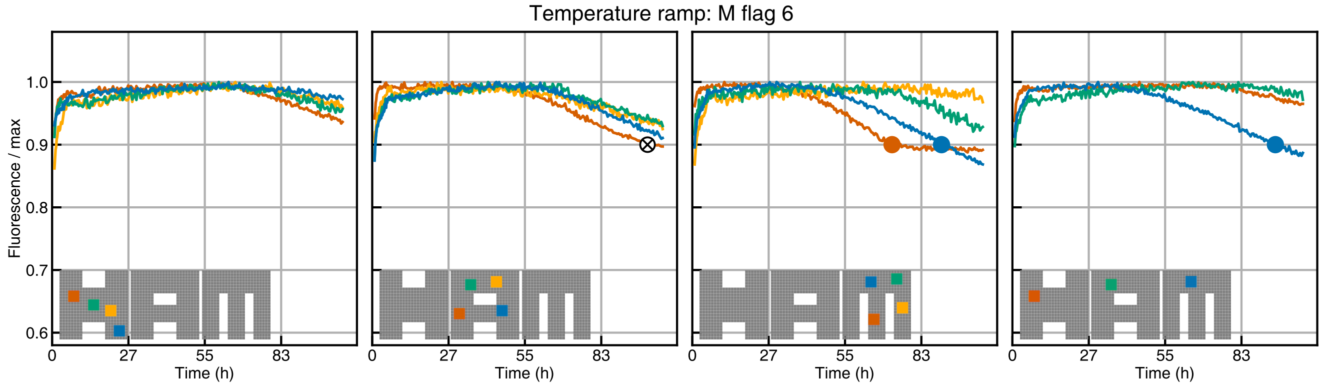
Temperature ramp: M flag 6
Fluorescence / max
1.0
0.9
0.8
0.7
0.6
0
27
55
83
Time (h)

5.3.31 M flag 7

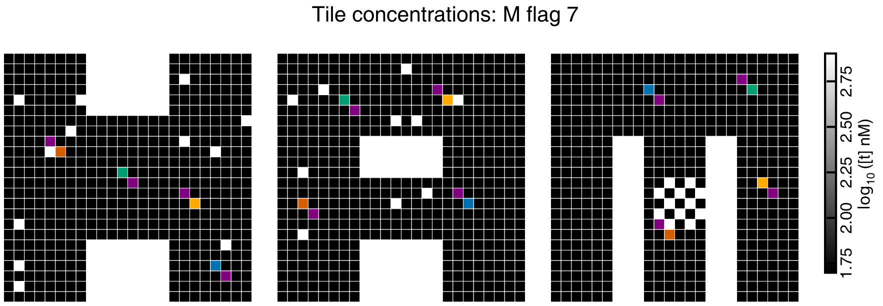
Tile concentrations: M flag 7
1.75
2.00
2.25
2.50
2.75
$\log_{10}([t]\ \mathrm{nM})$

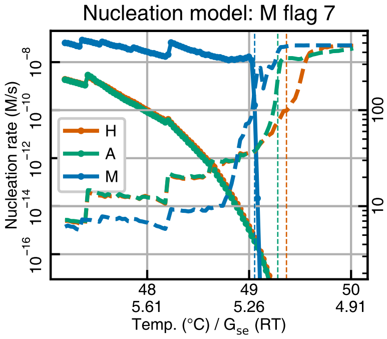
Nucleation model: M flag 7
Nucleation rate (M/s)
Average nucleus size
H
A
M
48
49
50
5.61
5.26
4.91
Temp. (°C) / G_{se} (RT)

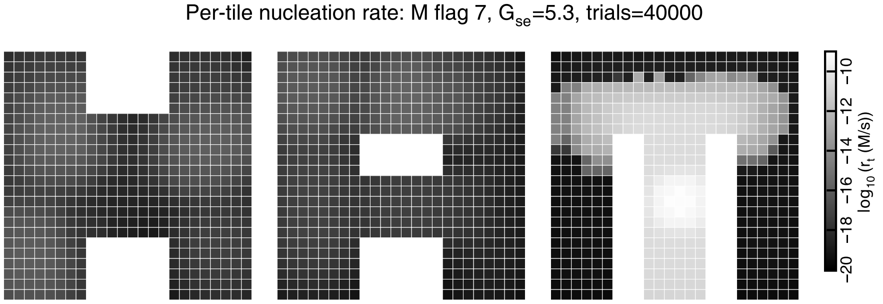
Per-tile nucleation rate: M flag 7, G_{se}=5.3, trials=40000
−20
−18
−16
−14
−12
−10
$\log_{10}(r_t\ (\mathrm{M/s}))$

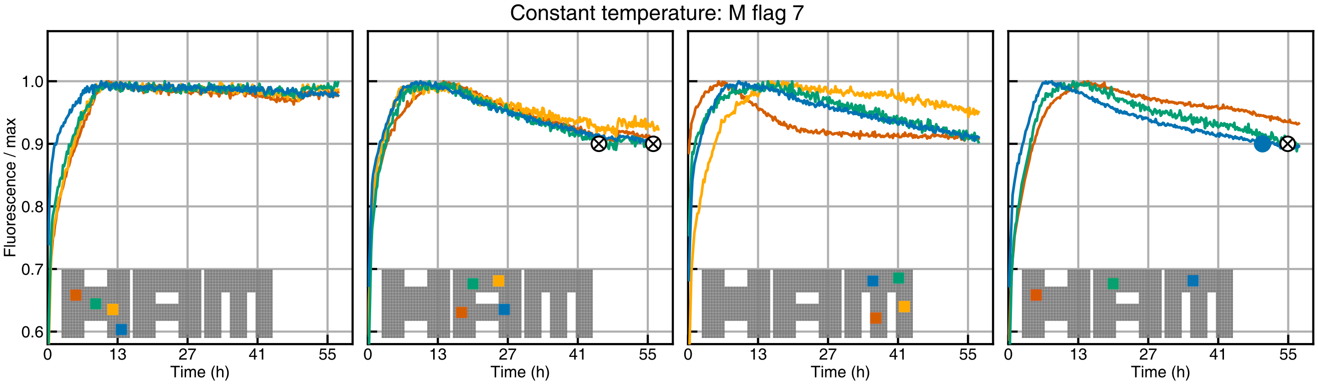
Constant temperature: M flag 7
Fluorescence / max
1.0
0.9
0.8
0.7
0.6
0
13
27
41
55
Time (h)

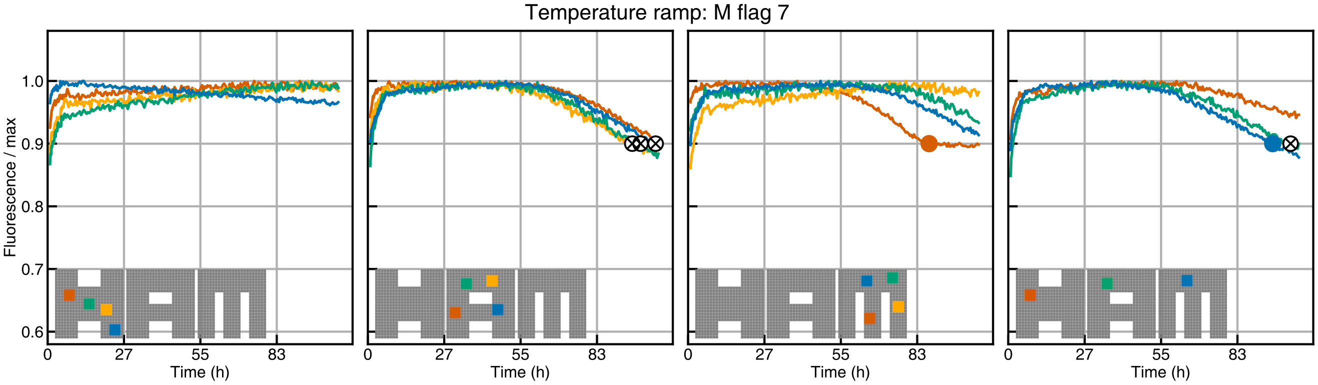
Temperature ramp: M flag 7
Fluorescence / max
1.0
0.9
0.8
0.7
0.6
0
27
55
83
Time (h)

5.3.32 M flag 8

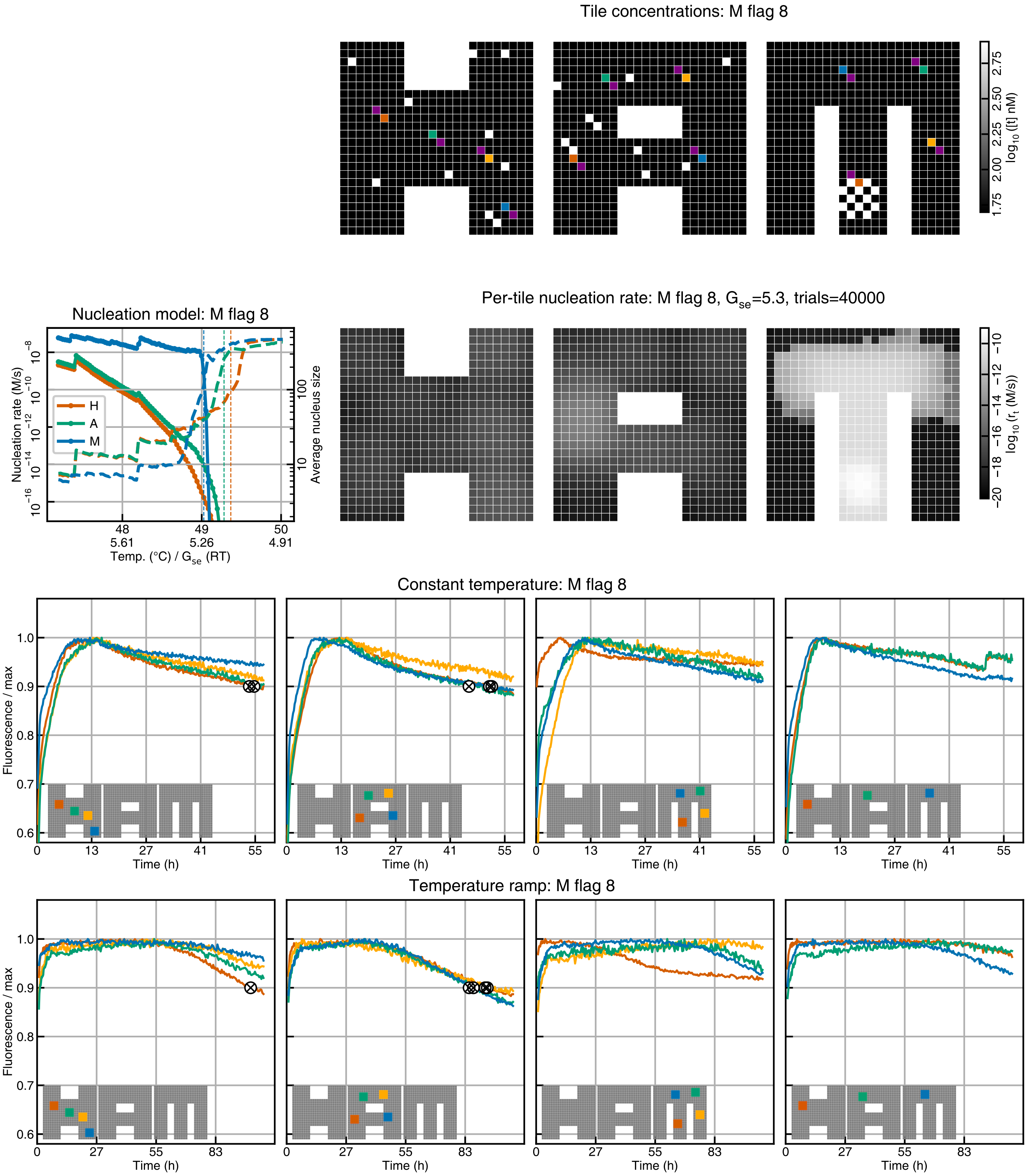
Tile concentrations: M flag 8
$\log_{10}([t]\ nM)$
Nucleation model: M flag 8
Nucleation rate (M/s)
Average nucleus size
H
A
M
Temp. (°C) / G_{se} (RT)
Per-tile nucleation rate: M flag 8, G_{se}=5.3, trials=40000
$\log_{10}(r_t\ (M/s))$
Constant temperature: M flag 8
Fluorescence / max
Time (h)
Temperature ramp: M flag 8
Fluorescence / max
Time (h)

5.3.33 M flag 9

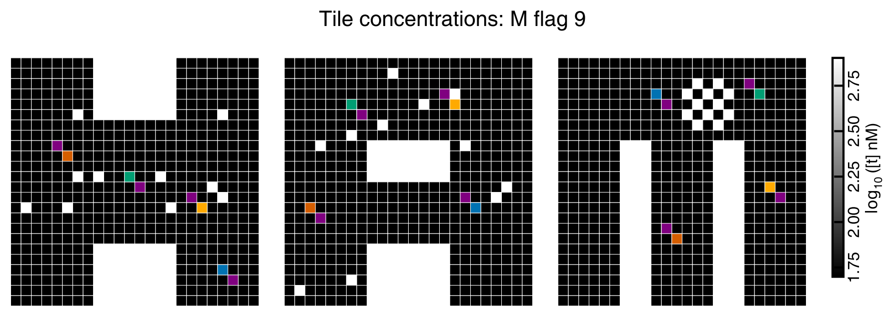
Tile concentrations: M flag 9
1.75 2.00 2.25 2.50 2.75
log10 ([t] nM)

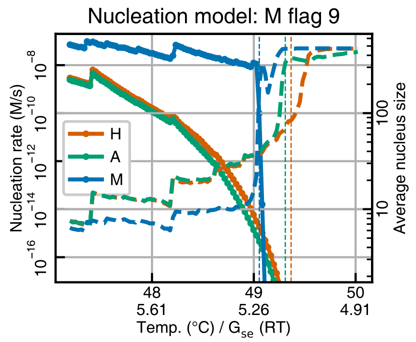
Nucleation model: M flag 9
Nucleation rate (M/s)
Average nucleus size
H
A
M
48 49 50
5.61 5.26 4.91
Temp. (°C) / Gse (RT)

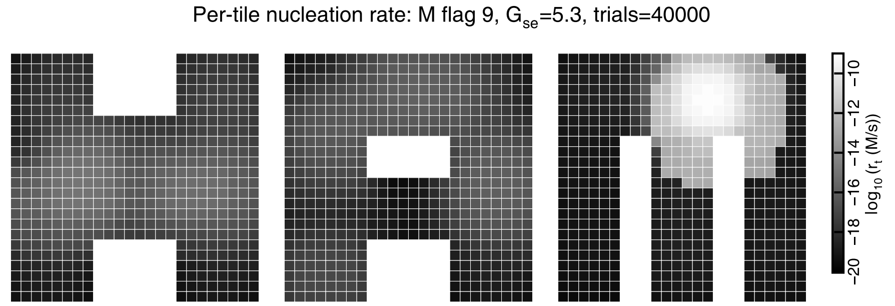
Per-tile nucleation rate: M flag 9, Gse=5.3, trials=40000
−20 −18 −16 −14 −12 −10
log10 (rt (M/s))

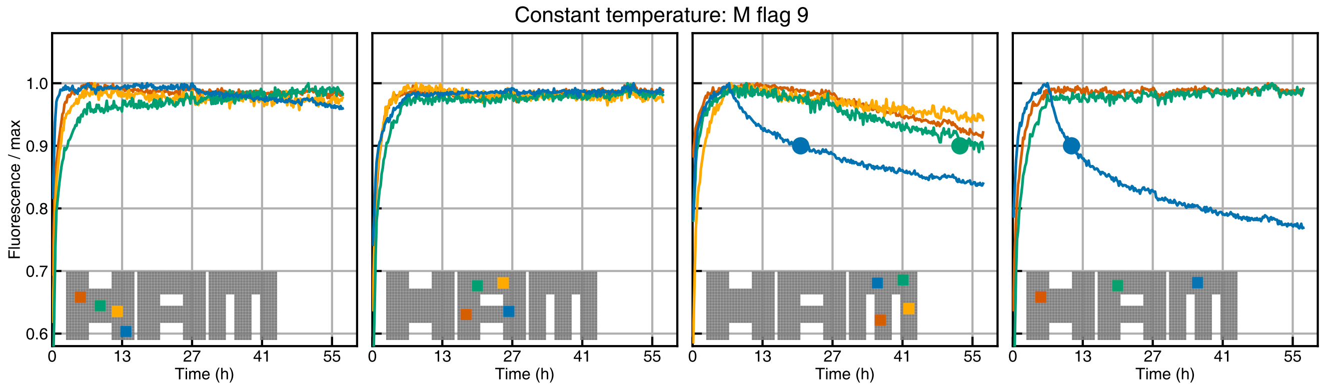
Constant temperature: M flag 9
Fluorescence / max
Time (h)
0 13 27 41 55

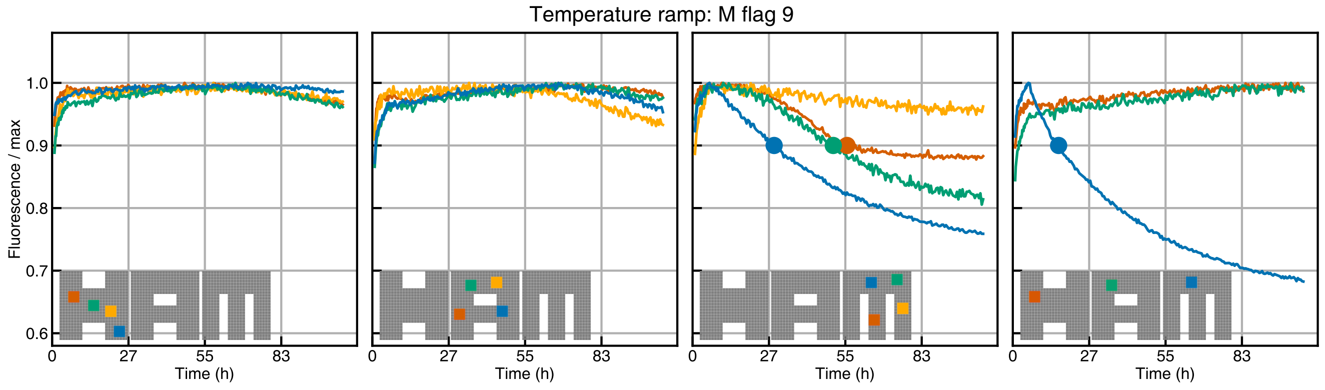
Temperature ramp: M flag 9
Fluorescence / max
Time (h)
0 27 55 83

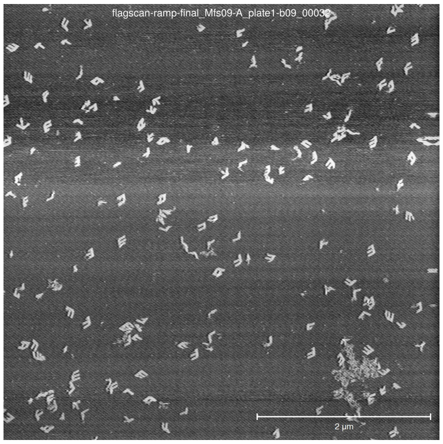
flagscan-ramp-final_Mfs09-A_plate1-b09_00032
2 µm

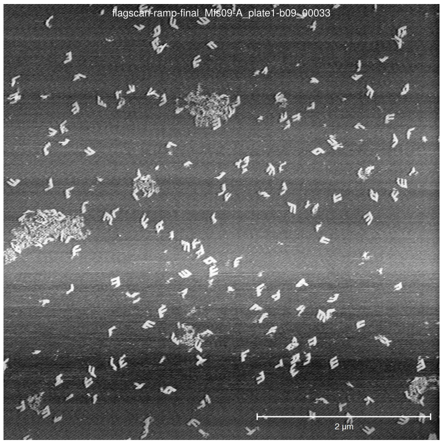
flagscan-ramp-final_Mfs09-A_plate1-b09_00033
2 µm

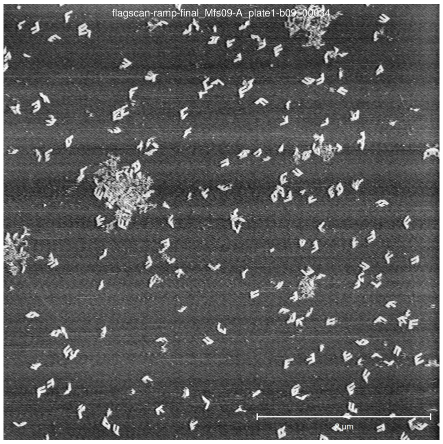
flagscan-ramp-final_Mfs09-A_plate1-b09_00034

5.3.34 M flag 10

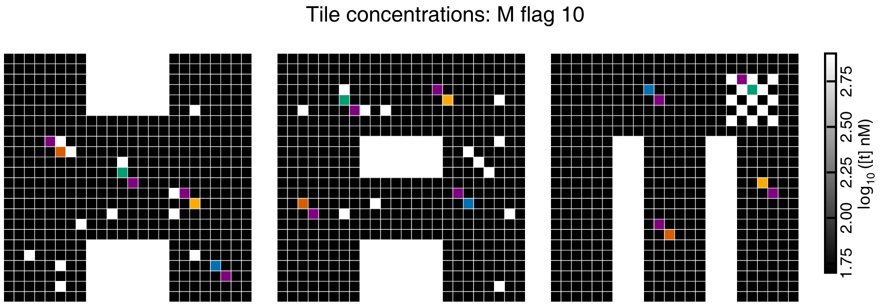

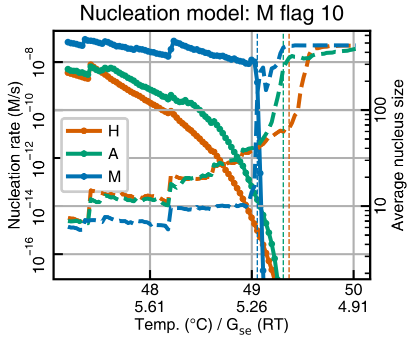

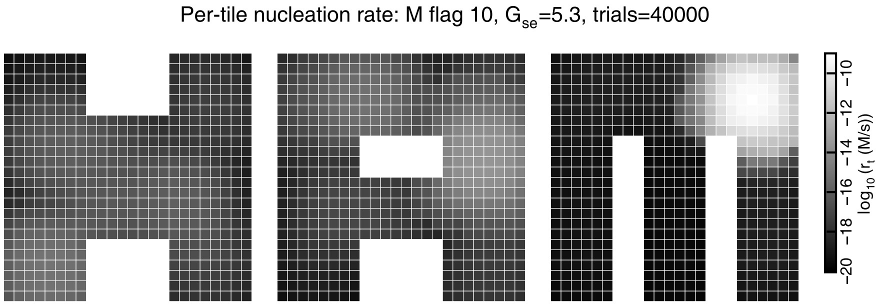

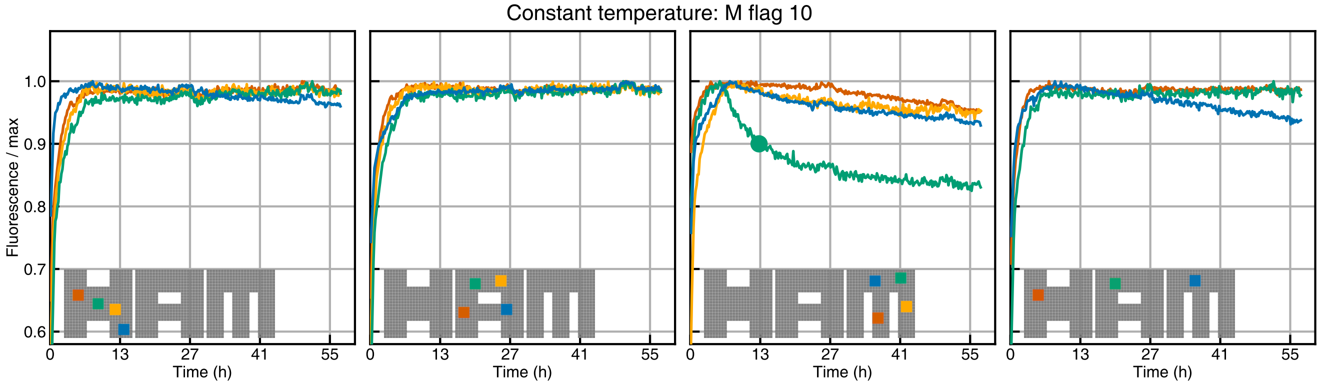

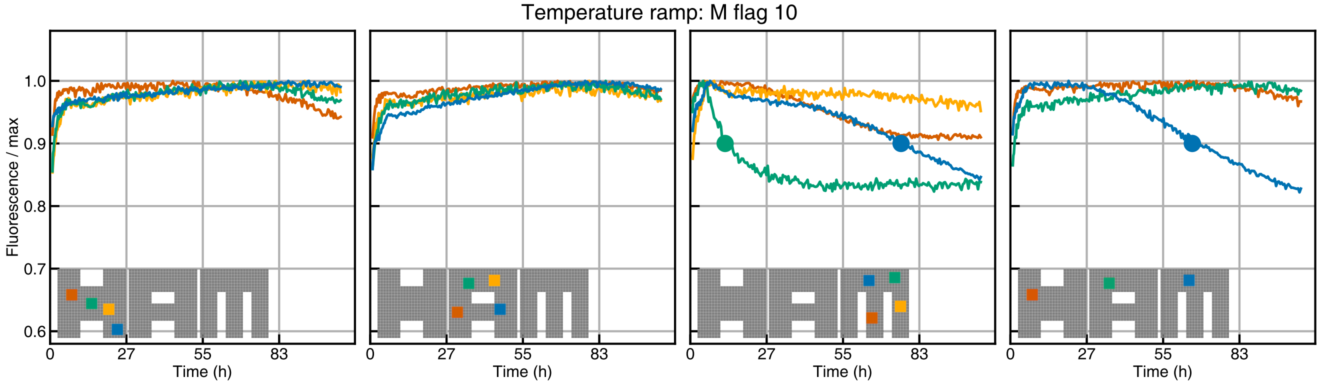

5.3.35 M flag 11

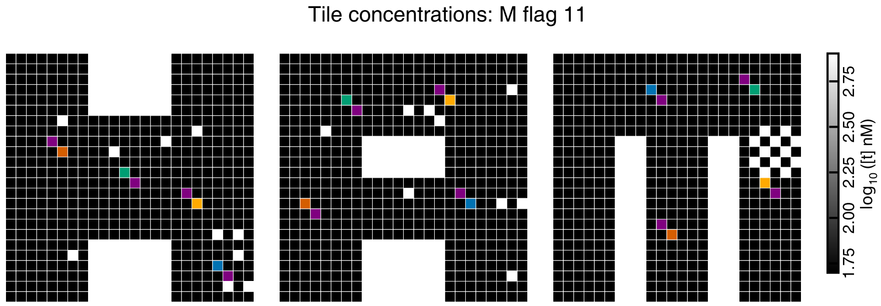

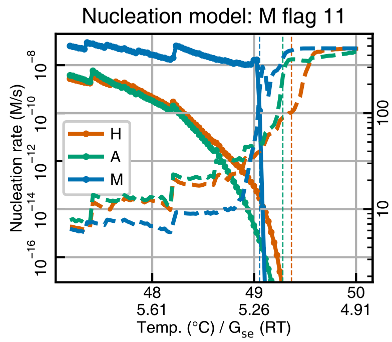

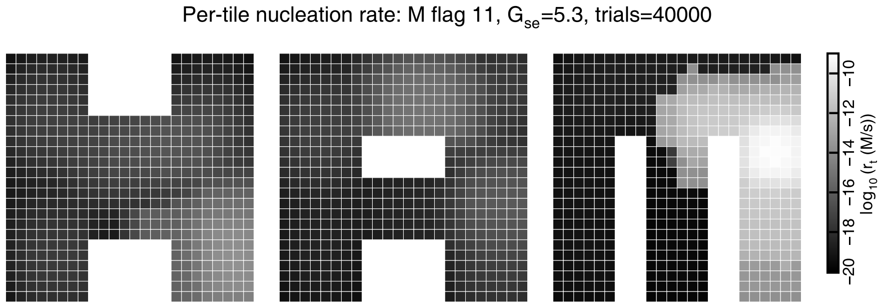

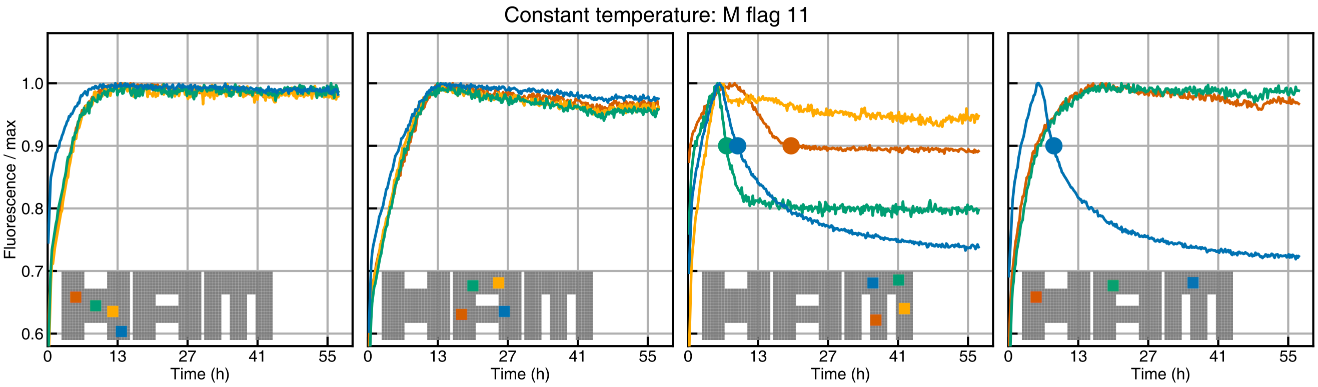

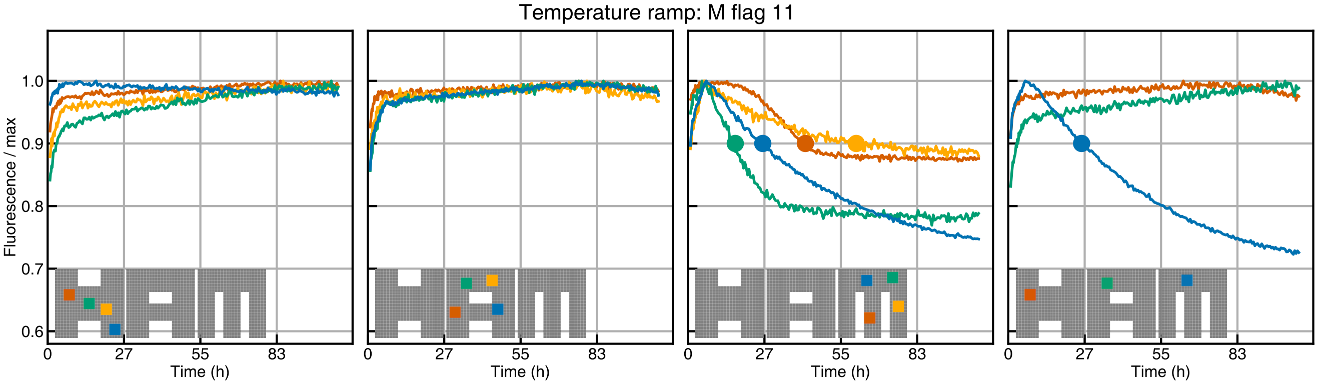

5.3.36 M flag 12

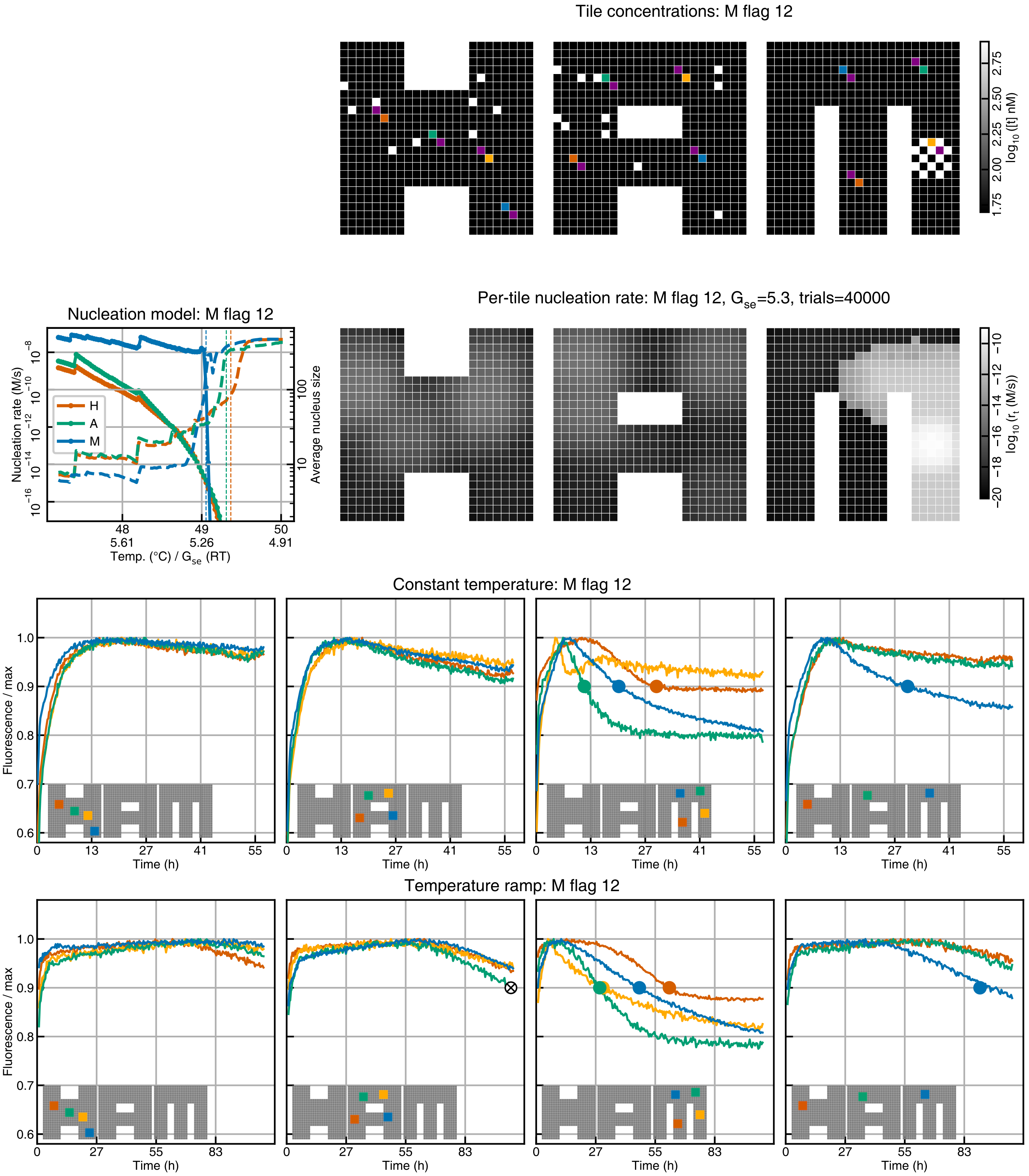

5.3.37 M flag 13

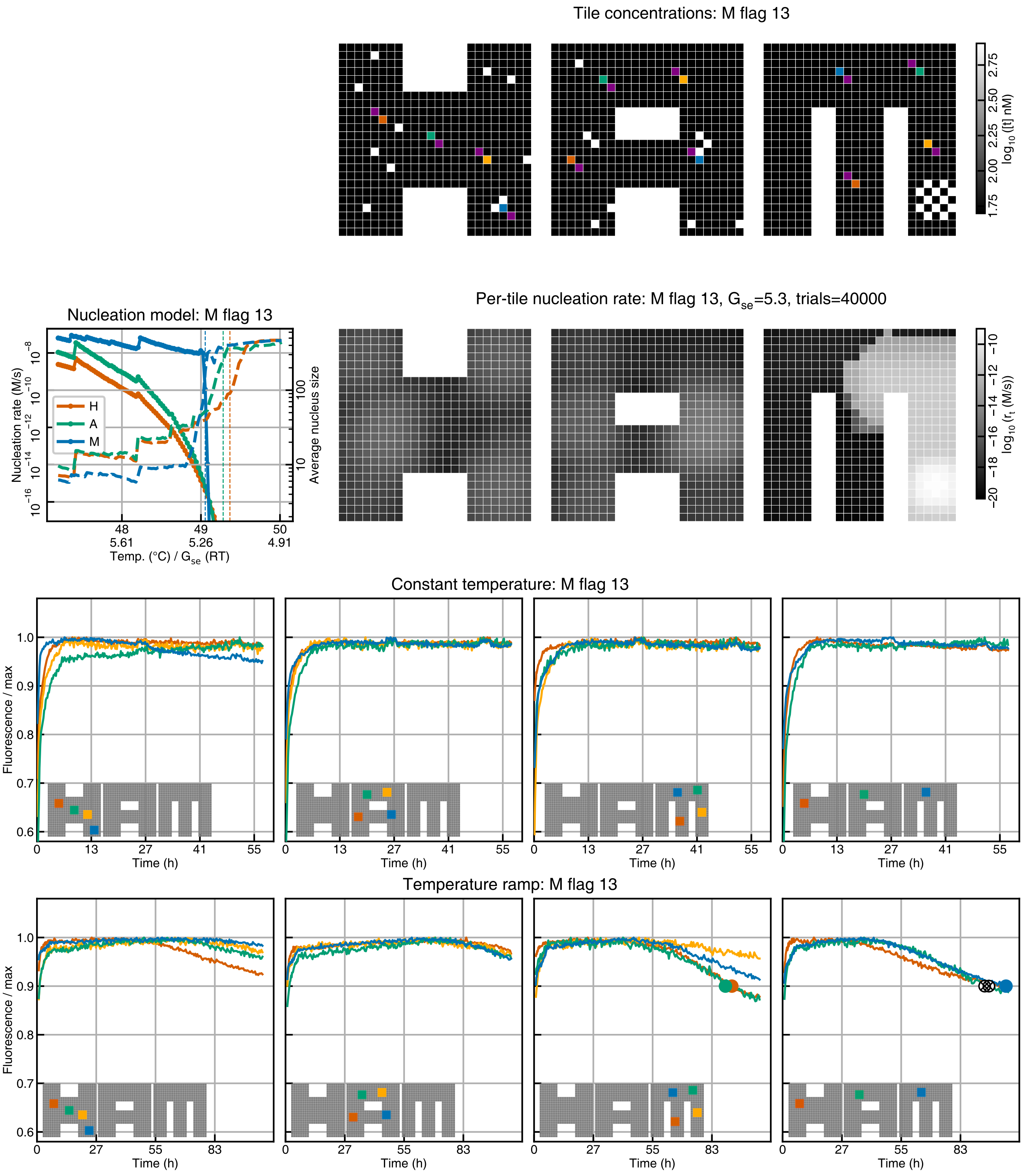

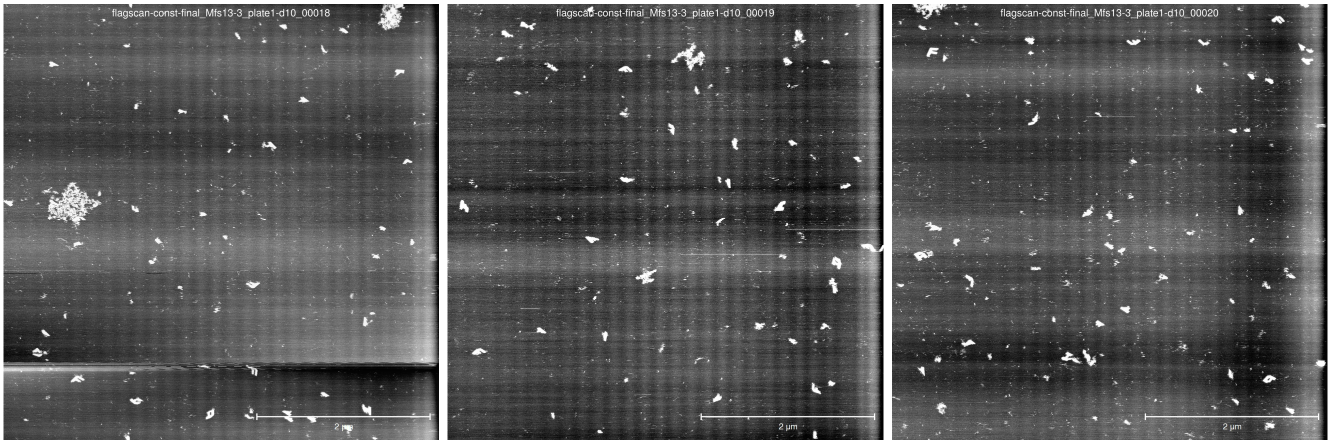
flagscan-const-final_Mfs13-3_plate1-d10_00018
2 µm
flagscan-const-final_Mfs13-3_plate1-d10_00019
2 µm
flagscan-const-final_Mfs13-3_plate1-d10_00020
2 µm

5.3.38 Uniform concentrations (50 nM)

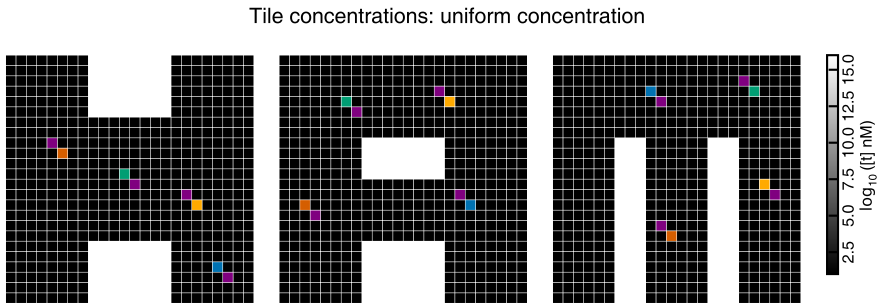

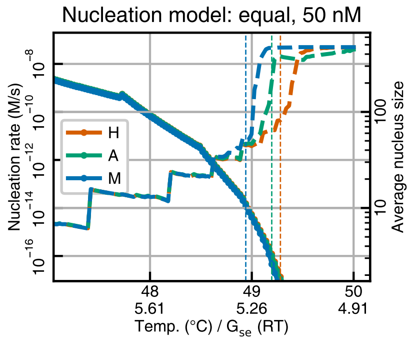

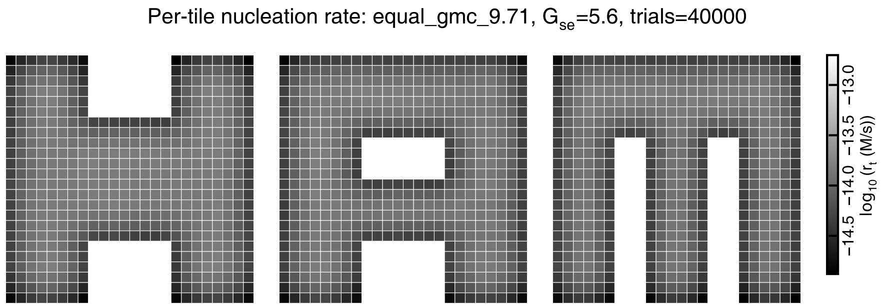

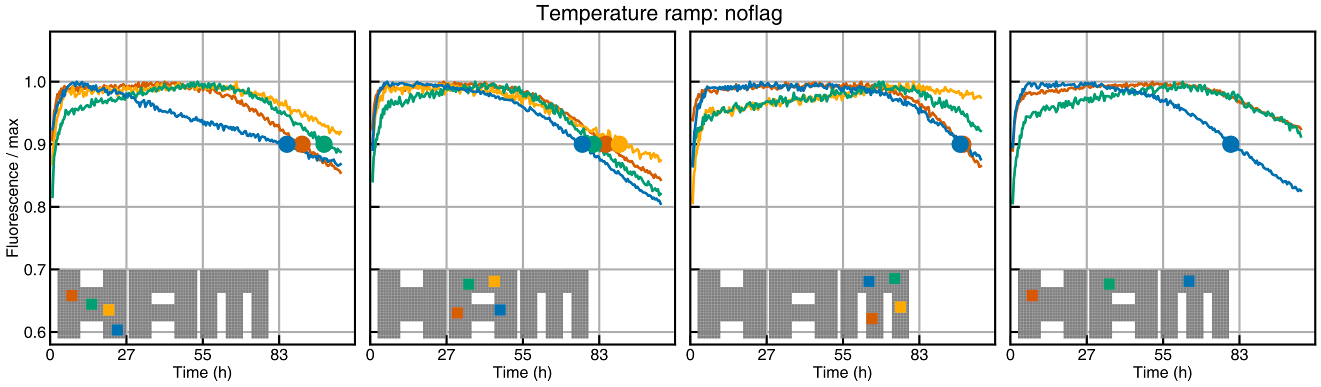

5.4 Plate-level fluorescence data used in Section 5.3

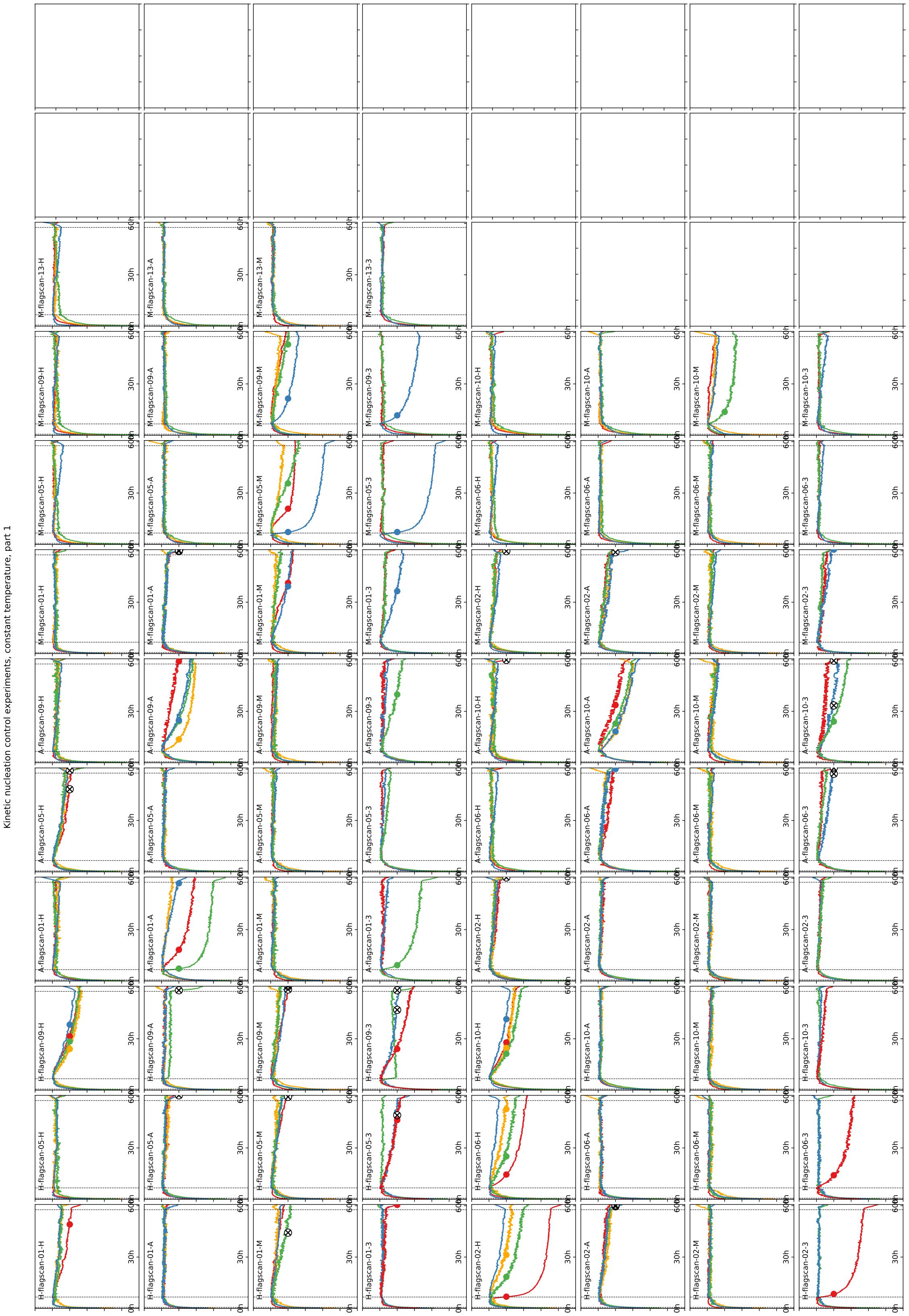

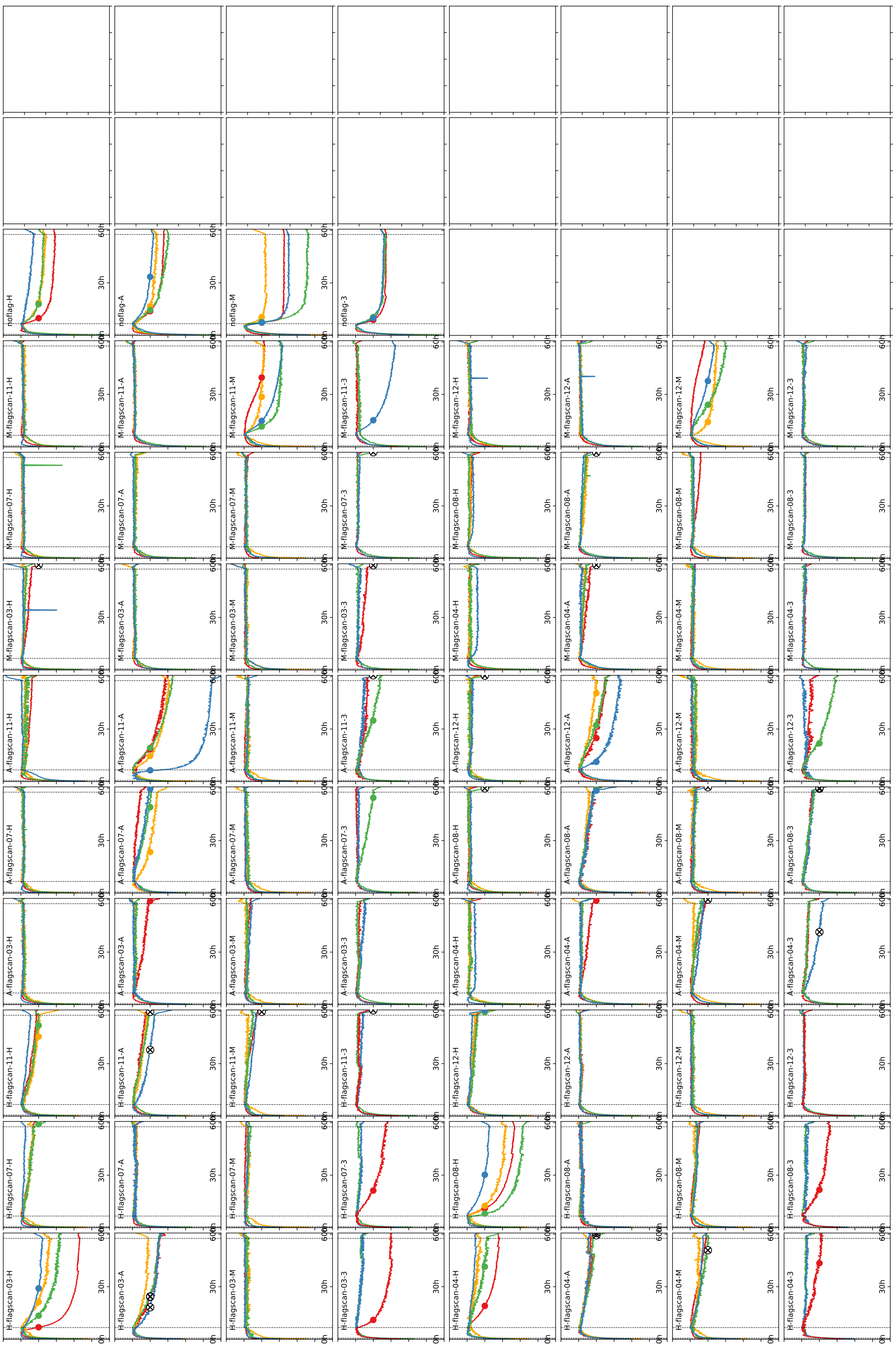

Kinetic nucleation control experiments, constant temperature, part 2

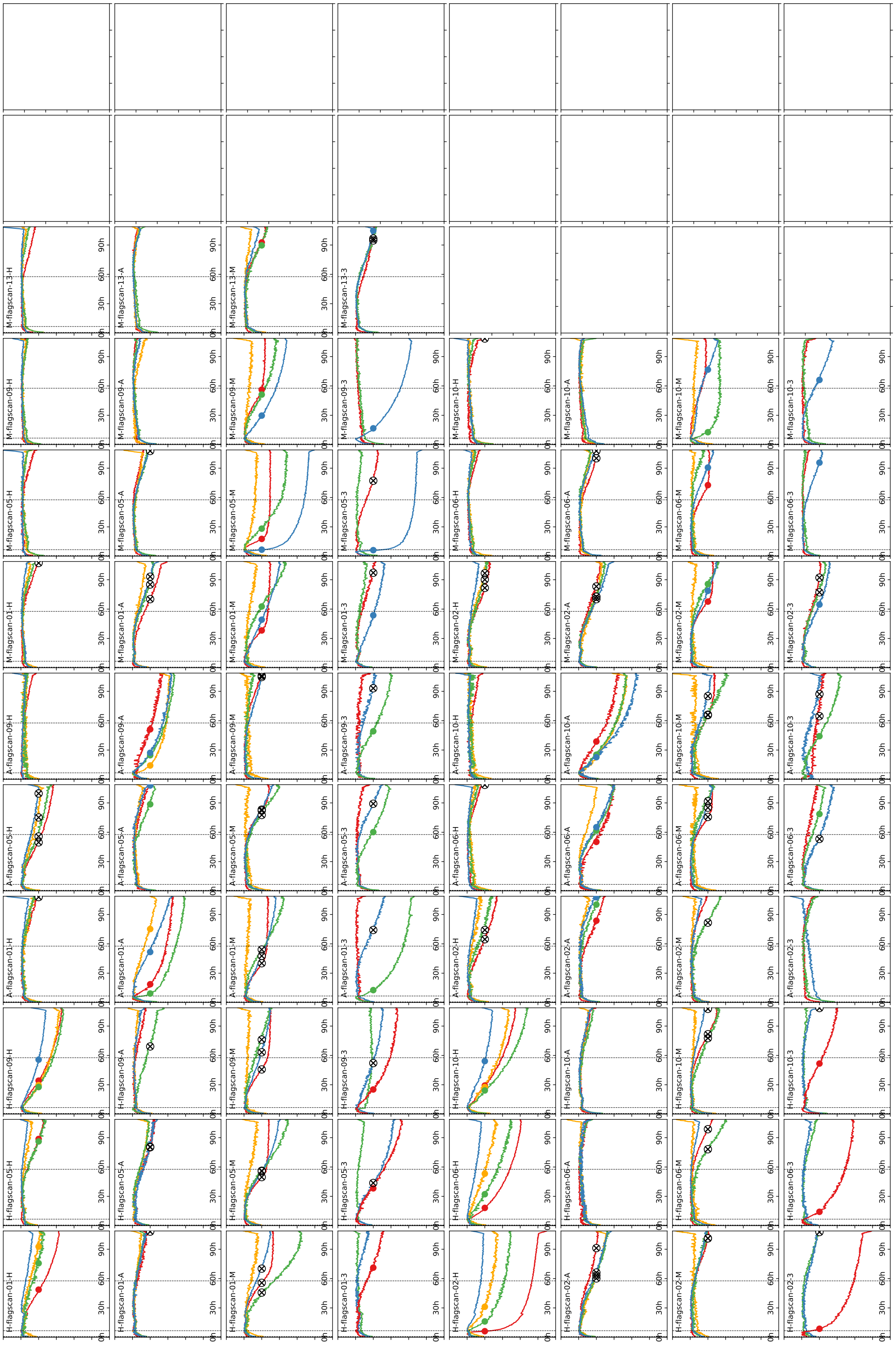

Kinetic nucleation control experiments, temperature ramp, part 1

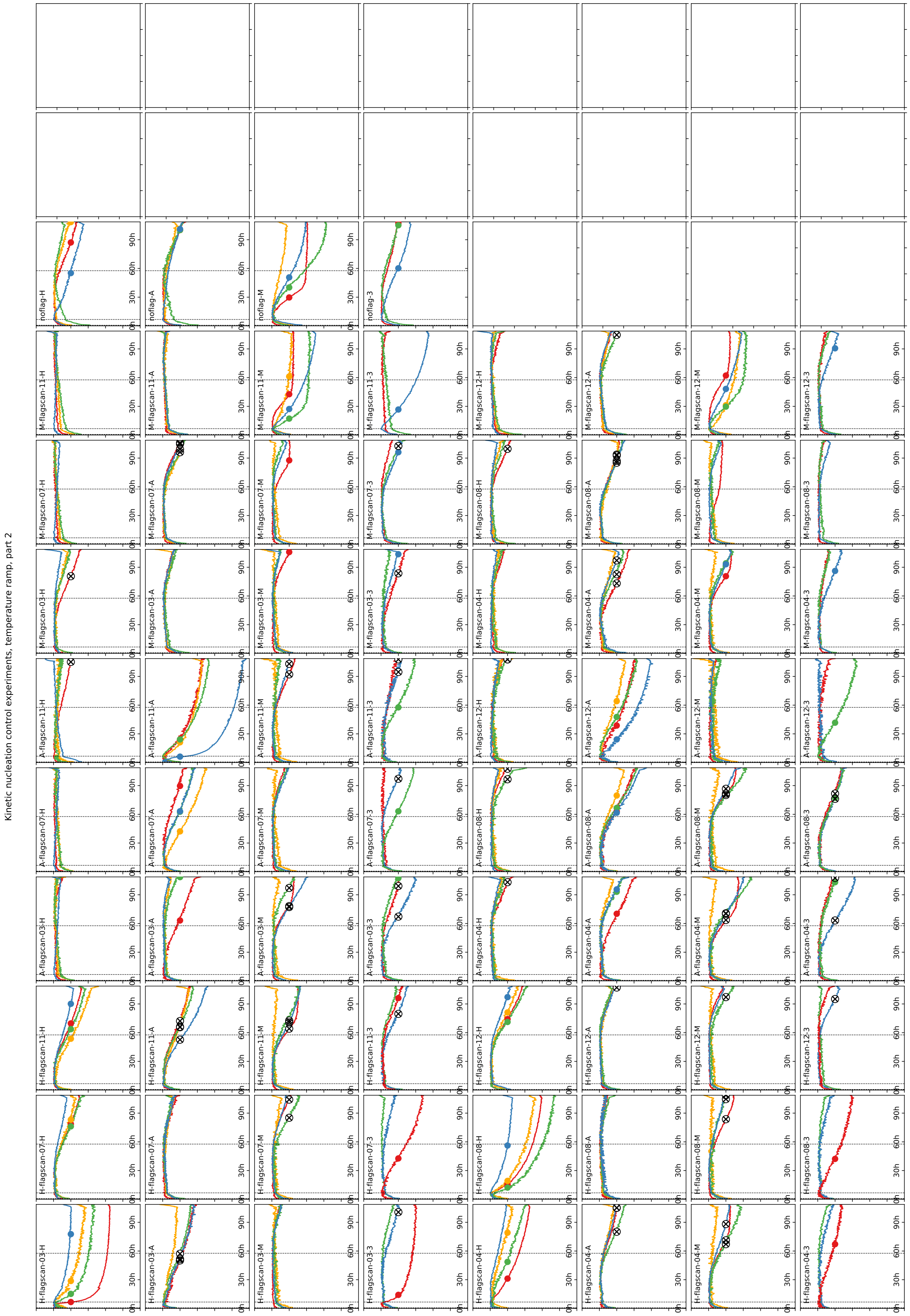
Kinetic nucleation control experiments, temperature ramp, part 2
noflag-H
noflag-A
noflag-M
noflag-3
M-flagscan-11-H
M-flagscan-11-A
M-flagscan-11-M
M-flagscan-11-3
M-flagscan-12-H
M-flagscan-12-A
M-flagscan-12-M
M-flagscan-12-3
M-flagscan-07-H
M-flagscan-07-A
M-flagscan-07-M
M-flagscan-07-3
M-flagscan-08-H
M-flagscan-08-A
M-flagscan-08-M
M-flagscan-08-3
M-flagscan-03-H
M-flagscan-03-A
M-flagscan-03-M
M-flagscan-03-3
M-flagscan-04-H
M-flagscan-04-A
M-flagscan-04-M
M-flagscan-04-3
A-flagscan-11-H
A-flagscan-11-A
A-flagscan-11-M
A-flagscan-11-3
A-flagscan-12-H
A-flagscan-12-A
A-flagscan-12-M
A-flagscan-12-3
A-flagscan-07-H
A-flagscan-07-A
A-flagscan-07-M
A-flagscan-07-3
A-flagscan-08-H
A-flagscan-08-A
A-flagscan-08-M
A-flagscan-08-3
A-flagscan-03-H
A-flagscan-03-A
A-flagscan-03-M
A-flagscan-03-3
A-flagscan-04-H
A-flagscan-04-A
A-flagscan-04-M
A-flagscan-04-3
H-flagscan-11-H
H-flagscan-11-A
H-flagscan-11-M
H-flagscan-11-3
H-flagscan-12-H
H-flagscan-12-A
H-flagscan-12-M
H-flagscan-12-3
H-flagscan-07-H
H-flagscan-07-A
H-flagscan-07-M
H-flagscan-07-3
H-flagscan-08-H
H-flagscan-08-A
H-flagscan-08-M
H-flagscan-08-3
H-flagscan-03-H
H-flagscan-03-A
H-flagscan-03-M
H-flagscan-03-3
H-flagscan-04-H
H-flagscan-04-A
H-flagscan-04-M
H-flagscan-04-3
0h
30h
60h
90h

Section 6

Pattern recognition

6.1 Protocol

1. 71 °C for 4 minutes, no fluorescence measurement.
2. 71 °C to 55 °C, in steps of -2.0 °C every 4 minutes, taking a measurement at the end of each hold (36 minutes in total).
3. 53 °C to 48.2 °C, in steps of -0.2 °C every 12 minutes, taking a measurement at the end of each hold (5 hours in total).
4. 48 °C to 44.9 °C, in steps of -0.1 °C every 5 hours, taking a measurement every 30 minutes (160 hours in total).
5. 45 °C to 39.5 °C, in steps of -0.5 °C every 13 minutes, taking a measurement at the end of each hold (2 hours 36 minutes in total).

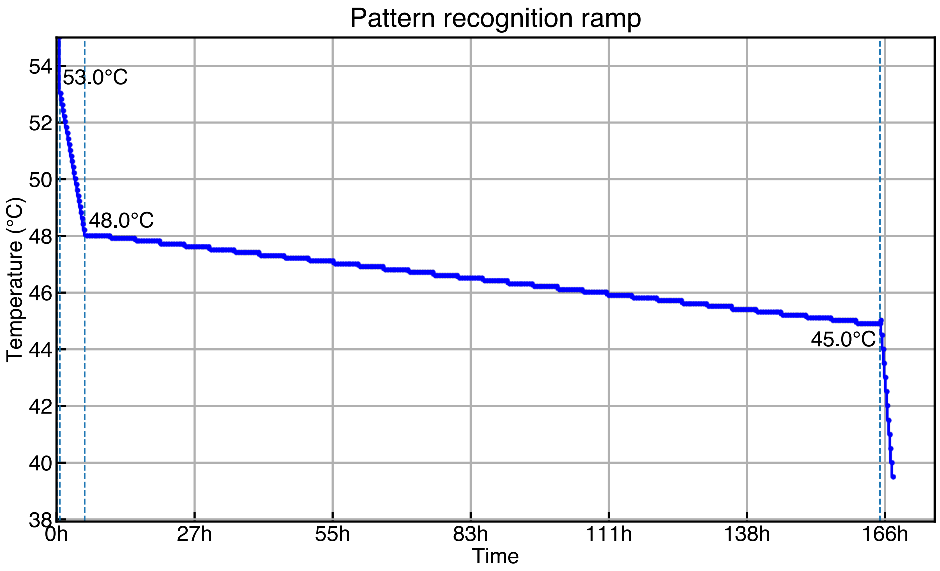

6.2 Nucleation model summaries

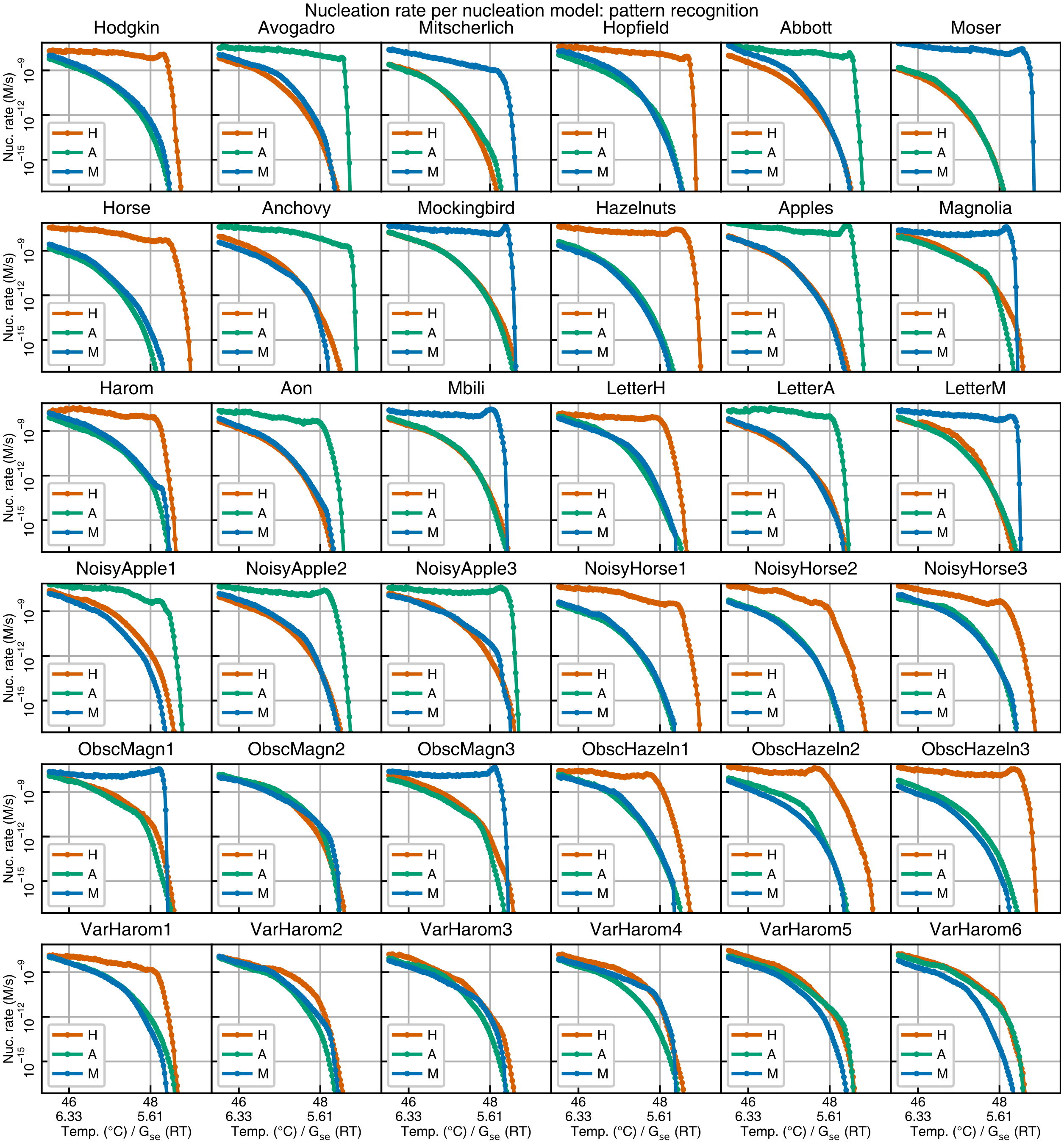

6.3 AFM shapes and counts summary

Counts of shapes were done manually by each of the four co-authors. Images were blinded by having a script assign random numbers to each image, and using PNG image files (with no AFM metadata) named using these numbers. CGE had previously seen all images associated with their corresponding samples while performing the AFM imaging.

Samples in the tables below are referred to as 'H', 'A', or 'M' for samples with four fluorophores on one shape, and '3' for samples where each shape had one fluorophore (resulting in three total fluorophores). Each imaged sample (with a different fluorophore configuration) of each pattern had three 5 µm × 5 µm images, with exception of the H, A, and M samples for Magnolia, which had two. Owing to the significantly larger numbers of shapes seen in the SHAM mix, only 1/4 of each image was used for counting. Numbers in the tables below are averaged across the counts from each co-author, and normalized to the number of shapes per μm^2.

To examine the potential effect of fluorophore and quencher tile modifications on shapes seen by AFM, several patterns had multiple samples imaged, while the '3' sample was imaged for every pattern. In cases where the concentration pattern did not result in extremely selective nucleation, so that non-target shapes could be counted and smaller effects on nucleation rate could be discerned, shape counts show a bias toward nucleating shapes with more fluorophore-quencher pairs. This effect is particularly evident for the equal-concentration SHAM mix: with one fluorophore-quencher pair on each shape (the '3' sample), counts of each shape are roughly equal, but when four fluorophore-quencher pairs are instead located on a single shape, either 'H' or 'A', that shape rises to over 1/2 of the observed shapes. Both the purification of flourophore- and quencher-modified strands resulting in a higher effective concentration compared to unpurified, unmodified strands, and fluorophore-quencher interactions, may play a role in the effect.

Shapes were classified into categories 1 and 2, where 1 were "almost complete" shapes, possibly missing small portions, while 2 were "clearly recognizable" shapes, which were distinctly not complete, but still clearly distinguishable. Detailed criteria distinguishing these categories were not developed: instead, each co-author interpreted the categories independently.

6.3.1 Counts averaged across co-authors and images, normalized to shapes per 25 μm^2:

Pattern	Sample	H-1	H-2	A-1	A-2	M-1	M-2	total #	% target	%H	%A	%M
Hodgkin	3	4.17	41.00	0.00	0.00	0.00	0.92	46.08	98	98	0	2
Avogadro	3	0.00	0.00	8.25	21.25	0.00	0.75	30.25	98	0	98	2
Mitscherlich	3	0.00	0.17	0.00	0.08	14.00	36.92	51.17	100	0	0	100
Hopfield	3	13.92	30.08	0.00	0.00	0.00	0.33	44.33	99	99	0	1
Abbott	3	0.08	0.42	18.17	20.33	0.00	0.08	39.08	99	1	99	0
Moser	3	0.00	0.00	0.00	0.00	4.33	32.25	36.58	100	0	0	100
Horse	H	18.12	37.62	0.00	0.00	0.00	0.00	55.75	100	100	0	0
	A	22.08	55.50	0.00	0.00	0.00	0.00	77.58	100	100	0	0
	M	5.67	33.50	0.00	0.00	0.00	0.00	39.17	100	100	0	0
	3	7.08	31.08	0.08	0.08	0.00	0.25	38.58	99	99	0	1
Anchovy	3	0.00	0.33	20.33	23.17	0.00	0.08	43.92	99	1	99	0
Mockingbird	H	0.83	4.75	0.00	0.00	1.25	4.92	11.75	52	48	0	52
	A	0.08	3.17	6.75	9.42	3.50	6.42	29.33	34	11	55	34
	M	0.00	2.25	0.00	1.17	1.58	10.58	15.58	78	14	7	78
	3	1.00	3.58	0.75	2.58	1.50	8.25	17.67	55	26	19	55
Hazelnuts	3	7.67	41.83	0.42	2.08	0.00	0.17	52.17	95	95	5	0
Apples	3	0.00	0.00	21.92	23.67	0.00	0.00	45.58	100	0	100	0
Magnolia	H	0.00	5.75	2.50	5.50	14.75	29.88	58.38	76	10	14	76
	A	0.00	3.00	9.25	10.38	23.25	45.50	91.38	75	3	21	75
	M	0.00	2.00	2.50	2.25	17.25	31.38	55.38	88	4	9	88
	3	0.00	0.25	0.33	0.42	3.25	17.25	21.50	95	1	3	95
Harom	3	5.33	47.83	0.08	1.25	0.08	1.17	55.75	95	95	2	2
Aon	H	0.00	1.67	3.17	8.42	1.25	4.17	18.67	62	9	62	29
	A	0.00	0.75	26.58	17.92	4.00	13.67	62.92	71	1	71	28
	M	0.25	2.75	16.75	23.12	4.00	20.12	67.00	60	4	60	36
	3	0.00	0.08	8.58	20.92	0.67	8.33	38.58	76	0	76	23
Mbili	H	0.42	8.75	1.83	4.17	22.75	29.83	67.75	78	14	9	78
	A	0.00	1.08	7.58	8.17	16.75	25.67	59.25	72	2	27	72
	M	0.00	0.83	0.42	0.75	10.67	17.58	30.25	93	3	4	93
	3	0.00	0.25	0.00	0.00	4.83	16.83	21.92	99	1	0	99
H	H	23.67	76.17	0.33	0.75	0.08	1.42	102.42	97	97	1	1
	A	4.33	36.92	2.00	3.17	0.00	1.17	47.58	87	87	11	2
	M	6.00	41.25	0.08	0.17	1.33	6.00	54.83	86	86	0	13
	3	0.67	26.17	0.00	0.25	0.17	1.67	28.92	93	93	1	6
A	3	0.00	1.42	19.17	17.83	0.33	4.00	42.75	87	3	87	10
M	3	0.00	0.33	1.50	4.58	14.50	19.67	40.58	84	1	15	84
NoisyApple1	3	0.08	0.08	0.08	0.42	0.08	0.08	0.83	60	20	60	20
NoisyApple2	3	0.00	0.00	0.00	0.67	0.00	0.42	1.08	62	0	62	38
NoisyApple3	3	0.00	0.00	2.67	10.42	0.00	0.17	13.25	99	0	99	1
NoisyHorse1	3	7.50	30.50	0.08	0.00	0.00	0.00	38.08	100	100	0	0
NoisyHorse2	3	0.00	0.50	0.00	0.00	0.00	0.00	0.50	100	100	0	0
NoisyHorse3	3	0.00	3.75	0.00	0.00	0.00	0.00	3.75	100	100	0	0
ObscMagn1	3	0.00	0.00	0.00	0.00	0.00	0.25	0.25	100	0	0	100
ObscMagn2	3	0.00	0.00	0.00	0.00	0.00	0.00	0.00	0	0	0	0
ObscMagn3	3	0.00	0.00	0.58	1.67	2.33	15.42	20.00	89	0	11	89
ObscHazeln1	3	0.00	1.00	0.00	0.00	0.00	0.08	1.08	92	92	0	8
ObscHazeln2	3	0.42	9.33	0.00	0.33	0.42	0.92	11.42	85	85	3	12
ObscHazeln3	3	0.00	0.58	0.00	0.00	0.00	0.08	0.67	88	88	0	12

Continued on next page

Pattern	Sample	H-1	H-2	A-1	A-2	M-1	M-2	total #	% target	%H	%A	%M
VarHarom1	3	0.00	0.00	0.00	0.00	0.00	0.00	0.00	0	0	0	0
VarHarom2	3	0.00	0.58	0.00	0.00	0.00	0.00	0.58	100	100	0	0
VarHarom3	3	0.00	0.00	0.00	0.00	0.00	0.00	0.00	0	0	0	0
VarHarom4	3	0.08	2.50	0.00	0.00	0.00	0.08	2.67	97	97	0	3
VarHarom5	3	0.00	1.08	0.08	0.58	0.00	0.17	1.92	57	57	35	9
VarHarom6	3	0.00	0.00	0.00	0.00	0.00	0.00	0.00	0	0	0	0
SHAM	H	53.75	164.08	48.25	47.00	44.92	50.00	408.00	100	53	23	23
	A	7.83	67.17	123.14	53.75	43.64	38.44	333.97	100	22	53	25
	3	18.58	92.42	48.08	47.08	64.75	35.33	306.25	100	36	31	33

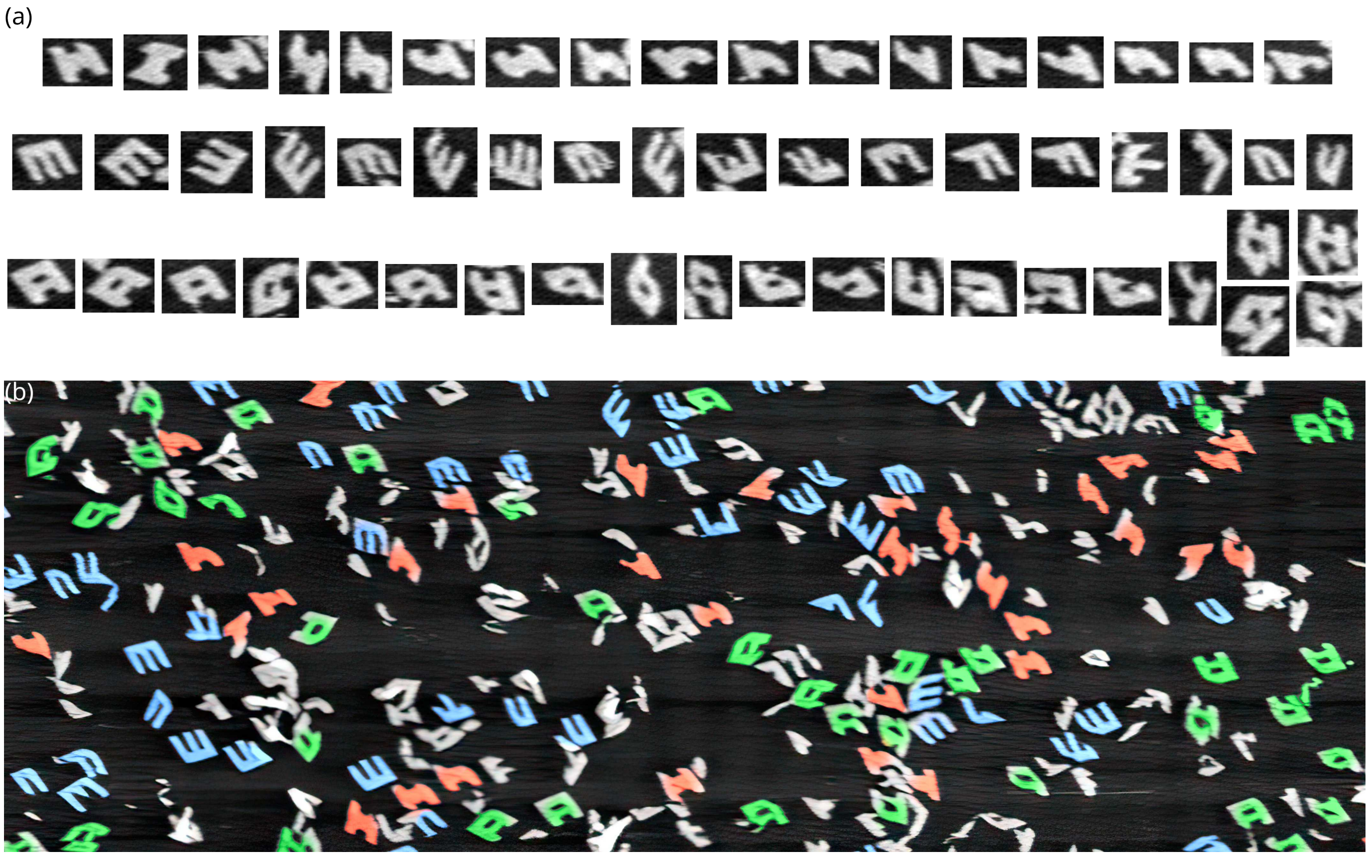

Figure S6.1: Examples of structures seen in AFM images. **a,** A collection of individual structures, shown from left to right in decreasing completeness, but still recognizable as H, A, or M. The far right for A shows a collection of structures with spiral defects, where each side of the circular region grew independently, rather than connecting to the other side. **b,** An example of shapes in an image of an equal-concentration sample with color added by identification (red for H, green for A, blue for M).

6.3.2 Counts averaged across co-authors, images and samples, normalized to shapes per 25 µm^2:

Pattern	H-1	H-2	A-1	A-2	M-1	M-2	total #	% target	%H	%A	%M
Hodgkin	4.17	41.00	0.00	0.00	0.00	0.92	46.08	98	98	0	2
Avogadro	0.00	0.00	8.25	21.25	0.00	0.75	30.25	98	0	98	2
Mitscherlich	0.00	0.17	0.00	0.08	14.00	36.92	51.17	100	0	0	100
Hopfield	13.92	30.08	0.00	0.00	0.00	0.33	44.33	99	99	0	1
Abbott	0.08	0.42	18.17	20.33	0.00	0.08	39.08	99	1	99	0
Moser	0.00	0.00	0.00	0.00	4.33	32.25	36.58	100	0	0	100
Horse	12.80	39.59	0.02	0.02	0.00	0.07	52.50	100	100	0	0
Anchovy	0.00	0.33	20.33	23.17	0.00	0.08	43.92	99	1	99	0
Mockingbird	0.48	3.44	1.88	3.29	1.96	7.54	18.58	51	21	28	51
Hazelnuts	7.67	41.83	0.42	2.08	0.00	0.17	52.17	95	95	5	0
Apples	0.00	0.00	21.92	23.67	0.00	0.00	45.58	100	0	100	0
Magnolia	0.00	2.47	3.28	4.17	13.36	29.47	52.75	81	5	14	81
Harom	5.33	47.83	0.08	1.25	0.08	1.17	55.75	95	95	2	2
Aon	0.05	1.18	13.50	17.09	2.34	10.80	44.95	68	3	68	29
Mbili	0.10	2.73	2.46	3.27	13.75	22.48	44.79	81	6	13	81
H	8.67	45.12	0.60	1.08	0.40	2.56	58.44	92	92	3	5
A	0.00	1.42	19.17	17.83	0.33	4.00	42.75	87	3	87	10
M	0.00	0.33	1.50	4.58	14.50	19.67	40.58	84	1	15	84
NoisyApple1	0.08	0.08	0.08	0.42	0.08	0.08	0.83	60	20	60	20
NoisyApple2	0.00	0.00	0.00	0.67	0.00	0.42	1.08	62	0	62	38
NoisyApple3	0.00	0.00	2.67	10.42	0.00	0.17	13.25	99	0	99	1
NoisyHorse1	7.50	30.50	0.08	0.00	0.00	0.00	38.08	100	100	0	0
NoisyHorse2	0.00	0.50	0.00	0.00	0.00	0.00	0.50	100	100	0	0
NoisyHorse3	0.00	3.75	0.00	0.00	0.00	0.00	3.75	100	100	0	0
ObscMagn1	0.00	0.00	0.00	0.00	0.00	0.25	0.25	100	0	0	100
ObscMagn2	0.00	0.00	0.00	0.00	0.00	0.00	0.00	nan	nan	nan	nan
ObscMagn3	0.00	0.00	0.58	1.67	2.33	15.42	20.00	89	0	11	89
ObscHazeln1	0.00	1.00	0.00	0.00	0.00	0.08	1.08	92	92	0	8
ObscHazeln2	0.42	9.33	0.00	0.33	0.42	0.92	11.42	85	85	3	12
ObscHazeln3	0.00	0.58	0.00	0.00	0.00	0.08	0.67	88	88	0	12
VarHarom1	0.00	0.00	0.00	0.00	0.00	0.00	0.00	nan	nan	nan	nan
VarHarom2	0.00	0.58	0.00	0.00	0.00	0.00	0.58	100	100	0	0
VarHarom3	0.00	0.00	0.00	0.00	0.00	0.00	0.00	nan	nan	nan	nan
VarHarom4	0.08	2.50	0.00	0.00	0.00	0.08	2.67	97	97	0	3
VarHarom5	0.00	1.08	0.08	0.58	0.00	0.17	1.92	57	57	35	9
VarHarom6	0.00	0.00	0.00	0.00	0.00	0.00	0.00	nan	nan	nan	nan
SHAM	26.72	107.89	73.16	49.28	51.10	41.26	349.41	100	39	35	26

6.4 Individual fluorescence and AFM results

6.4.1 Hodgkin

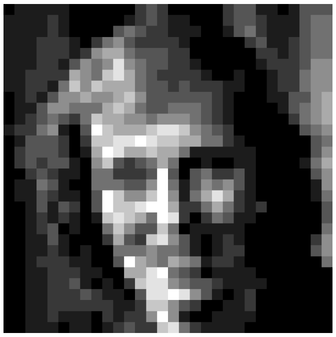

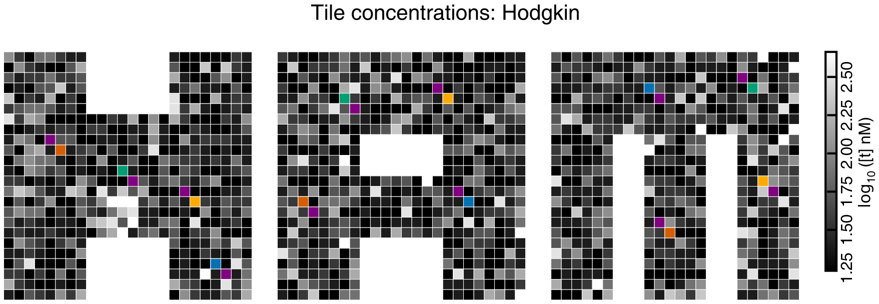

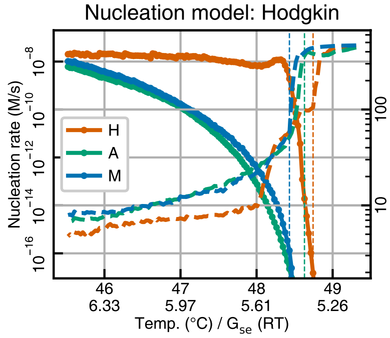

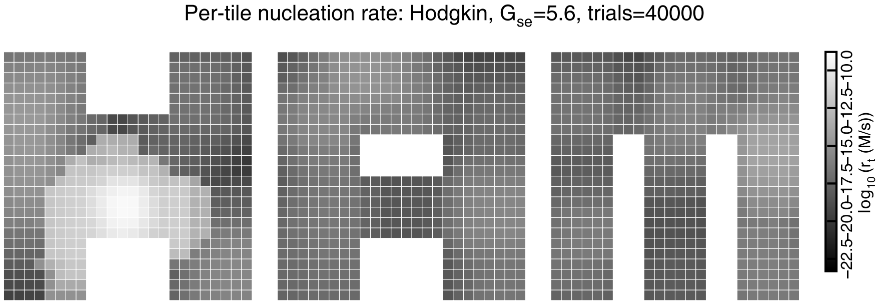

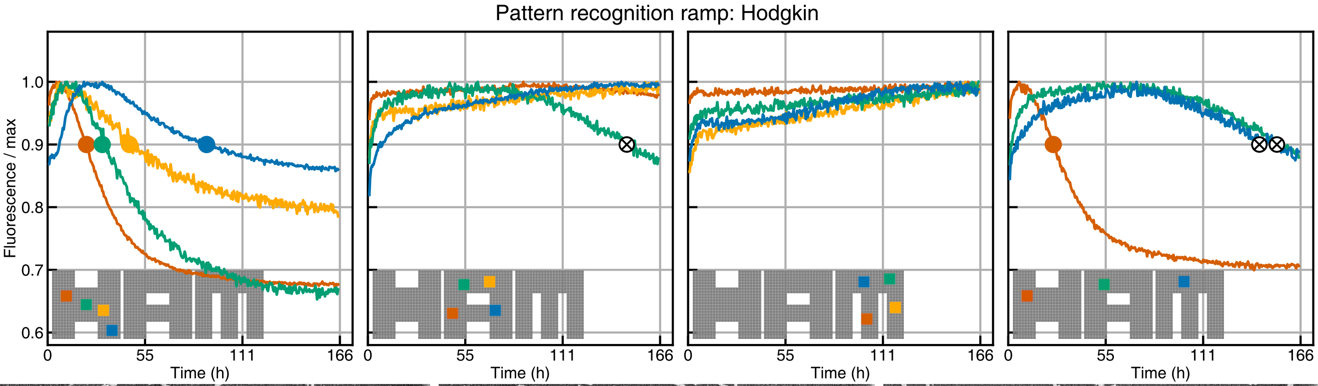

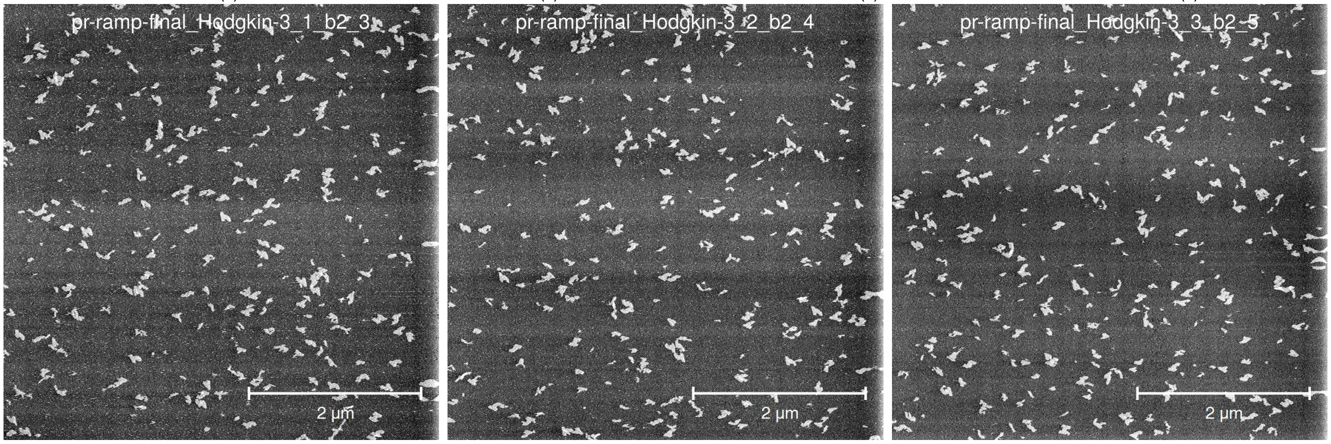

6.4.2 Avogadro

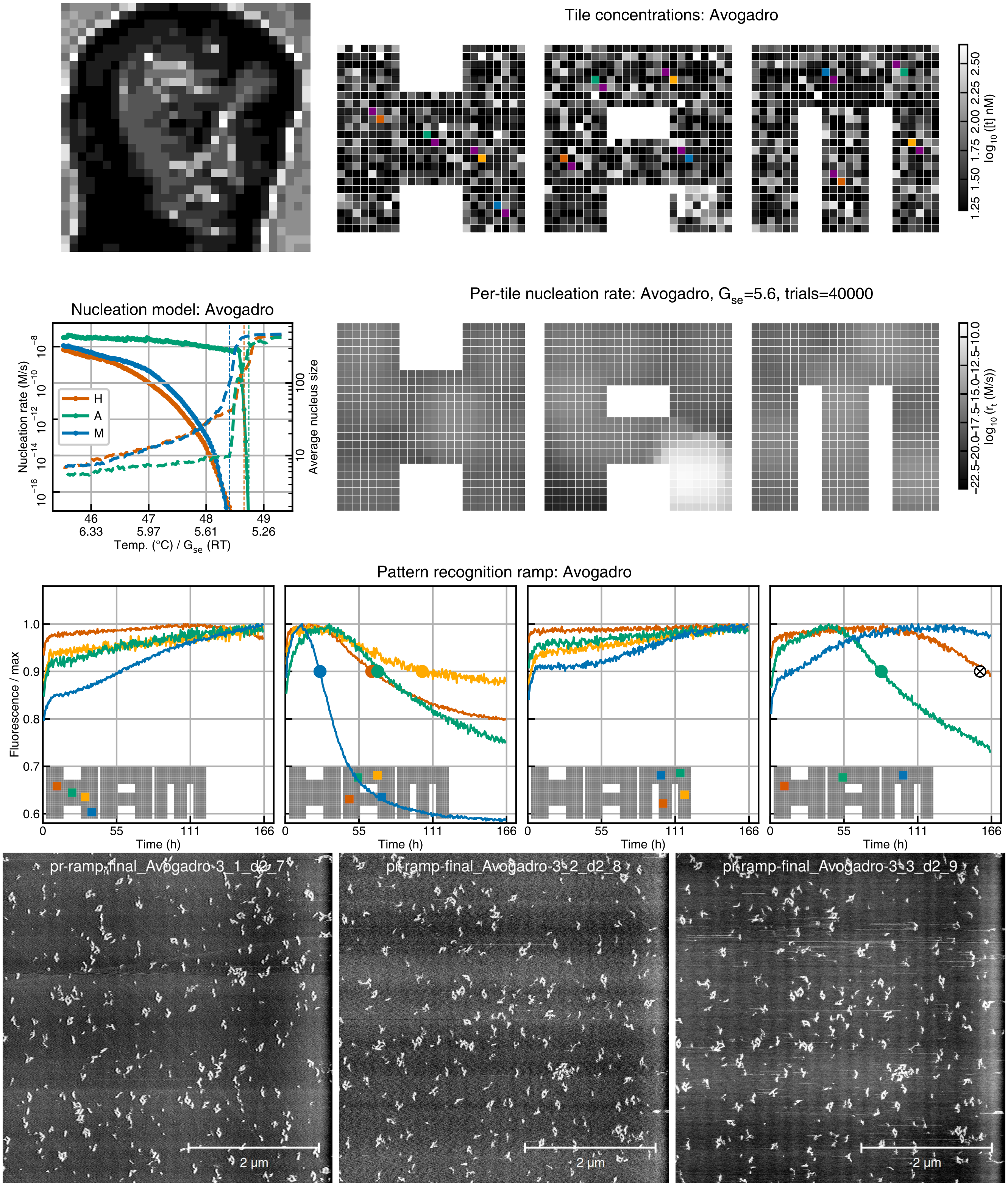

6.4.3 Mitscherlich

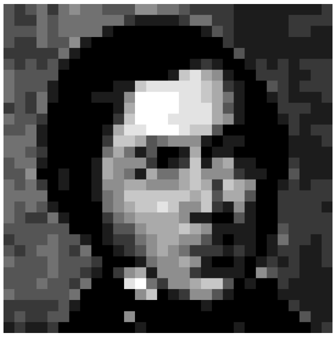

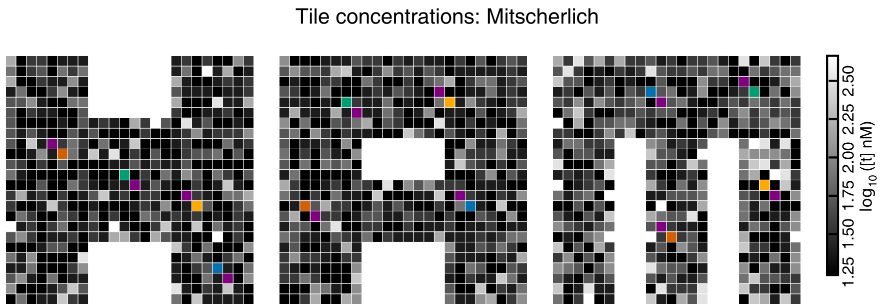

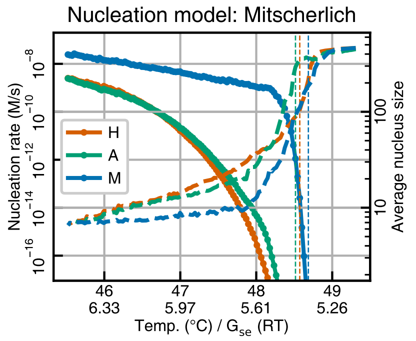

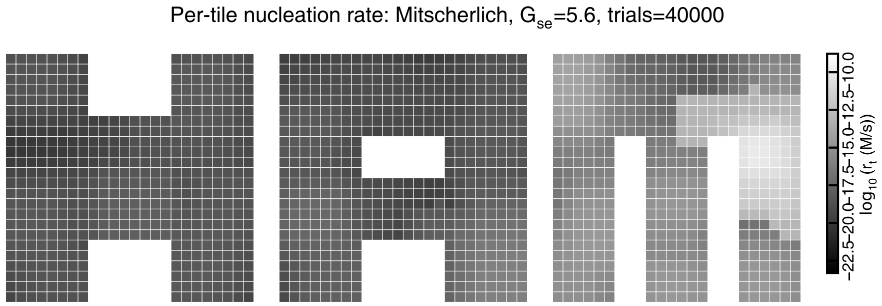

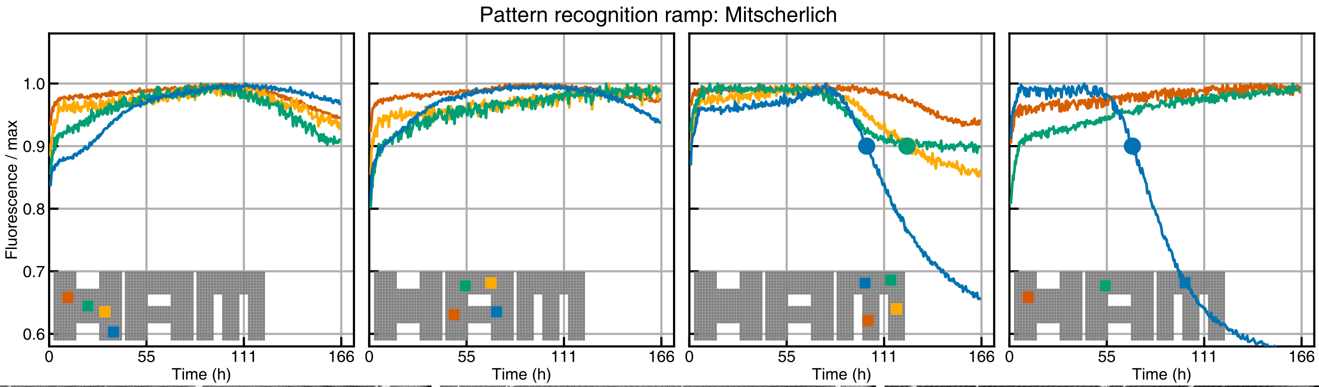

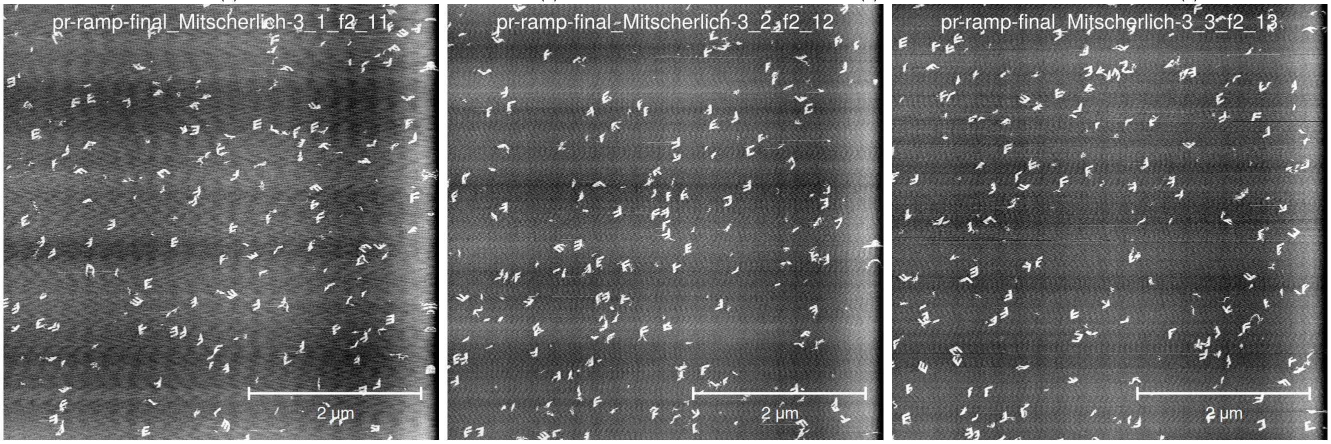

6.4.4 Hopfield

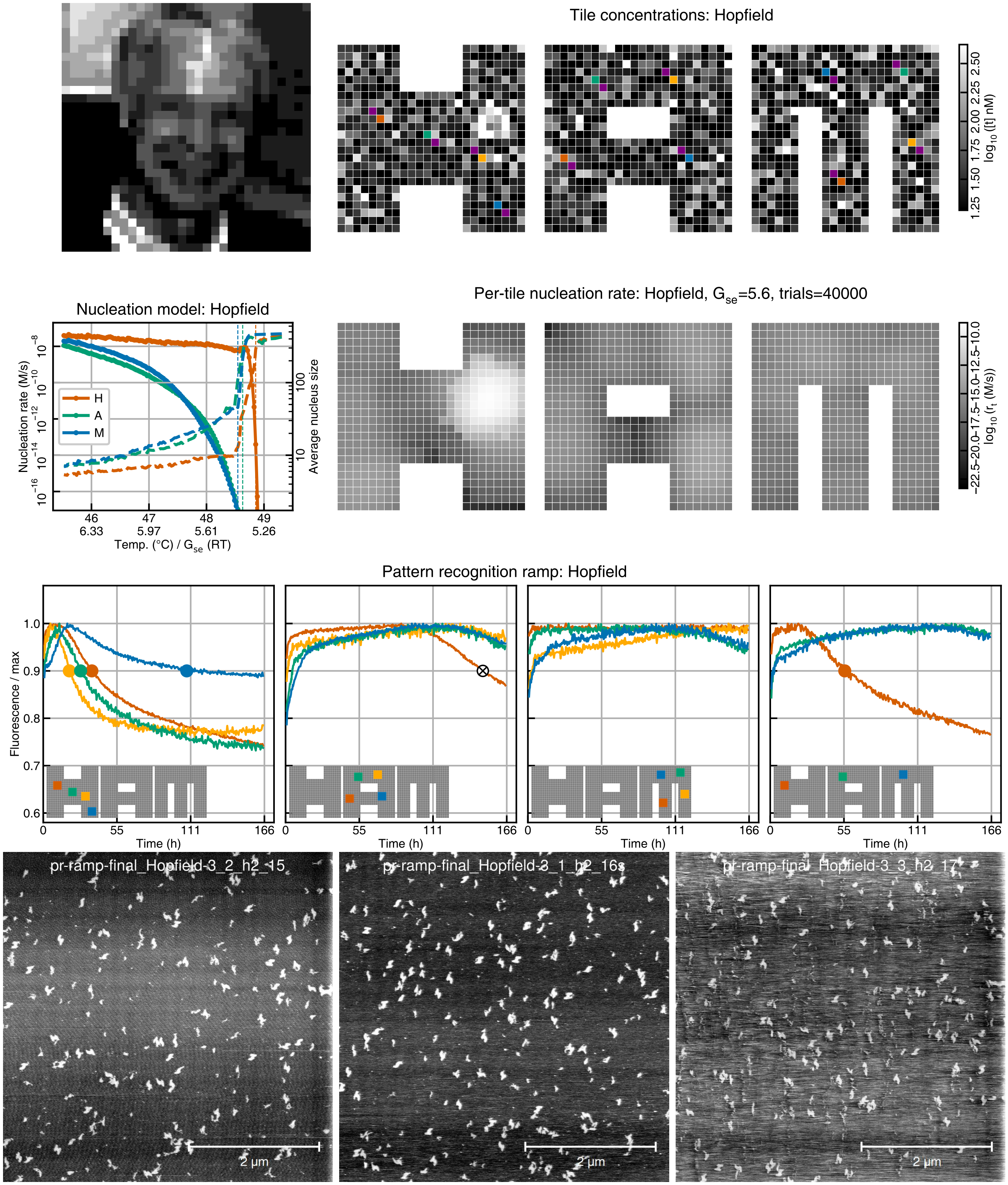

6.4.5 Abbott

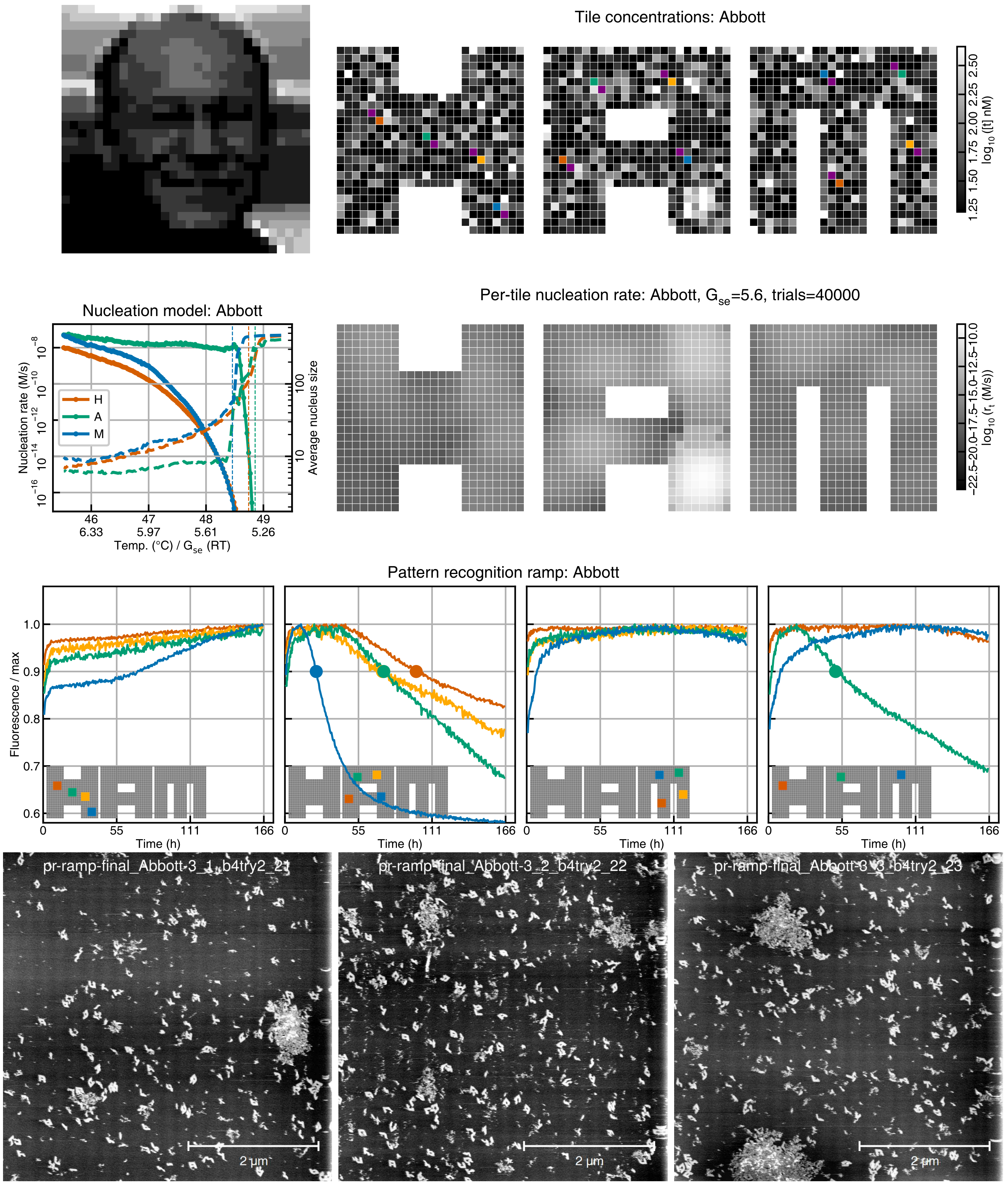
Tile concentrations: Abbott
$\log_{10}$ ([t] nM)
1.25 1.50 1.75 2.00 2.25 2.50
Nucleation model: Abbott
Nucleation rate (M/s)
Average nucleus size
H
A
M
Temp. (°C) / G_{se} (RT)
46 47 48 49
6.33 5.97 5.61 5.26
Per-tile nucleation rate: Abbott, G_{se}=5.6, trials=40000
$\log_{10}$ (r_t (M/s))
−22.5 −20.0 −17.5 −15.0 −12.5 −10.0
Pattern recognition ramp: Abbott
Fluorescence / max
Time (h)
0 55 111 166
pr-ramp-final_Abbott-3_1_b4try2_21
pr-ramp-final_Abbott-3_2_b4try2_22
pr-ramp-final_Abbott-3_3_b4try2_23
2 µm

6.4.6 Moser

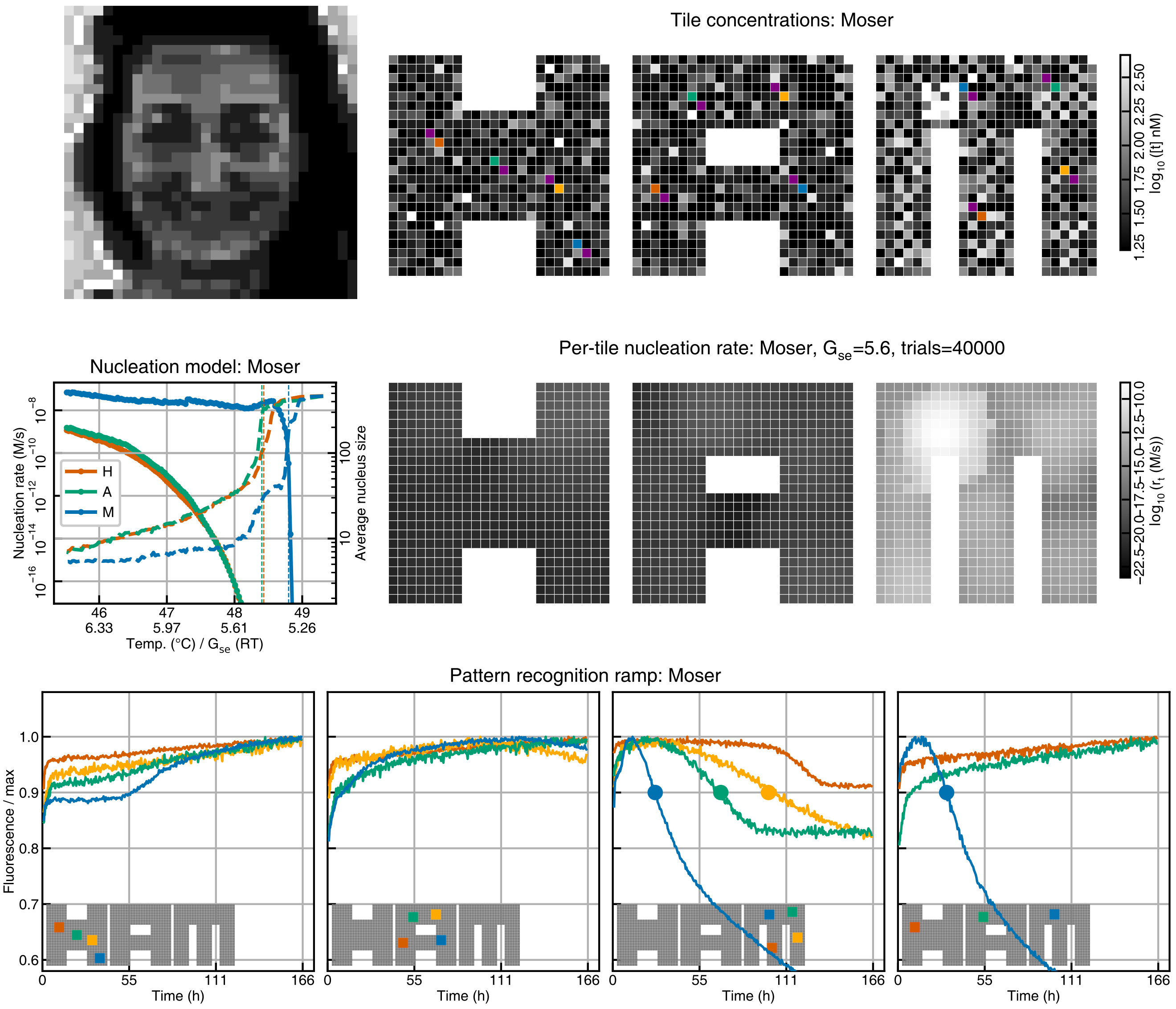
Tile concentrations: Moser
1.25 1.50 1.75 2.00 2.25 2.50
log10 ([t] nM)
Nucleation model: Moser
Nucleation rate (M/s)
Average nucleus size
H
A
M
46 47 48 49
6.33 5.97 5.61 5.26
Temp. (°C) / Gse (RT)
Per-tile nucleation rate: Moser, Gse=5.6, trials=40000
−22.5 −20.0 −17.5 −15.0 −12.5 −10.0
log10 (rt (M/s))
Pattern recognition ramp: Moser
Fluorescence / max
1.0 0.9 0.8 0.7 0.6
0 55 111 166
Time (h)

6.4.7 Horse

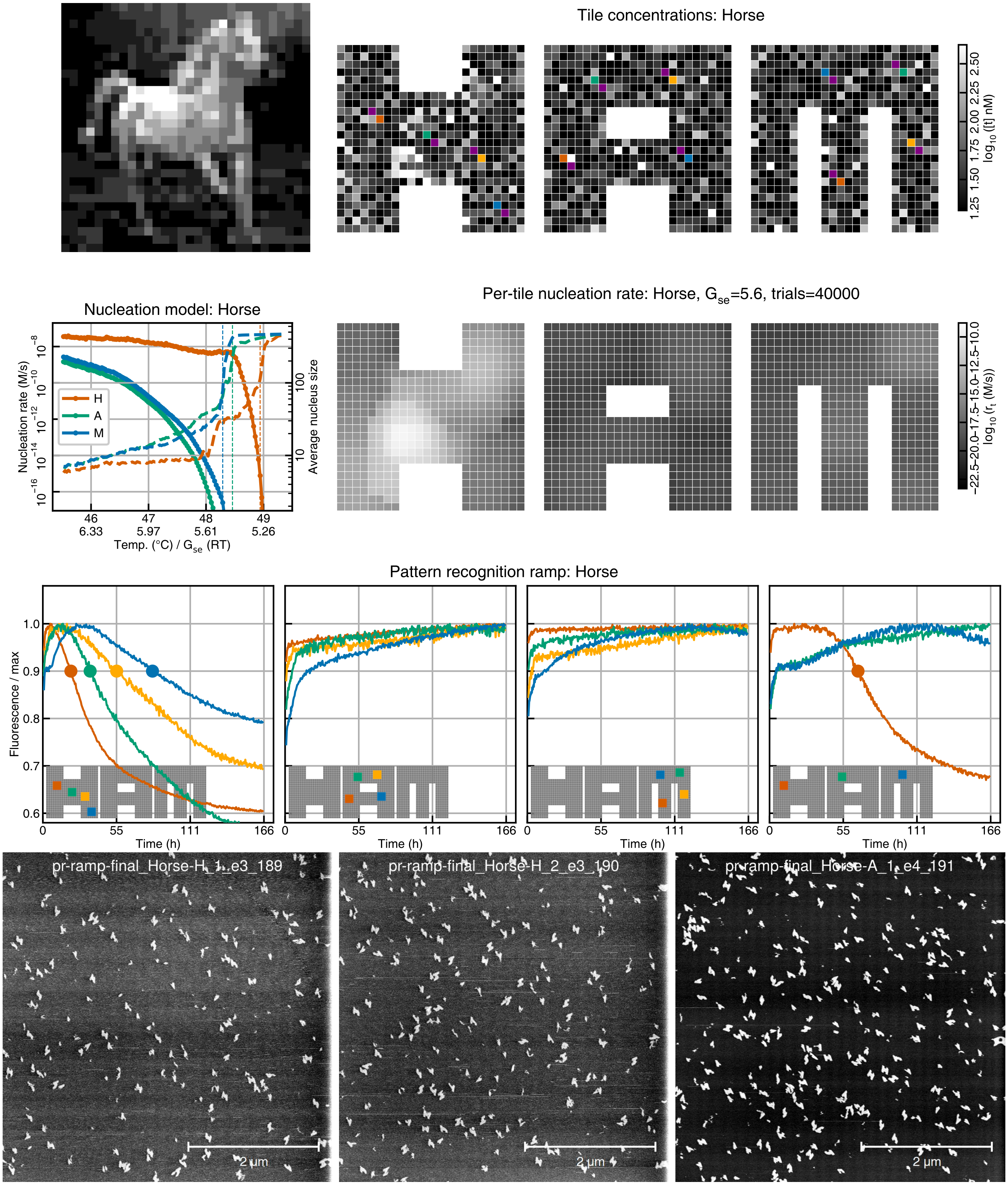
Tile concentrations: Horse
log10 ([t] nM)
Nucleation model: Horse
Nucleation rate (M/s)
Average nucleus size
H
A
M
Temp. (°C) / Gse (RT)
Per-tile nucleation rate: Horse, Gse=5.6, trials=40000
log10 (rt (M/s))
Pattern recognition ramp: Horse
Fluorescence / max
Time (h)
pr-ramp-final_Horse-H_1_e3_189
pr-ramp-final_Horse-H_2_e3_190
pr-ramp-final_Horse-A_1_e4_191
2 µm

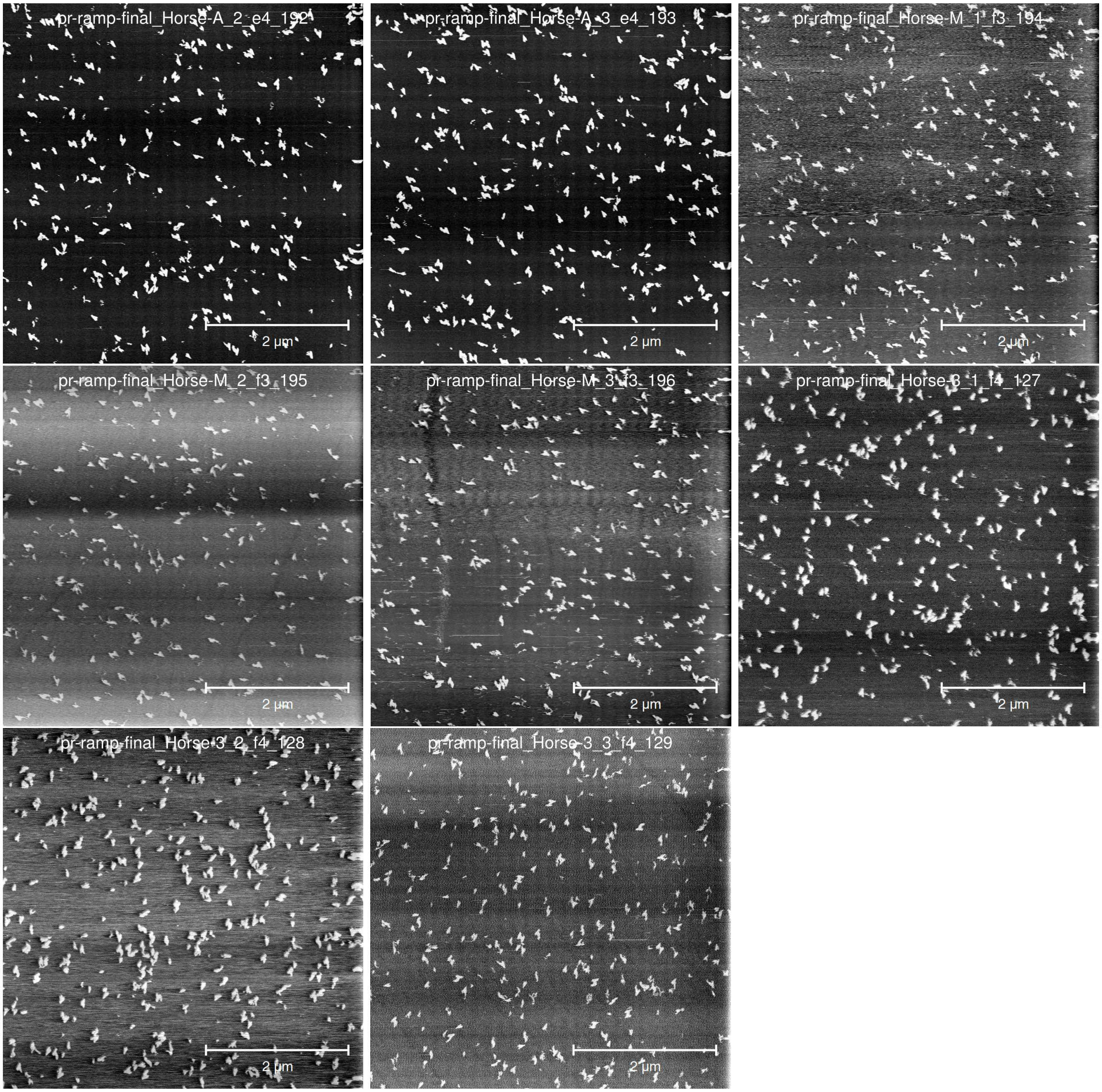
pr-ramp-final_Horse-A_2_e4_192
2 µm
pr-ramp-final_Horse-A_3_e4_193
2 µm
pr-ramp-final_Horse-M_1_f3_194
2 µm
pr-ramp-final_Horse-M_2_f3_195
2 µm
pr-ramp-final_Horse-M_3_f3_196
2 µm
pr-ramp-final_Horse-3_1_f4_127
2 µm
pr-ramp-final_Horse-3_2_f4_128
2 µm
pr-ramp-final_Horse-3_3_f4_129
2 µm

6.4.8 Anchovy

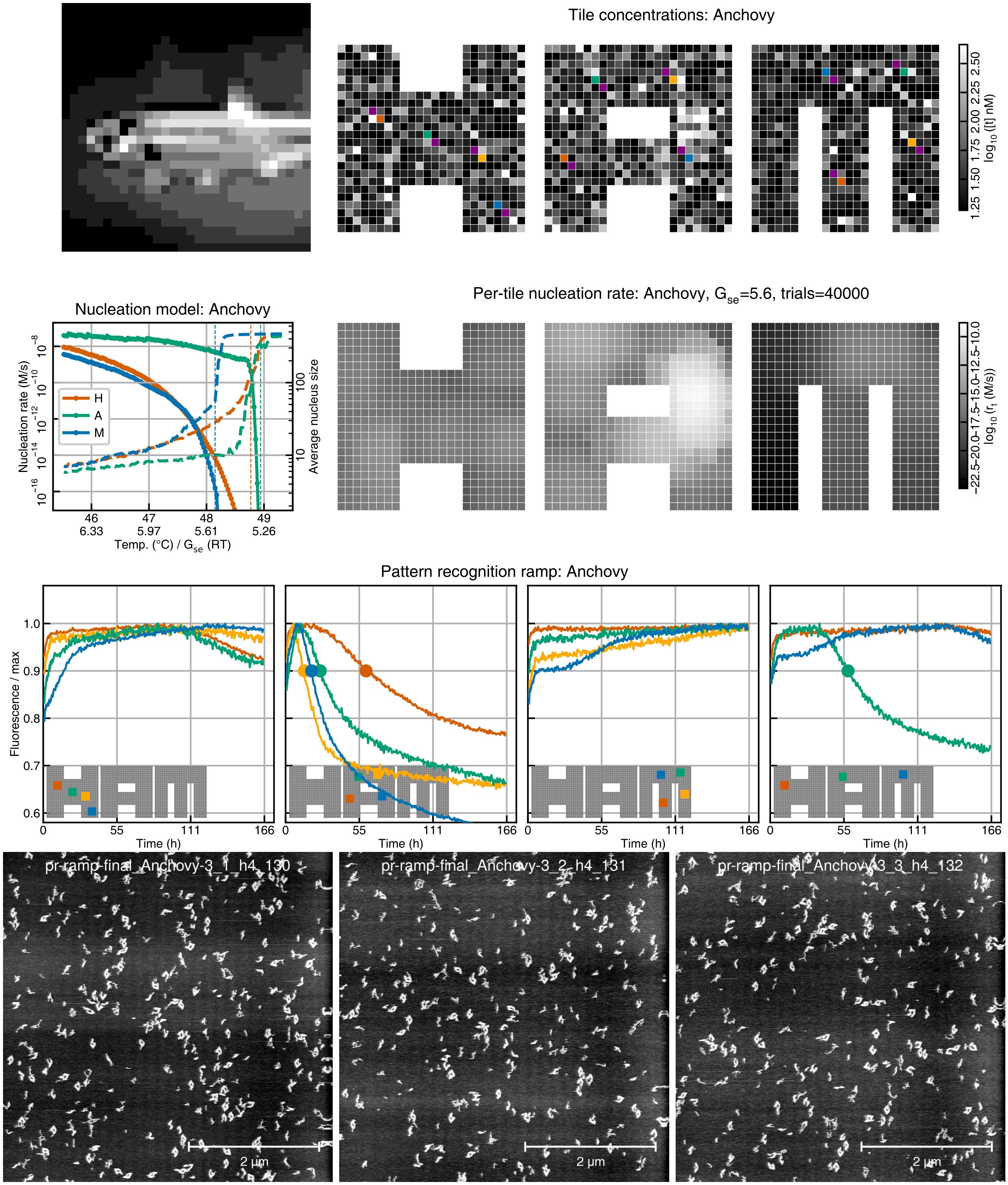

Tile concentrations: Anchovy
1.25 1.50 1.75 2.00 2.25 2.50
log10 ([t] nM)
Nucleation model: Anchovy
Nucleation rate (M/s)
Average nucleus size
H
A
M
46 47 48 49
6.33 5.97 5.61 5.26
Temp. (°C) / Gse (RT)
Per-tile nucleation rate: Anchovy, Gse=5.6, trials=40000
−22.5 −20.0 −17.5 −15.0 −12.5 −10.0
log10 (rt (M/s))
Pattern recognition ramp: Anchovy
Fluorescence / max
Time (h)
0 55 111 166
pr-ramp-final_Anchovy-3_1_h4_130
pr-ramp-final_Anchovy-3_2_h4_131
pr-ramp-final_Anchovy-3_3_h4_132
2 μm

6.4.9 Mockingbird

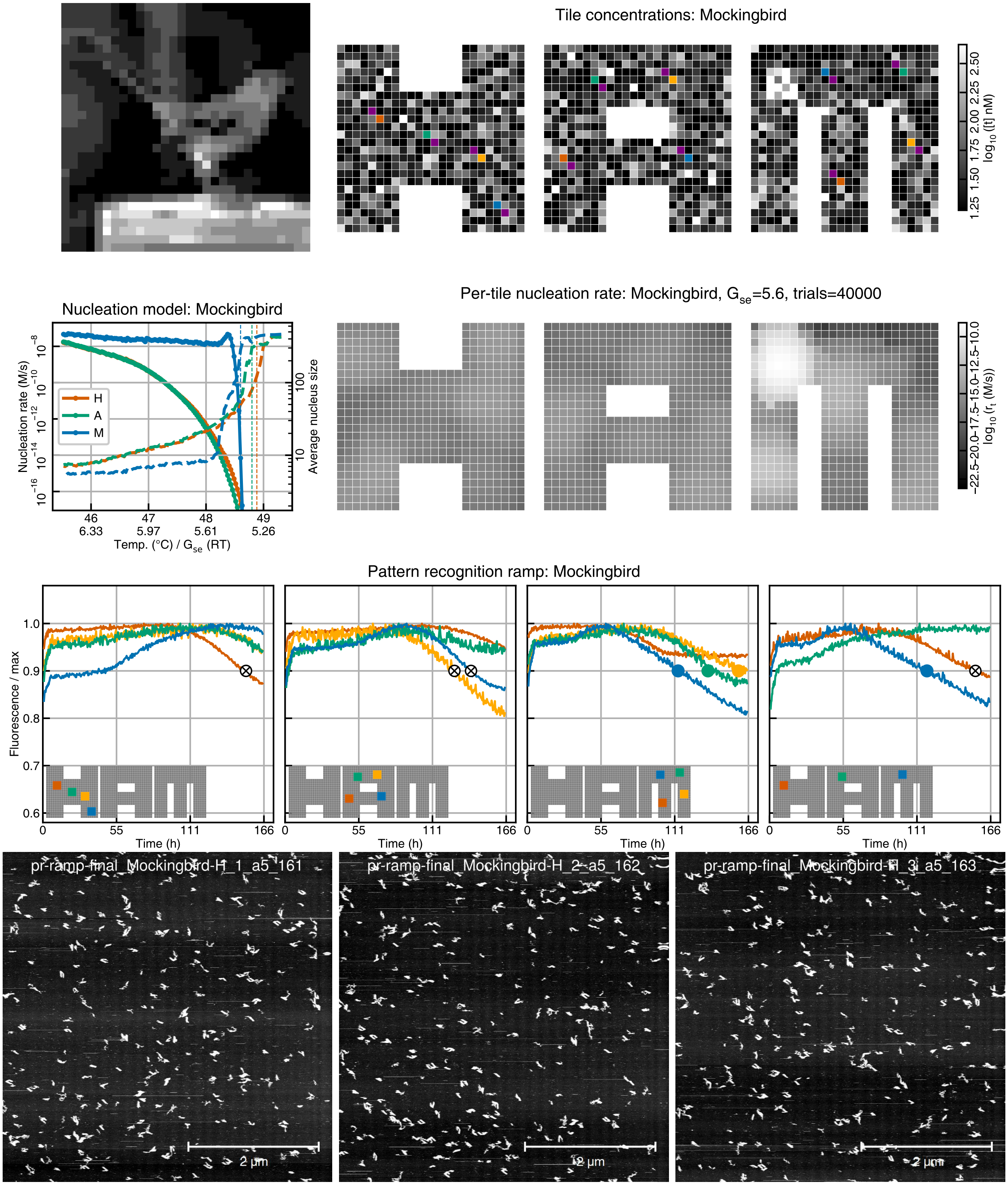

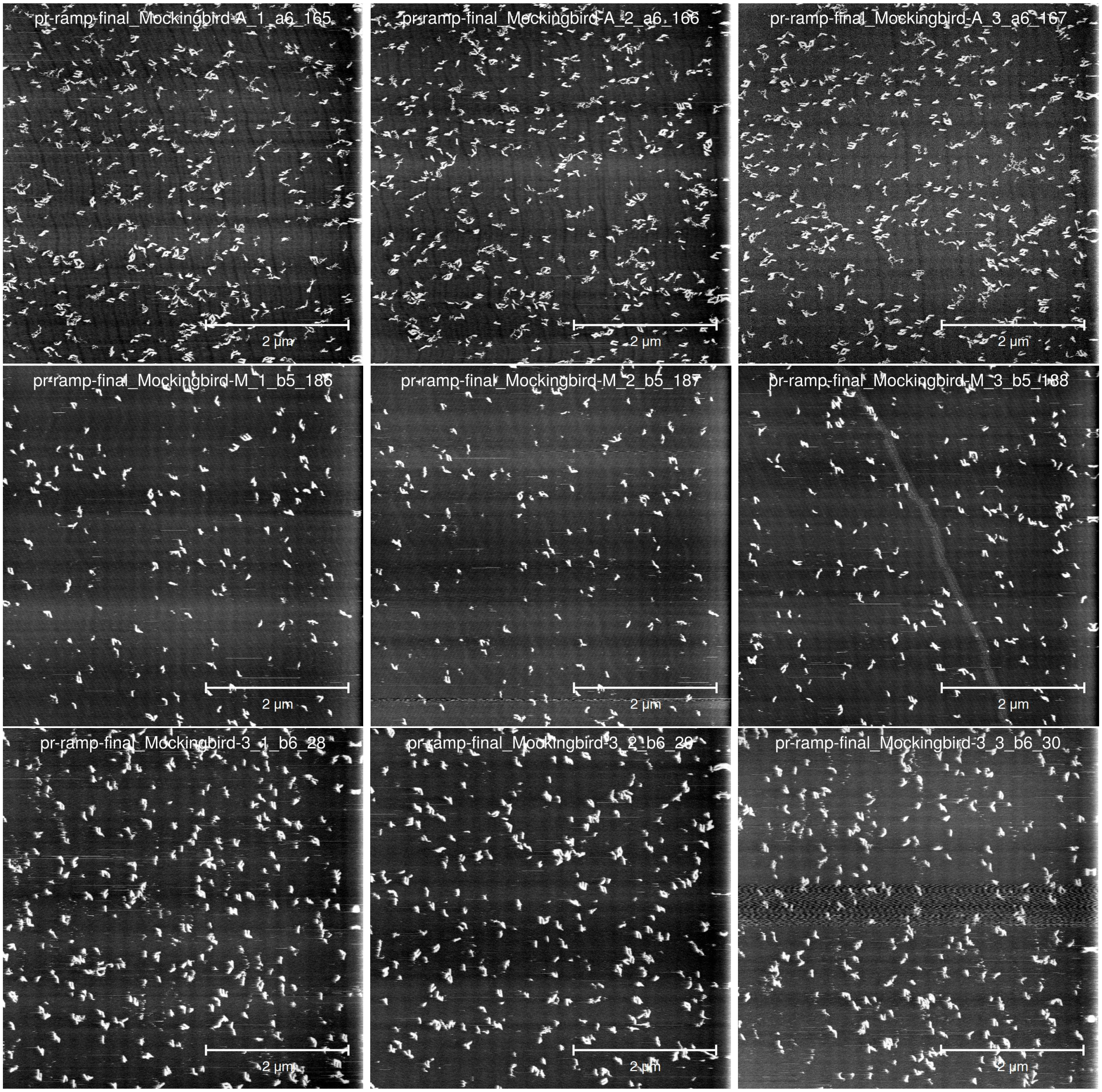
pr-ramp-final_Mockingbird-A_1_a6_165
2 µm
pr-ramp-final_Mockingbird-A_2_a6_166
2 µm
pr-ramp-final_Mockingbird-A_3_a6_167
2 µm
pr-ramp-final_Mockingbird-M_1_b5_186
2 µm
pr-ramp-final_Mockingbird-M_2_b5_187
2 µm
pr-ramp-final_Mockingbird-M_3_b5_188
2 µm
pr-ramp-final_Mockingbird-3_1_b6_28
2 µm
pr-ramp-final_Mockingbird-3_2_b6_29
2 µm
pr-ramp-final_Mockingbird-3_3_b6_30
2 µm

6.4.10 Hazelnuts

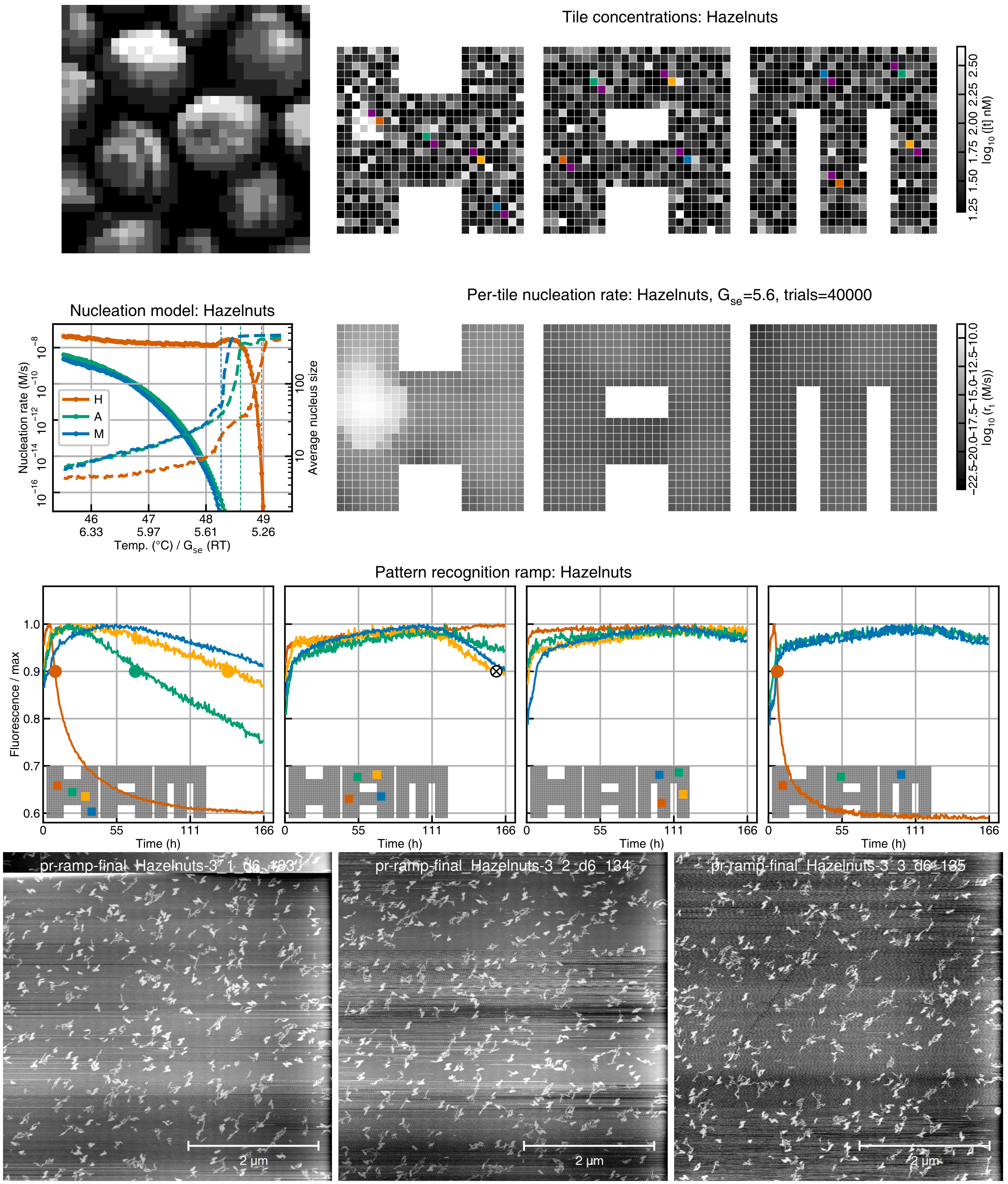

6.4.11 Apples

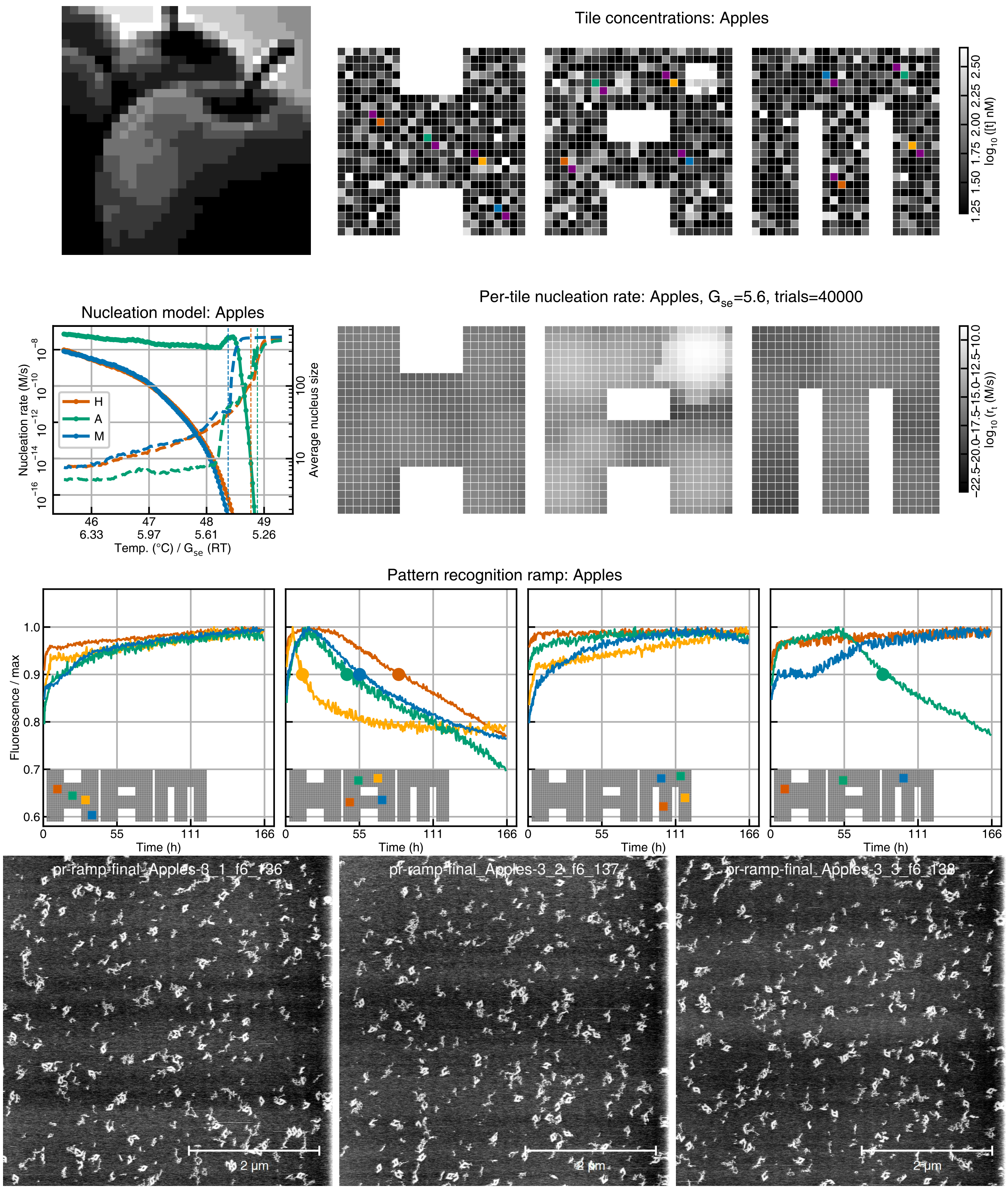
Tile concentrations: Apples
1.25 1.50 1.75 2.00 2.25 2.50
log10([t] nM)
Nucleation model: Apples
Nucleation rate (M/s)
Average nucleus size
H
A
M
46 47 48 49
6.33 5.97 5.61 5.26
Temp. (°C) / Gse (RT)
Per-tile nucleation rate: Apples, Gse=5.6, trials=40000
−22.5 −20.0 −17.5 −15.0 −12.5 −10.0
log10 (rt (M/s))
Pattern recognition ramp: Apples
Fluorescence / max
1.0 0.9 0.8 0.7 0.6
0 55 111 166
Time (h)
pr-ramp-final_Apples-3_1_f6_136
pr-ramp-final_Apples-3_2_f6_137
pr-ramp-final_Apples-3_3_f6_138
2 µm

6.4.12 Magnolia

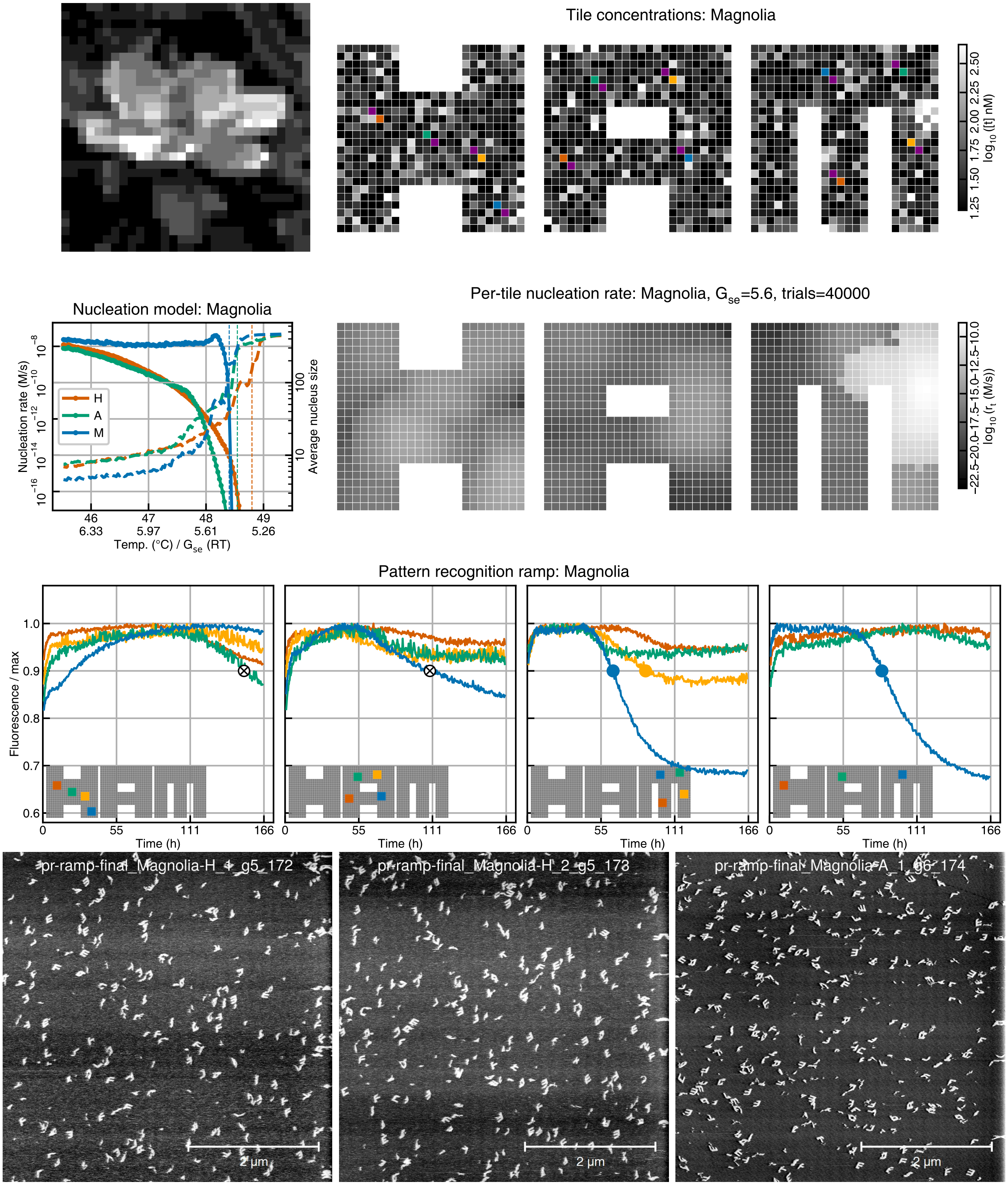

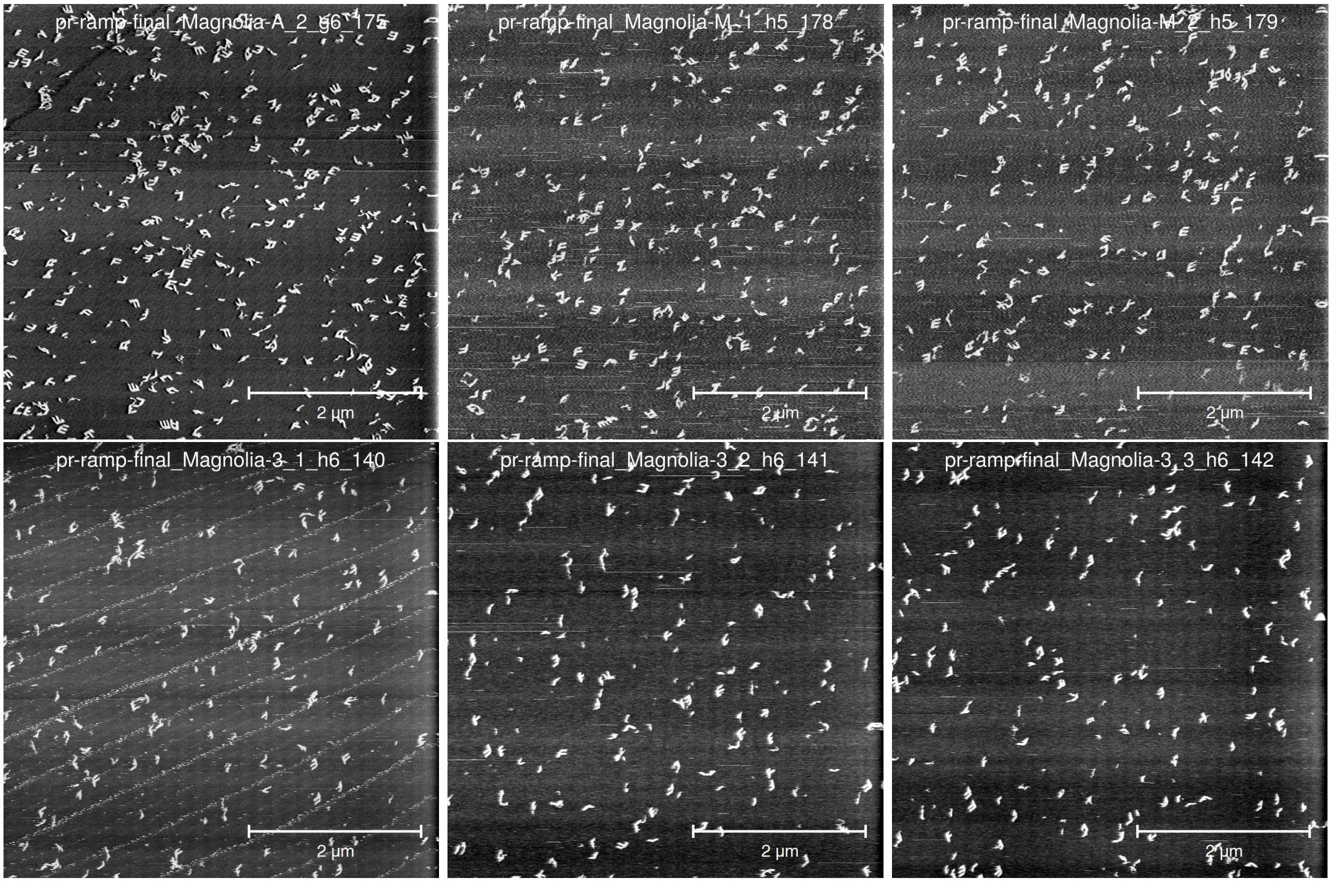
pr-ramp-final_Magnolia-A_2_g6_175
2 µm
pr-ramp-final_Magnolia-M_1_h5_178
2 µm
pr-ramp-final_Magnolia-M_2_h5_179
2 µm
pr-ramp-final_Magnolia-3_1_h6_140
2 µm
pr-ramp-final_Magnolia-3_2_h6_141
2 µm
pr-ramp-final_Magnolia-3_3_h6_142
2 µm

6.4.13 Harom

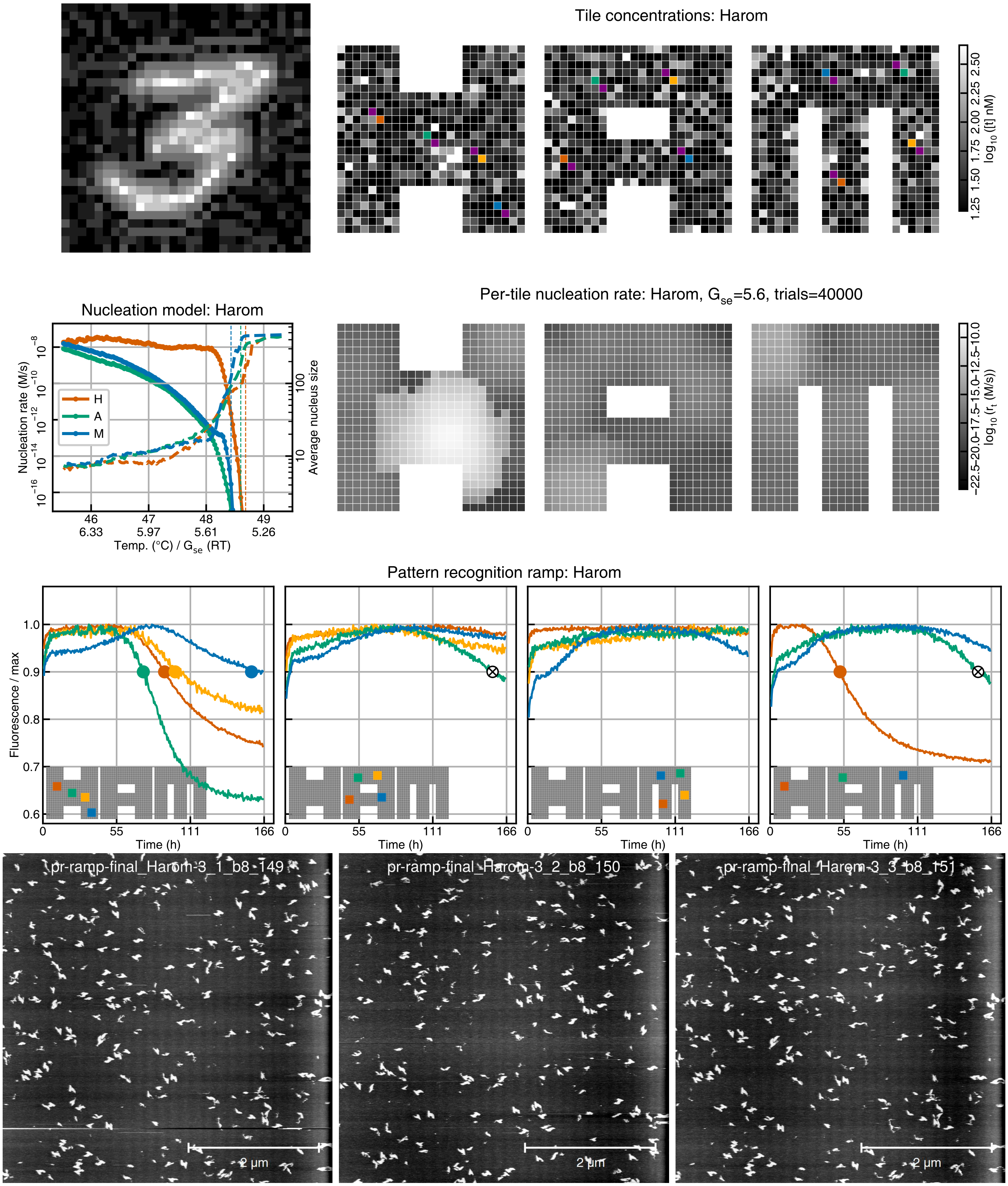

Tile concentrations: Harom
$\log_{10}$ ([t] nM)
1.25 1.50 1.75 2.00 2.25 2.50
Nucleation model: Harom
Nucleation rate (M/s)
Average nucleus size
H
A
M
Temp. (°C) / G_{se} (RT)
46 47 48 49
6.33 5.97 5.61 5.26
Per-tile nucleation rate: Harom, G_{se}=5.6, trials=40000
$\log_{10}$ (r_t (M/s))
−22.5 −20.0 −17.5 −15.0 −12.5 −10.0
Pattern recognition ramp: Harom
Fluorescence / max
Time (h)
0 55 111 166
pr-ramp-final_Harom-3_1_b8_149
pr-ramp-final_Harom-3_2_b8_150
pr-ramp-final_Harom-3_3_b8_151
2 µm

6.4.14 Aon

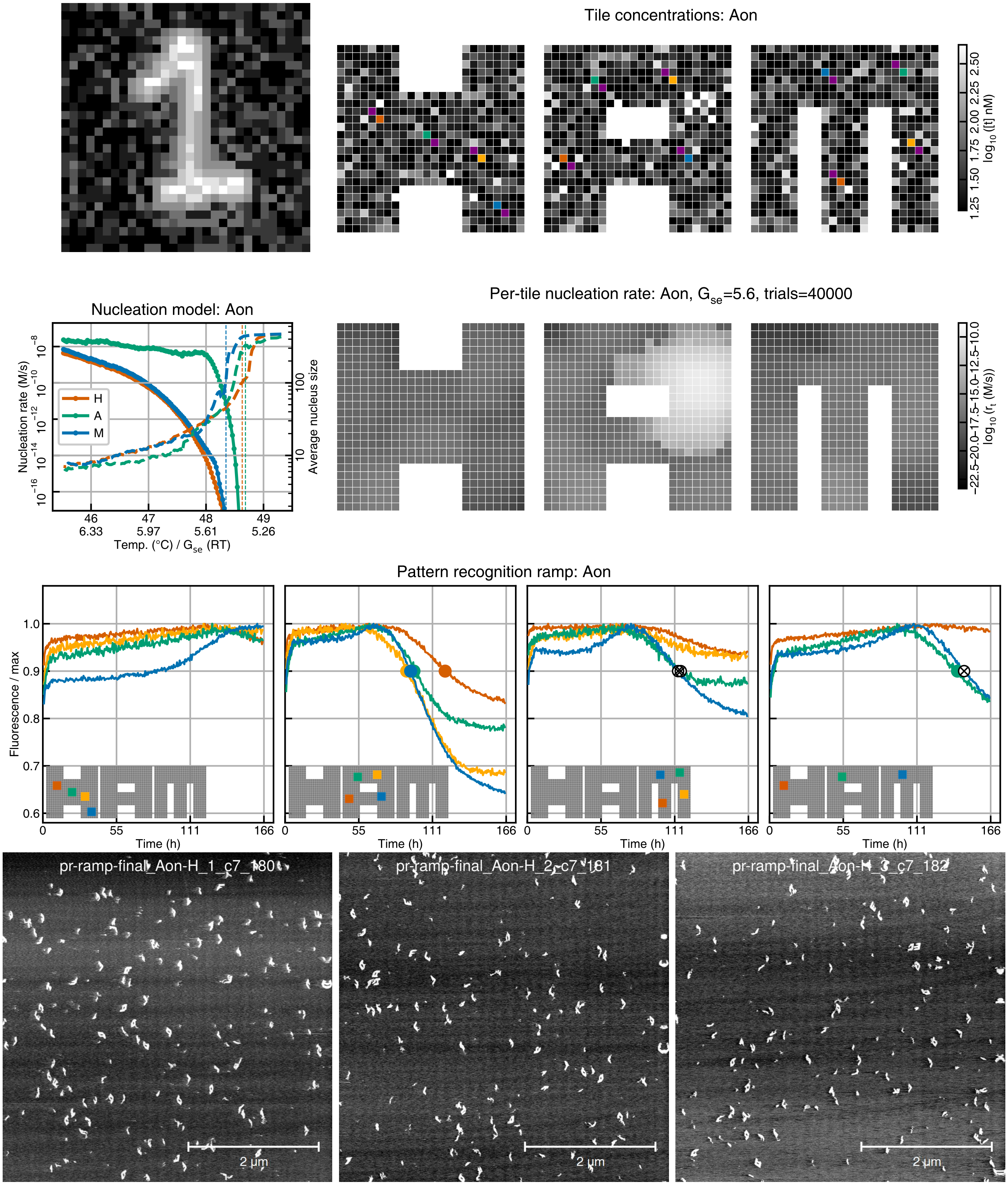

Tile concentrations: Aon
1.25 1.50 1.75 2.00 2.25 2.50
log10 ([t] nM)
Nucleation model: Aon
Nucleation rate (M/s)
Average nucleus size
H
A
M
46 6.33
47 5.97
48 5.61
49 5.26
Temp. (°C) / Gse (RT)
Per-tile nucleation rate: Aon, Gse=5.6, trials=40000
−22.5 −20.0 −17.5 −15.0 −12.5 −10.0
log10 (rt (M/s))
Pattern recognition ramp: Aon
Fluorescence / max
1.0
0.9
0.8
0.7
0.6
0
55
111
166
Time (h)
pr-ramp-final_Aon-H_1_c7_180
pr-ramp-final_Aon-H_2_c7_181
pr-ramp-final_Aon-H_3_c7_182
2 µm

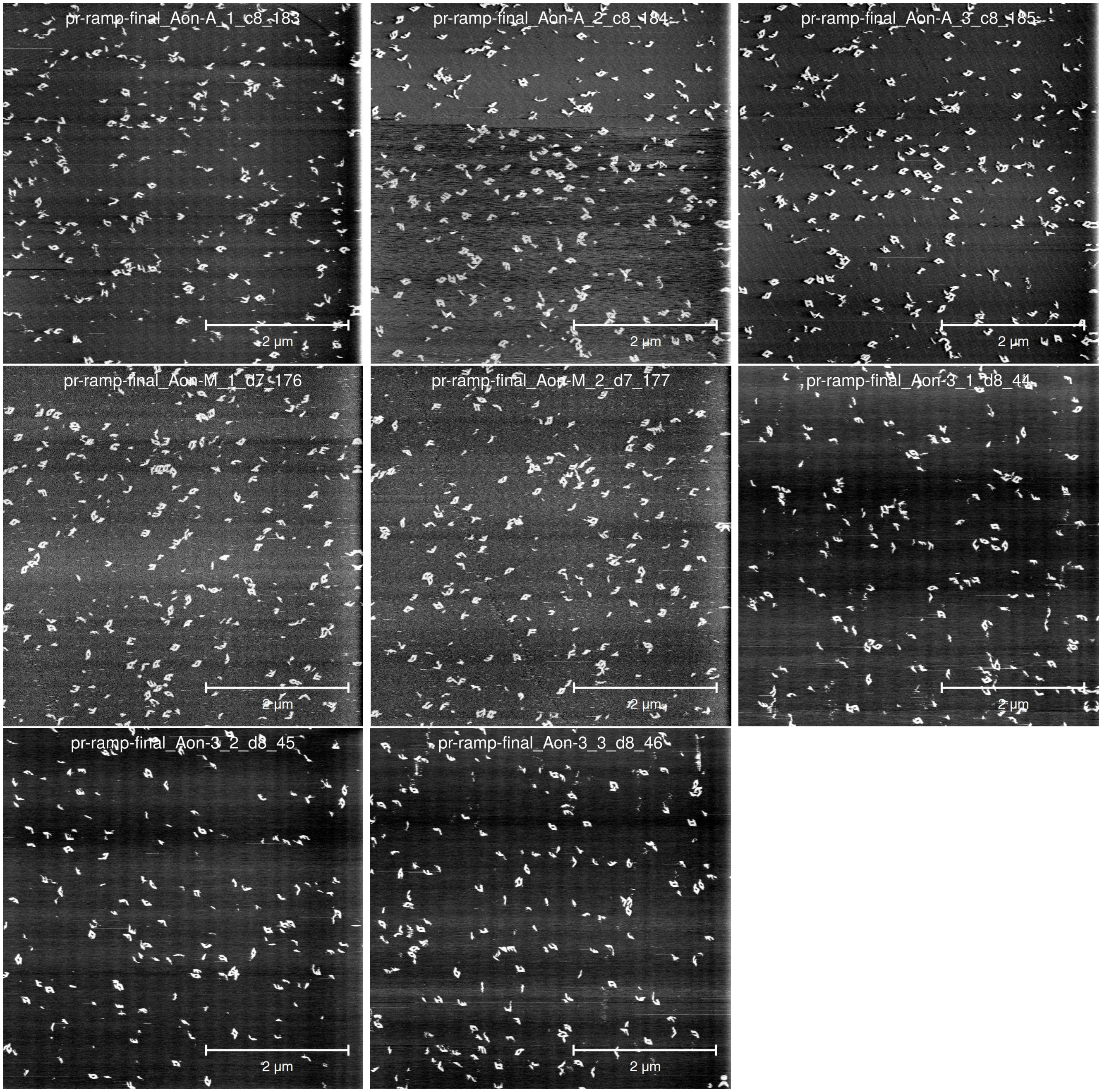
pr-ramp-final_Aon-A_1_c8_183
2 µm
pr-ramp-final_Aon-A_2_c8_184
2 µm
pr-ramp-final_Aon-A_3_c8_185
2 µm
pr-ramp-final_Aon-M_1_d7_176
2 µm
pr-ramp-final_Aon-M_2_d7_177
2 µm
pr-ramp-final_Aon-3_1_d8_44
2 µm
pr-ramp-final_Aon-3_2_d8_45
2 µm
pr-ramp-final_Aon-3_3_d8_46
2 µm

6.4.15 Mbili

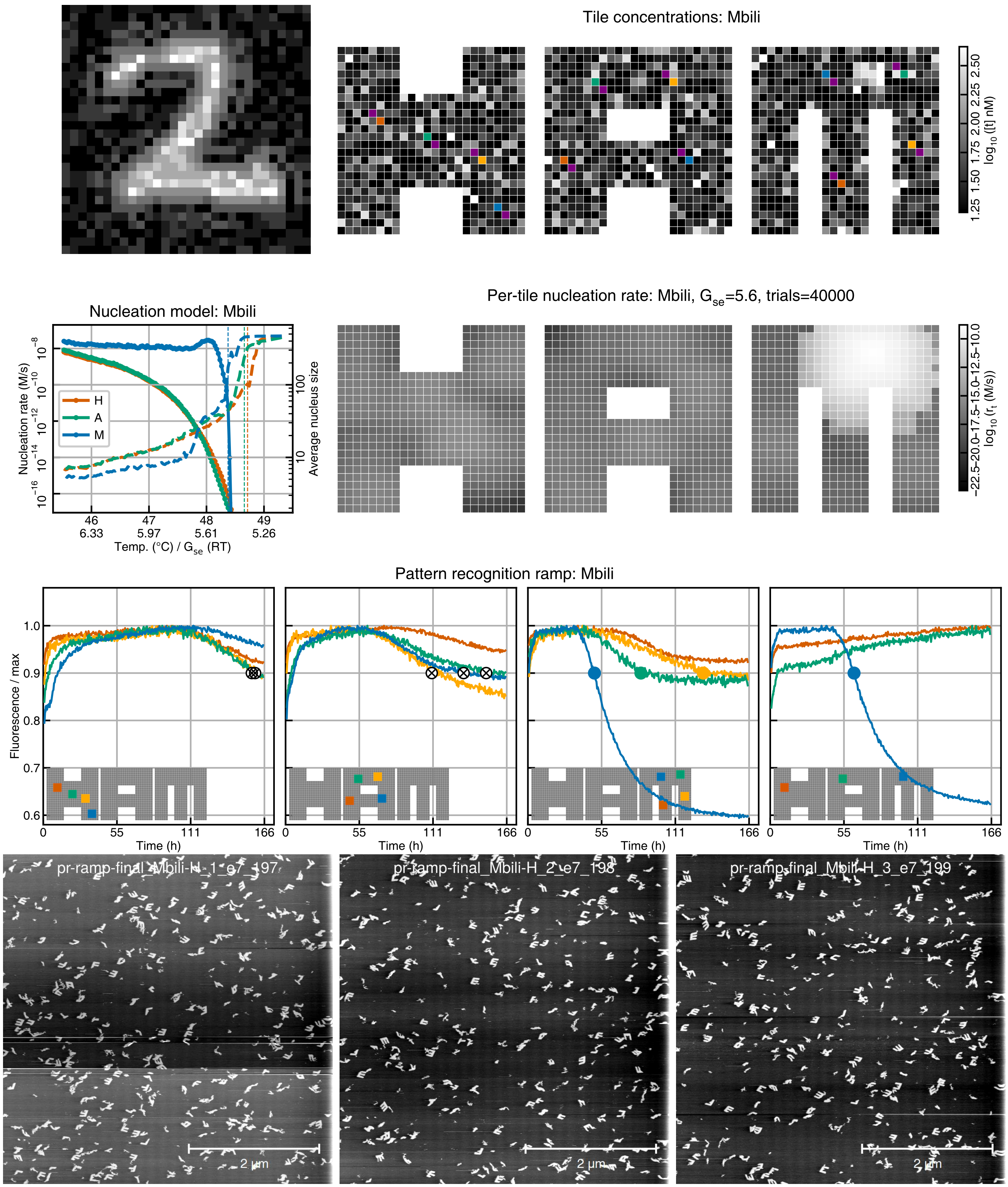
Tile concentrations: Mbili
1.25 1.50 1.75 2.00 2.25 2.50
log10 ([t] nM)
Nucleation model: Mbili
Nucleation rate (M/s)
Average nucleus size
H
A
M
46 47 48 49
6.33 5.97 5.61 5.26
Temp. (°C) / Gse (RT)
Per-tile nucleation rate: Mbili, Gse=5.6, trials=40000
−22.5 −20.0 −17.5 −15.0 −12.5 −10.0
log10 (rt (M/s))
Pattern recognition ramp: Mbili
Fluorescence / max
1.0 0.9 0.8 0.7 0.6
0 55 111 166
Time (h)
pr-ramp-final_Mbili-H_1_e7_197
pr-ramp-final_Mbili-H_2_e7_198
pr-ramp-final_Mbili-H_3_e7_199
2 µm

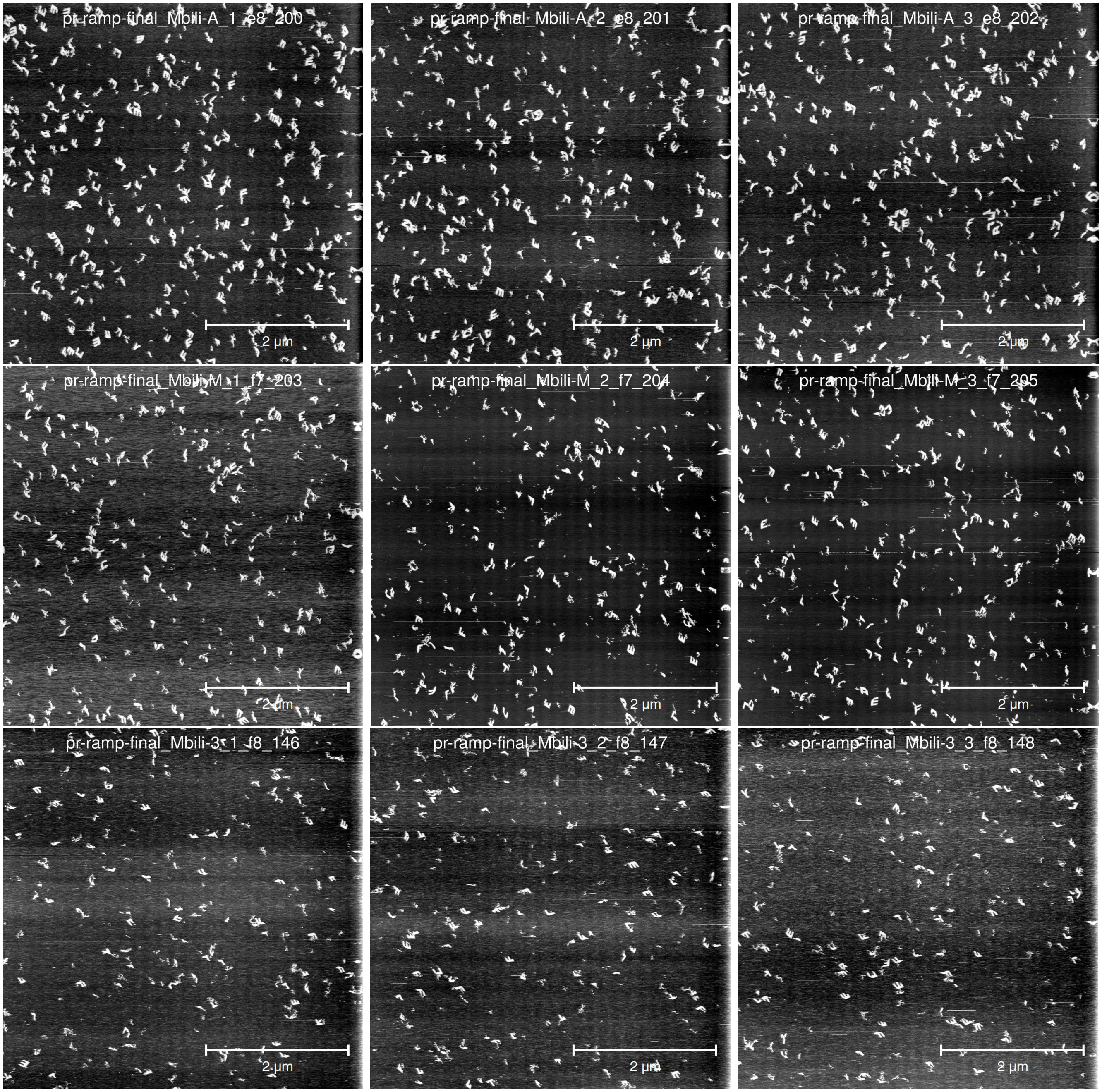
pr-ramp-final_Mbili-A_1_e8_200
2 µm
pr-ramp-final_Mbili-A_2_e8_201
2 µm
pr-ramp-final_Mbili-A_3_e8_202
2 µm
pr-ramp-final_Mbili-M_1_f7_203
2 µm
pr-ramp-final_Mbili-M_2_f7_204
2 µm
pr-ramp-final_Mbili-M_3_f7_205
2 µm
pr-ramp-final_Mbili-3_1_f8_146
2 µm
pr-ramp-final_Mbili-3_2_f8_147
2 µm
pr-ramp-final_Mbili-3_3_f8_148
2 µm

6.4.16 LetterH

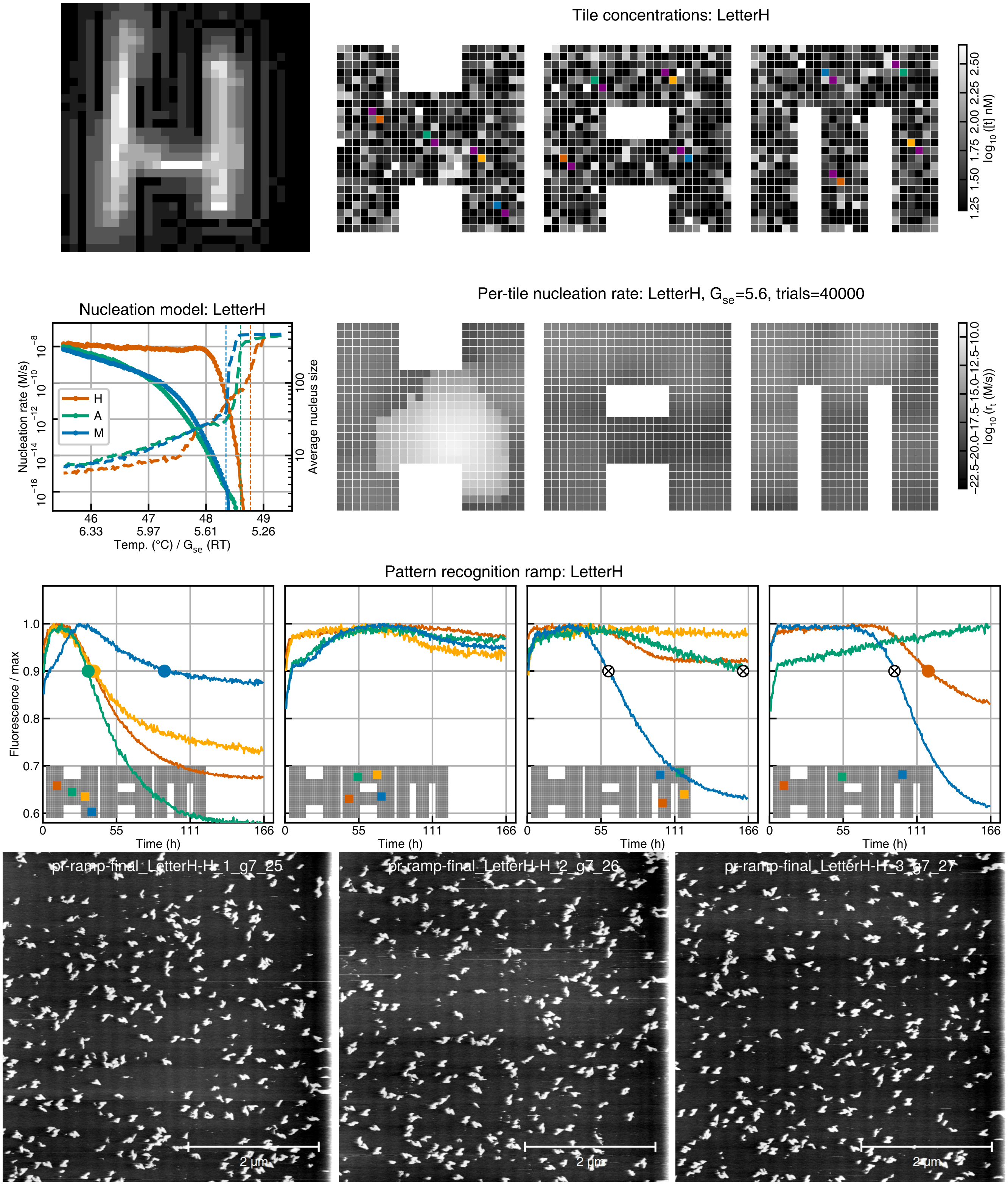

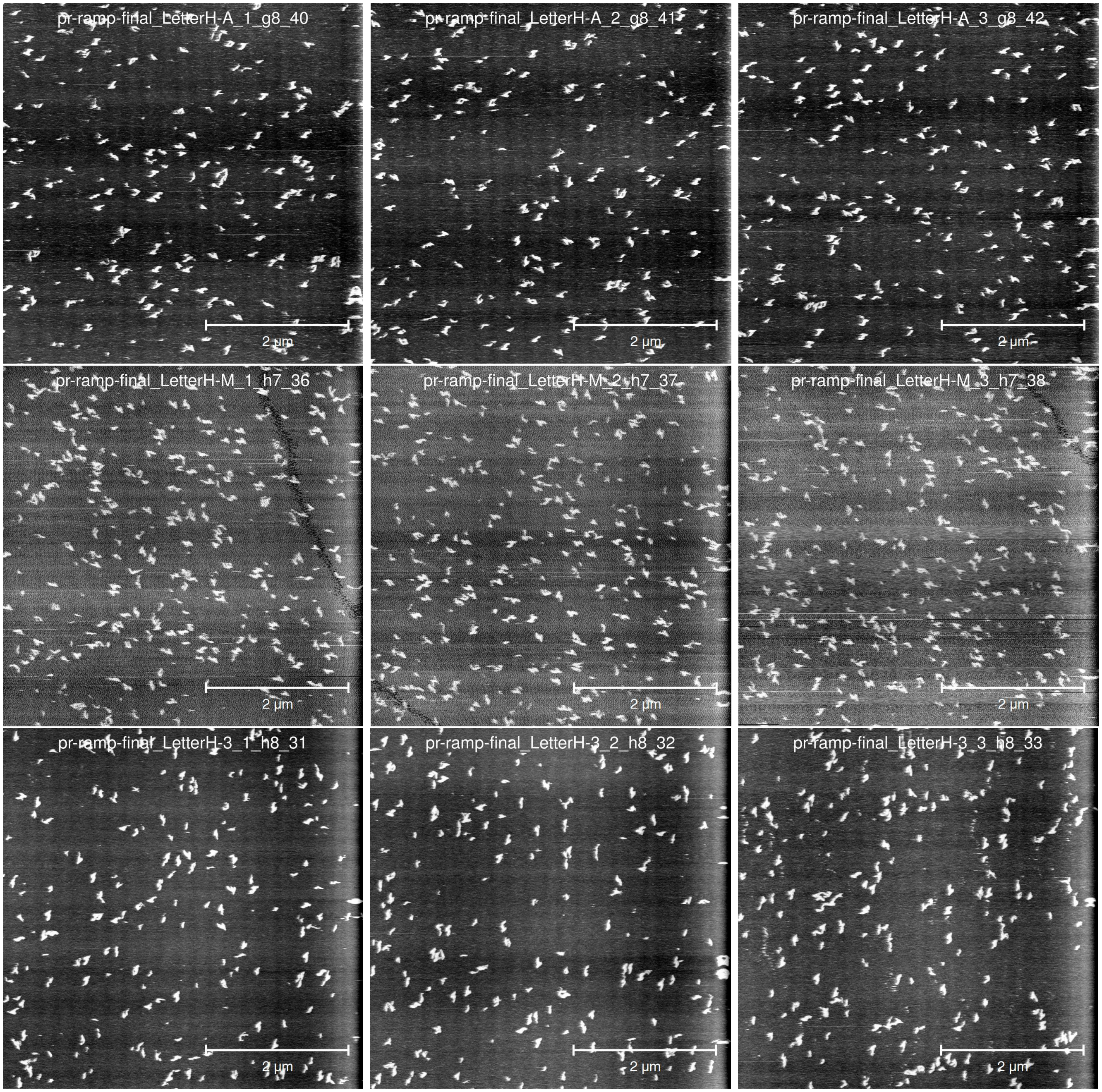
pr-ramp-final_LetterH-A_1_g8_40
2 μm
pr-ramp-final_LetterH-A_2_g8_41
2 μm
pr-ramp-final_LetterH-A_3_g8_42
2 μm
pr-ramp-final_LetterH-M_1_h7_36
2 μm
pr-ramp-final_LetterH-M_2_h7_37
2 μm
pr-ramp-final_LetterH-M_3_h7_38
2 μm
pr-ramp-final_LetterH-3_1_h8_31
2 μm
pr-ramp-final_LetterH-3_2_h8_32
2 μm
pr-ramp-final_LetterH-3_3_h8_33
2 μm

6.4.17 LetterA

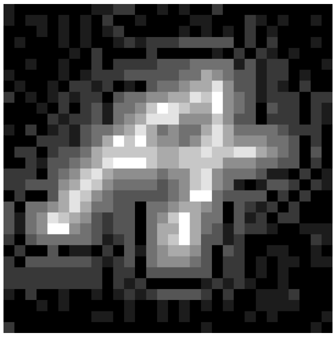

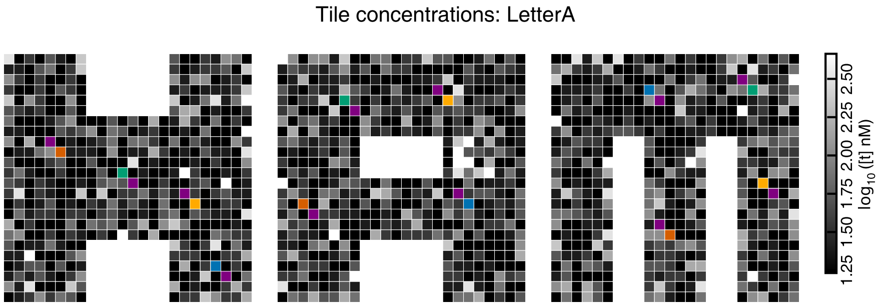

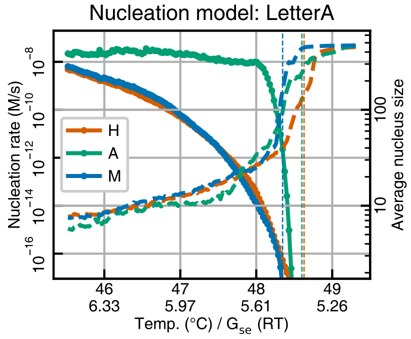

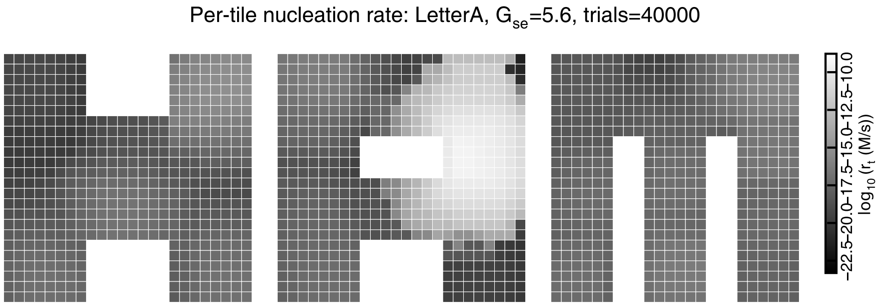

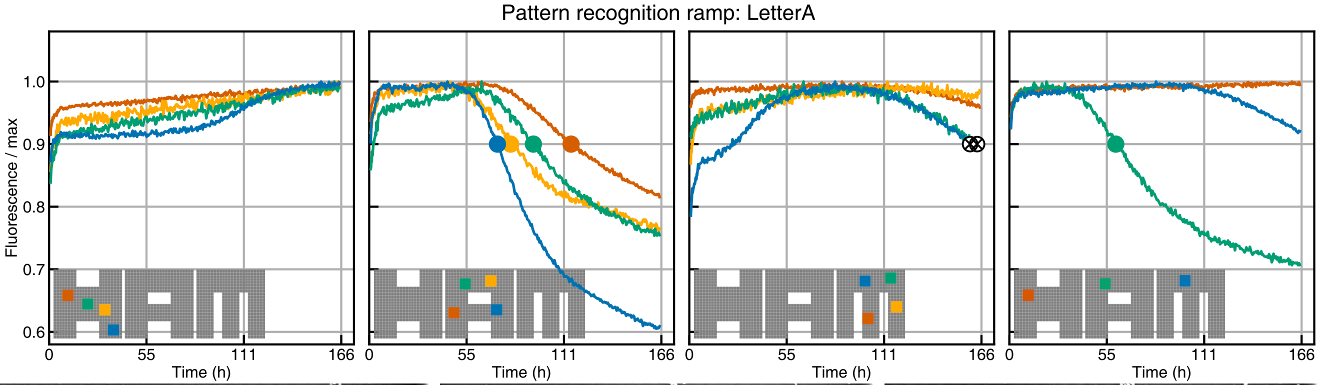

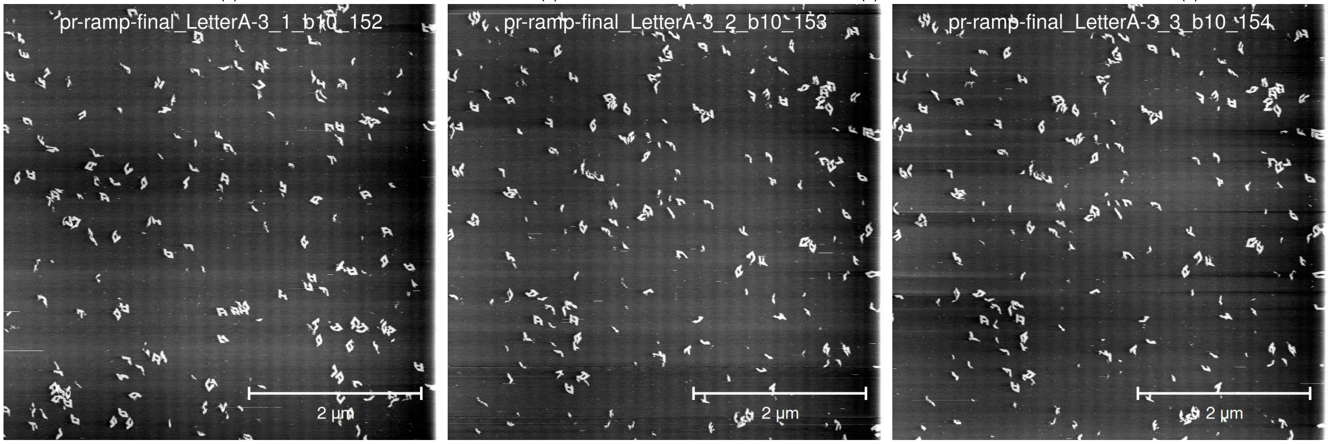

6.4.18 LetterM

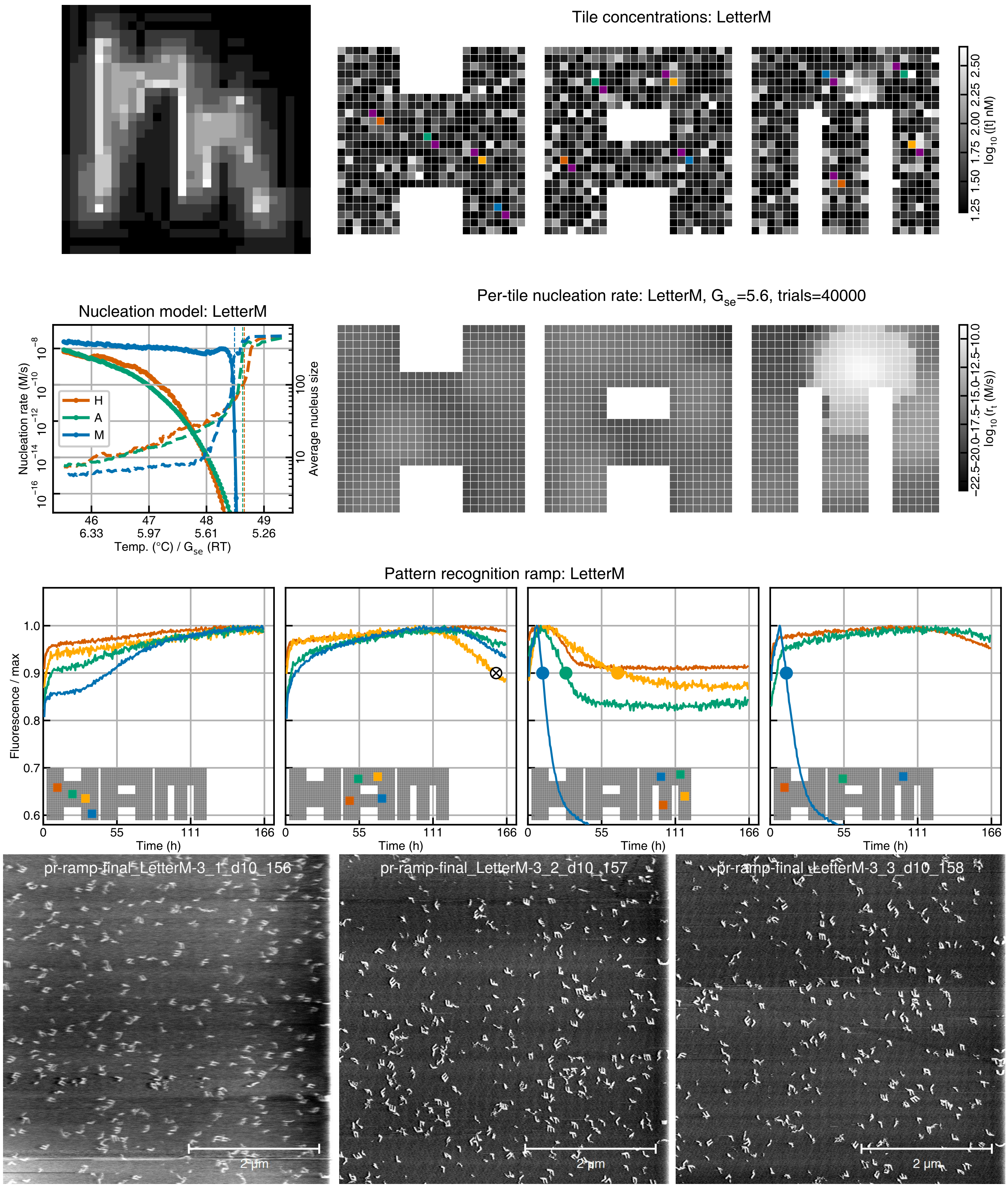

6.4.19 NoisyApple1

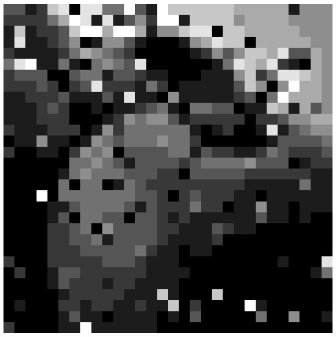

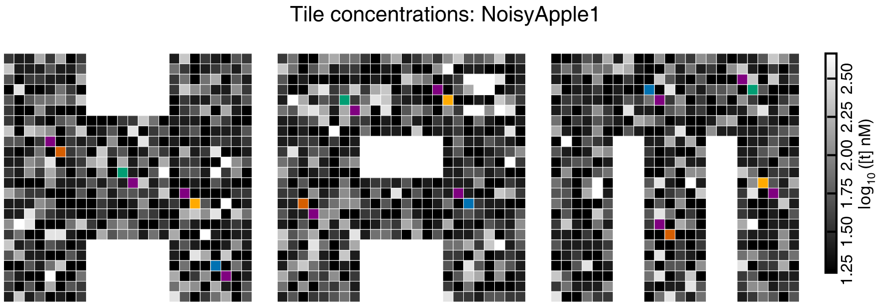

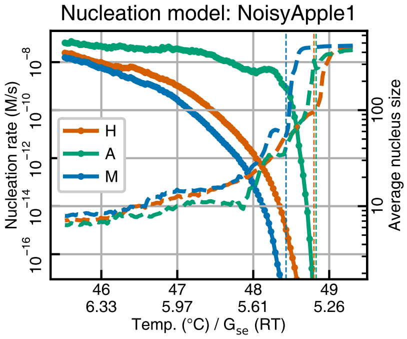

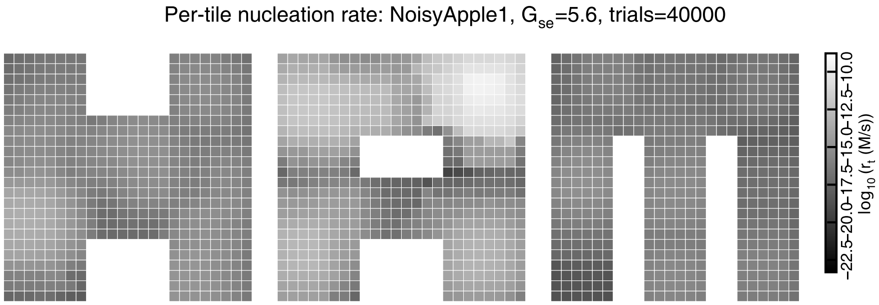

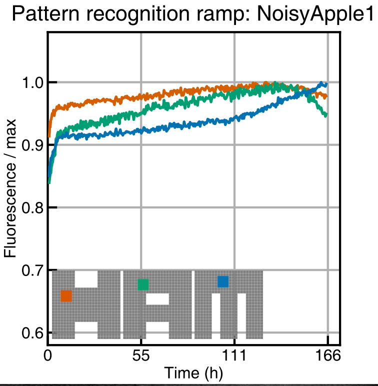

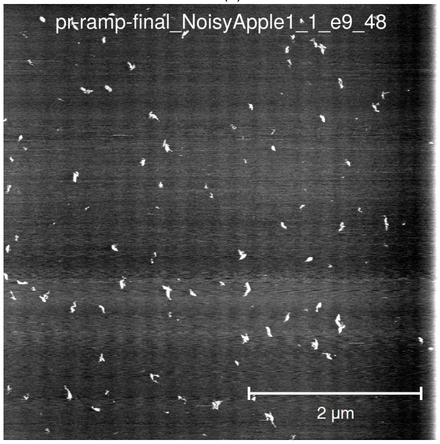

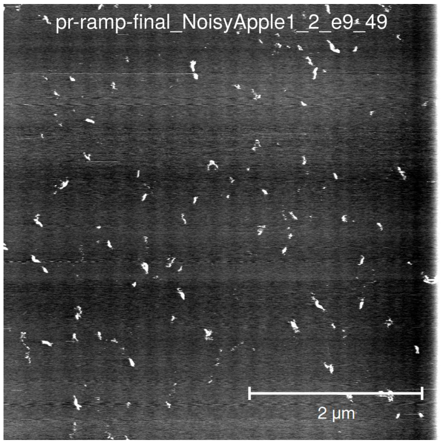

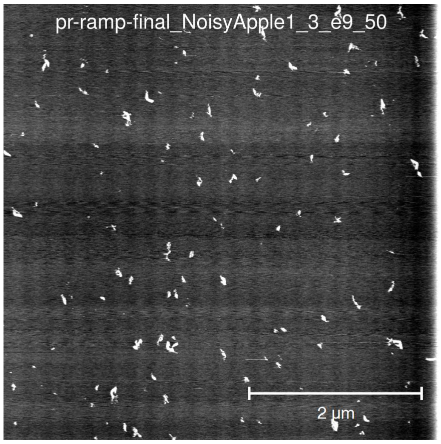

6.4.20 NoisyApple2

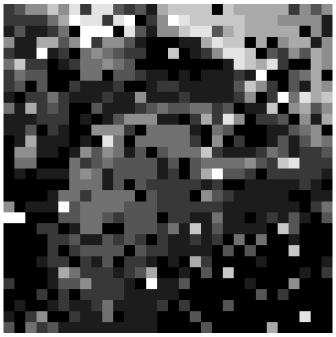

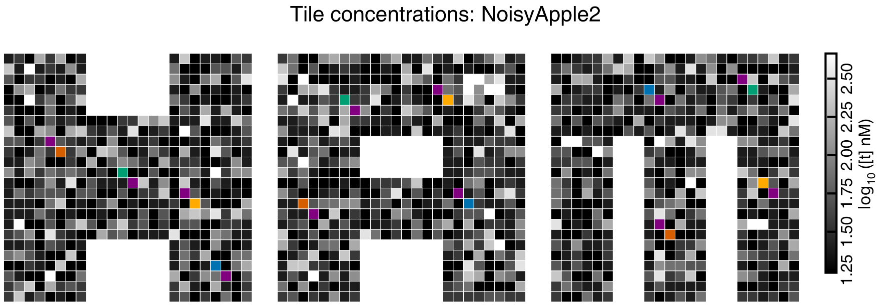

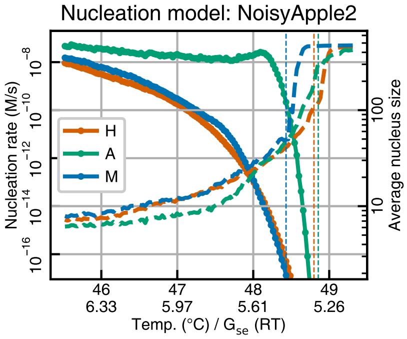

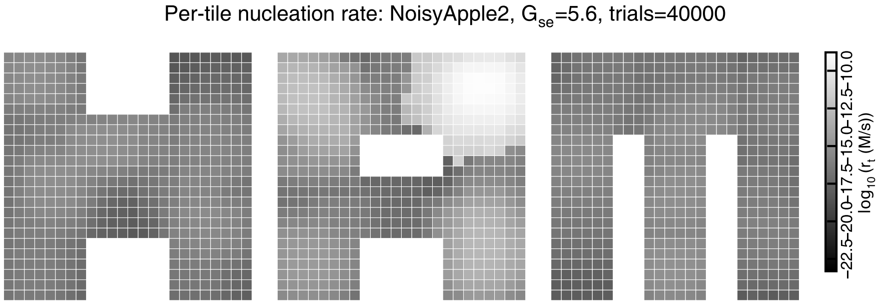

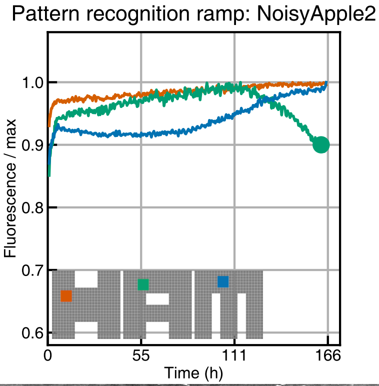

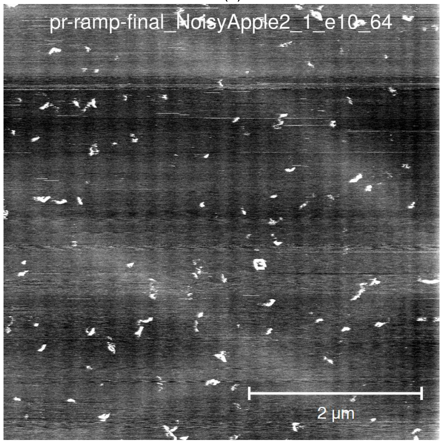

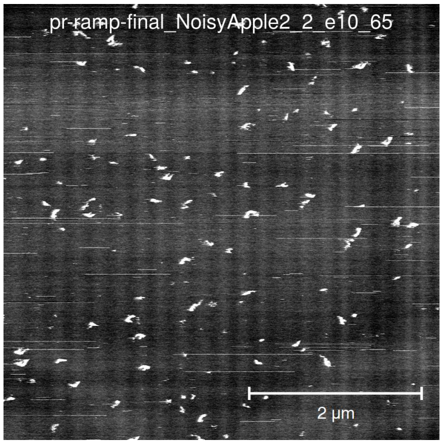

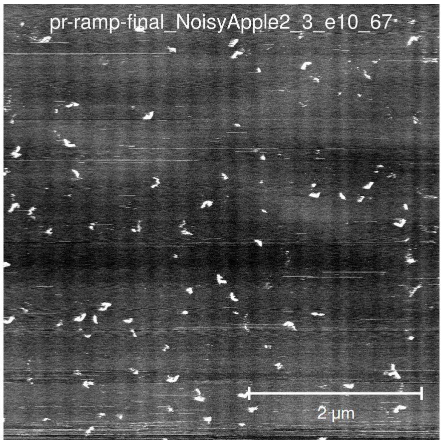

6.4.21 NoisyApple3

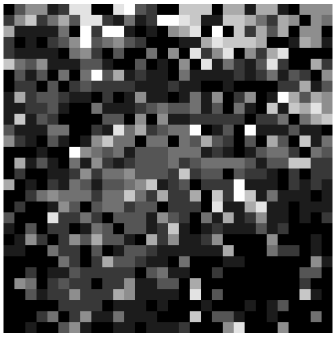

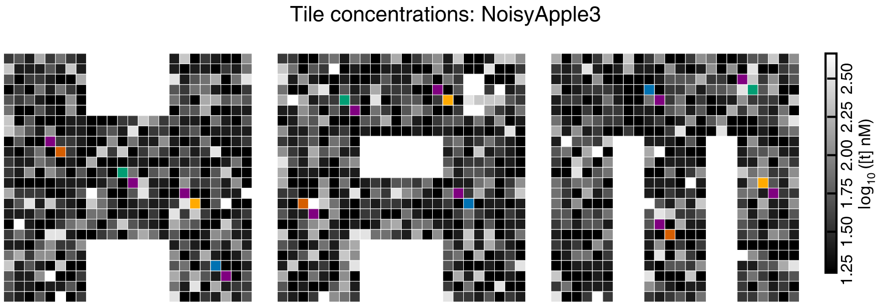

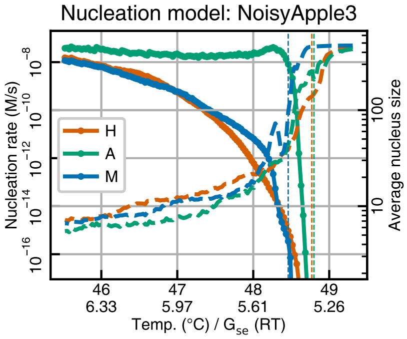

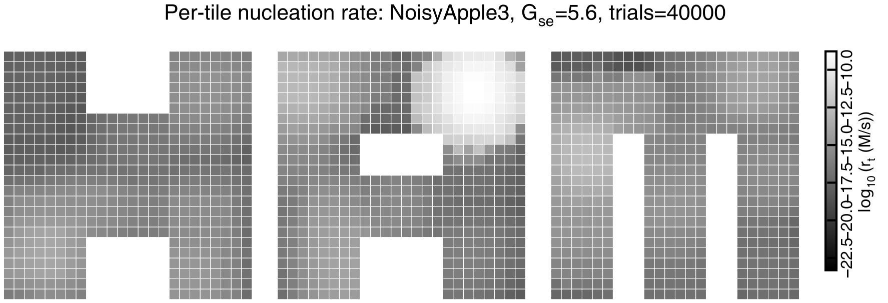

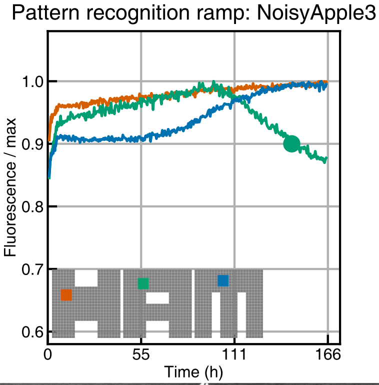

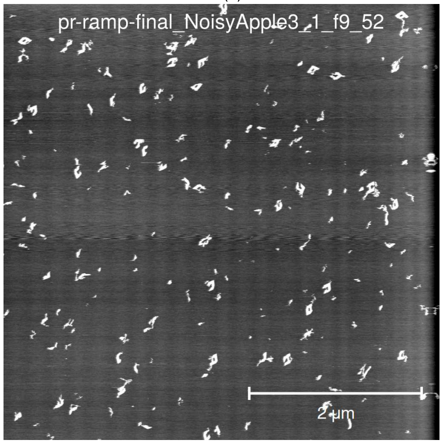

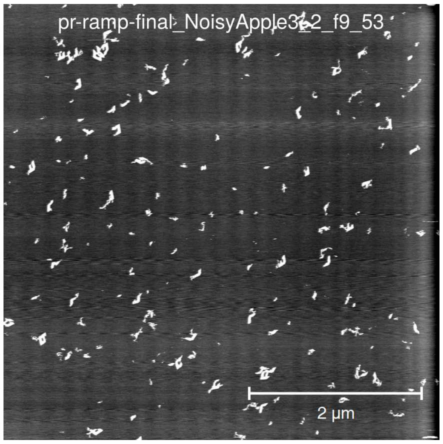

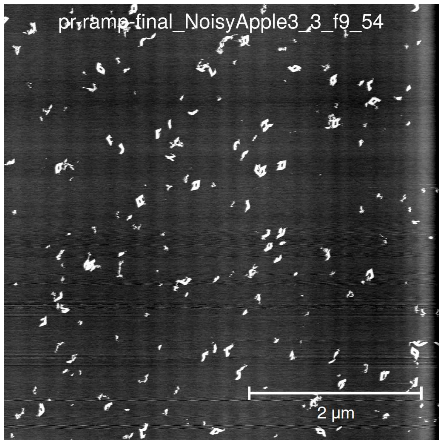

6.4.22 NoisyHorse1

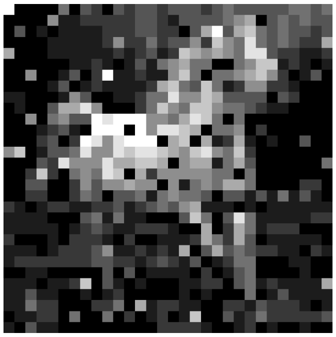

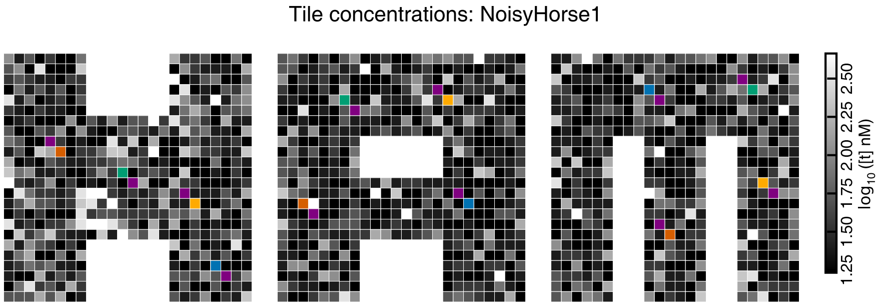

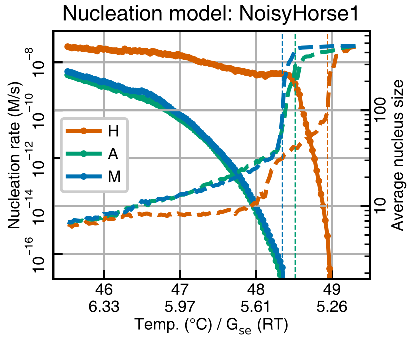

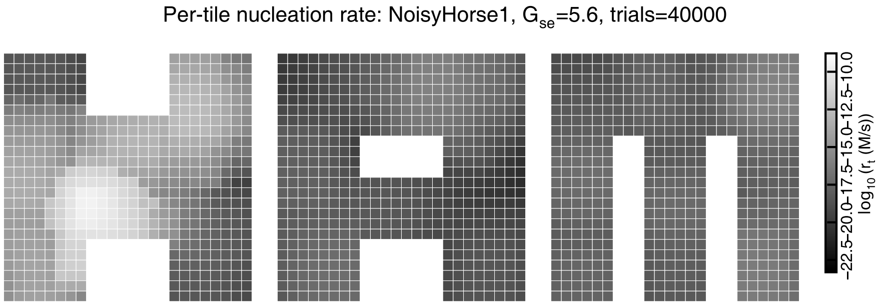

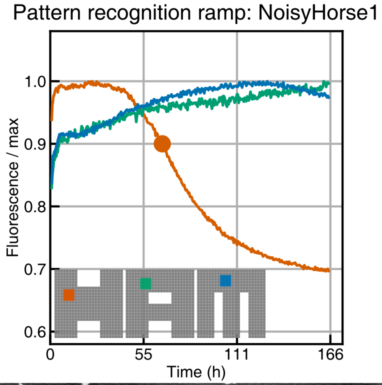

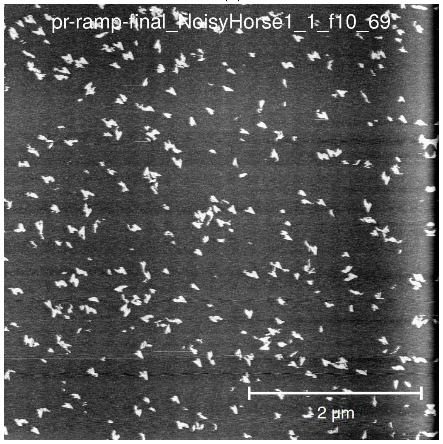

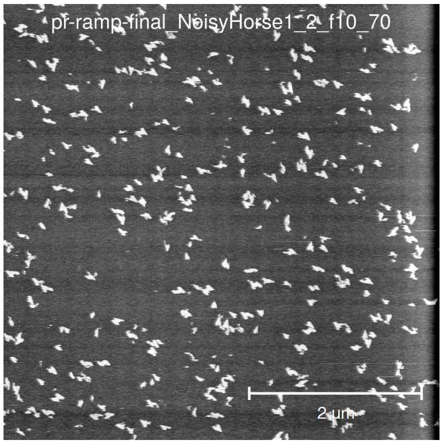

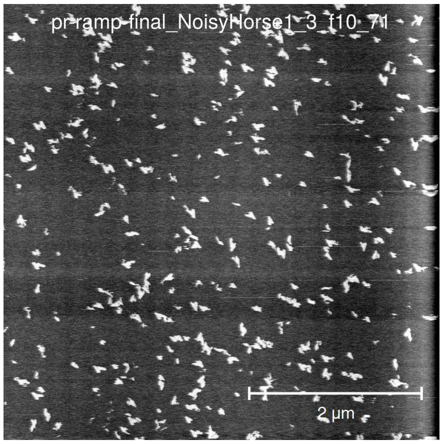

6.4.23 NoisyHorse2

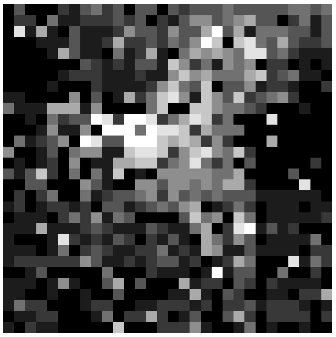

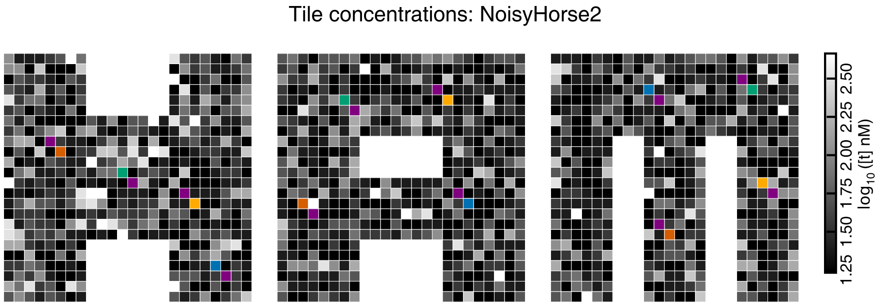

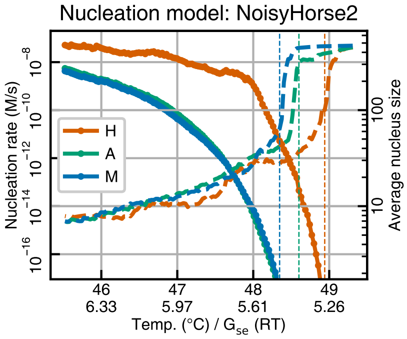

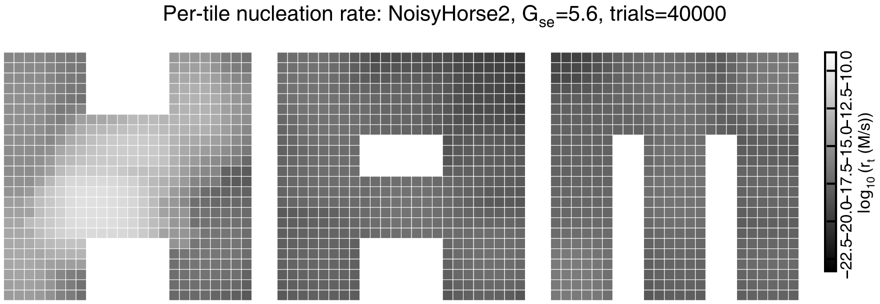

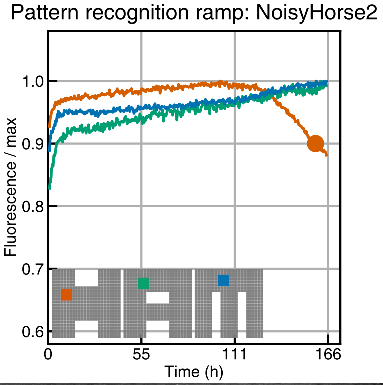

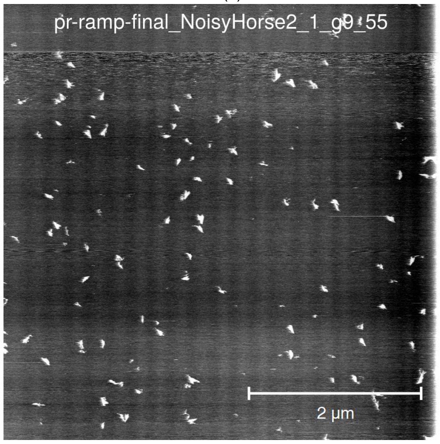

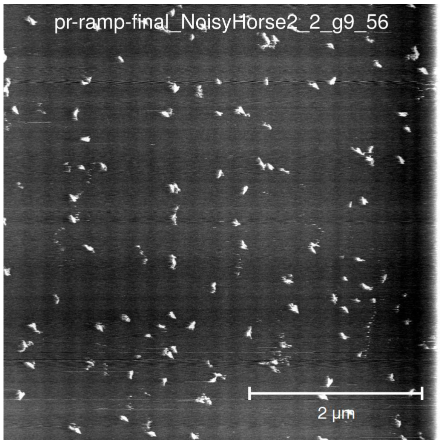

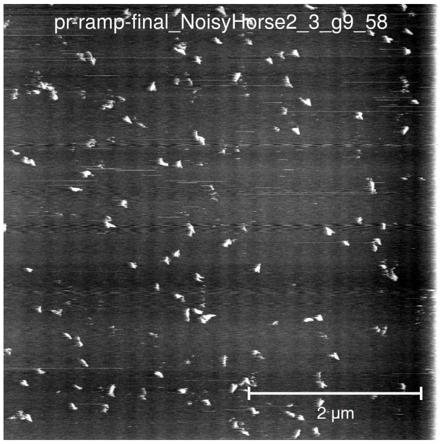

6.4.24 NoisyHorse3

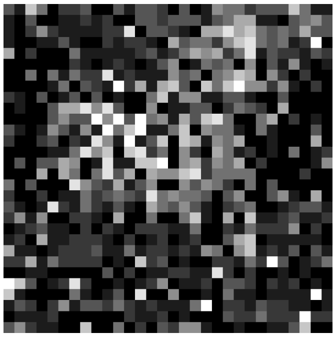

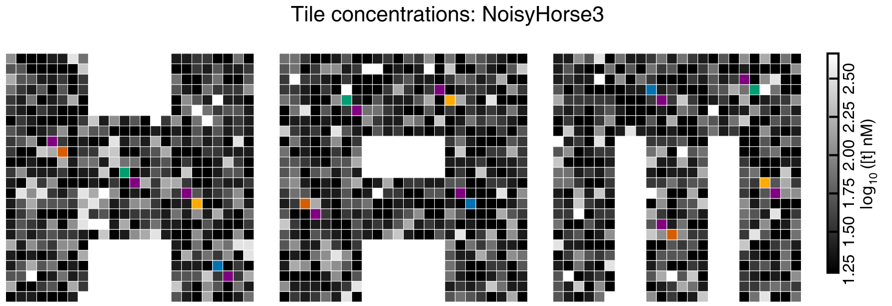

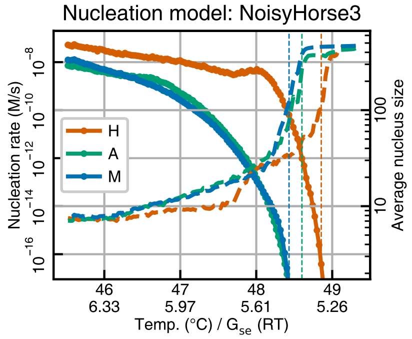

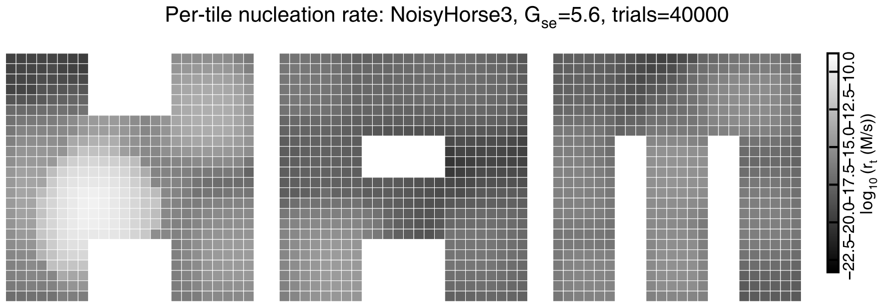

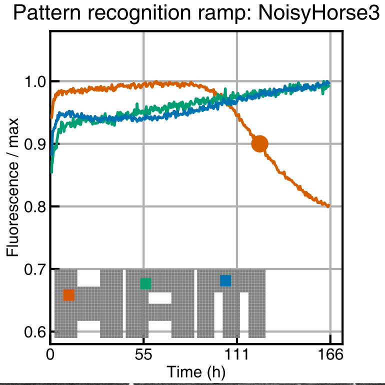

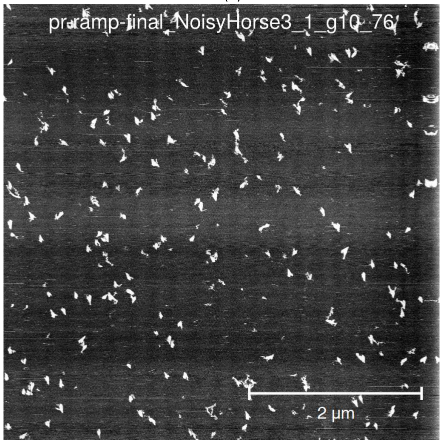

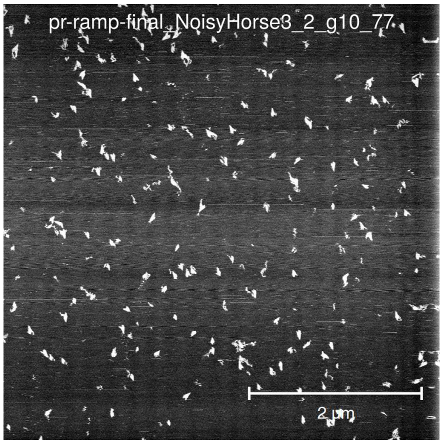

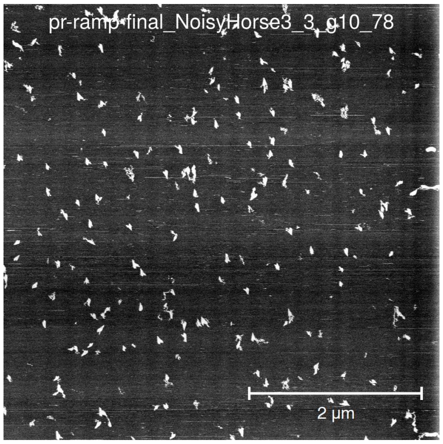

6.4.25 ObscMagn1

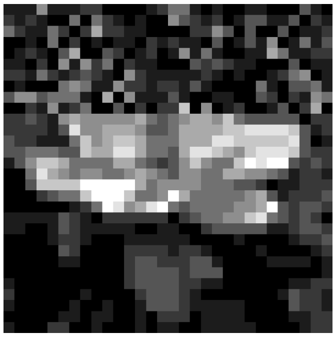

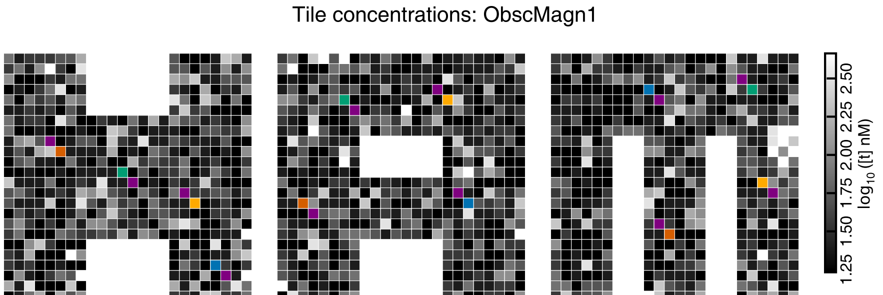

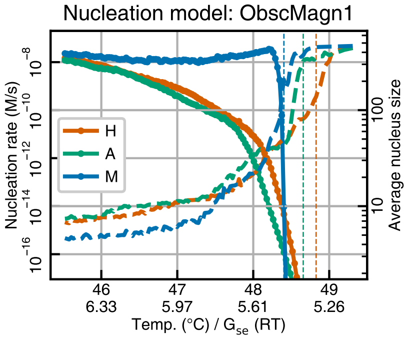

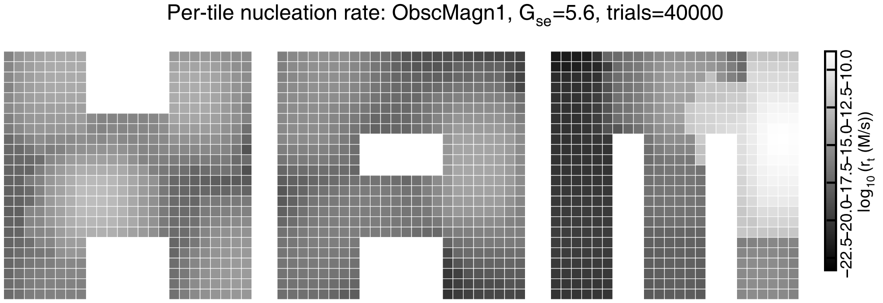

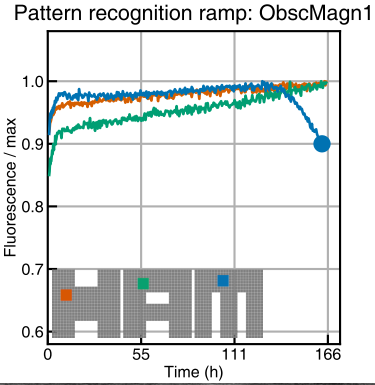

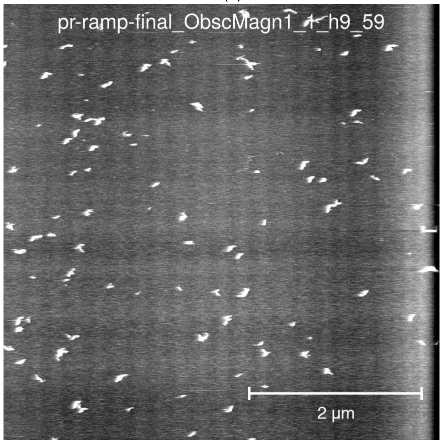

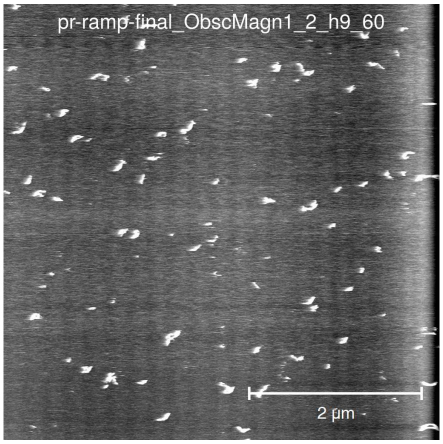

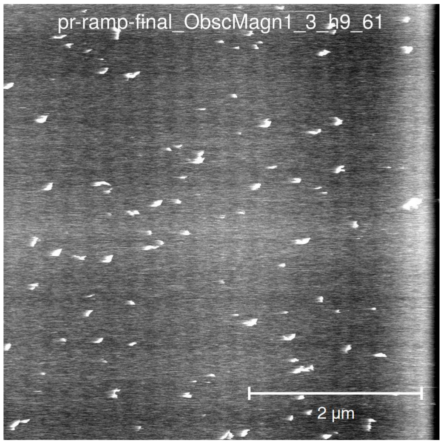

6.4.26 ObscMagn2

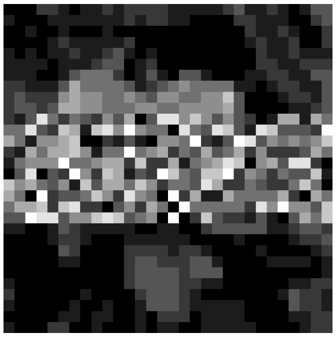

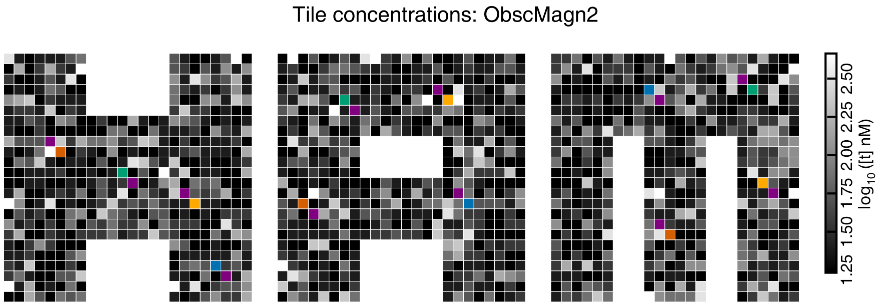

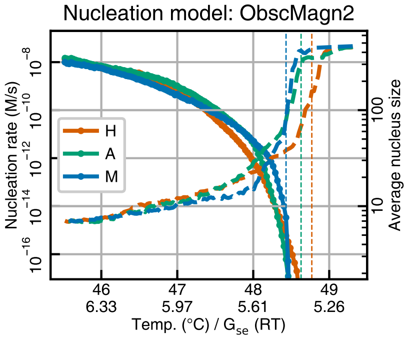

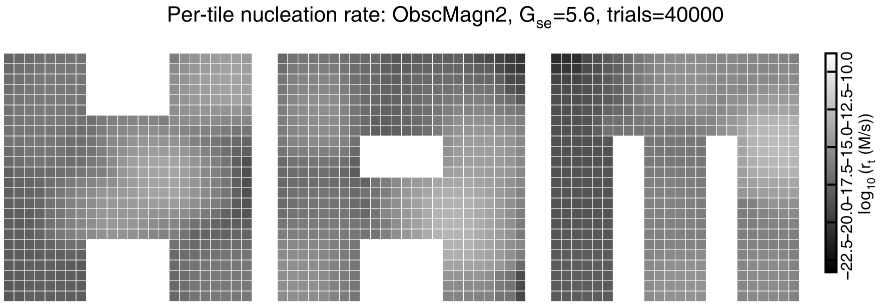

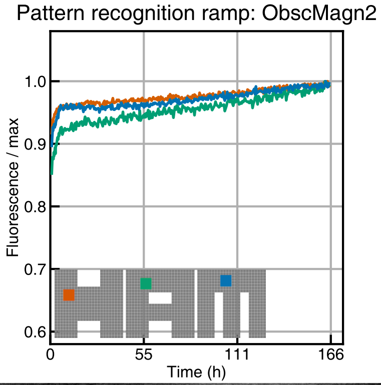

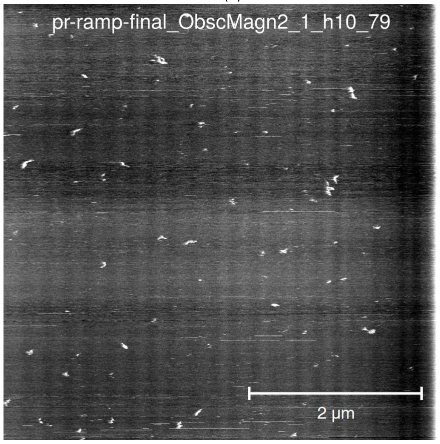

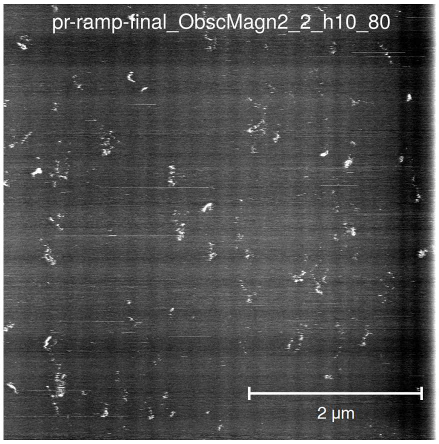

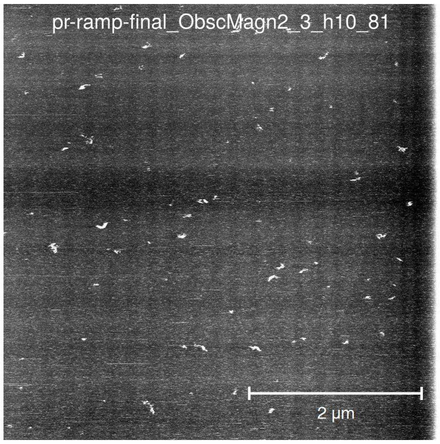

6.4.27 ObscMagn3

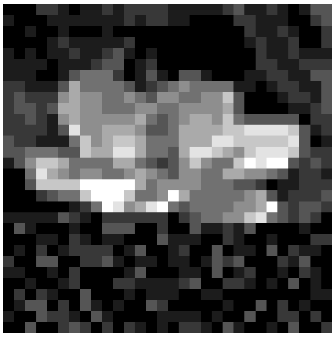

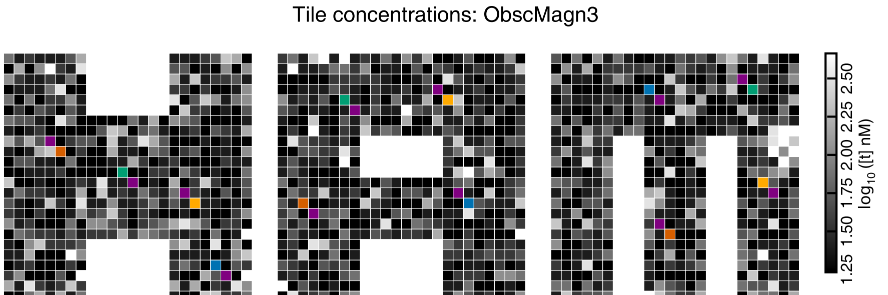

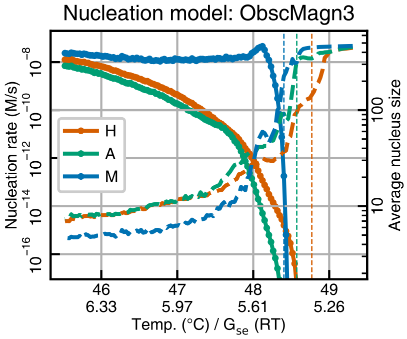

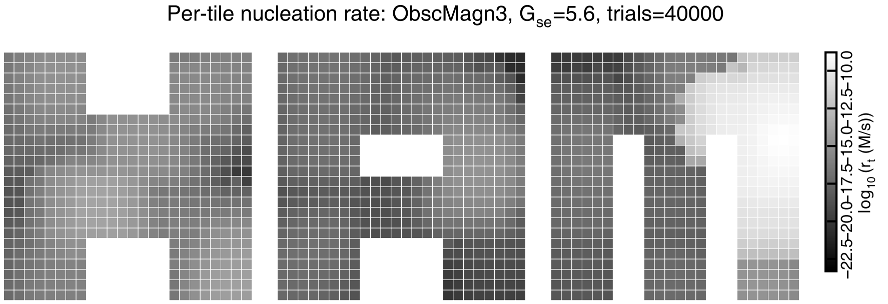

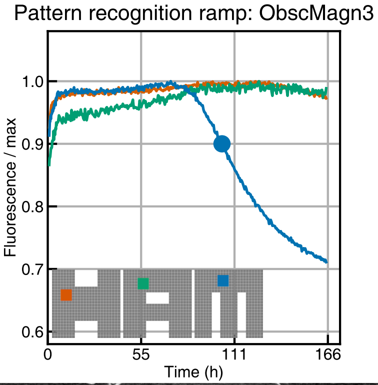

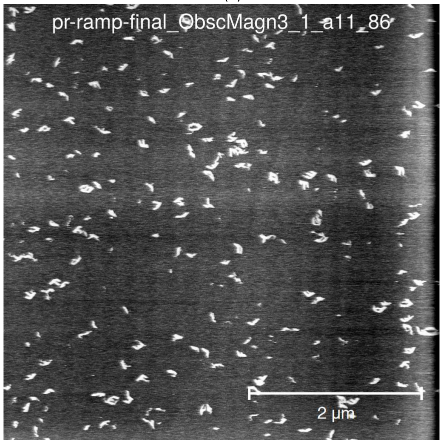

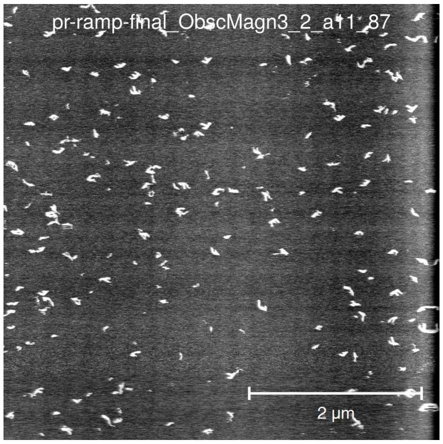

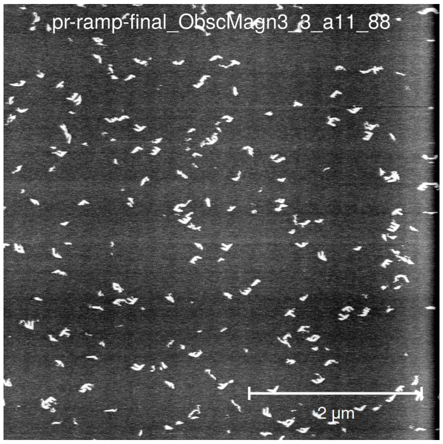

6.4.28 ObscHazeln1

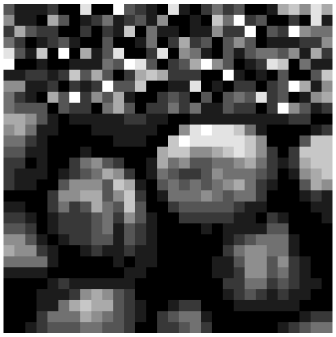

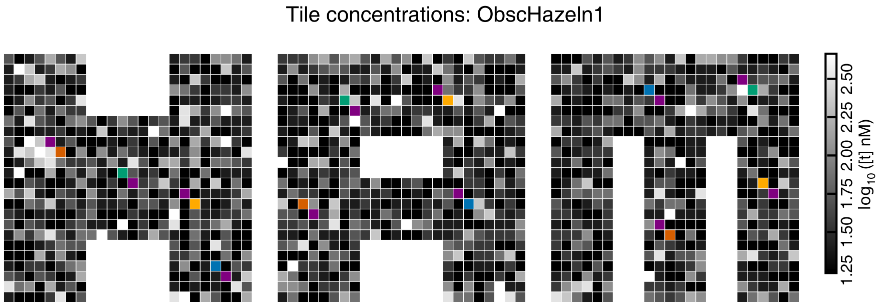

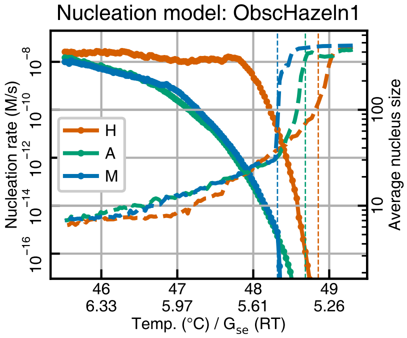

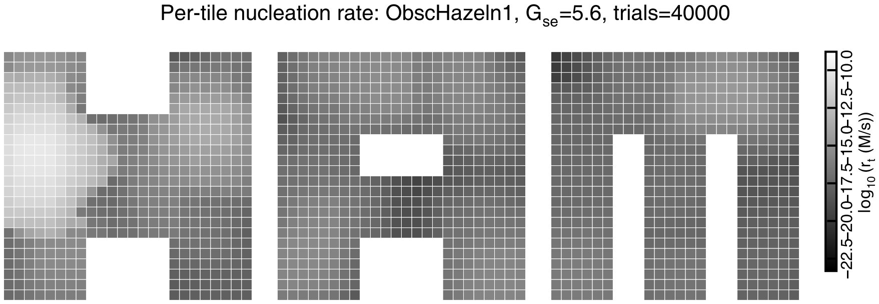

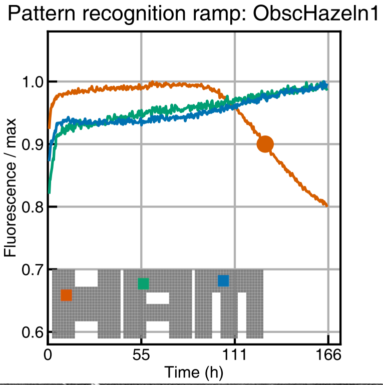

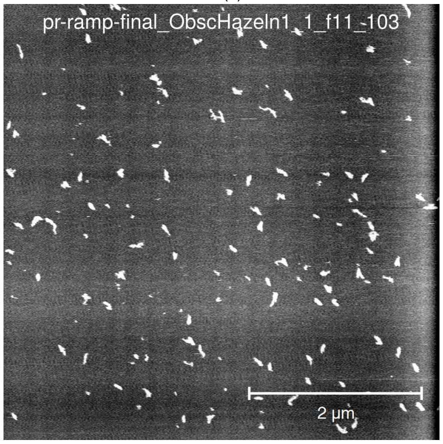

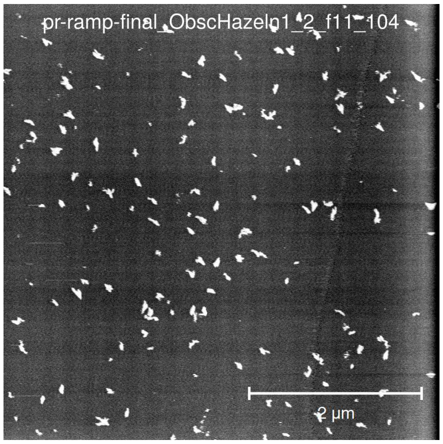

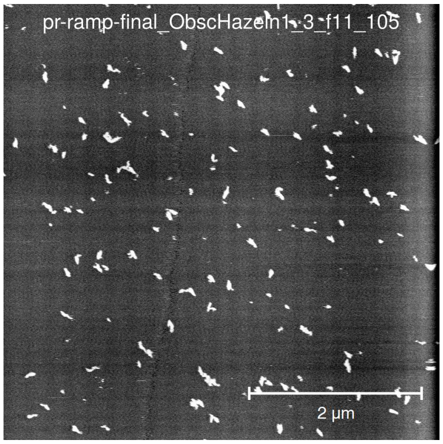

6.4.29 ObscHazeln2

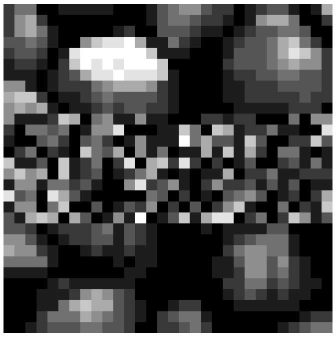

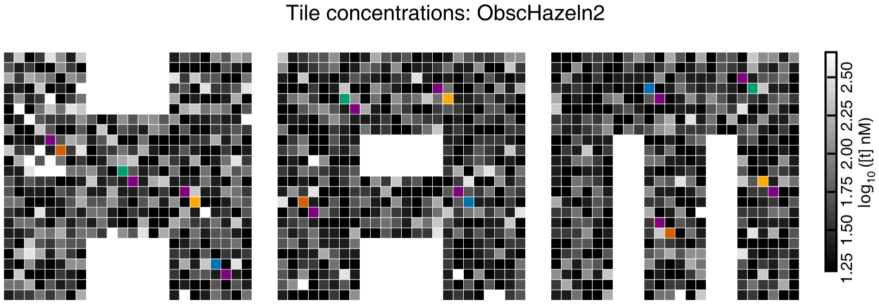

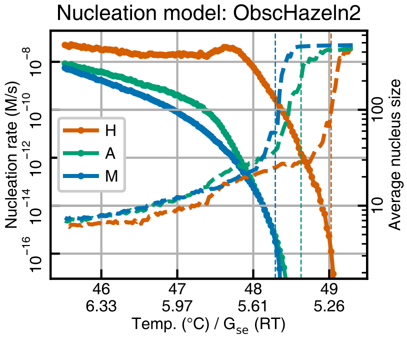

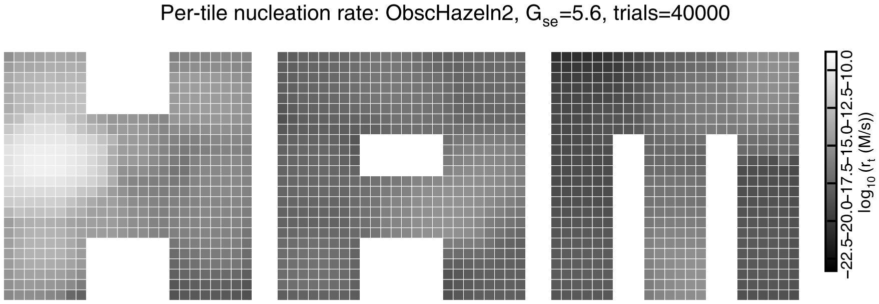

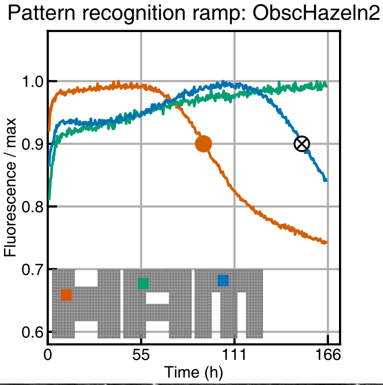

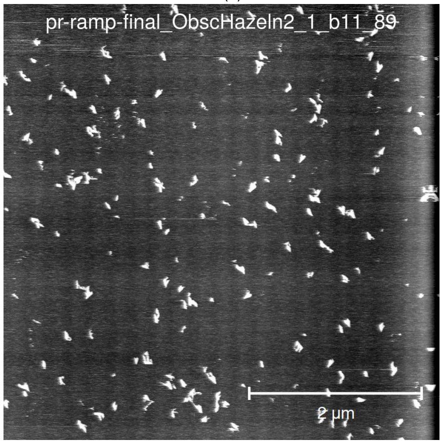

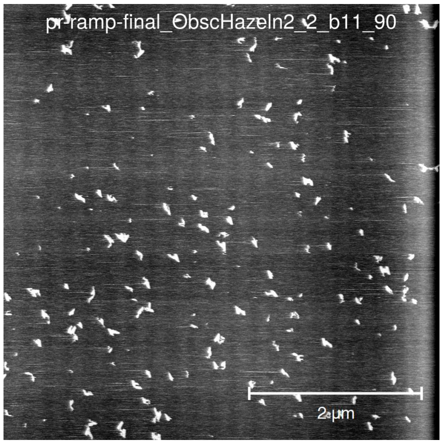

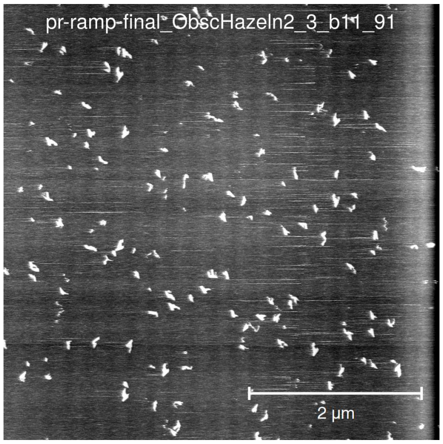

6.4.30 ObscHazeln3

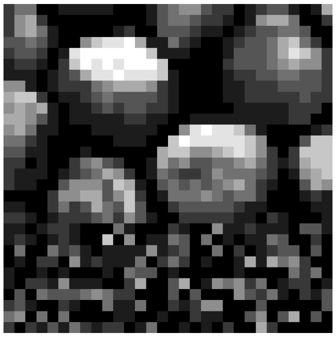

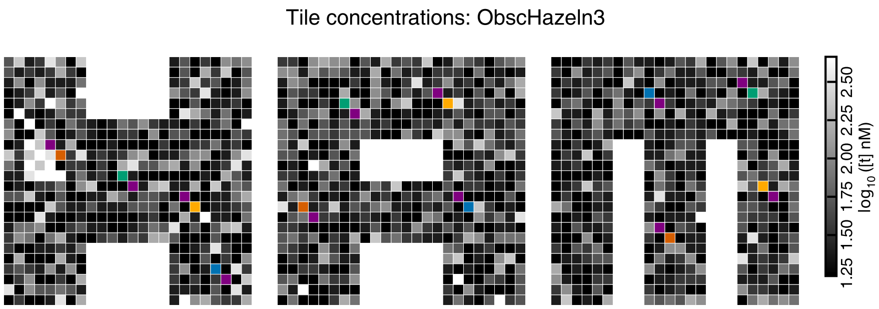

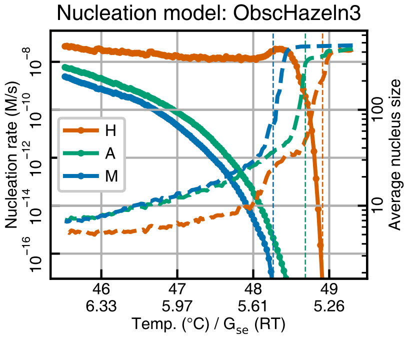

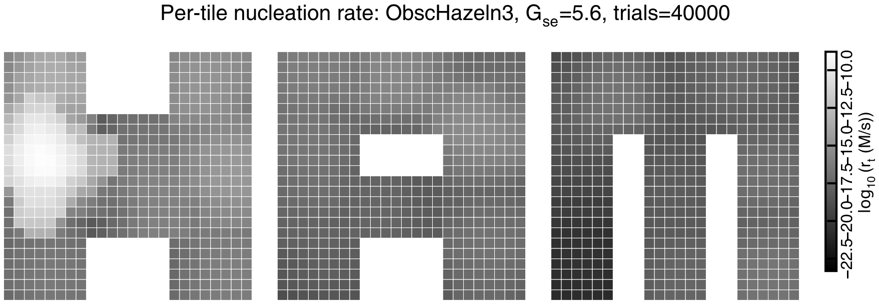

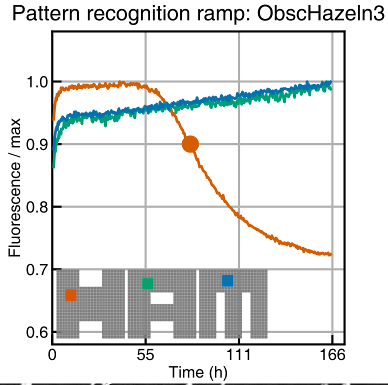

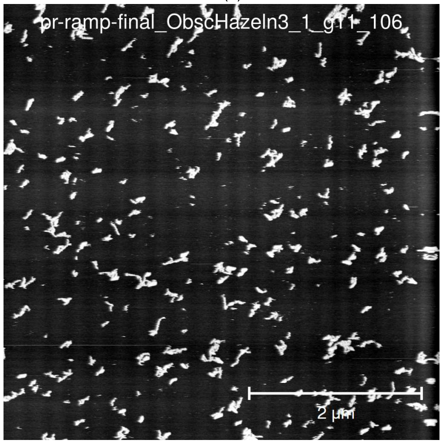

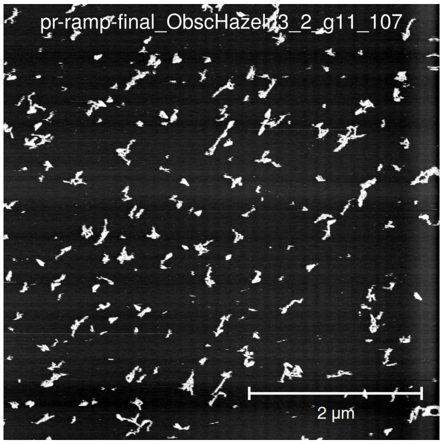

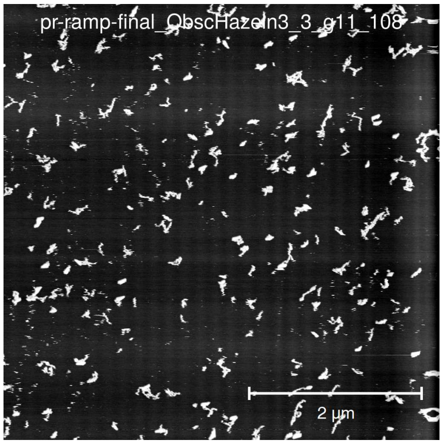

6.4.31 VarHarom1

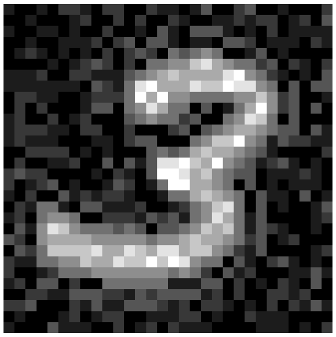

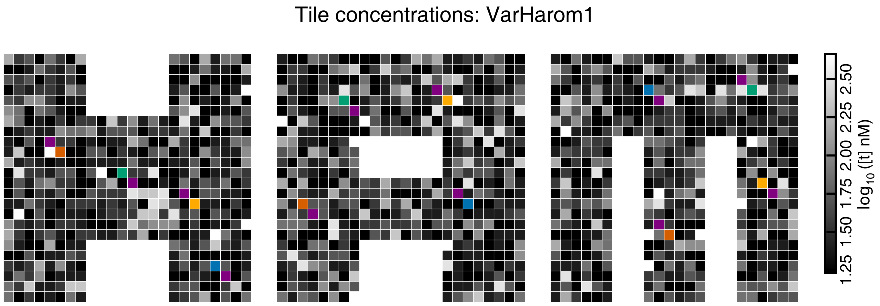

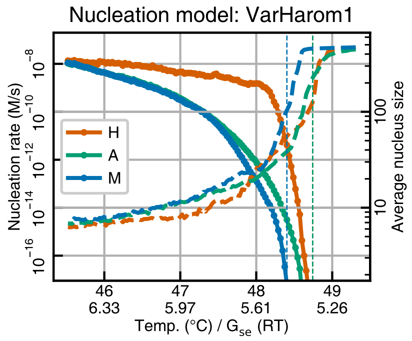

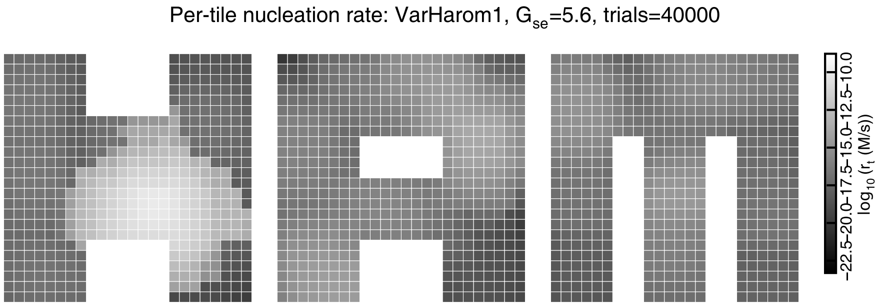

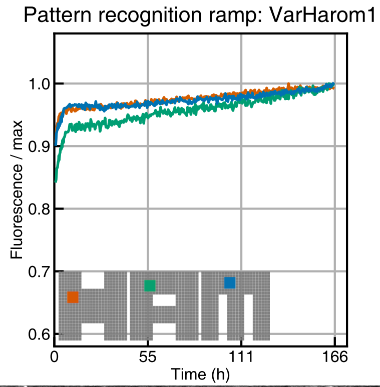

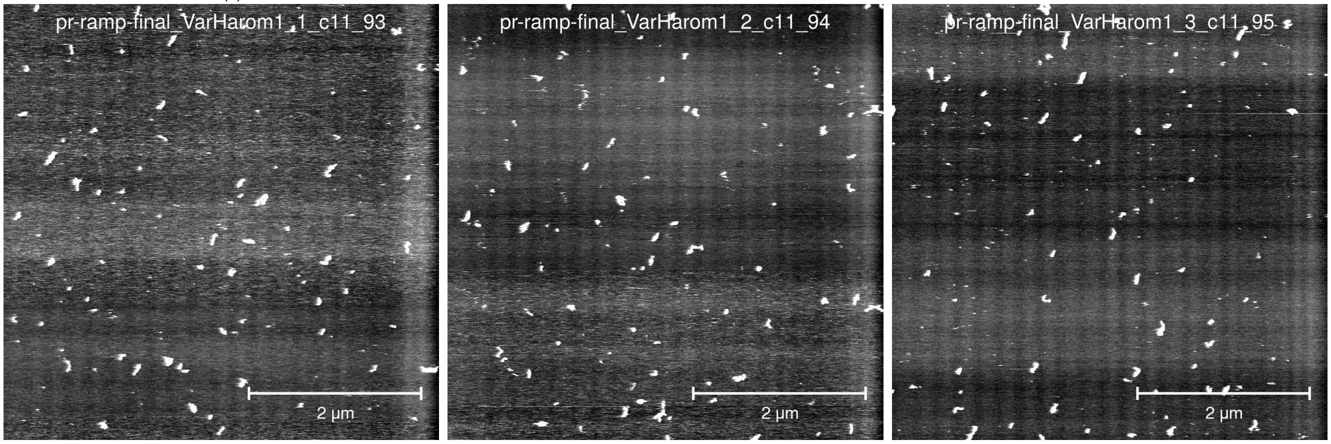

6.4.32 VarHarom2

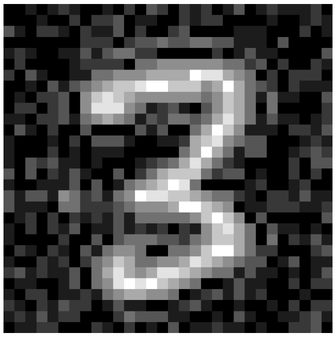

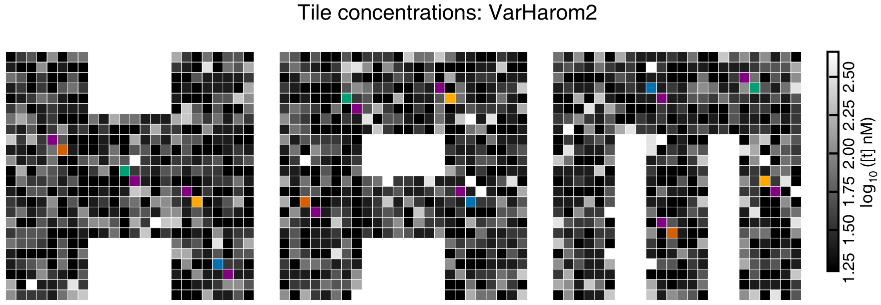

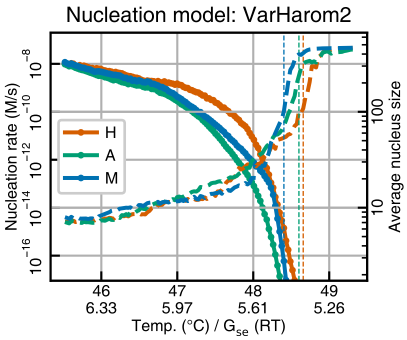

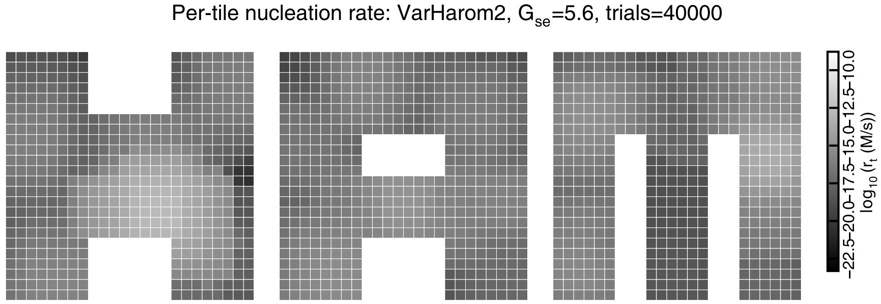

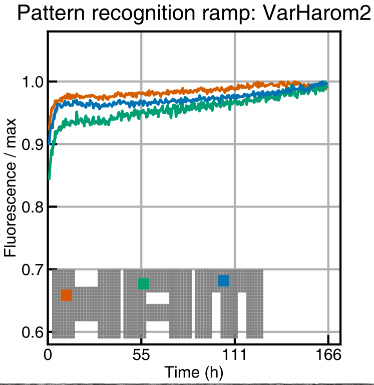

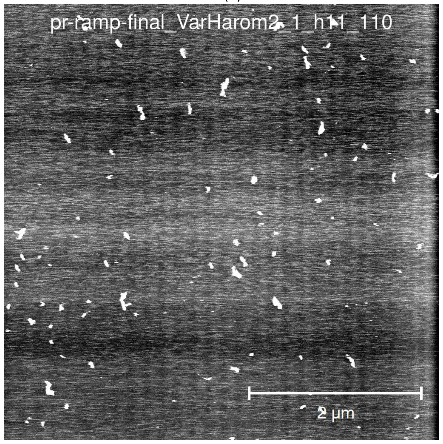

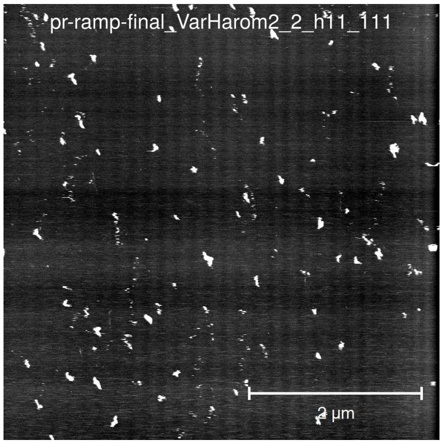

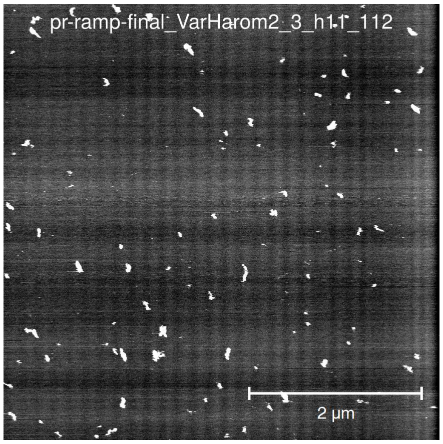

6.4.33 VarHarom3

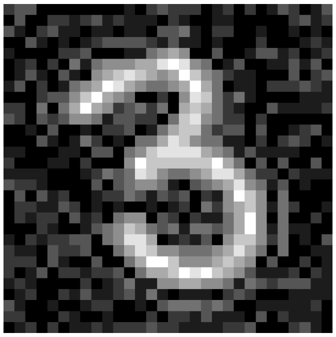

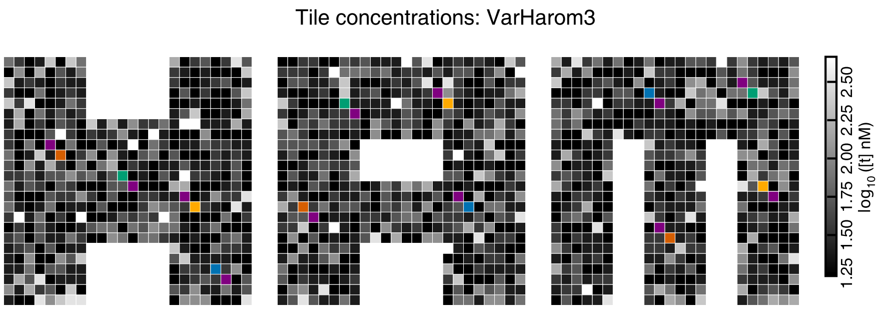

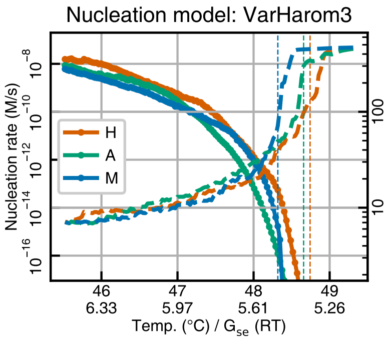

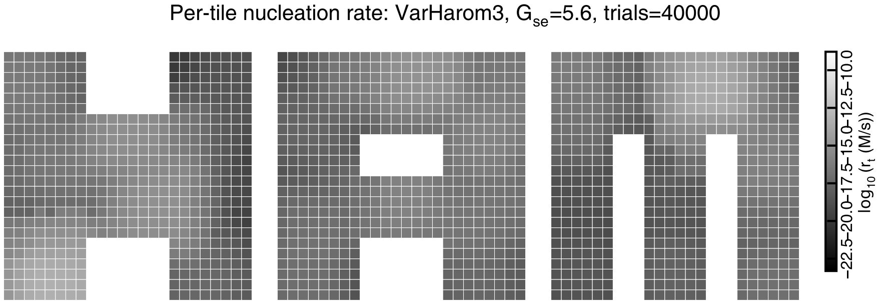

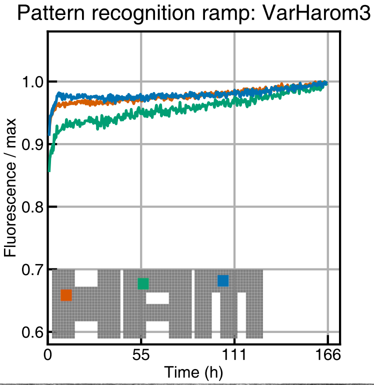

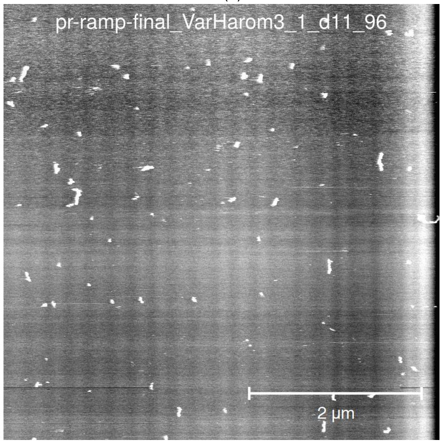

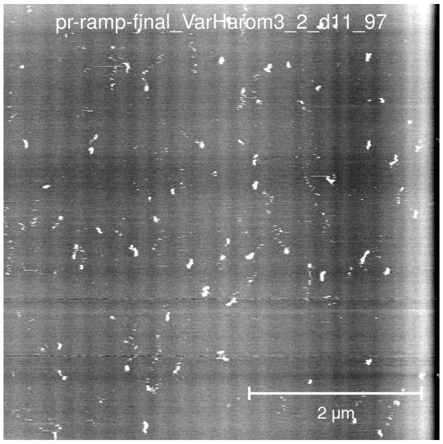

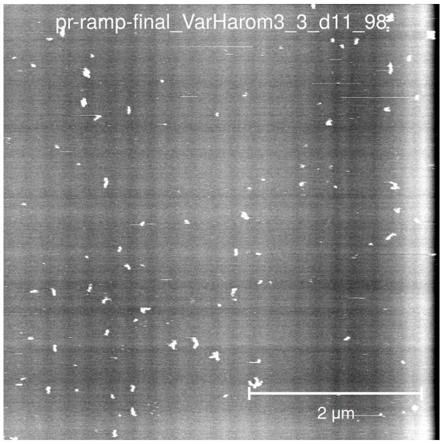

6.4.34 VarHarom4

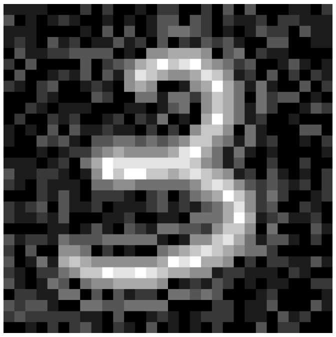

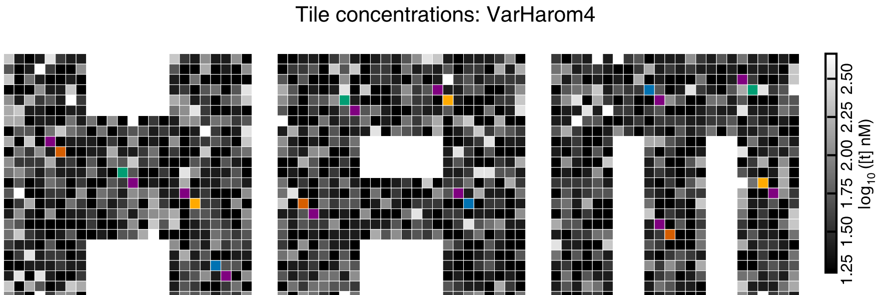

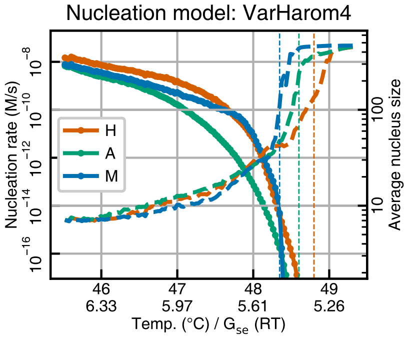

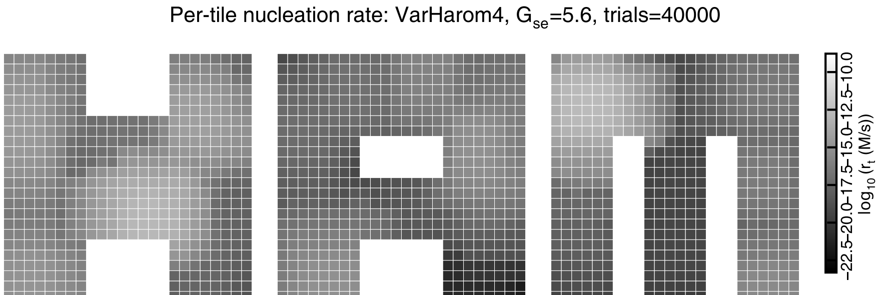

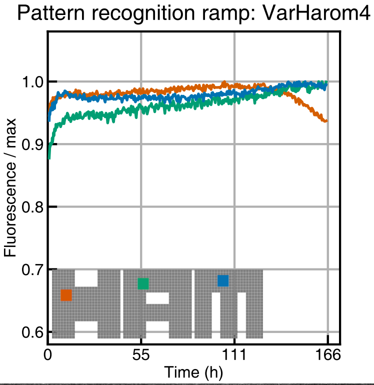

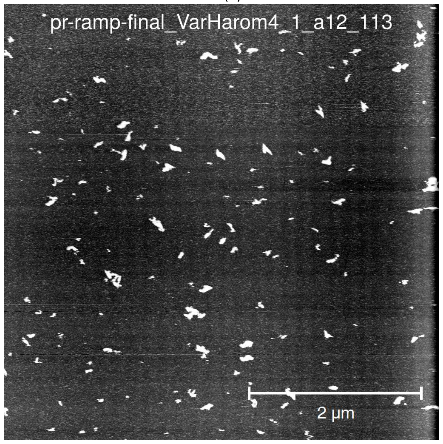

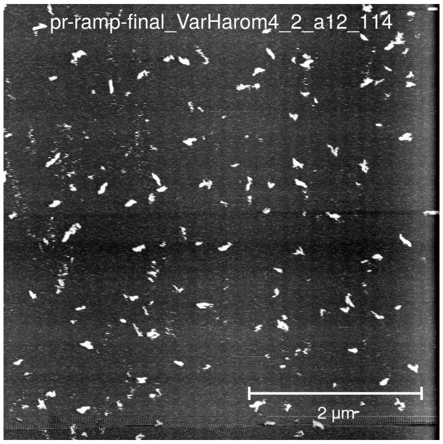

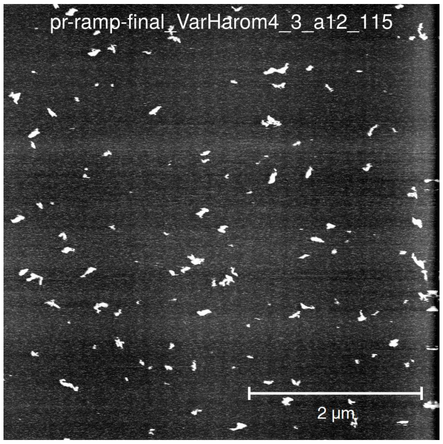

6.4.35 VarHarom5

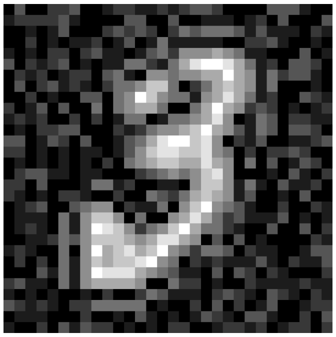

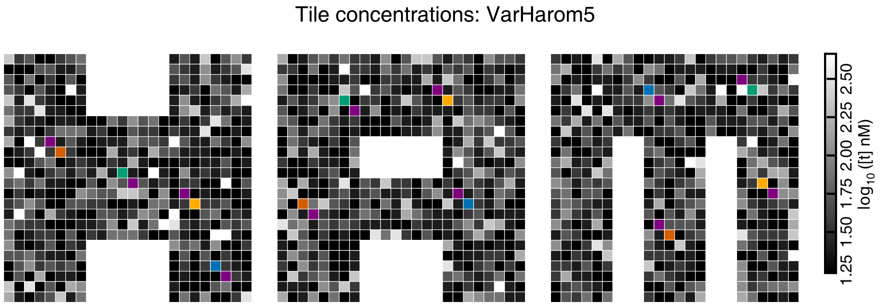

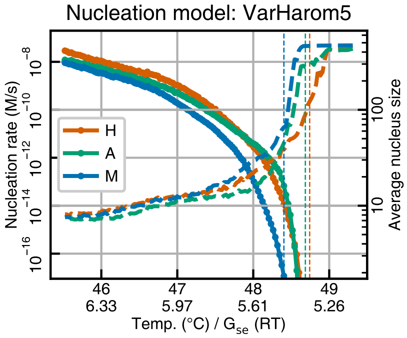

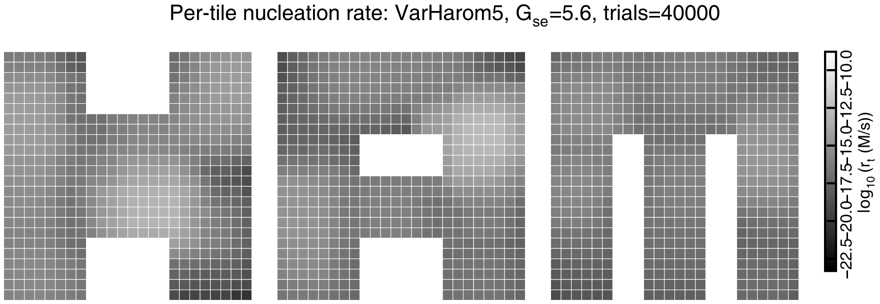

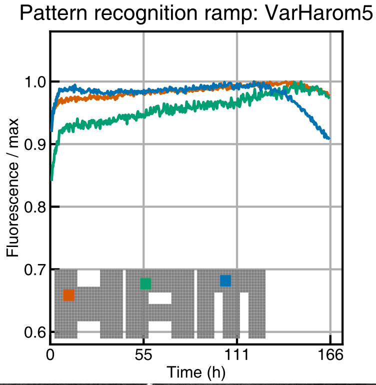

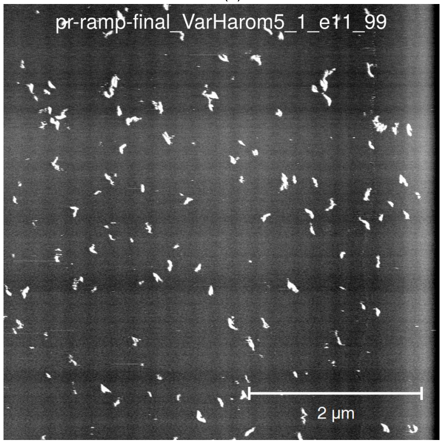

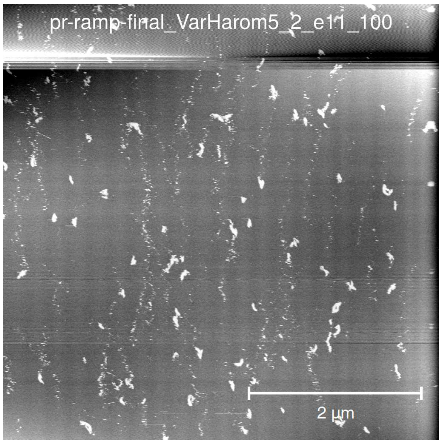

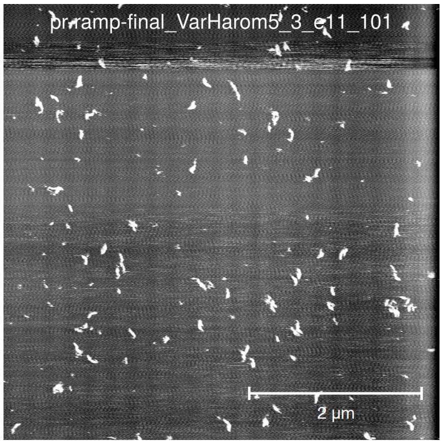

6.4.36 VarHarom6

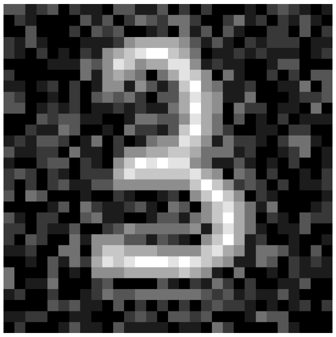

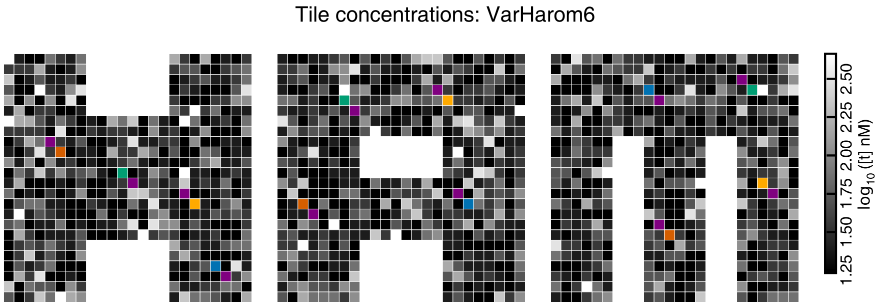

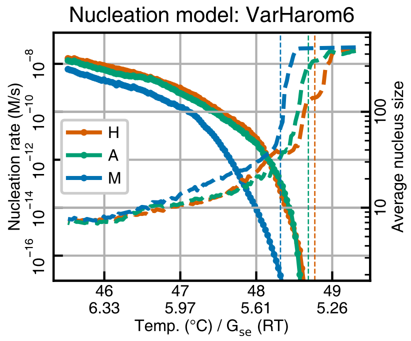

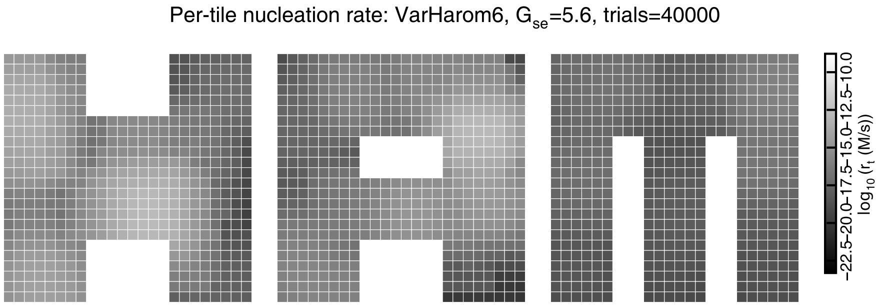

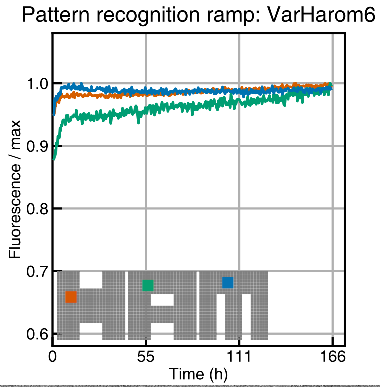

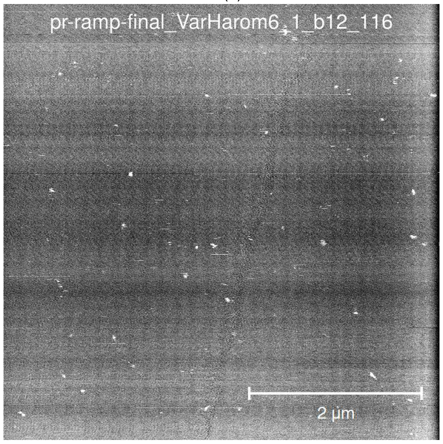

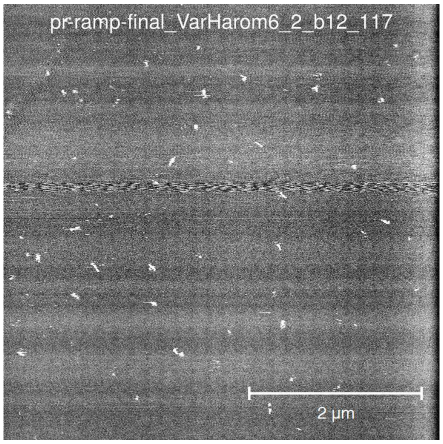

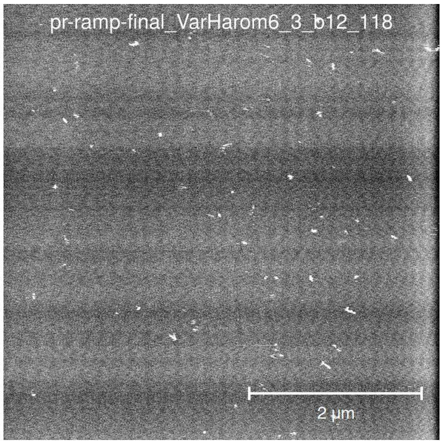

6.4.37 Uniform concentrations (60 nM)

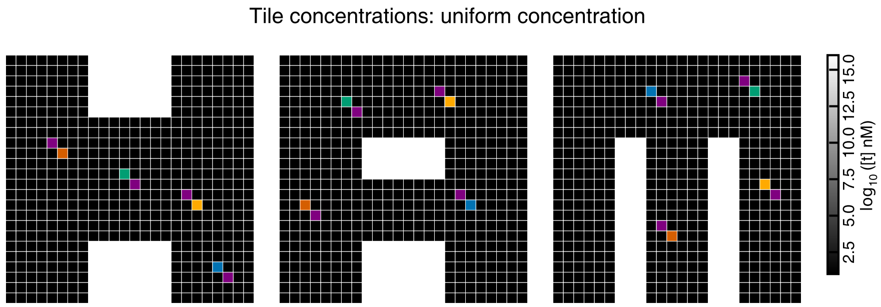

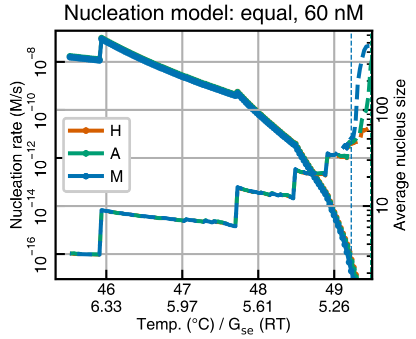

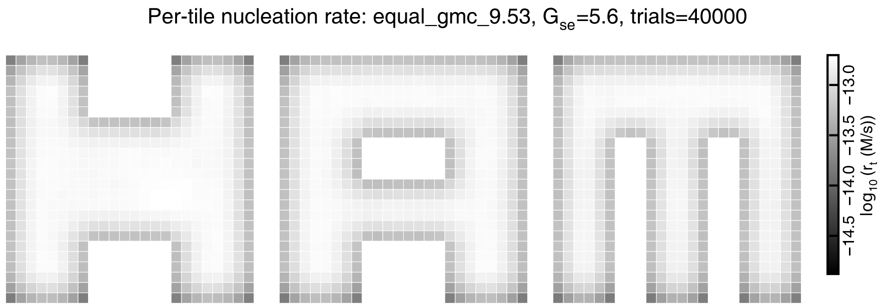

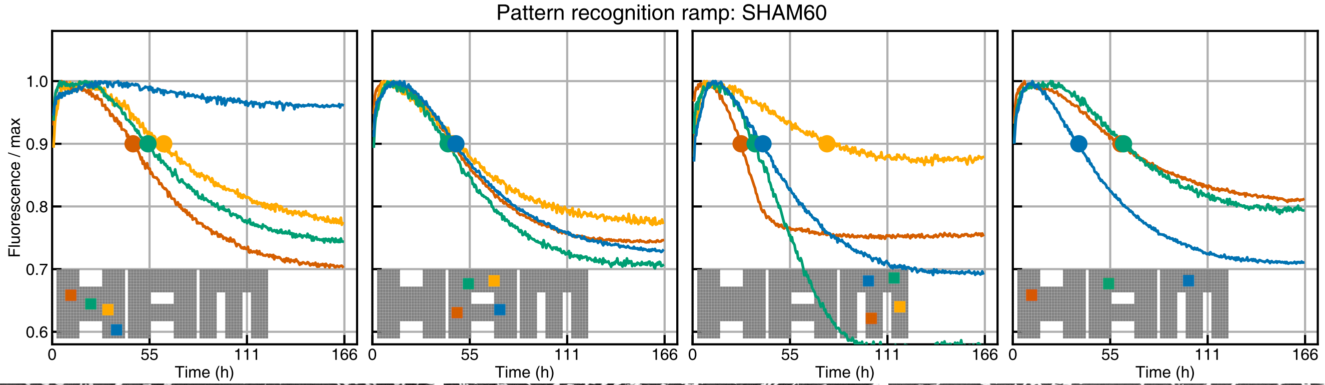

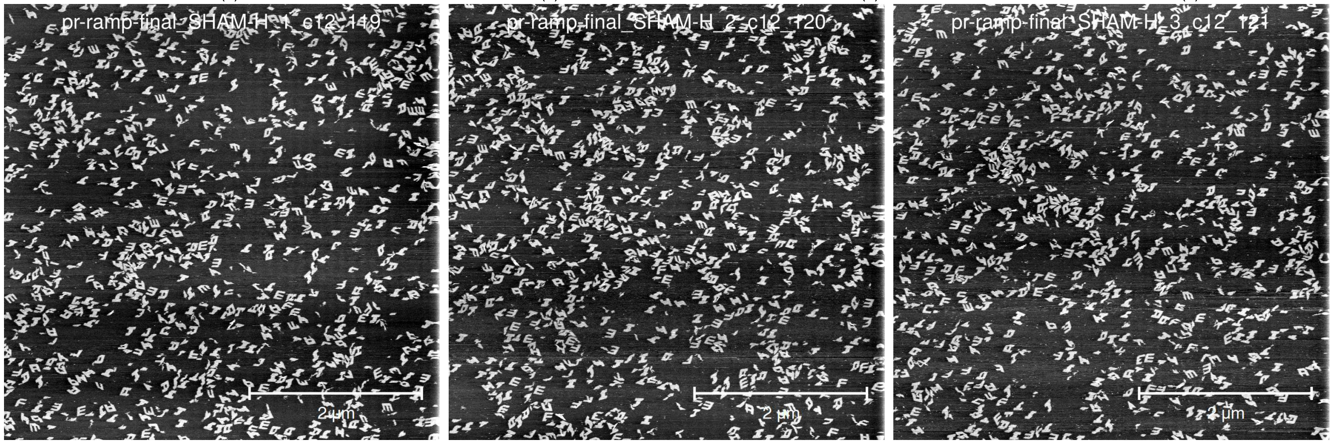

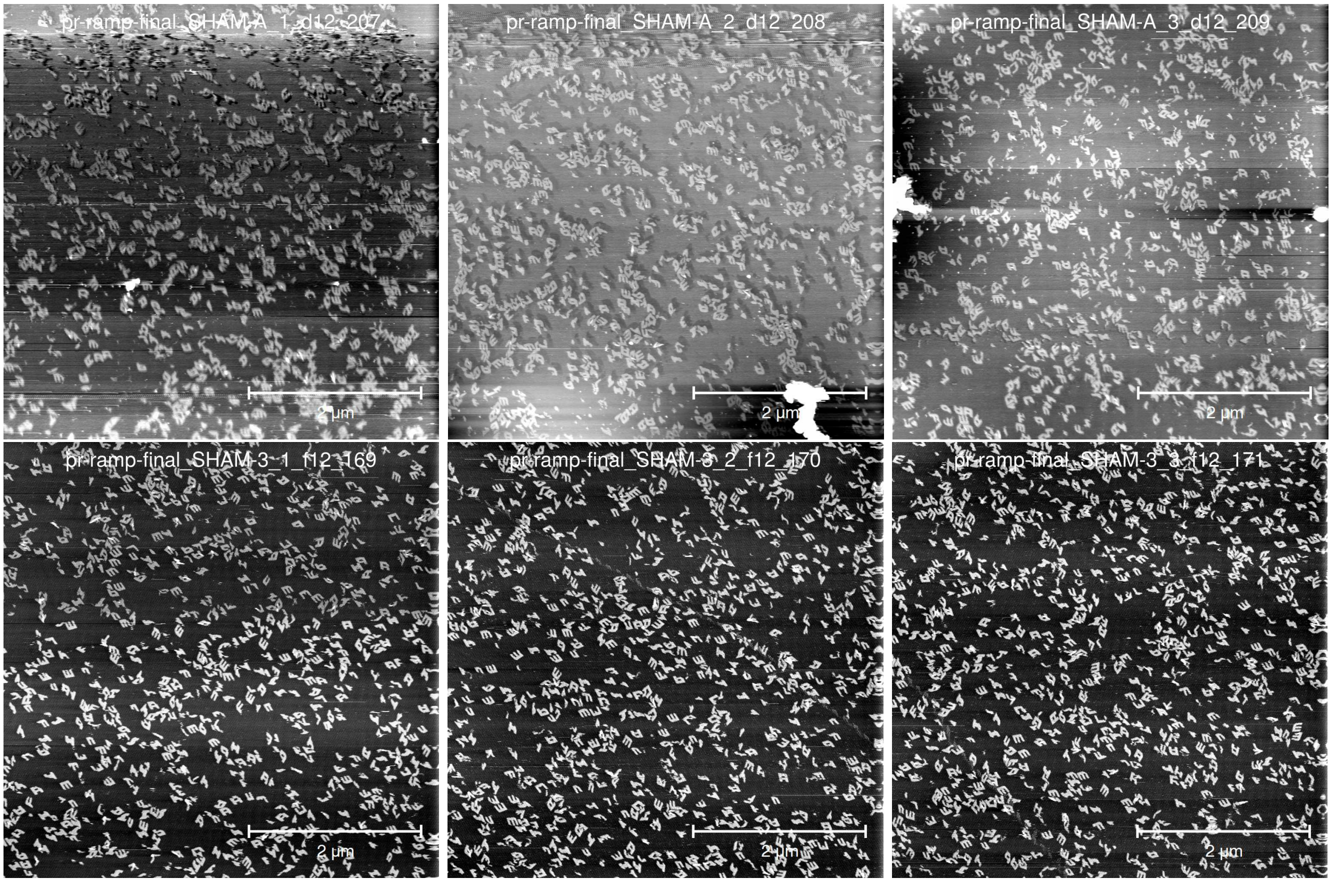
pr-ramp-final_SHAM-A_1_d12_207
2 µm
pr-ramp-final_SHAM-A_2_d12_208
2 µm
pr-ramp-final_SHAM-A_3_d12_209
2 µm
pr-ramp-final_SHAM-3_1_f12_169
2 µm
pr-ramp-final_SHAM-3_2_f12_170
2 µm
pr-ramp-final_SHAM-3_3_f12_171
2 µm
